# Chrysosplenetin-induced TMED3 aggregation triggers unfolded protein response in pancreatic cancer

**DOI:** 10.1038/s44321-026-00506-5

**Published:** 2026-08-13

**Authors:** Zhe Zhang, Ningna Weng, Xuanhao Gu, Yi Zang, Qitai Chen, Rujia Zheng, Xishan Yang, Gubu Amu, Danyang Zhao, Zhihao Ma, Jinyan Huang, Tingbo Liang, Qi Zhang

**Affiliations:** 1 MOE Joint International Research Laboratory of Pancreatic Diseases, Zhejiang Provincial Key Laboratory of Pancreatic Disease, The First Affiliated Hospital, Zhejiang University School of Medicine, Hangzhou, 310003 China; 2 Department of Medical Oncology, Clinical Oncology School of Fujian Medical University, Fujian Cancer Hospital, Fujian, 350011 China; 3 Department of Hepatobiliary and Pancreatic Surgery, The First Affiliated Hospital, Zhejiang University School of Medicine, Hangzhou, 310003 China; 4 Department of Clinical Pharmacy, Zhejiang Provincial Key Laboratory for Drug Evaluation and Clinical Research, The First Affiliated Hospital, Zhejiang University School of Medicine, Hangzhou, 310003 China; 5 Center for Biomedical Big Data, The First Affiliated Hospital, School of Medicine, Zhejiang University, Hangzhou, 310003 China; 6 Zhejiang University Cancer Center, Zhejiang University, Zhejiang Clinical Research Center of Hepatobiliary and Pancreatic Diseases, Hangzhou, 310003 China

**Keywords:** Cancer

## Abstract

Pancreatic cancer (PC) continues to demand urgent therapeutic innovation given its limited treatment options. Here, through phenotypic screening of a natural product library followed by systematic validation, we identified chrysosplenetin (CHR) as a bioactive compound with anti-PC activity. Transcriptomic profiling and functional analyses demonstrated that CHR induced endoplasmic reticulum (ER) stress, thereby activating the unfolded protein response (UPR) and subsequent apoptosis, while paradoxically triggering a protective autophagy. Genetic or pharmacological inhibition of autophagy potentiated CHR-induced antitumor efficacy. Using an integrated approach including proteomic analysis, bio-layer interferometry, cellular thermal shift assay, and molecular docking, we confirmed TMED3 as a direct target of CHR. Functional studies revealed that disruption of TMED3 expression partially restored ER homeostasis, attenuating CHR-induced UPR activation and apoptosis. Furthermore, CHR combined with standard chemotherapy or autophagy inhibitors exhibited enhanced antitumor activity in preclinical models, providing a basis for future therapeutic exploration of the TMED3–ER stress axis. Together, our findings establish TMED3 as a novel therapeutic target in PC, revealing that disrupting ER proteostasis via TMED3 perturbation represents a potential therapeutic strategy warranting further investigation.

## Synopsis

Chrysosplenetin (CHR) induces TMED3 aggregation, causing lethal ER stress in pancreatic cancer and identifying TMED3 as a direct target and druggable vulnerability. CHR combined with gemcitabine or autophagy inhibitors enhances antitumor activity in preclinical models.

- Phenotypic screening identifies CHR as a bioactive compound with anti-pancreatic cancer activity.
- CHR induces ER stress-mediated apoptosis but also triggers protective autophagy, which limits its antitumor activity.
- TMED3 is validated as a direct target of CHR, linking its disruption to UPR activation and apoptosis.
- Combining CHR with gemcitabine or autophagy inhibitors enhances antitumor activity in PDX and PDO models.

## Abstract (web summary)

Chrysosplenetin (CHR) induces TMED3 aggregation, causing lethal ER stress in pancreatic cancer and identifying TMED3 as a direct target and druggable vulnerability. CHR combined with gemcitabine or autophagy inhibitors enhances antitumor activity in preclinical models.

## Graphical abstract

**Figure:**
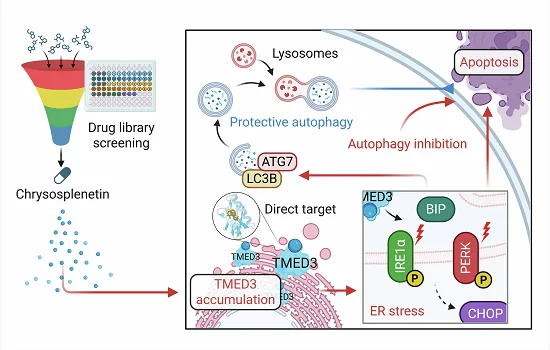

The paper explained **Problem**

Pancreatic cancer (PC) remains one of the most lethal malignancies, with current first-line chemotherapeutic regimens yielding only modest survival improvements. This unmet clinical need urgently requires the discovery of novel, actionable therapeutic targets and mechanism-based intervention strategies. Natural products represent a valuable reservoir for anticancer compound discovery; however, their precise molecular targets and underlying mechanisms often remain poorly defined, hindering rational translational application.

**Results**

Through phenotypic screening of a natural product library, we identified chrysosplenetin (CHR) as a bioactive compound with anti-PC activity. Using an integrated target identification strategy combining chemical proteomics, biophysical assays, and computational modeling, we validated TMED3 as a direct target of CHR. CHR-induced TMED3 aggregation disrupts endoplasmic reticulum (ER) homeostasis and triggers sustained pro-death unfolded protein response (UPR) signaling, thereby suppressing PC progression. Meanwhile, CHR treatment concurrently activates protective autophagy, which partially attenuates its antitumor efficacy. Genetic or pharmacological autophagy inhibition significantly potentiates CHR-mediated cancer cell death. Consistent with these mechanistic findings, combinatorial regimens of CHR with gemcitabine or autophagy inhibitors exhibit superior antitumor effects in patient-derived organoid and xenograft models.

**Impact**

Our study identifies TMED3 as a novel druggable vulnerability in PC and reveals a previously unrecognized regulatory axis linking TMED3 perturbation to lethal ER stress activation. These findings establish the TMED3–ER stress axis as a potential therapeutic vulnerability, providing a mechanistic foundation for future exploration of TMED3-targeted interventions in this intractable malignancy.

## Introduction

Pancreatic cancer (PC) represents one of the most lethal malignancies, characterized by an exceptionally poor prognosis (Kane et al, 2026). The disease’s insidious onset and frequent absence of early-stage symptoms typically result in delayed diagnosis until advanced stages, when local invasion or distant metastases have already occurred (Stoop et al, 2025). Consequently, the majority of PC patients are ineligible for radical surgical resection, rendering systemic therapy the mainstay of treatment (Stoop et al, 2024). However, first-line chemotherapeutic regimens, primarily based on gemcitabine or 5-fluorouracil, provide only a negligible survival benefit, prolonging life by only a few months in the case of palliative care (Conroy et al, 2011). In addition, the inherent toxicity of chemotherapy significantly reduces the patient’s quality of life (Conroy et al, 2018). Although significant advances in novel therapies, including immune checkpoint therapy and cellular therapy, have revolutionized treatment paradigms for various malignancies in recent years, PC management remains a formidable clinical challenge (Beatty et al, 2018; Padrón et al, 2022; Reiss et al, 2022; Salgado-Pascual et al, 2026). This grim reality highlights the urgent need for safe and effective innovative antitumor therapies, especially mechanism-driven therapies targeting novel vulnerabilities in PC.

Natural products have emerged as a primary source of novel anticancer agents, owing to their unique structural diversity, biocompatibility, and evolutionarily refined bioactivity (Newman and Cragg, 2020; Parthasarathy et al, 2020). For example, elliptinium, a plant alkaloid, has been developed into the clinically used anticancer drug Celiptium for the treatment of breast cancer, while paclitaxel, extracted from the bark of the Pacific yew tree, has become a first-line chemotherapeutic agent for a variety of cancers (Rouëssé et al, 1993; Seidman et al, 1998; Tropé et al, 1997). Notably, over 50% of the Food and Drug Administration (FDA)-approved drugs from 1939–2016 originated from natural compounds, with promising candidates like resveratrol, curcumin, and celastrol currently in pharmaceutical development pipelines (Patel et al, 2010; Rodrigues et al, 2016; Weng and Goel, 2022; Xu et al, 2023). However, the development of natural product-based medicines faces substantial challenges, primarily due to the complexity of their polypharmacological mechanisms. Most natural products interact with multiple protein targets rather than a single target, complicating target identification and mechanistic elucidation (Kabir and Muth, 2022). This knowledge gap has historically hindered their clinical development, underscoring the importance of robust target deconvolution strategies. Recent advancements in chemical proteomics, encompassing both activity-based protein profiling and compound-centric chemical proteomics, along with nonlabeling approaches such as cellular thermal shift assays (CETSA) and drug affinity responsive target stability (DARTS), now enable comprehensive target mapping while preserving native compound activity (Chen et al, 2020). Despite remaining challenges in sensitivity and optimization, these methodologies are accelerating the development of mechanism-driven therapies (Cohen et al, 2008). This progress is particularly impactful for malignancies with dire therapeutic shortages, including PC. In this context, natural product-derived agents may offer breakthrough solutions to overcome the limitations of conventional chemotherapy and address the critical unmet clinical needs.

Chrysosplenetin (CHR), a bioactive polymethoxylated flavonoid derived from Asteraceae species, including *Artemisia annua*, exhibits a range of biological activities (Tang et al, 2000). A previous study demonstrated its ability to inhibit estrogen deficiency-induced osteoporosis by promoting osteoblast differentiation via the Wnt/β-catenin pathway in animal models (Hong et al, 2019). Moreover, CHR exhibited significant cytotoxicity in a variety of breast cancer cell lines, likely through activation of the spindle checkpoint or inhibition of topoisomerase I/II, thereby inducing apoptosis (Sinha et al, 2015; Şöhretoğlu et al, 2020). However, the potential effects and direct targets of CHR across a wider range of cancer-related settings, particularly in pancreatic cancer, remains underexplored.

The endoplasmic reticulum (ER) serves as the primary site for biosynthesis, folding, and post-translational modification of secretory and transmembrane proteins (Bakambamba et al, 2026). While tightly regulated, this process remains vulnerable to disruption by both extracellular stimuli and intracellular perturbations, leading to ER stress characterized by accumulation of misfolded proteins (Hetz, 2012). Cancer cells frequently experience chronic ER stress due to their characteristic genomic instability, transcriptional dysregulation, and metabolic reprogramming, which collectively impair proteostatic balance and influence malignant progression (Chen and Cubillos-Ruiz, 2021; Wei et al, 2026). Mammalian cells employ a sophisticated surveillance system comprising three ER-resident stress sensors: inositol-requiring enzyme 1α (IRE1α), PRKR-like ER kinase (PERK), and activating transcription factor 6 (ATF6) (Sicari et al, 2020). IRE1α-X-box binding protein 1 (XBP1) is the most evolutionarily conserved unfolded protein response (UPR) branch (Cox et al, 1993). Activated IRE1α oligomerizes and autophosphorylates, activating its RNase domain to splice XBP1 mRNA. The spliced mRNA encodes the functional transcription factor XBP1s (Yoshida et al, 2001). IRE1α also degrades certain mRNAs via regulated IRE1-dependent decay (RIDD), reducing ER protein load (Hollien and Weissman, 2006). Through adapter interactions, IRE1α further activates JNK, p38 and NF-κB pathways, modulating inflammation, autophagy and apoptosis (Zhou et al, 2025). PERK, a serine/threonine kinase, primarily phosphorylates eukaryotic initiation factor-2α (eIF2α) (Harding et al, 1999). This attenuates global translation while promoting ATF4 activation. ATF4 subsequently upregulates the expression of the transcription factor C/EBP‑homologous protein (CHOP) (Liu et al, 2015). ATF6 exerts its function following translocation to the Golgi apparatus, where S1P/S2P-mediated cleavage liberates its transcriptionally active N-terminal fragment (Haze et al, 1999). Under physiological conditions, these sensors remain inactive through association with the molecular chaperone binding-immunoglobulin protein (BIP; also known as GRP78). Stress conditions trigger BIP sequestration by misfolded proteins, liberating and activating these sensors to initiate the UPR (Walter and Ron, 2011). This multifaceted adaptive program restores proteostasis through: (1) transcriptional regulation of chaperones and folding enzymes, (2) selective mRNA decay, (3) transient translational suppression, (4) ER-associated protein degradation (ERAD)-mediated protein clearance, and (5) autophagy activation (Christianson et al, 2023; Kaufman, 1999; Yorimitsu et al, 2006). The UPR demonstrates remarkable functional plasticity. While moderate activation promotes cellular adaptation and survival, sustained or severe ER stress commits cells to apoptosis (Wang et al, 2018). Accordingly, understanding the detailed mechanisms linking cancer therapy to ER stress responses is crucial, as it governs clinical outcomes implicated in either response or resistance to treatment.

In this study, we elucidated the mechanisms underlying CHR-induced cell death and identified TMED3 as a direct target of CHR in PC. Notably, CHR combined with gemcitabine or the autophagy inhibitor chloroquine (CQ) resulted in significantly enhanced antitumor activity in patient-derived xenografts (PDX) and organoids (PDOs), providing preclinical evidence supporting further exploration of the TMED3–ER stress axis as a therapeutic strategy. Our findings establish TMED3 as a novel therapeutic target in PC and reveal that disrupting ER proteostasis via TMED3 perturbation represents a potential strategy for PC treatment.

## Results

### Discovery and validation of CHR as a bioactive compound with anti-PC activity

**Figure 1:**
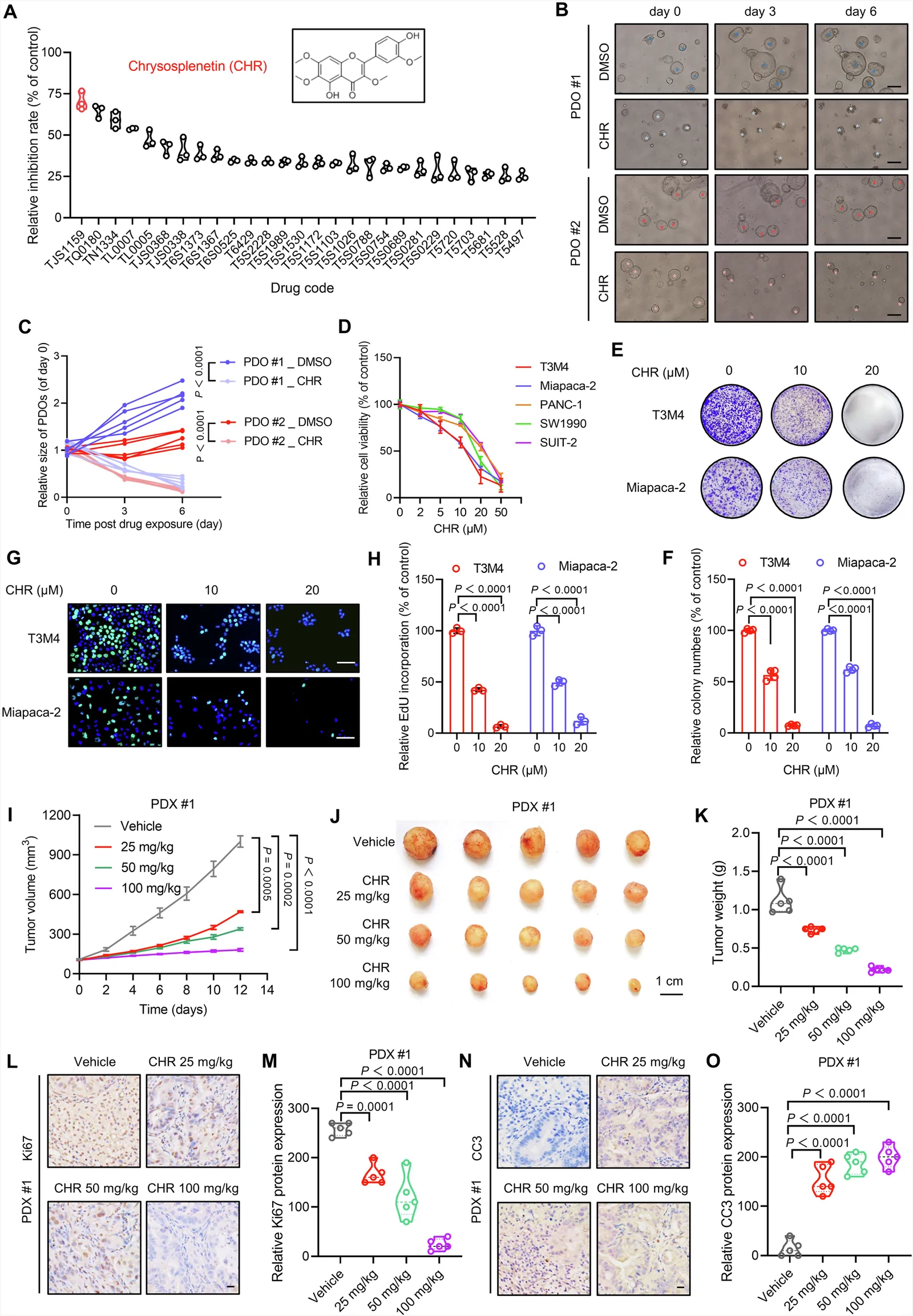
Discovery and validation of CHR as a bioactive compound with anti-PC activity. (**A**) 27 natural compounds with inhibition rates exceeding 25% on Miapaca-2 cells from high-throughput screening. *N* = 3 independent biological replicates. (**B**, **C**) Representative images (**B**) and quantification of relative sizes of PDO #1 and PDO #2 (**C**) after DMSO or CHR (20 μM) treatment over time, scale bar: 75 μm. * denoted the PDO of interest, with matching color in (**C**). Two-way ANOVA was used to analyze statistical differences in (**C**). *N* = 5 independent organoids. (**D**) Cell viability of human PC cell lines treated with various concentrations of CHR for 48 h. Mean with ± SD. *N* = 3 independent biological replicates. (**E**, **F**) Representative images (**E**) and quantification of colony numbers (**F**) of T3M4 and Miapaca-2 cells treated with DMSO or CHR. One-way ANOVA was used to analyze statistical differences in (**F**). Mean with ± SD. *N* = 4 independent biological replicates. (**G**, **H**) Representative images (**G**) and quantification of EdU incorporation rate (**H**) of T3M4 and Miapaca-2 cells treated with CHR for 48 h, scale bar: 50 μm. One-way ANOVA was used to analyze statistical differences in (**H**). Mean with ± SD. *N* = 3 independent biological replicates. (**I**–**K**) Tumor volumes (**I**), images (**J**) and weights (**K**) of PDX #1 xenografts treated with vehicle or CHR (25 mg/kg, 50 mg/kg, or 100 mg/kg) for 12 days. Two-way ANOVA was used to analyze statistical differences in (**I**). Mean with ± SEM. One-way ANOVA was used to analyze statistical differences in (**K**). *N* = 5 independent mice. (**L**–**O**) Representative images (**L**, **N**) and quantification (**M**, **O**) of immunohistochemical staining for Ki67 and CC3 of PDX #1 xenografts treated with vehicle or CHR, scale bar: 20 μm. One-way ANOVA was used to analyze statistical differences in (**M**, **O**). *N* = 5 independent mice. Source data are available online for this figure.

In our systematic effort to identify novel natural compounds with potent anti-PC activity, we developed a high-throughput screening platform utilizing Miapaca-2 cells, with cell viability quantified at a standardized concentration of 20 μM (Appendix Fig. S1A and Dataset EV1). From the initial screening of 411 compounds, 27 exhibited significant anti-proliferative effects (> 25% inhibition). Among them, CHR exhibited the most pronounced antitumor activity (Fig. 1A).

To validate these screening results, we established PDOs from two PC patients, observing a progressive and substantial reduction in PDO size over a 6-day treatment period with CHR (Fig. 1B,C). Given the absence of prior reports on CHR’s efficacy against PC, we conducted comprehensive evaluations of its anti-proliferative effects across multiple human PC cell lines (T3M4, Miapaca-2, PANC-1, SW1990, and SUIT-2) using CCK-8 viability assays. All tested lines showed dose-dependent sensitivity to CHR treatment (Fig. 1D). Further characterization revealed CHR’s potent suppression of PC cell proliferation through both colony formation assays (Fig. 1E,F) and EdU incorporation experiments (Fig. 1G,H), collectively demonstrating its robust anti-cancer activity in vitro.

To evaluate the therapeutic potential of CHR in vivo, we established two distinct subcutaneous xenograft models using T3M4 and Miapaca-2 cells in nude mice. As shown in Appendix Fig. S1B–D and G–I, CHR treatment resulted in marked inhibition of tumor growth, with significant reductions in tumor volume and weight compared to vehicle controls. Immunohistochemical analysis revealed substantially weaker Ki67 staining in CHR-treated tumors (Appendix Fig. S1E,F and J,K), confirming the anti-proliferative effects observed in vitro. To enhance clinical relevance and further characterize the dose‑response relationship, we conducted an additional PDX experiment evaluating CHR at 0, 25, 50, and 100 mg/kg. The results demonstrate a clear dose‑dependent suppression of tumor growth, with higher doses achieving more pronounced inhibition (Fig. 1I–K). Consistent with the enhanced antitumor efficacy, Ki67 positivity was progressively reduced with increasing CHR doses (Fig. 1L,M). Notably, even at the highest dose tested (100 mg/kg), we observed no significant toxicity based on comprehensive assessments including body weight monitoring, serum biochemistry profiles, and histopathological examination (H&E staining) of major organs (Appendix Fig. S2A–H). These consistent results across different in vivo models demonstrate that CHR exhibits robust anti-PC activity with a tolerable safety profile at the doses tested.

Previous studies have indicated that CHR can induce apoptosis in breast cancer cells (Sinha et al, 2015; Şöhretoğlu et al, 2020), prompting us to investigate whether CHR similarly exerts its anti-proliferative effects on PC cells through apoptosis induction. As expected, our flow cytometric analysis confirmed that CHR treatment significantly induced apoptosis in T3M4 and Miapaca-2 cells (Appendix Fig. S3A,B). Western blot analysis revealed increased levels of cleaved caspase3 (CC3) and cleaved PARP, key markers of apoptotic cell death (Appendix Fig. S3C). Moreover, the pan-caspase inhibitor Z-VAD substantially rescued CHR-mediated growth inhibition (Appendix Fig. S3D–F). In vivo validation also showed enhanced CC3 staining in CHR-treated xenografts (Fig. 1N,O; Appendix Fig. S3G–J), confirming apoptosis induction as a consistent feature of CHR’s anti-tumor activity. These findings collectively demonstrate that CHR exerts its anti-PC effects, at least in part, through induction of apoptotic cell death.

### CHR induces apoptosis by activating ER stress-mediated UPR in PC cells

**Figure 2:**
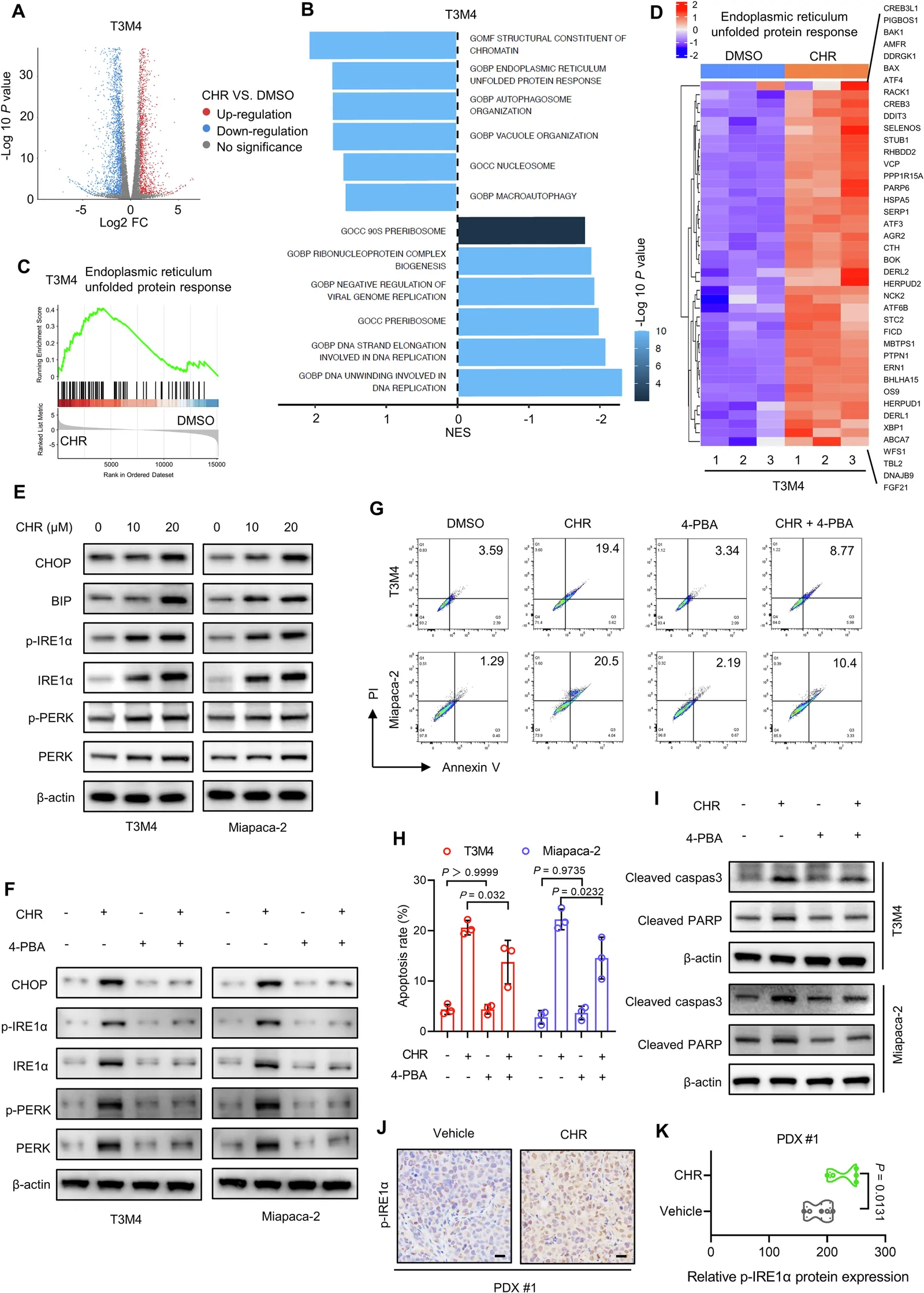
CHR induces apoptosis by activating ER stress-mediated UPR in PC cells. (**A**) Volcano plot of differentially expressed genes in T3M4 cells treated with CHR (20 μM) versus DMSO. Upregulated (red) and downregulated (blue) genes are highlighted. Wald test was used to analyze statistical differences. The Benjamini–Hochberg procedure was used to adjust. *N* = 3 independent biological replicates. (**B**) Pathway enrichment analysis of upregulated and downregulated pathways in T3M4 cells treated with CHR. Permutation test was used to analyze statistical differences. The Benjamini–Hochberg procedure was used to adjust. (**C**) Gene Set Enrichment Analysis of the ER UPR pathway in T3M4 cells treated with CHR versus DMSO. (**D**) Heatmap of ER UPR-associated gene expression in T3M4 cells treated with CHR versus DMSO. (**E**) Immunoblotting analysis of UPR markers in T3M4 and Miapaca-2 cells treated with indicated concentrations of CHR for 48 h. (**F**) Immunoblotting analysis of UPR markers in T3M4 and Miapaca-2 cells treated with CHR (10 μM) in the presence or absence of 4-PBA (2 mM) for 48 h. (**G**, **H**) Flow cytometric analysis (**G**) and quantification (**H**) of apoptosis rate in T3M4 and Miapaca-2 cells treated with CHR in the presence or absence of 4-PBA for 48 h. One-way ANOVA was used to analyze statistical differences in (**H**). Mean with ± SD. *N* = 3 independent biological replicates. (**I**) Immunoblotting analysis of CC3 and cleaved PARP in T3M4 and Miapaca-2 cells treated with CHR in the presence or absence of 4-PBA for 48 h. (**J**, **K**) Representative images (**J**) and quantification (**K**) of immunohistochemical staining for p-IRE1α of PDX #1 xenografts treated with vehicle or 25 mg/kg CHR, scale bar: 20 μm. Student’s t test was used to analyze statistical differences in (**K**). *N* = 5 independent mice. Source data are available online for this figure.

**Figure EV1:**
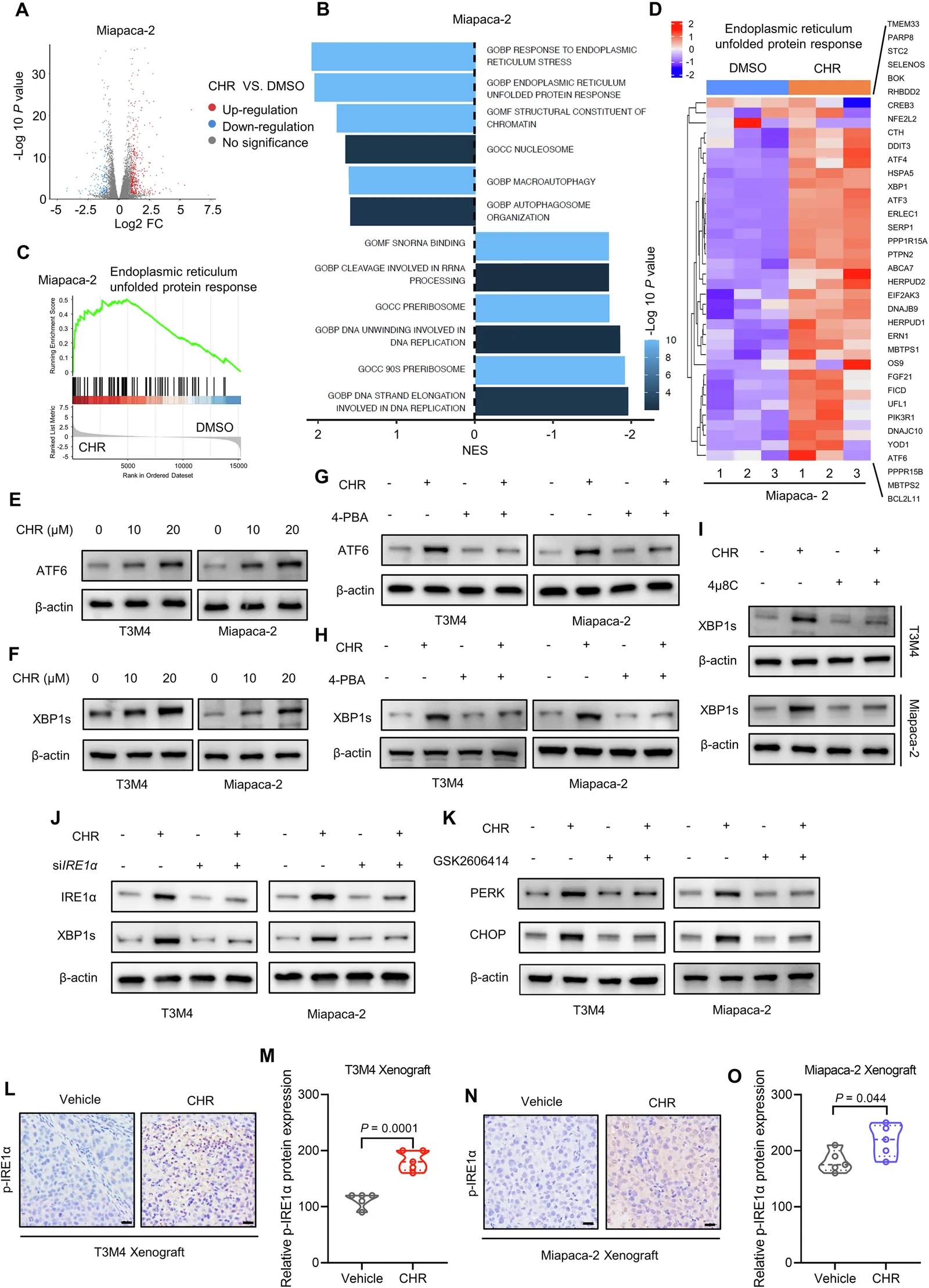
CHR triggers ER stress in PC cells. (**A**) Volcano plot of differentially expressed genes in Miapaca-2 cells treated with CHR (20 μM) versus DMSO. Upregulated (red) and downregulated (blue) genes are highlighted. Wald test was used to analyze statistical differences. The Benjamini–Hochberg procedure was used to adjust. *N* = 3 independent biological replicates. (**B**) Pathway enrichment analysis of upregulated and downregulated pathways in Miapaca-2 cells treated with CHR. Permutation test was used to analyze statistical differences. The Benjamini–Hochberg procedure was used to adjust. (**C**) Gene Set Enrichment Analysis of the ER UPR pathway in Miapaca-2 cells treated with CHR versus DMSO. (**D**) Heatmap of ER UPR-associated gene expression in Miapaca-2 cells treated with CHR versus DMSO. (**E**, **F**) Immunoblotting analysis of ATF6 (**E**) and XBP1s (**F**) in T3M4 and Miapaca-2 cells treated with indicated concentrations of CHR for 48 h. (**G**, **H**) Immunoblotting analysis of ATF6 (**G**) and XBP1s (**H**) in T3M4 and Miapaca-2 cells treated with CHR (10 μM) in the presence or absence of 4-PBA (2 mM) for 48 h. (**I**) Immunoblotting analysis of XBP1s in T3M4 and Miapaca-2 cells treated with CHR in the presence or absence of 4μ8C (5 μM) for 48 h. (**J**) Immunoblotting analysis of IRE1α and XBP1s in T3M4 and Miapaca-2 cells treated with CHR for 48 h after transfected with si*IRE1α* or si*Scramble*. (**K**) Immunoblotting analysis of PERK and CHOP in T3M4 and Miapaca-2 cells treated with CHR in the presence or absence of GSK2606414 (5 μM) for 48 h. (**L**–**O**) Representative images (**L**, **N**) and quantification (**M**, **O**) of immunohistochemical staining for p-IRE1α of T3M4 and Miapaca-2 xenografts treated with vehicle or CHR, scale bar: 20 μm. Student’s t test was used to analyze statistical differences in (**M**, **O**). *N* = 5 independent mice. Source data are available online for this figure.

To investigate the molecular basis of CHR-induced apoptosis, we performed RNA sequencing (RNA-seq) on T3M4 and Miapaca-2 cells treated with 20 μM CHR. Transcriptomic analysis identified significant differential gene expression (|fold change| ≥  2, *P*(adj) ≤ 0.05) compared to untreated controls (Figs. 2A and EV1A). Subsequent gene set enrichment analysis (GSEA) revealed substantial enrichment of pathways associated with apoptosis, such as the ER UPR, DNA replication, and autophagy (Figs. 2B and EV1B). Notably, the ER UPR pathway activation was observed in both cell lines following CHR exposure (Fig. 2C,D and EV1C,D).

To validate whether ER stress-mediated UPR was activated by CHR in PC cells, we examined the protein expression of classic UPR markers, including PERK, p-PERK, IRE1α, p-IRE1α, ATF6, BIP, XBP1s, and CHOP. Immunoblotting results showed increased expression of these UPR markers in CHR-treated PC cells (Figs. 2E and EV1E,F). Co-treatment with the ER stress inhibitor 4-PBA significantly reduced CHR-mediated elevation of UPR markers (Figs. 2F and EV1G,H). Furthermore, pharmacological inhibition of IRE1α with 4μ8C, as well as siRNA‑mediated knockdown of *IRE1α*, significantly attenuated CHR‑induced upregulation of the downstream splicing variant XBP1s (Fig. EV1I,J). Similarly, inhibition of PERK with GSK2606414 blunted CHR‑induced CHOP expression (Fig. EV1K). These data collectively demonstrate that CHR activates multiple branches of the UPR in PC cells. To clarify the relationship between CHR-induced UPR and apoptosis, we investigated whether 4-PBA could reverse the apoptosis and cell-killing effects induced by CHR. Flow cytometric analysis displayed that 4-PBA markedly compromised CHR-triggered apoptosis in PC cells (Fig. 2G,H). Downregulation of CC3 and cleaved PARP protein expression was also observed in CHR-treated PC cells in combination with 4-PBA (Fig. 2I). In addition, cell viability assays revealed that 4-PBA rescued CHR-induced cytotoxicity (Appendix Fig. S4A). Consistent with this finding, 4-PBA restored the colony formation capacity in CHR-treated PC cells (Appendix Fig. S4B,C). We also confirmed UPR activation in vivo through the immunohistochemical detection of p-IRE1α expression. As shown in Figs. 2J,K and EV1L–O, the CHR-treated PDX model, T3M4 and Miapaca-2 xenografts exhibited stronger p-IRE1α staining than the controls. Taken together, these finding indicate that CHR-induced apoptosis is attributed to ER stress-mediated UPR in PC cells.

### CHR initiates autophagy to respond to ER stress in PC cells

In response to ER stress, cancer cells often activate autophagy, including selective ER-phagy, to degrade fragmented ER components and misfolded proteins, thereby restoring ER homeostasis (Miller and Thorburn, 2021). Interestingly, autophagy-related biological processes, such as GOBP MACROAUTOPHAGY and GOBP AUTOPHAGOSOME ORGANIZATION, were concomitantly enriched in T3M4 and Miapaca-2 cells after CHR treatment (Fig. 2B, EV1B and 3A; Appendix Fig. S5A,B). These findings prompted us to investigate whether CHR triggers autophagy in PC cells.

**Figure 3:**
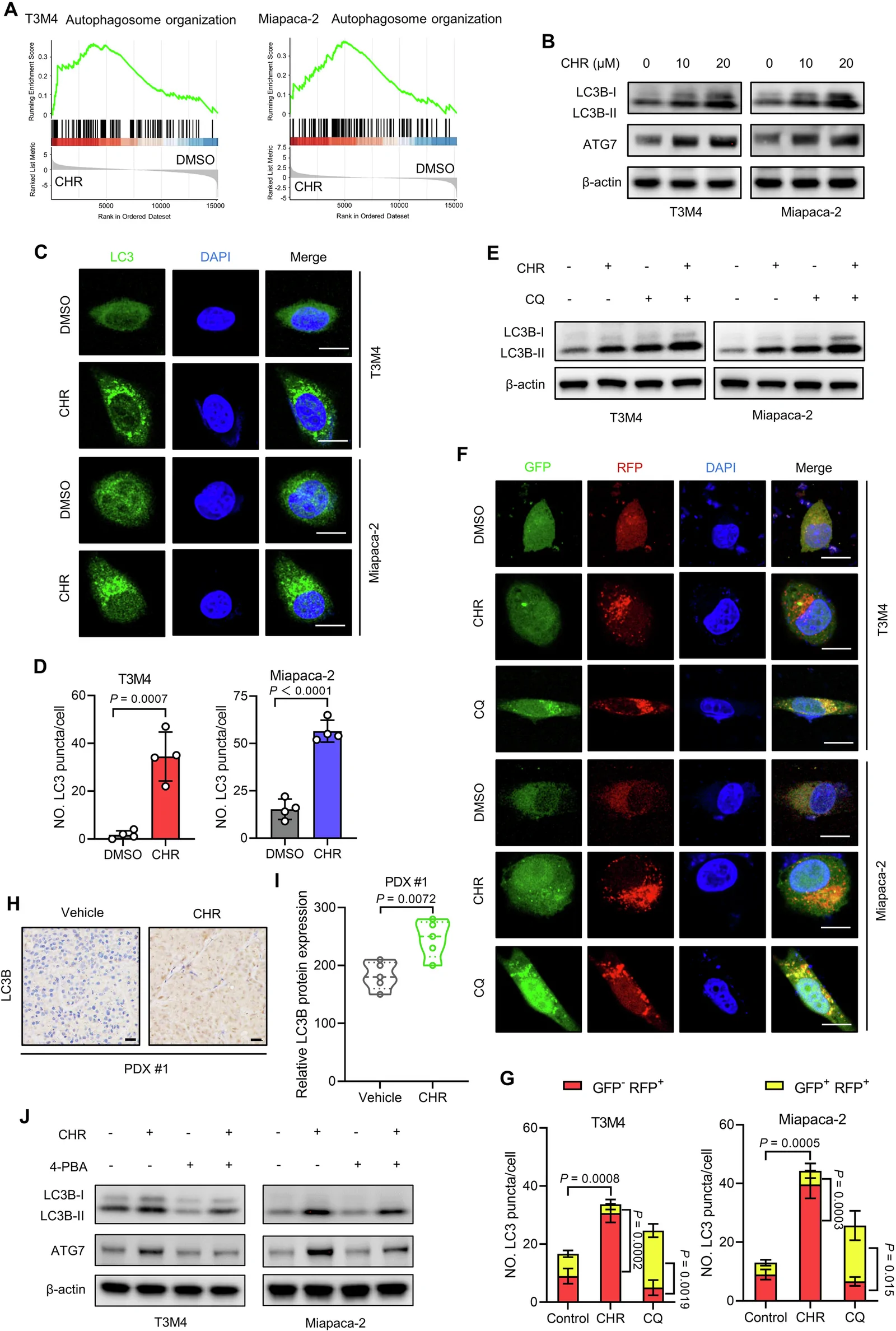
CHR initiates autophagy to respond to ER stress in PC cells. (**A**) Gene Set Enrichment Analysis of the autophagosome organization gene sets in T3M4 and Miapaca-2 cells treated with CHR (20 μM) versus DMSO. (**B**) Immunoblotting analysis of autophagy markers in T3M4 and Miapaca-2 cells treated with indicated concentrations of CHR. (**C**,** D**) Representative images (**C**) and quantification of LC3 puncta (**D**) of immunofluorescent staining for LC3 (green) and nuclei (DAPI, blue) in T3M4 and Miapaca-2 cells treated with DMSO or CHR (10 μM) for 48 h, scale bar: 10 μm. Student’s t test was used to analyze statistical differences in (**D**). Mean with ± SD. *N* = 4 independent cells. (**E**) Immunoblotting analysis of LC3B I/II in T3M4 and Miapaca-2 cells treated with CHR in the presence or absence of CQ (10 μM) for 48 h. (**F**,** G**) Representative images (**F**) and quantification of GFP⁺ RFP⁺ (autophagosomes) and GFP⁻ RFP⁺ (autolysosomes) puncta (**G**) of T3M4 and Miapaca-2 cells transiently transfected with mRFP-GFP-LC3 and treated with DMSO, CHR or CQ for 48 h, scale bar: 10 μm. Student’s t test was used to analyze statistical differences in (**G**). Mean with ± SD. *N* = 3 independent cells. (**H**, **I**) Representative images (**H**) and quantification (**I**) of immunohistochemical staining for LC3B of PDX #1 xenografts treated with vehicle or 25 mg/kg CHR, scale bar: 20 μm. Student’s t test was used to analyze statistical differences in (**I**). *N* = 5 independent mice. (**J**) Immunoblotting analysis of autophagy markers in T3M4 and Miapaca-2 cells treated with CHR in the presence or absence of 4-PBA for 48 h. Source data are available online for this figure.

As expected, CHR treatment induced a dose-dependent increase in the lipidation of LC3B-I to LC3B-II and elevated ATG7 expression, two well-established markers of autophagy activation, in both T3M4 and Miapaca-2 cells (Fig. 3B). Furthermore, immunofluorescence microscopy confirmed a pronounced accumulation of LC3B puncta in CHR-treated cells (Fig. 3C,D). To distinguish between enhanced autophagy initiation and impaired autophagic flux, we assessed LC3B-II levels in the presence of CQ, an autolysosome inhibitor. Co-treatment with CQ further augmented LC3B-II accumulation (Fig. 3E), suggesting active autophagic flux rather than blockade. This was further corroborated using tandem mRFP-GFP-LC3 reporters, where CHR-treated cells exhibited a higher proportion of autolysosomes (GFP^−^ RFP^+^) compared to autophagosomes (GFP^+^ RFP^+^), whereas CQ treatment, as expected, led to autophagosome accumulation (Fig. 3F,G). Additionally, immunohistochemical analysis of tumor xenografts revealed significantly stronger LC3B staining in CHR-treated tissues compared to controls (Fig. 3H,I; Appendix Fig. S5C,D), further supporting autophagy induction in vivo.

To determine whether CHR-induced autophagy was an adaptive response to ER stress, we first performed a detailed time-course analysis using an effective concentration of CHR (20 µM) (Appendix Fig. S5E). The data clearly showed the activation kinetics of key pathways over 48 h. Firstly, ATF6 and phosphorylation of PERK and IRE1α were detectable as early as 2–4 h post-CHR treatment, preceding the upregulation of downstream effectors like CHOP and XBP1s (evident by 8–12 h). For autophagy induction, the conversion of LC3B-I to LC3B-II and upregulation of ATG7 began by 8–12 h, following the initial phosphorylation of IRE1α. Cleavage of caspase-3 and PARP became prominent at 24–48 h, consistent with apoptosis being a later-stage outcome of sustained ER stress. This timeline supports a model in which autophagy is a secondary, adaptive response triggered by CHR-induced ER stress.

To further validate this sequence, we evaluated autophagy markers in cells co-treated with 4-PBA. As shown in Fig. 3J, 4-PBA substantially attenuated CHR-mediated upregulation of autophagy-related proteins. The functional linkage between UPR and autophagy was further delineated. Specific inhibition of IRE1α (using 4μ8C or si*IRE1α*) significantly suppressed CHR-induced LC3B lipidation and ATG7 expression, placing autophagic activation downstream of this UPR branch, whereas inhibition of the PERK branch with GSK2606414 did not produce a comparable effect (Appendix Fig. S6A–C). Conversely, pharmacological inhibition of autophagy using either wortmannin (Wort, early‑stage) or bafilomycin A1 (Baf-A1, late-stage), as well as genetic knockdown of *ATG7*, did not significantly alter UPR marker expression (Appendix Fig. S6D–F), indicating that autophagy acts downstream of ER stress in this context. Taken together, these data demonstrate that CHR triggers functional autophagic flux in PC cells to respond to ER stress.

### Autophagy promotes cell survival in CHR-treated PC cells

**Figure 4:**
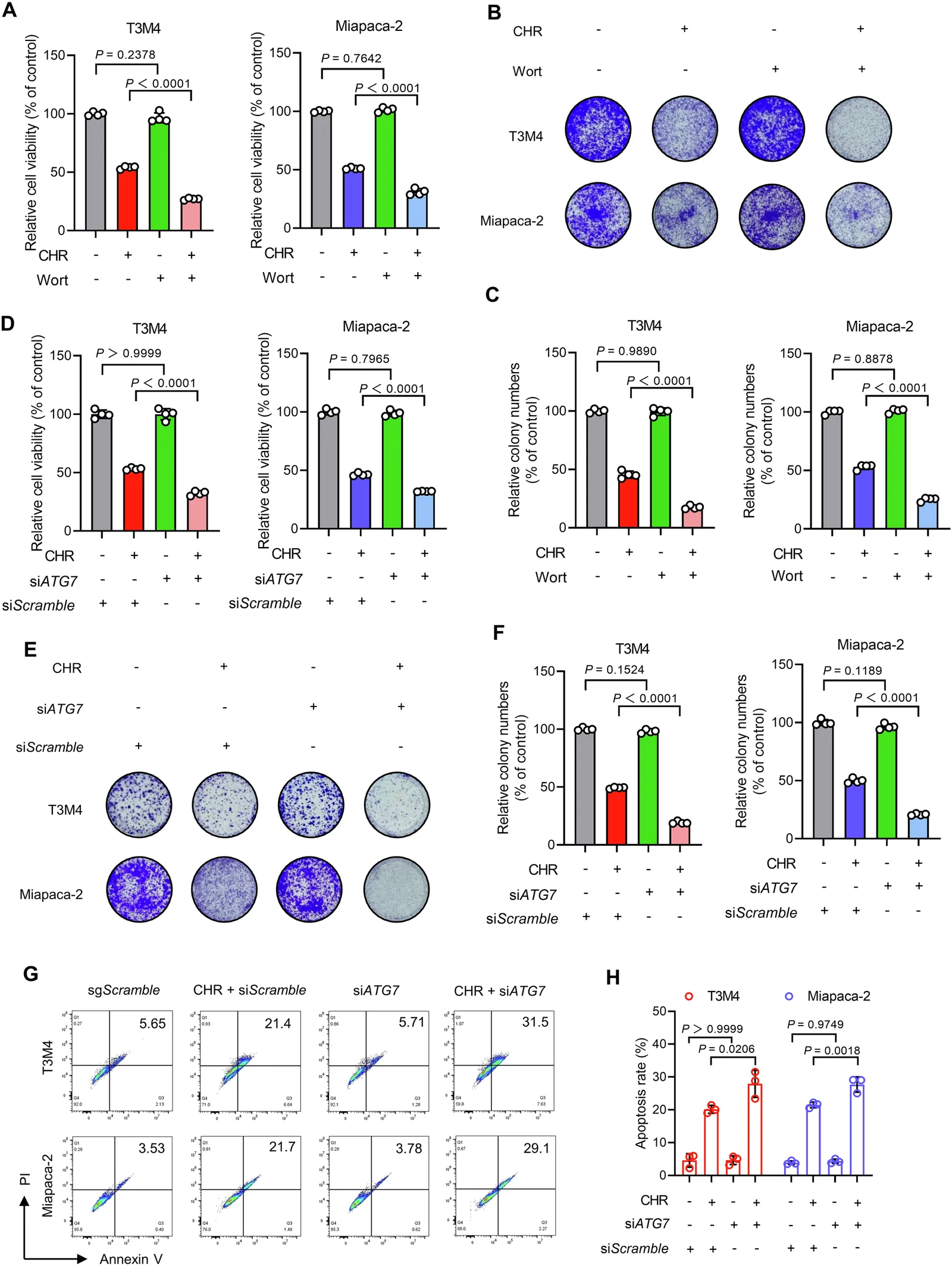
Autophagy promotes cell survival in CHR-treated PC cells. (**A**) Cell viability of T3M4 and Miapaca-2 cells treated with CHR (10 μM) in the presence or absence of Wort (1 μM) for 48 h. One-way ANOVA was used to analyze statistical differences. Mean with ± SD. *N* = 4 independent biological replicates. (**B**, **C**) Representative images (**B**) and quantification of colony numbers (**C**) of T3M4 and Miapaca-2 cells treated in the presence or absence of Wort. One-way ANOVA was used to analyze statistical differences in (**C**). Mean with ± SD. *N* = 4 independent biological replicates. (**D**) Cell viability of T3M4 and Miapaca-2 cells treated with CHR for 48 h after transfected with si*ATG7* or si*Scramble*. One-way ANOVA was used to analyze statistical differences. Mean with ± SD. *N* = 4 independent biological replicates. (**E**, **F**) Representative images (**E**) and quantification of colony numbers (**F**) of T3M4 and Miapaca-2 cells treated with CHR after transfected with si*ATG7* or si*Scramble*. One-way ANOVA was used to analyze statistical differences in (**F**). Mean with ± SD. *N* = 4 independent biological replicates. (**G**, **H**) Flow cytometric analysis (**G**) and quantification (**H**) of apoptosis rate in T3M4 and Miapaca-2 cells treated with CHR for 48 h after transfected with si*ATG7* or si*Scramble*. One-way ANOVA was used to analyze statistical differences in (**H**). Mean with ± SD. *N* = 3 independent biological replicates. Source data are available online for this figure.

While CHR-induced UPR simultaneously activates apoptosis and autophagy in PC cells, the ultimate cell fate, survival or death, depends on the stress intensity and duration (Zhang et al, 2019). Paradoxically, although autophagy is initially a pro-survival response to ER stress, sustained activation can also contribute to cell death (Muñoz-Guardiola et al, 2021). To delineate the role of autophagy in CHR’s anticancer effects, we pharmacologically inhibited autophagy using Wort, CQ, or Baf-A1 in CHR-treated PC cells. All three inhibitors significantly reduced cell viability (Fig. 4A; Appendix Fig. S7A,D) and clonogenic survival (Fig. 4B,C; Appendix Fig. S7B,C, E,F). Similarly, *ATG7* knockdown enhanced CHR-mediated growth suppression (Fig. 4D–F), indicating that autophagy supports cell survival under CHR-induced ER stress.

Notably, autophagy inhibition, whether pharmacologically (via Wort, CQ or Baf-A1) or genetically (via si*ATG7*), exacerbated apoptosis in CHR-treated cells, as evidenced by increased apoptotic populations (Fig. 4G,H; Appendix Fig. S8A–F) and elevated levels of CC3 and cleaved PARP (Appendix Fig. S8G,H). These findings collectively demonstrate that autophagy acts as a cytoprotective mechanism in CHR-treated PC cells, and its suppression enhances CHR-induced cell death.

### CHR directly binds to TMED3 in PC cells

**Figure 5:**
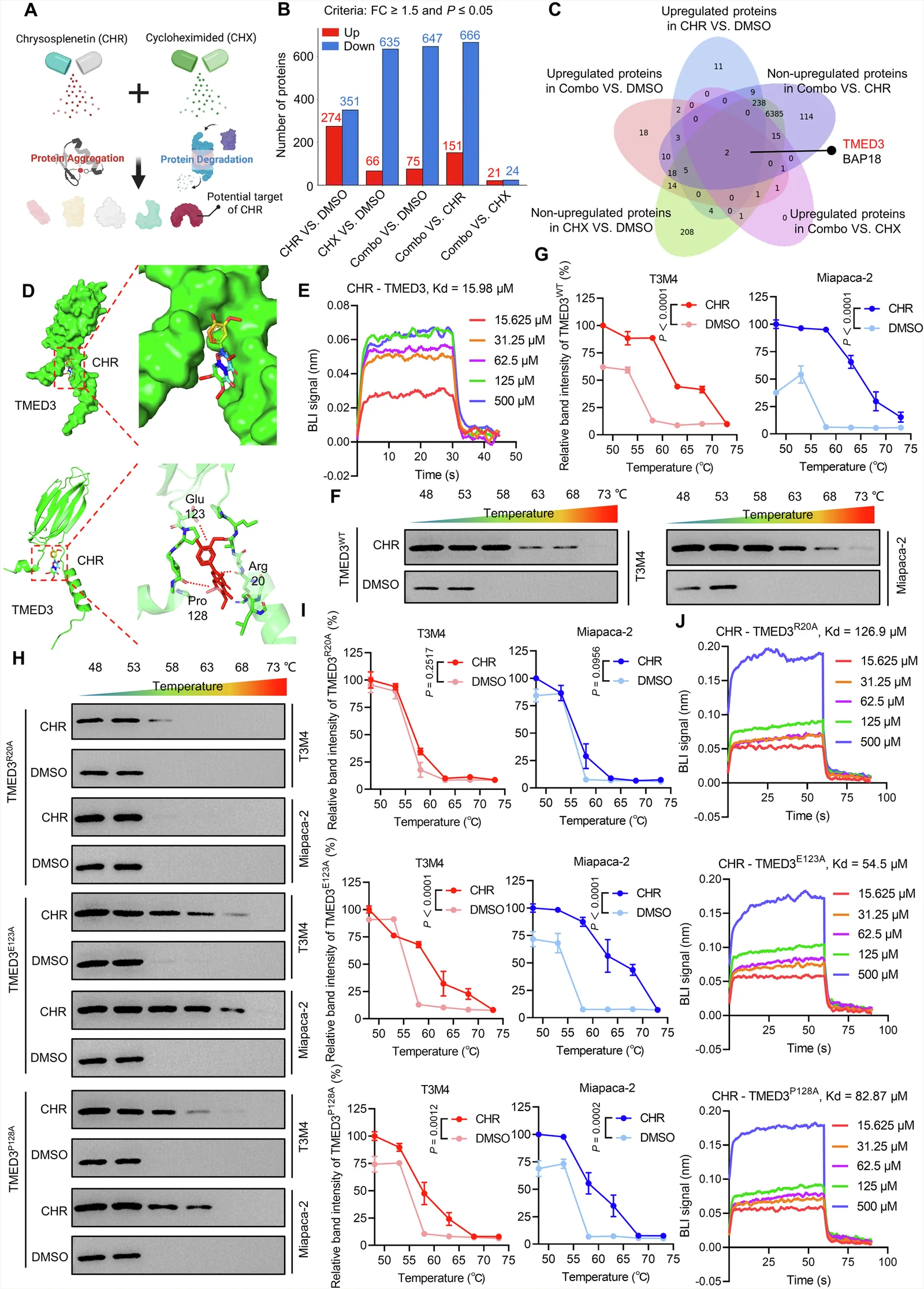
CHR directly binds to TMED3 in PC cells. (**A**) Schematic illustration of the protein aggregation profiling method combining CHR (20 μM) and CHX (50 μg) treatments for 24 h to identify CHR targets. (**B**) Summary of proteins significantly up- or down-regulated under different treatment conditions from proteomics. Student’t test was used to analyze statistical differences. The Benjamini–Hochberg procedure was used to adjust. *N* = 3 independent biological replicates. (**C**) Venn diagram showing the overlap of upregulated and non-upregulated proteins under different comparisons. (**D**) Molecular docking model of CHR binding to TMED3, with key interacting residues shown. (**E**) Bio-layer interferometry sensorgrams indicating direct binding of CHR to TMED3 with a Kd = 15.98 µM. (**F**, **G**) Immunoblotting analysis (**F**) and quantification of relative band intensity (**G**) of CETSA for CHR binding to wild‑type TMED3 proteins in TMED3‑KO cells. Two-way ANOVA was used to analyze statistical differences in (**G**). Mean with ± SD. *N* = 3 independent biological replicates. (**H**, **I**) Immunoblotting analysis (**H**) and quantification of relative band intensity (**I**) of CETSA for CHR binding to TMED3 R20A, E123A and P128A mutant proteins in TMED3-KO cells. Two-way ANOVA was used to analyze statistical differences in (**I**). Mean with ± SD. *N* = 3 independent biological replicates. (**J**) Bio‑layer interferometry sensorgrams indicating direct binding of CHR to TMED3 R20A, E123A and P128A mutant proteins with corresponding Kd values. Source data are available online for this figure.

Given that the core hallmark of ER stress is the accumulation of misfolded proteins, we hypothesize that the specific targets of CHR may be particular protein aggregates directly or indirectly caused by it. To discriminate between general ER stress effectors and CHR-specific targets, we employed the protein synthesis inhibitor cycloheximide (CHX) as a pharmacological tool. By globally inhibiting de novo protein synthesis, CHX forces cells to rely on endogenous degradation pathways (e.g., the autophagy-lysosome system and ubiquitin-proteasome system) to clear pre-existing proteins, a property widely utilized in protein half-life assays (Miao et al, 2023; Schneider-Poetsch et al, 2010). The combined treatment of CHX (blocking new protein synthesis) and CHR (inducing ER stress) enables the following causal inferences: (1) Under CHX treatment, general ER stress-related proteins will gradually degrade due to synthesis inhibition. (2) Only proteins whose degradation pathways are specifically impaired by CHR or that undergo abnormal conformational changes will exhibit significant accumulation due to the dual effects of “synthesis and degradation obstruction” (Fig. 5A). Thus, by applying proteomics to screen for such proteins, we significantly enhance the signal-to-noise ratio for identifying bona fide CHR targets.

As shown in Fig. 5B, numberous differentially expressed proteins were identified by two-by-two comparisons between the four groups using |fold change| ≥ 1.5 and *P* (adj) ≤ 0.05 as screening conditions. Cellular component and protein domain analysis reconfirmed earlier findings regarding ER and autophagy pathways (Appendix Fig. S9A,B). To exclude CHX-induced upregulated proteins, we plotted Venn diagrams based on the annotations shown in Fig. 5C and identified 2 potential targets, TMED3 and BPTF associated protein of 18 kDa (BAP18), that specifically responded to CHR-associated protein aggregation. To prioritize these candidates, we performed functional validation using siRNA-mediated knockdown. While knockdown of *BAP18* did not rescue CHR-induced cytotoxicity, knockdown of *TMED3* significantly attenuated the cytotoxic effects of CHR (Appendix Fig. S10A–D). This result establishes TMED3, but not BAP18, as the primary functional target relevant to CHR’s mechanism of action. We next investigated the mechanism underlying TMED3 upregulation. Integrated proteomic and transcriptomic analysis revealed that TMED3 was elevated at the protein level without a corresponding increase in its mRNA (Appendix Fig. S10E,F), indicating post-translational regulation. Mechanistically, neither co-treatment with the proteasome inhibitor MG132 nor assessment of poly-ubiquitination status showed that CHR stabilizes TMED3 by inhibiting its proteasomal degradation (Appendix Fig. S10G,H), suggesting a degradation-independent accumulation mechanism. This post-translational accumulation was consistently observed as elevated TMED3 levels in both in vitro and in vivo models following CHR treatment (Appendix Fig. S11A–G). To further characterize the properties of accumulated TMED3, we examined TMED3 under non-denaturing conditions. Generally, TMED3 migrated as a distinct band at approximately 100 kDa, consistent with its stable oligomeric form (Zheng et al, 2026). CHR treatment induced a marked intensification of the 100 kDa band along with the appearance of higher-molecular-weight species, indicating progressive oligomerization or aggregation (Appendix Fig. S11H). And immunofluorescence studies demonstrated significant colocalization of TMED3 with the ER marker ERp72 or SEC61β (Appendix Fig. S12A–I), and partially overlaped with the Golgi marker GM130, consistent with previous reports of TMED3’s ER localization (Emery et al, 2000; Jenne et al, 2002). To rule out the possibility that CHR exerts its effects through broad organellar toxicity, we analyzed the cellular component distribution of all proteins downregulated in CHR-treated samples. Notably, downregulated proteins showed no specific enrichment in ER, Golgi, or mitochondrial compartments, with the majority instead localized to the nucleus (Appendix Fig. S9C). Collectively, these data demonstrate that CHR induces the degradation-independent accumulation and ER-specific retention of TMED3, rather than generalized organellar disruption, which in turn triggers downstream ER stress.

**Figure EV2:**
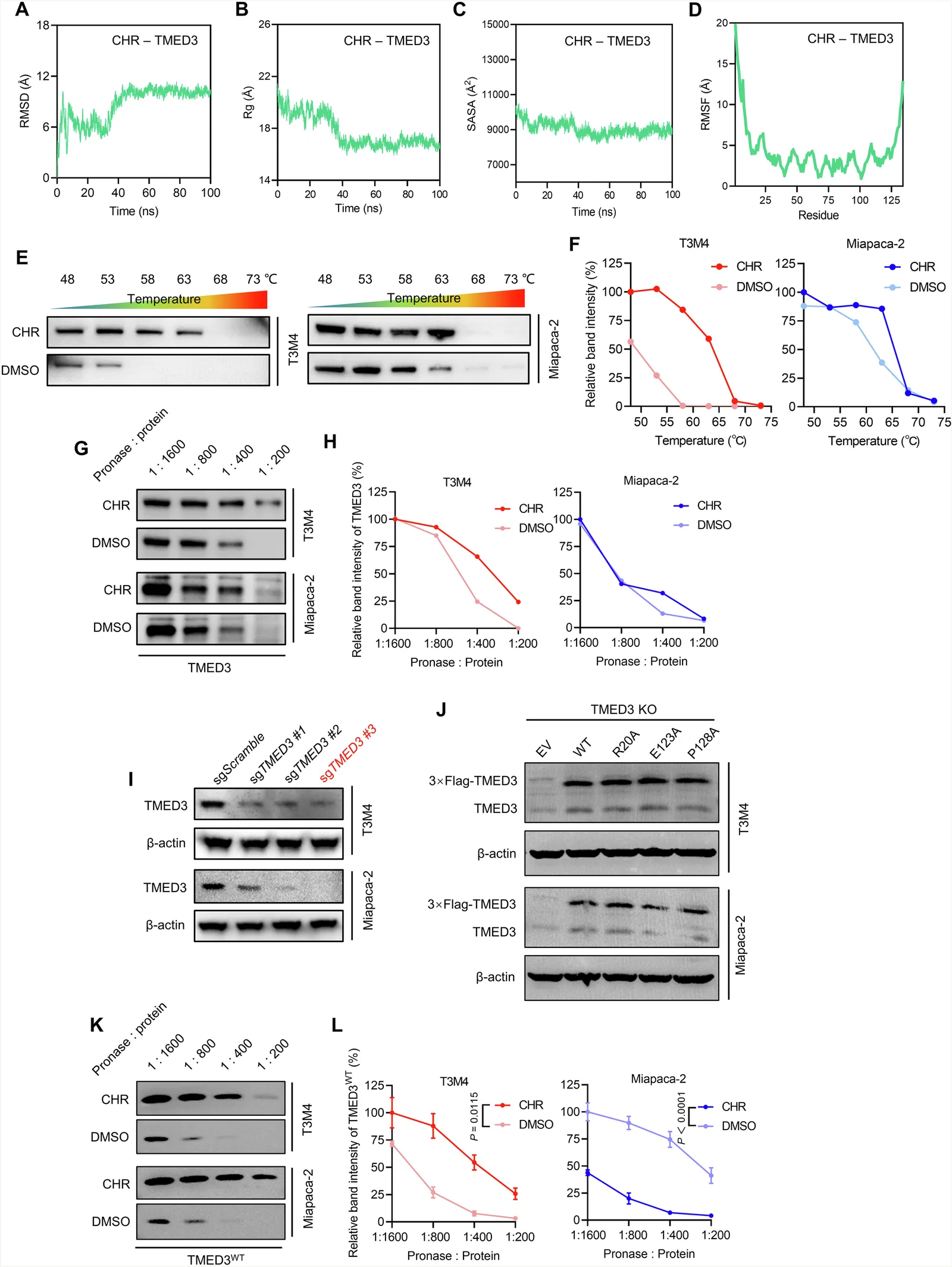
Validation of direct interaction between CHR and TMED3. (**A**) Molecular dynamics simulation showing root-mean-square deviation (RMSD) of the CHR-TMED3 complex. (**B**) Radius of gyration (Rg) plot of CHR-TMED3 complex over 100 ns. (**C**) Solvent accessible surface area (SASA) of the CHR-TMED3 complex during the 100 ns simulation. (**D**) Root mean square fluctuation (RMSF) analysis of each residue in the CHR-TMED3 complex. (**E**, **F**) Immunoblotting analysis (**E**) and quantification of relative band intensity (**F**) of cellular thermal shift assay (CETSA) for CHR binding to TMED3 in T3M4 and Miapaca-2 cells. (**G**, **H**) Immunoblotting analysis (**G**) and quantification of relative band intensity (**H**) of drug affinity responsive target stability (DARTS) for CHR binding to TMED3 in T3M4 and Miapaca-2 cells. (**I**) Immunoblotting analysis verifying TMED3 knockout efficiency in T3M4 and Miapaca‑2 cells generated using three independent sgRNAs targeting *TMED3*. (**J**) Immunoblotting analysis verifying TMED3 expression in TMED3‑KO T3M4 and Miapaca‑2 cells (generated with sgRNA#3) upon re‑expression of wild‑type TMED3 or TMED3 point mutants (R20A, E123A, P128A). (**K**, **L**) Immunoblotting analysis (**K**) and quantification of relative band intensity (**L**) of DARTS for CHR binding to wild‑type TMED3 proteins in TMED3‑KO cells. Two-way ANOVA was used to analyze statistical differences in (**L**). Mean with ± SD. *N* = 3 independent biological replicates. Source data are available online for this figure.

**Figure EV3:**
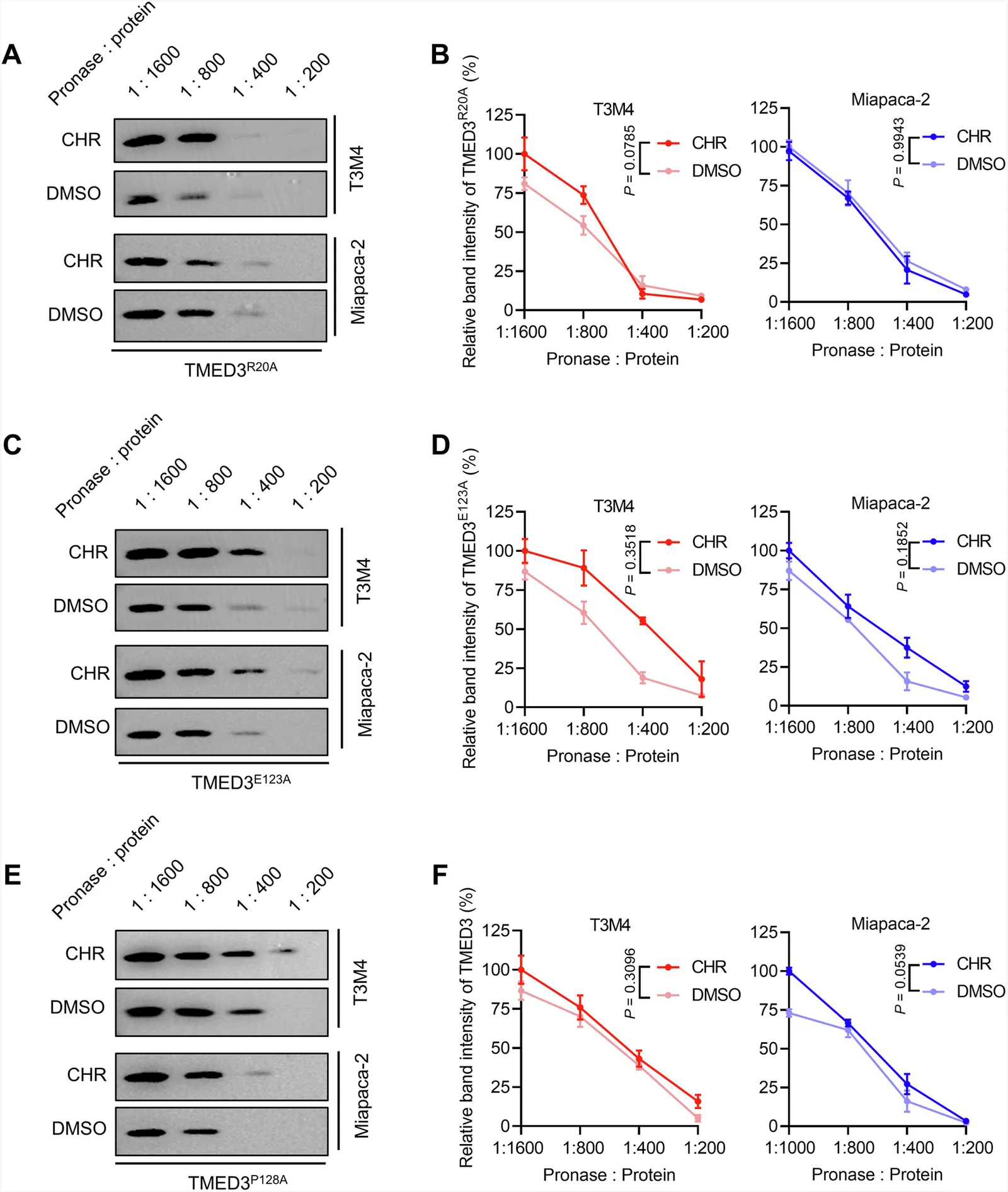
Identification of key binding residues mediating direct interaction between CHR and TMED3. (**A**, **B**) Immunoblotting analysis (**A**) and quantification of relative band intensity (**B**) of DARTS for CHR binding to TMED3 R20A mutant proteins in TMED3-KO cells. Two-way ANOVA was used to analyze statistical differences in (**B**). Mean with ± SD. *N* = 3 independent biological replicates. (**C**, **D**) Immunoblotting analysis (**C**) and quantification of relative band intensity (**D**) of DARTS for CHR binding to TMED3 E123A mutant proteins in TMED3-KO cells. Two-way ANOVA was used to analyze statistical differences in (**D**). Mean with ± SD. *N* = 3 independent biological replicates. (**E**, **F**) Immunoblotting analysis (**E**) and quantification of relative band intensity (**F**) of DARTS for CHR binding to TMED3 P128A mutant proteins in TMED3-KO cells. Two-way ANOVA was used to analyze statistical differences in (**F**). Mean with ± SD. *N* = 3 independent biological replicates. Source data are available online for this figure.

In order to verify whether CHR can bind directly to TMED3, we first performed molecular docking to elucidate the binding site of CHR to TMED3. The results identified specific interactions between CHR and TMED3, revealing hydrogen bond formation with Arg20, Glu123, and Pro128 residues (Fig. 5D). Subsequently, molecular dynamic simulations were performed to assess the stability of the interactions between CHR and TMED3. The root-mean-square deviation (RMSD) values showed that the complex system reached equilibrium after 40 ns and finally fluctuated up and down at 10.4 Å, indicating that CHR exhibited high stability when bound to TMED3 (Fig. EV2A). Further analysis revealed that the radius of gyration (Rg) value and solvent accessible surface area (SASA) of the complex system showed slight fluctuations, indicating that the small molecule-target protein complexes underwent conformational changes during the movement (Fig. EV2B,C). And the root mean square fuctuation (RMSF) values for CHR and TMED3 were relatively low (mostly below 5 Å), suggesting less flexible and more stable (Fig. EV2D). To experimentally validate this predicted interaction, we performed a series of biochemical binding assays. CETSA analysis confirmed that CHR significantly stabilized TMED3 against thermal denaturation in living cells (Fig. EV2E,F). DARTS analysis showed that CHR binding enhanced TMED3 resistance to pronase digestion (Fig. EV2G,H). Furthermore, we conducted bio-layer interferometry (BLI) assays using high precision streptavidin biosensors. As shown in Fig. 5E, quantitative BLI measurements demonstrated concentration-dependent CHR-TMED3 interaction with a Kd of 15.98 μM, establishing an accurate binding affinity. To confirm the functional relevance of the computationally identified binding interface, we generated TMED3 point mutants by substituting key residues (Arg20, Glu123, and Pro128) with alanine (R20A, E123A, and P128A). Firstly, we generated stable TMED3-knockout (TMED3‑KO) cell lines using CRISPR/Cas9 technology. Three independent sgRNA sequences were screened, and the clone with the highest knockout efficiency was selected for all subsequent experiments (Fig. EV2I,J). When these mutants were subjected to the same biochemical assays (CETSA, DARTS, and BLI), they consistently failed to show the CHR-induced stabilization or binding observed with wild-type TMED3 (Figs. 5F–J, EV2K,L and EV3A–F). Notably, the Arg20 mutant exhibited the most striking loss of such activities, providing direct genetic evidence that these residues, especially Arg20, are essential for CHR recognition. We also assessed CHR binding to other ER transmembrane proteins (PERK, IRE1α, SEC61β) in parallel CETSA experiments to evaluate specificity. As shown in Appendix Fig. S13A–F, none of these control proteins exhibited the significant thermal stabilization in the presence of CHR that was consistently observed for TMED3. This demonstrates that CHR selectively stabilizes TMED3, distinguishing it from broad-spectrum ER stressors like DTT or thapsigargin. Collectively, these data establish that CHR directly and specifically binds to TMED3, with Arg20 serving as the core pivotal residue, together with Glu123 and Pro128, to mediate this interaction. This interaction stabilizes TMED3, and leads to aberrant accumulation in the ER, thereby inducing ER stress in PC cells.

### Disruption of TMED3 expression impairs the efficacy of CHR

**Figure 6:**
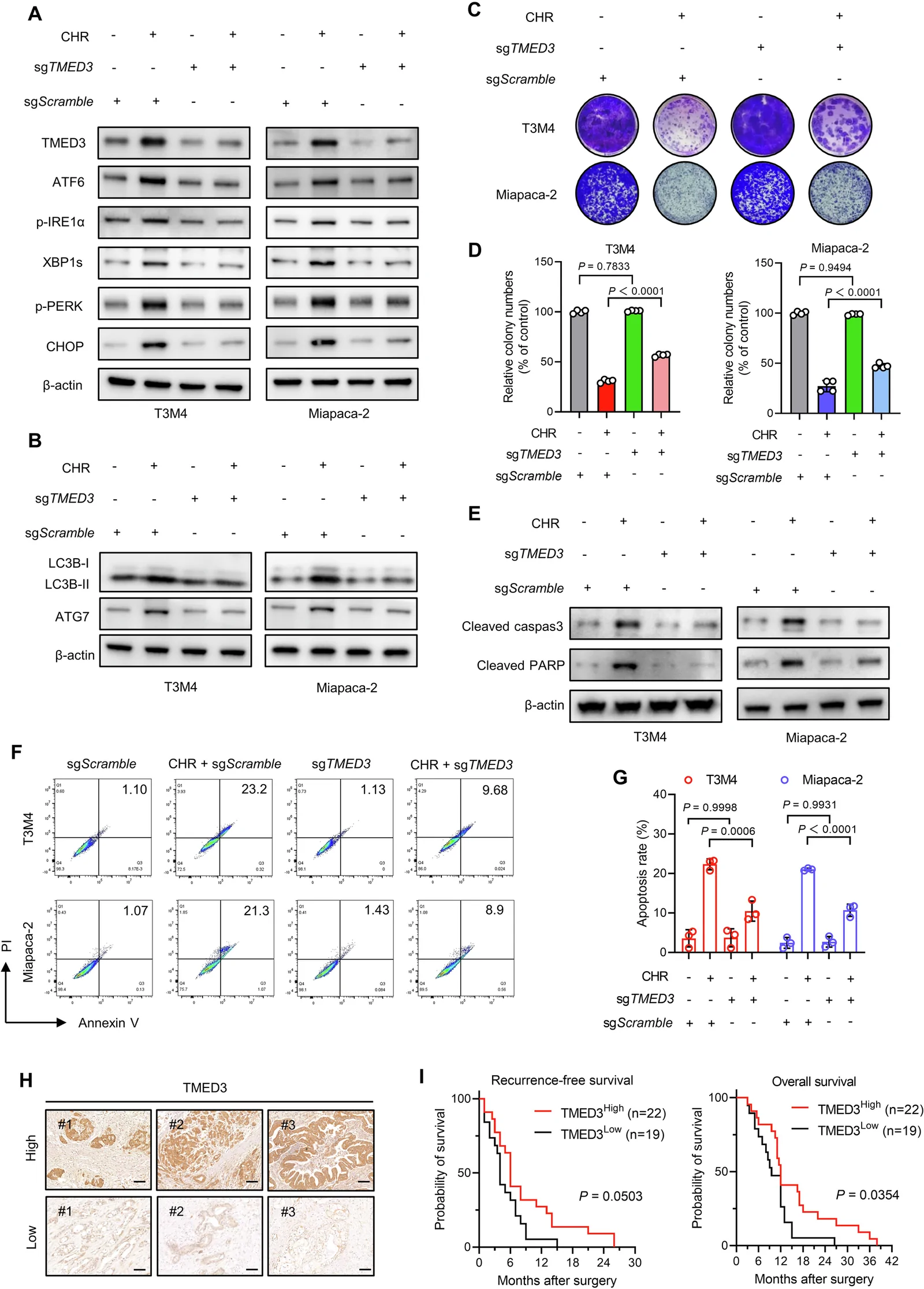
Disruption of TMED3 expression impairs the efficacy of CHR. (**A**, **B**) Immunoblotting analysis of ER stress markers (**A**) and autophagy markers (**B**) in wild‑type and TMED3‑KO T3M4 and Miapaca-2 cells treated with CHR (10 μM) for 48 h. (**C**, **D**) Representative images (**C**) and quantification of colony numbers (**D**) of wild‑type and TMED3‑KO T3M4 and Miapaca-2 cells treated with CHR. One-way ANOVA was used to analyze statistical differences in (**D**). Mean with ± SD. *N* = 4 independent biological replicates. (**E**) Immunoblotting analysis of CC3 and cleaved PARP in wild‑type and TMED3‑KO T3M4 and Miapaca-2 cells treated with CHR for 48 h. (**F**, **G**) Flow cytometric analysis (**F**) and quantification (**G**) of apoptosis rate in wild‑type and TMED3‑KO T3M4 and Miapaca-2 cells treated with CHR for 48 h. One-way ANOVA was used to analyze statistical differences in (**G**). Mean with ± SD. *N* = 3 independent biological replicates. (**H**) Representative images of immunohistochemical staining for TMED3 in PDAC patient tumor tissueswith three representative cases each for high‑ and low‑TMED3 expression groups. Scale bar: 100 μm. (**I**) Kaplan–Meier analysis of PDAC patients based on TMED3 expression levels derived from tissue microarray cohort (**H**). Log-rank test was used to analyze statistical differences. Source data are available online for this figure.

To elucidate the role of TMED3 on the CHR-induced phenotype, we treated the TMED3-KO PC cells with CHR. As shown in Fig. 6A, downregulating TMED3 expression markedly attenuated CHR-induced UPR activation, as demonstrated by reduced levels of PERK, p-PERK, IRE1α, p-IRE1α, ATF6, XBP1s and CHOP. In addition, the increased autophagy markers following CHR treatment were reversed in TMED3-KO cells (Fig. 6B). Functionally, TMED3 knockout rendered PC cells resistant to CHR’s cytotoxic effects, as evidenced by restored cell viability (Appendix Fig. S14A) and colony formation capacity (Fig. 6C,D). As expected, TMED3 knockout also significantly reduced the protein expression of CC3 and cleaved PARP in CHR-treated PC cells (Fig. 6E). Consistently, the impairment of TMED3 expression decreased CHR-triggered apoptotic cell populations (Fig. 6F,G). To further confirm the functional importance of the specific CHR-TMED3 binding interface, we performed functional analyses using pre-established TMED3-knockout cells reconstituted with wild-type TMED3 or its binding-deficient point mutants. As shown in Appendix Fig. S14B, CHR treatment markedly increased the protein level of wild-type TMED3, consistent with our earlier observations, whereas this upregulation was substantially attenuated in cells expressing mutant TMED3. Functionally, re-expression of wild-type TMED3 fully restored cellular sensitivity to CHR, while cells bearing point mutants exhibited only partial responsiveness and remained partially resistant to CHR-induced cell death (Appendix Fig. S14C). These results provide direct evidence that CHR binding to TMED3 at this interface is essential for TMED3 upregulation and subsequent cytotoxicity. To further evaluate the clinical relevance of TMED3 in PC, we conducted survival analysis via the online Kaplan–Meier Plotter platform (https://kmplot.com/analysis/), which integrates pooled clinical data from 16 independent cohorts (Posta and Gyorffy, 2023). This analysis revealed that high TMED3 expression correlates with improved overall survival in PC patients (Appendix Fig. S14D). Importantly, these findings were also validated in an independent tissue microarray cohort (Fig. 6H,I). Collectively, our data demonstrate that TMED3 plays an essential role in mediating CHR-induced UPR activation and apoptosis.

**Figure EV4:**
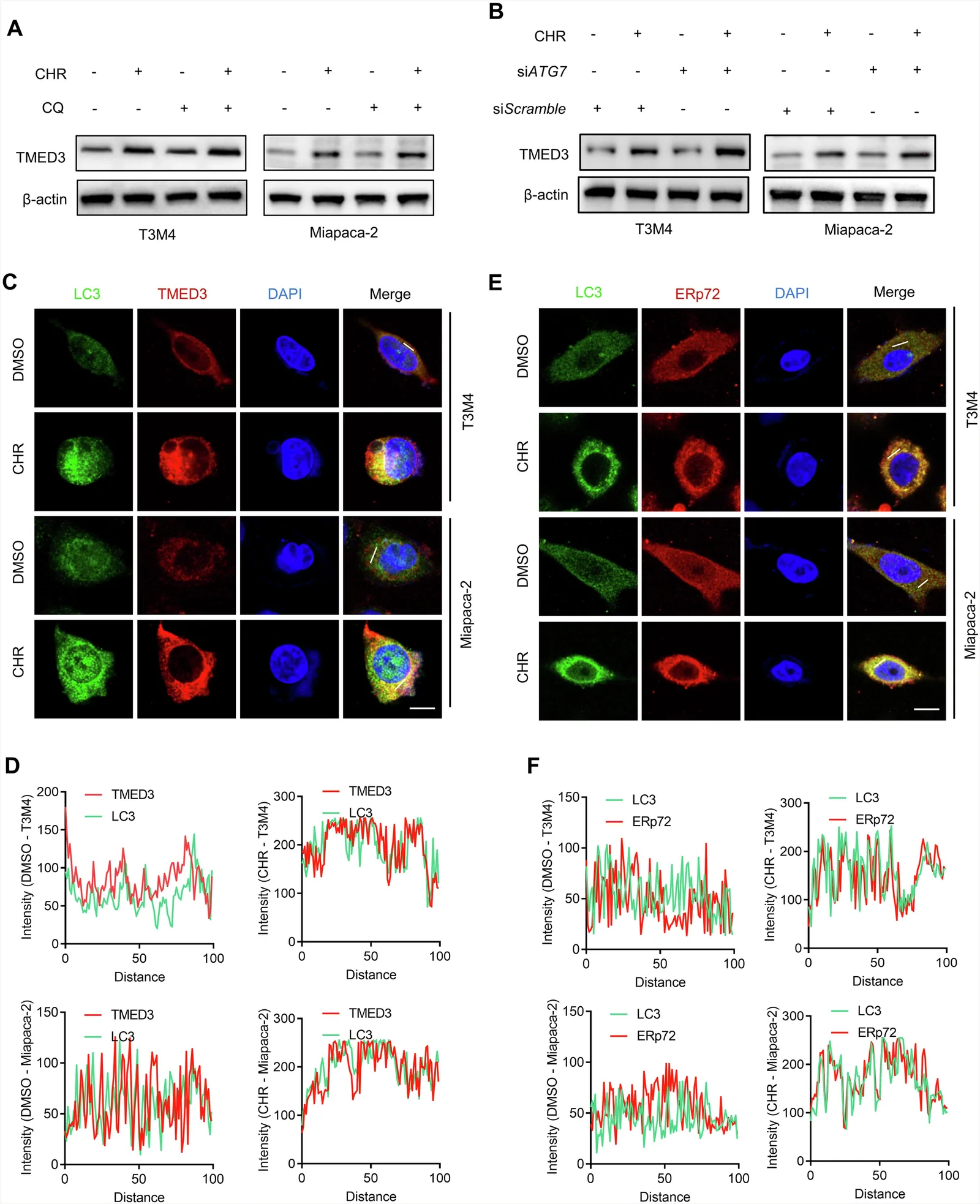
CHR-mediated autophagy may be in response to TMED3 protein accumulation. (**A**, **B**) Immunoblotting analysis of TMED3 expression in T3M4 and Miapaca-2 cells treated with CHR (10 μM) in presence or absence of CQ (10 μM) (**A**), or after transfected with si*ATG7* or si*Scramble* (B). (**C**, **D**) Representative images (**C**) and colocalization analysis of LC3 and TMED3 (**D**) of immunofluorescent staining for LC3 (green), TMED3 (red) and DAPI (blue) in T3M4 and Miapaca-2 cells treated with DMSO or CHR, scale bar: 10 μm. (**E**, **F**) Representative images (**E**) and colocalization analysis of LC3 and ERp72 (**F**) of immunofluorescent staining for LC3 (green), ERp72 (red) and DAPI (blue) in T3M4 and Miapaca-2 cells treated with DMSO or CHR, scale bar: 10 μm. Source data are available online for this figure.

Given that the anti-PC activity of CHR is determined by the TMED3 aggregation on the ER and that previous data have elucidated the protective role of CHR-mediated autophagy, we wondered whether autophagy activation might represent an adaptive response to specifically clear aberrant TMED3 accumulation. Supporting this notion, inhibition of autophagy pathways using CQ or si*ATG7* led to further TMED3 protein accumulation in CHR-treated cells, suggesting autophagic degradation of TMED3 aggregates (Fig. EV4A,B). Furthermore, immunofluorescence analysis revealed significant colocalization of LC3B with both TMED3 and ERp72 following CHR treatment, implying ER-localized TMED3 aggregate engulfed by the autophagosomes (Fig. EV4C–F). Collectively, these findings indicate CHR-induced autophagy as a potential process for scavenging TMED3 accumulation. However, the detailed underlying mechanisms warrant further investigation.

### CHR combined with autophagy inhibition or chemotherapy enhances antitumor efficacy in preclinical models

Our previous findings established that autophagy inhibition potentiates CHR’s anti-tumor activity. Beyond this, gemcitabine (GEM)-based chemotherapy, which serves as a first-line regimen for PC treatment, exerts its antitumor effects via a distinct signaling pathway. Given this, we hypothesized that GEM and CHR might synergistically improve therapeutic outcomes in PC. To this end, we co-treated T3M4 and Miapaca-2 cells with GEM in combination with CHR based on a dose-matrix approach. Utilizing cell viability assays and the zero interaction potency (ZIP) model (Ianevski et al, 2022), we identified 50 nM GEM + 2.5 μM CHR as the optimal synergistic combination (Appendix Fig. S15A,B). Consistently, the proliferation of PC cells was markedly inhibited by this regimen, as evidenced by a significant reduction in colony formation (Appendix Fig. S15C,D).

**Figure 7:**
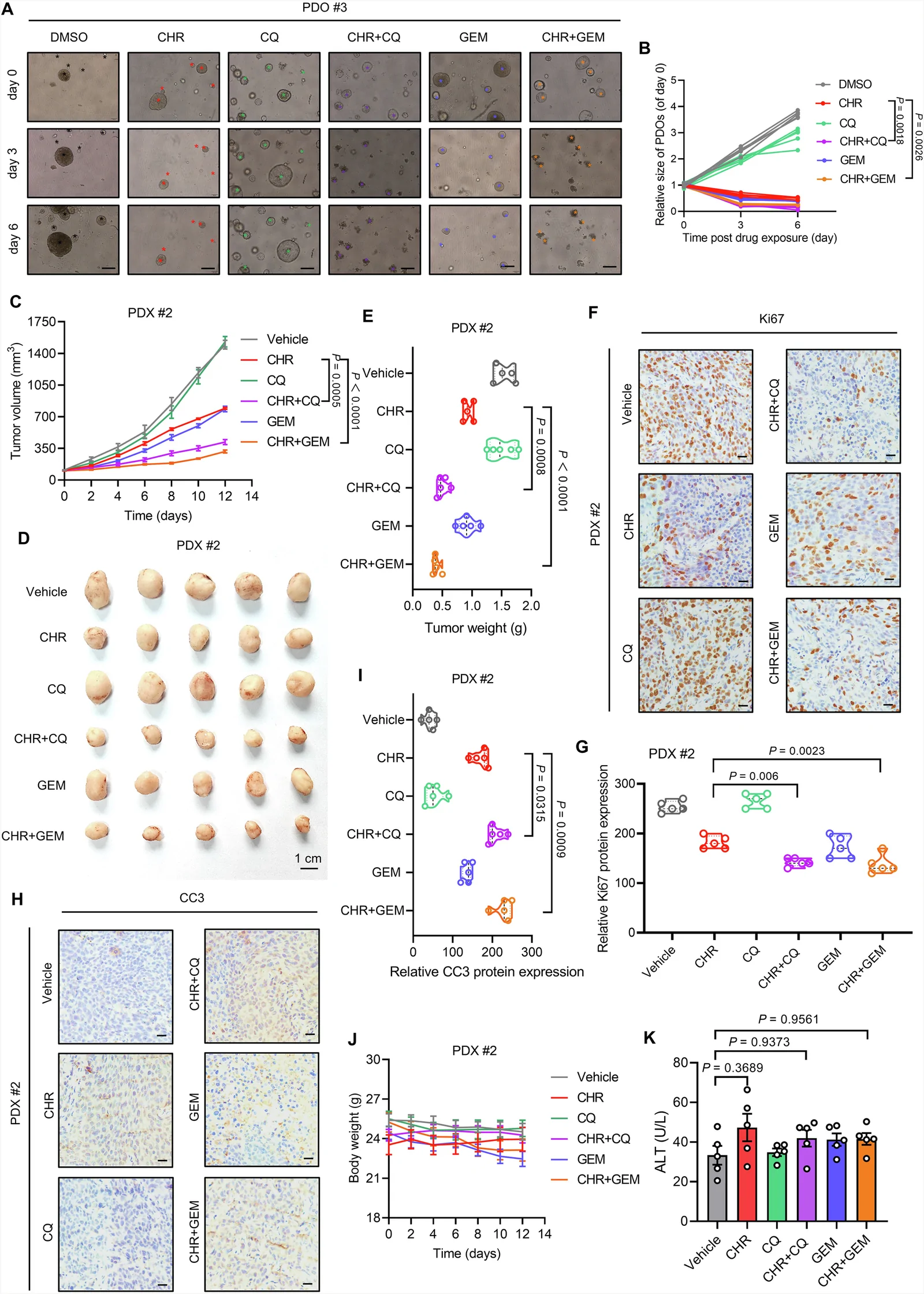
CHR combined with autophagy inhibition or chemotherapy enhances antitumor efficacy in preclinical models. (**A**, **B**) Representative images (**A**) and quantification of relative sizes of PDO #3 (**B**) after DMSO, CHR (20 μM), CQ (10 μM), CHR (20 μM) + CQ (10 μM), GEM (100 nM) and CHR (20 μM) + GEM (100 nM) treatment over time, scale bar: 75 μm. * denoted the PDO of interest, with matching color in (**B**). Two-way ANOVA was used to analyze statistical differences in (**B**). *N* = 5 independent organoids. (**C**–**E**) Tumor volumes (**C**), images (**D**) and weights (**E**) of PDX #2 xenografts treated with vehicle, CHR (25 mg/kg), CQ (25 mg/kg), CHR (25 mg/kg) + CQ (25 mg/kg), GEM (15 mg/kg), CHR (25 mg/kg) + GEM (15 mg/kg) for 12 days. Two-way ANOVA was used to analyze statistical differences in (**C**). Mean with ± SEM. One-way ANOVA was used to analyze statistical differences in (**E**). *N* = 5 independent mice. (**F**–**I**) Representative images (**E**, **H**) and quantification (**G**, **I**) of immunohistochemical staining for Ki67 and CC3 of PDX #2 xenografts treated as in (**C**), scale bar = 20 μm. One-way ANOVA was used to analyze statistical differences in (**G**, **I**). *N* = 5 independent mice. (**J**) Mice body weight change of PDX #2 xenografts throughout the treatment period. Mean with ± SEM. *N* = 5 independent mice. (**K**) Serum ALT levels of PDX #2 xenografts at the endpoint of treatment. One-way ANOVA was used to analyze statistical differences. Mean with ± SEM. *N* = 5 independent mice. Source data are available online for this figure.

To further evaluate CHR-based combination strategies, we generated new sets of PDOs and PDX models for this therapeutic evaluation. Mirroring our findings in PC cell lines, either CQ or GEM obviously enhanced CHR’s anti-tumor efficacy compared to CHR monotherapy in PDOs (Fig. 7A,B). In PDX models, the combination treatment groups demonstrated markedly suppressed tumor growth rate, with significantly reduced final tumor volumes and weights relative to all control groups (Fig. 7C–E). Immunohistochemical analysis revealed characteristic molecular signatures of CHR activity, including strong p-IRE1α, LC3B and TMED3 expression in treated xenografts (Appendix Fig. S16A–F). Importantly, combination therapies exhibited enhanced therapeutic effects, as evidenced by decreased Ki67 proliferation markers and increased CC3 apoptotic signals compared to single-agent treatments (Fig. 7F–I).

**Figure EV5:**
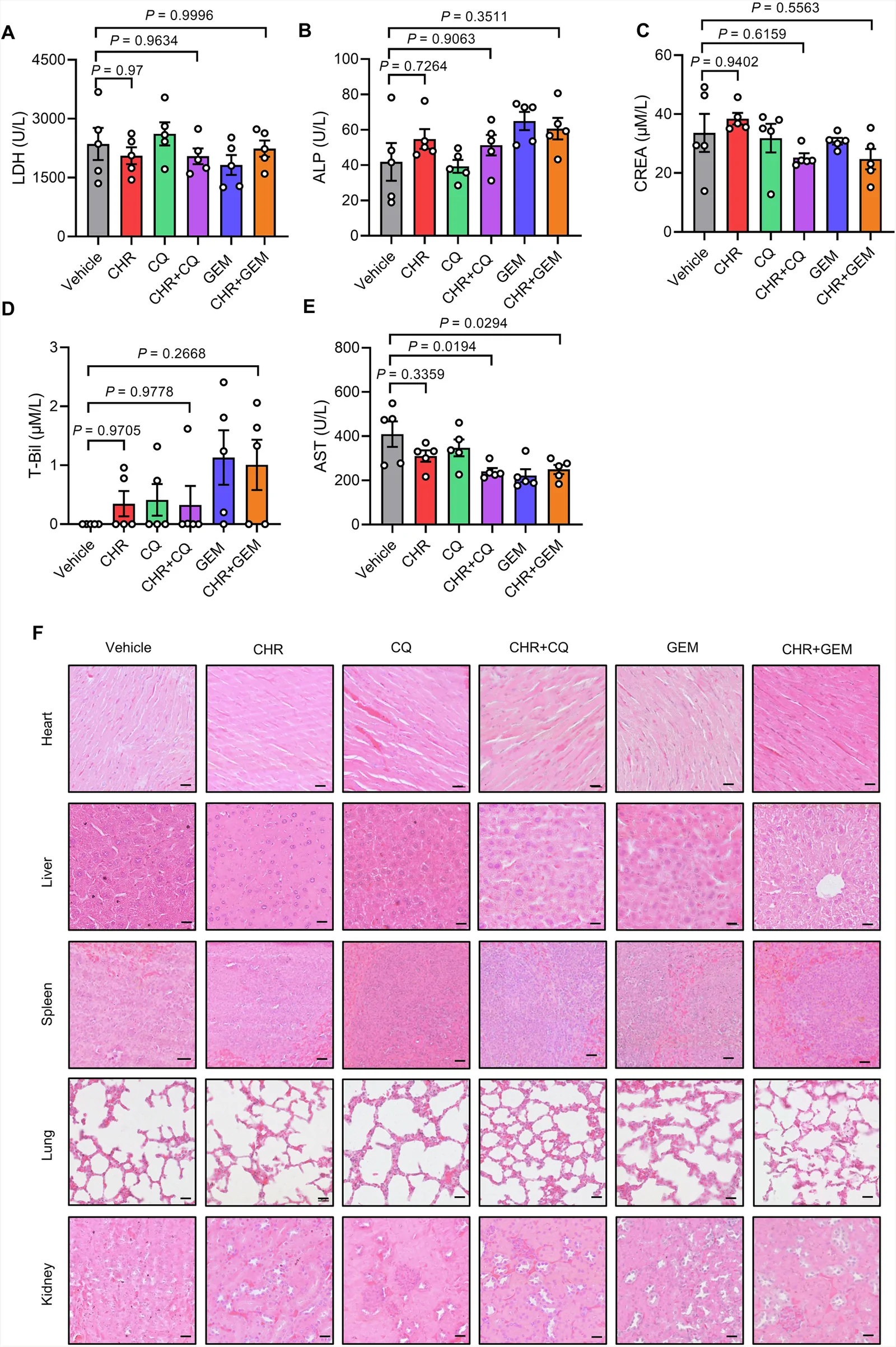
CHR-based combination therapy has no obvious toxic side effects. (**A**–**E**) Serum levels of LDH (**A**), ALP (**B**), CREA (**C**), T-Bil (**D**) and AST (**E**) in PDX #2 xenografts treated with vehicle, CHR (25 mg/kg), CQ (25 mg/kg), CHR (25 mg/kg) + CQ (25 mg/kg), GEM (15 mg/kg), CHR (25 mg/kg) + GEM (15 mg/kg) for 12 days. One-way ANOVA was used to analyze statistical differences. Mean with ± SEM. *N* = 5 independent mice. (**F**) Representative H&E staining images of major organs (heart, liver, spleen, lung, and kidney) from mice treated with the indicated treatments, scale bars: 20 μm. Source data are available online for this figure.

Then, we assessed the in vivo safety profile of CHR-based combination therapy through comprehensive toxicity evaluations. Longitudinal monitoring revealed no significant alterations in body weight between treatment groups (Fig. 7J), indicating good tolerability. Serum biochemistry analysis demonstrated good hepatic and renal safety profiles, with several measured parameters (ALT, LDH, ALP, CREA, T-Bil) remaining within normal ranges compared to controls (Figs. 7K and EV5A–D). Although AST levels showed a modest reduction, this change remained within physiological variation (Fig. EV5E). Histopathological examination of major organs (heart, liver, spleen, lungs, kidneys) revealed no treatment-related abnormalities across all groups (Fig. EV5F). Altogether, these findings demonstrate that CHR-containing regimens enhance anti-tumor efficacy in preclinical models without inducing overt systemic toxicity, providing a basis for further investigation of combination strategies targeting the TMED3–ER stress axis.

## Discussion

Despite significant advances in conventional cancer therapies including surgery, radiotherapy, and chemotherapy, treatment limitations remain evident through persistently high mortality rates, particularly in aggressive malignancies like PC, often termed the “king of cancers” due to its poor prognosis (Stoop et al, 2025). This clinical challenge underscores the urgent need for novel therapeutic approaches. Natural products have emerged as a crucial resource for anticancer drug discovery, as evidenced by historically significant plant-derived compounds like vinca alkaloids (vincristine, vinblastine), taxanes (paclitaxel), and camptothecin derivatives that form the backbone of modern chemotherapy (Mann, 2002). Supporting their therapeutic potential, the World Health Organization (WHO) estimates approximately 80% of populations in developing regions rely on traditional plant-based medicine, while remarkably over 50% of FDA-approved anticancer agents are natural products or their derivatives (Asma et al, 2022; Bishayee and Sethi, 2016). Capitalizing on this rich resource, we conducted high-throughput screening of a 411-compound natural product library for PC treatment, which identified CHR, a bioactive polymethoxylated flavonoid. In the present study, we characterize CHR as a chemical probe to investigate its anti-tumor mechanism and identify novel therapeutic vulnerabilities in PC.

Elucidating the molecular targets of bioactive natural products is essential for understanding their mechanisms of action and identifying potential therapeutic vulnerabilities. Recent progress in chemical proteomics has provided powerful tools for this purpose, with current methodologies broadly classified into two paradigms: covalent modification-based and interaction-dependent approaches (Chen et al, 2020). The former strategy, while widely implemented, faces inherent constraints including potential perturbation of native bioactivity and structural incompatibility with certain natural product scaffolds. These limitations have motivated alternative development of label-free techniques including DARTS and CETSA, which monitor target engagement through biophysical properties rather than molecular labeling (Lomenick et al, 2009; Martinez Molina et al, 2013). In this study, through an endogenous degradation-activation-based proteomic sequencing for potential UPR-responsive proteins of CHR, we identified TMED3 as a CHR target, then validated this interaction using several complementary approaches. DARTS demonstrated reduced trypsin sensitivity of TMED3 at varying enzyme concentrations, indicating direct binding protection. And CETSA showed enhanced thermal stability in the living cells. These findings align with previous natural product studies employing similar strategies for target identification, including Bruceine D/ICAT (Huang et al, 2021), gambogic amide/WDR1 (Qu et al, 2023), and periplocin/LGALS3 (Wang et al, 2023) interactions. In addition, we confirmed the direct interaction between CHR and human recombinant TMED3 protein using BLI, a technique capable of measuring biomolecular interaction kinetics and widely used to assess protein-drug binding (Bates et al, 2025; Bellail et al, 2021). Computational validation through molecular docking and dynamics simulations (Śledź and Caflisch, 2018) also revealed stable binding conformations and favorable interaction energetics between CHR and TMED3. Collectively, our three-dimensional validation strategy, combining endogenous cellular interactions (DARTS/CETSA), exogenous biophysical measurements (BLI), and in silico structural analyses, provides conclusive evidence establishing TMED3 as a direct molecular target of CHR in PC.

TMED3, a pivotal member of the type I transmembrane protein family, orchestrates cargo trafficking in the early secretory pathway by serving as a receptor for COPI vesicle formation and secretory cargo recruitment (Schimmöller et al, 1995). Emerging evidence revealed its context-dependent functions in cancer progression (Mishra et al, 2019; Qiao et al, 2025). While elevated TMED3 expression promoted tumor aggressiveness in osteosarcoma and breast cancer through Wnt/β-catenin and ERK/MAPK signaling (Xu et al, 2021; Zhang et al, 2020), it conversely suppressed metastasis in colon cancer by modulating WNT-TCF activity (Duquet et al, 2014). Our study unveiled a novel mechanism wherein CHR directly binds TMED3, inducing its aggregation and triggering TMED3-mediated ER stress. Interestingly, a recent study supported our novel findings regarding the involvement of TMED3 in ER stress, specifically that TMED3 acted as a key regulator of ER stress-associated protein secretion. In this context, the TMED2/3/9/10 complex facilitated the surface translocation of core-glycosylated membrane cargos (Park et al, 2022). However, the precise molecular cascade linking TMED3 aggregation to canonical UPR sensor activation remains to be fully elucidated. Specifically, whether this occurs directly or through secondary disruption of ER-Golgi trafficking, and how individual UPR branches contribute to the downstream phenotype. Collectively, our findings identify a previously unrecognized TMED3-dependent regulatory axis governing ER homeostasis in PC, highlighting TMED3 as a druggable vulnerability warranting further investigation. Meanwhile, the dual and context-dependent functions of TMED3 in different malignancies warrant comprehensive exploration in future studies.

To date, targeting unresolved or lethal ER stress, or disrupting cytoprotective UPR mechanisms, represents a promising therapeutic strategy for cancer treatment. For instance, cannabigerol, a non-psychoactive phytocannabinoid derived from Cannabis sativa, exerted a potent antiproliferative effect on PC cells, concurrently inducing apoptosis and ferroptosis via ER stress-driven activation of the IRE1α pathway (Park et al, 2026). Similarly, diphyllin was shown to enhance SERCA2-dependent apoptosis in lung cancer by impairing ER calcium homeostasis, thereby exacerbating ER stress and inducing mitochondrial dysfunction (Xu et al, 2024b). On the other hand, genetic ablation of PERK in ER-stressed tumor cells activated SEC61β-mediated paraptosis, eliciting immunogenic cell death and systemic antitumor immunity (Mandula et al, 2022). Additionally, the ER stress sensor IRE1α served as a critical immune checkpoint in triple-negative breast cancer (TNBC), dampening the immunostimulatory effects of taxane chemotherapy. Pharmacological inhibition of IRE1α in Trp53-deficient TNBC models enabled taxane-induced accumulation of double-stranded RNAs, which were recognized by ZBP1, subsequently triggering NLRP3 inflammasome activation and GSDMD-dependent pyroptosis (Xu et al, 2024a). In line with these studies, our work confirms that CHR-driven TMED3 aggregation provokes sustained ER stress and UPR activation, thereby suppressing PC malignant progression. Although our preclinical data demonstrate that CHR combined with gemcitabine yields improved anti-tumor efficacy, the core translational significance of this study lies in the identification of the druggable TMED3–ER stress axis. Notably, CHR treatment disrupted ER homeostasis by inducing aberrant accumulation of TMED3, which simultaneously triggered adaptive autophagy as a compensatory survival mechanism, partially counteracting its anti-tumor effects. Inhibition of autophagy significantly enhanced the effect of CHR, whereas direct knockout of TMED3 did the opposite. From this, it was not difficult to speculate that autophagy was activated to degrade CHR-mediated TMED3 accumulation in the ER. And the hypothesis preliminarily was supported by our data (Fig. EV4). Indeed, the autophagic degradation of target proteins has been a focal point of numerous studies. Recently, a study highlighted tetrandrine, a bis-benzylisoquinoline alkaloid, as an immunological adjuvant that enhances antitumor immunity by inhibiting the autophagic degradation of the STING1 protein (Zhang et al, 2025). In addition, baicalein, a monomer derived from traditional Chinese medicine, has been shown to potentiate the CD274-LC3 interaction, thereby facilitating the autophagic-lysosomal degradation of CD274 and bolstering T cell-mediated antitumor immunity (Hao et al, 2025). Overall, a more in-depth understanding of the mechanisms underlying the UPR is crucial for the rational design of effective therapeutic interventions that can address current clinical challenges and ultimately improve patient outcomes.

Our study demonstrates that CHR suppresses PC progression by inducing TMED3 aggregation, which disrupts ER homeostasis and triggers lethal ER stress. Beyond delineating the anti-tumor mechanism of CHR, the central contribution of this work is the identification of TMED3 as a previously unrecognized druggable vulnerability in PC. Perturbation of TMED3 destabilizes ER proteostasis and activates pro-death UPR signaling, providing a novel mechanistic basis for ER stress-targeted PC therapy. Our work also highlights the modulatory role of UPR-associated autophagy in shaping therapeutic responses, offering a promising combinatorial strategy to improve treatment efficacy. Future validation in orthotopic or genetically engineered models, together with systematic PK/PD and toxicity profiling, will be essential to assess the translational relevance of this regulatory axis.

## Methods

**Table:** Reagents and tools table

Reagent/Resource	Reference or Source	Identifier or Catalog Number
**Experimental models**		
T3M4 (human pancreatic cancer cell line)	ATCC	CVCL_4056
Miapaca-2 (human pancreatic cancer cell line)	ATCC	CVCL_0428
PANC-1 (human pancreatic cancer cell line)	ATCC	CVCL_0480
SW1990 (human pancreatic cancer cell line)	ATCC	CVCL_1723
SUIT-2 (human pancreatic cancer cell line)	ATCC	CVCL_3172
BALB/c nude mice (7 weeks, male)	GemPharmatech (Jiangsu, China)	N/A
Patient-derived xenografts (PDX)	This study (patient-derived)	Fig. 1 (PDX #1) and Fig. 7 (PDX #2)
Patient-derived organoids (PDO)	This study (patient-derived)	Fig. 1 (PDO #1 / #2) and Fig. 7 (PDO #3)
**Recombinant DNA**		
pLV3-U6-puromycin (sgRNA targeting human *TMED3*)	This study (constructed by MIAOLING PLASMID)	knockout validation in Fig. EV2
pLV3-CMV-hygromycin (*3×FLAG-TMED3* WT/R20A/E123A/P128A)	This study (constructed by MIAOLING PLASMID)	expression validation in Fig. EV2 and Appendix Fig. S14
psPAX2 (lentiviral packaging plasmid)	Addgene	Cat. # 12260
pMD2.G (lentiviral packaging plasmid)	Addgene	Cat. # 12259
mRFP-GFP-LC3 (autophagy flux reporter)	This study (plasmid from lab stock)	Fig. 3
**Antibodies**		
Cleaved caspase-3 (Rabbit mAb)	Cell Signaling Technology	Cat. # 9661; RRID: AB_2341188
Cleaved PARP (Rabbit mAb)	Cell Signaling Technology	Cat. # 9541; RRID: AB_331426
LC3B (Mouse mAb)	Cell Signaling Technology	Cat. # 83506; RRID: AB_2800018
ATG7 (Rabbit mAb)	Cell Signaling Technology	Cat. # 8558S; RRID: AB_10831194
XBP1s (Rabbit mAb)	Cell Signaling Technology	Cat. # 40435; RRID: AB_2891025
CHOP (Mouse mAb)	Santa Cruz Biotechnology	Cat. # sc-56107; RRID: AB_783507
BIP/GRP78 (Mouse mAb)	Santa Cruz Biotechnology	Cat. # sc-376768; RRID: AB_2819145
IRE1α (Mouse mAb)	Santa Cruz Biotechnology	Cat. # sc-390960; RRID: AB_2927490
PERK (Mouse mAb)	Santa Cruz Biotechnology	Cat. # sc-377400; RRID: AB_2762850
p-PERK (Mouse mAb)	Santa Cruz Biotechnology	Cat. # sc-32577; RRID: AB_2293243
ERp72 (Mouse mAb)	Santa Cruz Biotechnology	Cat. # sc-135901; RRID: AB_2160986
β-actin (Mouse mAb)	Santa Cruz Biotechnology	Cat. # sc-47778; RRID: AB_626632
SEC61β (Mouse mAb)	Santa Cruz Biotechnology	Cat. # sc-393633; RRID: AB_2885065
GM130 (Mouse mAb)	Santa Cruz Biotechnology	Cat. # sc-55591; RRID: AB_1124984
HRP-conjugated anti-rabbit IgG	Santa Cruz Biotechnology	Cat. # sc-2004; RRID: AB_631746
HRP-conjugated anti-mouse IgG	Santa Cruz Biotechnology	Cat. # sc-2005; RRID: AB_631736
p-IRE1α (Rabbit pAb)	Abcam	Cat. # ab48187; RRID: AB_873899
ATF6 (Rabbit pAb)	Abcam	Cat. # ab37149; RRID: AB_725571
Ki-67 (Rabbit pAb)	Abcam	Cat. # ab15580; RRID: AB_443209
LC3B (Rabbit pAb)	Novus Biologicals	Cat. # NB100-2220; RRID: AB_10003146
BAP18 (Rabbit pAb)	Novus Biologicals	Cat. # NBP1-82670; RRID: AB_11039210
TMED3 (Rabbit pAb)	Novus Biologicals	Cat. # NBP2-94321; RRID: AB_3464248
Goat anti-mouse Alexa Fluor 488	Invitrogen	Cat. # A28175; RRID: AB_2536161
Goat anti-rabbit Alexa Fluor 488	Invitrogen	Cat. # A27034; RRID: AB_2536097
Goat anti-mouse Alexa Fluor 594	Invitrogen	Cat. # A21044; RRID: AB_2535713
Goat anti-rabbit Alexa Fluor 594	Invitrogen	Cat. # A32740; RRID: AB_2762824
HRP-conjugated secondary antibodies (for WB)	Proteintech	Cat. # SA00001
**Oligonucleotides and other sequence-based reagents**		
siRNA targeting *TMED3* (sense: GCUGAAGUCAAGGGCGUUUTT)	GenePharma (Shanghai, China)	Custom synthesis Appendix Fig. S10
siRNA targeting *ATG7* (sense: CAGUGGAUCUAAAUCUCAAACUGAU)	GenePharma	Custom synthesis Fig. 4 and Appendix Figs. S6, S8, EV4
siRNA targeting *IRE1α* (sense: GGACGUGAGCGACAGAAUA)	GenePharma	Custom synthesis Fig. EV1 and Appendix Fig. S6
siRNA targeting *BAP18* (sense: GGGACGAUCUUAAUCACAUTT)	GenePharma	Custom synthesis Appendix Fig. S10
Scramble siRNA	GenePharma	Custom synthesis
sgRNA sequences for TMED3-KO (three independent guides)	This study (cloned into pLV3-U6)	knockout validation in Fig. EV2; phenotypic validation in Fig. 6
**Chemicals, Enzymes and other reagents**		
Chrysosplenetin (CHR)	Target Molecule (Boston, MA, USA)	Cat. # TJS1159
Chloroquine (CQ)	Selleck Chemicals	Cat. # S6999
Gemcitabine (GEM)	Selleck Chemicals	Cat. # S1714
Z-VAD-FMK (pan-caspase inhibitor)	Selleck Chemicals	Cat. # S7023
4-Phenylbutyrate (4-PBA)	Selleck Chemicals	Cat. # S4125
Wortmannin (Wort)	Selleck Chemicals	Cat. # S2758
Bafilomycin A1 (Baf-A1)	Selleck Chemicals	Cat. # S1413
4μ8C (IRE1α inhibitor)	Selleck Chemicals	Cat. # S7272
GSK2606414 (PERK inhibitor)	Selleck Chemicals	Cat. # S7307
Cycloheximide (CHX)	Selleck Chemicals	Cat. # S7418
MG132 (proteasome inhibitor)	Selleck Chemicals	Cat. # S2619
DMEM (high glucose)	Gibco	Cat. # 11965092
Fetal bovine serum (FBS)	VivaCell Biosciences	Cat. # C04001
Penicillin/streptomycin	Gibco	Cat. # 15140122
Trypsin-EDTA	Gibco	Cat. # 25200056
TrypLE (for organoid dissociation)	Gibco	Cat. # 12604013
Lipofectamine 3000	Invitrogen	Cat. # L3000008
Puromycin	Beyotime Biotechnology	Cat. # ST551
Hygromycin B	Beyotime Biotechnology	Cat. # ST1389
Polybrene	Beyotime Biotechnology	Cat. # C0351
Cell Counting Kit-8 (CCK-8)	GLPBIO	Cat. # GK10001
Annexin V-FITC / PI apoptosis detection kit	Beyotime Biotechnology	Cat. # C1062
RIPA lysis buffer	Beyotime Biotechnology	Cat. # P0013
Protease & phosphatase inhibitor cocktail	Beyotime Biotechnology	Cat. # P1045
BCA protein assay kit	Beyotime Biotechnology	Cat. # P0011
SDS-PAGE loading buffer (5×)	ABclonal	Cat. # RM00001
PVDF membrane	ABclonal	Cat. # RM00018
ECL chemiluminescence substrate	ABclonal	Cat. # RM00021P
4% Paraformaldehyde	Beyotime Biotechnology	N/A
Triton X-100	Beyotime Biotechnology	Cat. # P0096
DAPI	Beyotime Biotechnology	Cat. # P0131
Crystal violet	Beyotime Biotechnology	Cat. # Y268091
Collagenase XI	Sigma-Aldrich	N/A
DNase I	Sigma-Aldrich	N/A
Y-27632 (ROCK inhibitor)	Selleck Chemicals	N/A
R-spondin1 conditioned medium	This study	Figs. 1B,C and 7A,B)
Wnt3A-conditioned medium	This study	Figs. 1B,C and 7A,B)
Recombinant human TMED3 (WT and mutants for BLI)	OriGene China	Cat. # TP760290 (WT), TP810497 (R20A), TP810498 (E123A), TP810499 (P128A)
Biotin Protein Conjugation Kit	Beyotime Biotechnology	Cat. # P0632
Pronase (for DARTS)	Sigma-Aldrich	Cat. # 10165921001
Super Streptavidin (SSA) biosensors	Sartorius	Cat. # 18-5056
**Software**		
GraphPad Prism (v8.0.2)	https://www.graphpad.com	
ImageJ (v1.53)	https://imagej.nih.gov/ij/	
SynergyFinder (v3.0)	https://synergyfinder.fimm.fi/	
R (v4.3.0)	https://www.r-project.org/	
DESeq2 (v1.42.0)	https://bioconductor.org/packages/DESeq2/	
clusterProfiler (v4.0)	https://bioconductor.org/packages/clusterProfiler/	
ComplexHeatmap (v2.20.0)	https://bioconductor.org/packages/ComplexHeatmap/	
DIA-NN (v1.8) for proteomics	https://www.dia-nn.org/	
AutoDock Vina (v1.2) for docking	https://vina.scripps.edu/	
AMBER (v20) for MD simulations	https://ambermd.org/	
Octet software (v6.4) for BLI	included with instrument	
**Other**		
NanoDrop One spectrophotometer	Thermo Fisher Scientific	N/A
timsTOF Pro mass spectrometer	Bruker Daltonics	N/A
Cytoflex LX flow cytometer	Beckman Coulter	N/A
Leica TCS SP8 confocal microscope	Leica	N/A
Akoya Vectra Polaris slide scanner	Akoya Biosciences	N/A

### Cell culture

Human pancreatic cancer cell lines T3M4 (RRID:CVCL_4056), Miapaca-2 (RRID:CVCL_0428), PANC-1 (RRID:CVCL_0480), SW1990 (RRID:CVCL_1723) and SUIT-2 (RRID:CVCL_3172) were obtained from the American Type Culture Collection (ATCC). All cell lines were cultured in high-glucose Dulbecco’s modified Eagle’s medium (DMEM; Gibco, 11965092) supplemented with 10% fetal bovine serum (FBS; VivaCell Biosciences, C04001) and 1% penicillin/streptomycin (Gibco, 15140122) and grown in a humidified chamber at 37 °C, 5% CO_2_. Cells were trypsinized using trypsin (Gibco, 25200056) and all cell lines were passaged for a maximum of 20 passages. All cell lines were recently authenticated via short tandem repeat (STR) profiling for identity verification and cross-contamination exclusion. Routine mycoplasma testing was performed during cell culture, and all cell lines were confirmed to be mycoplasma-free throughout the study.

### Reagents and antibodies

The drug library containing 411 natural products was customized by Target Molecule (Boston, MA, USA). Chrysosplenetin (CHR) was purchased from Target Molecule (Boston, MA, USA, TJS1159). chloroquine (CQ, S6999), gemcitabine (Gem, S1714), benzyloxycarbonyl-Val-Ala-Asp-fluoromethyl ketone (Z-VAD, S7023), 4-phenylbutyrate (4-PBA, S4125), wortmannin (Wort, S2758), bafilomycin A1 (Baf-A1, S1413), 4μ8C (S7272), GSK2606414 (S7307), cycloheximide (CHX, S7418) were purchased from Selleck.

Antibodies used in this study: cleaved caspase3 (#9661, RRID:AB_2341188), cleaved PARP (#9541, RRID:AB_331426), LC3B (#83506, Host: Mouse, RRID:AB_2800018), ATG7 (#8558S, RRID:AB_10831194), XBP1s (#40435, RRID: AB_2891025) were purchased from Cell Signaling Technology. CHOP (sc-56107, RRID:AB_783507), BIP (sc-376768, RRID:AB_2819145), IRE1α (sc-390960, RRID:AB_2927490), PERK (sc-377400, RRID:AB_2762850), p-PERK (sc-32577, RRID:AB_2293243), ERp72 (sc-135901, AB_2160986), β-actin (sc-47778, RRID:AB_626632), SEC61β (sc-393633, RRID: AB_2885065), GM130 (sc-55591, RRID: AB_1124984), horseradish peroxidase-conjugated anti-rabbit secondary antibody (sc-2004, RRID:AB_631746), and horseradish peroxidase-conjugated anti-mouse secondary antibody (sc-2005, RRID:AB_631736) were purchased from Santa Cruz Biotechnology. p-IRE1α (ab48187, RRID: AB_873899), ATF6 (ab37149, RRID: AB_725571) and Ki-67 (ab15580, RRID:AB_443209) were purchased from Abcam. LC3B (NB100-2220, Host: Rabbit, RRID:AB_10003146), BAP18 (NBP1-82670, RRID: AB_11039210) and TMED3 (NBP2-94321, RRID:AB_3464248) was purchased from Novus. Goat anti-mouse Alexa Fluor 488 (A28175, RRID:AB_2536161), goat anti-rabbit Alexa Fluor 488 (A27034, RRID:AB_2536097), goat anti-mouse Alexa Fluor 594 (A21044, RRID:AB_2535713) and goat anti-rabbit Alexa Fluor 594 (A32740, RRID:AB_2762824) were obtained from Invitrogen.

Primary antibodies were diluted 1:1000 for immunoblotting, 1:200 for immunofluorescence, and 1:100 for immunohistochemistry. Secondary antibodies were applied at 1:5000 for immunoblotting and 1:200 for immunofluorescence.

### Patients’ derived organoid culture and drug respohelsinkinse assay

Primary culture information: The PDOs used in this study were primary cultures originating from human (Homo sapiens). A total of three independent PDO lines derived from three different individual patients all of which were male were utilized. No genetic modification was performed on all primary organoid cultures in this study. The collection and use of all patient-derived tumor samples in this study were conducted in strict accordance with ethical standards and were approved by the appropriate institutional review board. The study protocol involving human samples (PDO, PDX and tissue microarray cohort) was reviewed and approved by the Clinical Research Ethics Committee of the First Affiliated Hospital, Zhejiang University School of Medicine (Approval Number: [2025B] IIT Ethics Approval No. 1019). All participants provided informed consent before sample collection. All human-related procedures complied with the principles of the WMA Declaration of Helsinki and the Department of Health and Human Services Belmont Report, and were approved in accordance with the ethical regulations for human biomedical research in China. The establishment and culture of patients’ derived organoid lines was performed in accordance with previous studies (Boj et al, 2015; Murthy et al, 2024). In short, the surgical resection specimens were stored in tissue preservation solution, and then were first minced and then incubated in a digestion solution. This solution contained 1 mg/mL of collagenase XI, 10 µg/mL of DNase I, and 10.5 µM/L of Y-27632, all dissolved in DMEM/F12 medium. The incubation took place at 37 °C with gentle agitation for a duration of up to 30 min. Following this, the cells that were released from the tissue were collected. These cells were then placed on a Matrigel substrate and cultivated in a specific growth medium designed for human cells. This medium was composed of advanced DMEM/F12, HEPES at a concentration of 10 mM/L, Glutamax at 1×, A83-01 at 500 nM/L, hEGF at 50 ng/mL, Noggin at 100 ng/ml, hFGF10 at 100 ng/mL, Gastrin I at 0.01 µM/L, N-acetylcysteine at 1.25 mM/L, nicotinamide at 10 mM/L, PGE2 at 1 µM/L, B27 supplement at 1× final concentration, R-spondin1 conditioned medium at 10% final concentration, and Wnt3A-conditioned medium at 50% final concentration. Several days later, the growth status of the organoids was assessed under a microscope.

For the organoid drug response assay, the organoids were digested into single cells using trypLE (Gibco, 12604013), seeded on average into 48-well plates, and cultured using the above method. PDOs were cultured until reaching approximately 50 μm in diameter, and then treated with indicated concentrations of agents diluted in fresh complete organoid medium. The medium was changed every 3 days and photographed under a microscope. The size (diameter) of organoids was statistically analyzed using Image J software.

### Cell viability assay and synergy analysis

Cell viability was detected by using Cell Counting Kit-8 (CCK-8; GLPBIO, GK10001) assay according to the manufacturer’s guidance. Briefly, 4000 cells per well were seeded in 96-well plates. After 24 h, the medium was aspirated and then the cells were treated with the indicated concentrations of chemical agents for 48 h in complete medium. At the end of the treatment, the medium containing chemical agents was aspirated, and 10% v/v of a solution of CCK-8 agent was then added for about 1 h. Relative cytotoxicity was determined by measuring the absorbance at 450 nm using a microplate reader (BioTek Instruments). For synergy analysis, zero interaction potency (ZIP) model was employed to calculate synergy index according to the template on https://synergyfinder.fimm.fi/.

### Clonogenicity assay

T3M4 and Miapaca-2 cells were seeded at 1000 and 1500 cells per well, respectively, in 12-well plates and allowed to grow for 3 days, then replaced with culture medium containing the indicated concentration of chemical agents. The culture medium was changed every 3 days. After 1 week, the culture medium containing the agents was aspirated, and the cells were carefully rinsed with PBS, fixed in methanol for 30 min and stained with crystal violet (Beyotime Biotech, Y268091) solution (0.5% w/v) for 30 min at room temperature. Finally, the plates were washed with water and dry. Then clones were diluted with 0.1% SDS, the absorbance was measured at 570 nm.

### EdU incorporation assay

The 5-ethynyl-20-deoxyuridine (EdU) incorporation assay was used to detect cells in the proliferative phase, using the EdU Cell Proliferation Kit with AF488 (Beyotime Biotech, C0071) according to the manufacturer’s instructions. To summarize, 5 × 10^4^ cells were seeded in each well of 12-well plates. After 24 h, the original culture medium was replaced with a medium containing the indicated concentration of the chemical agents and treated for 48 h. Following the treatment, the cells were labeled with a final concentration of 10 μM EdU work solution at 37 °C for another 2 h. After fixation with 4% paraformaldehyde and permeabilization with 0.2% Triton X-100 (Beyotime Biotech, P0096), the cells were incubated at room temperature (RT) in the dark with click reaction solution for 30 min, followed by staining of the cell nucleus with Hoechst 33342 for 10 min. Finally, immediately and randomly capture images of the cells per well using a fluorescence microscope. The proportion of EdU-incorporative cells was statistically analyzed using Image J software.

### Flow cytometry

Measure cell apoptosis rate using Annexin V-FITC/propidium iodide (PI) detection kit (Beyotime Biotech, C1062). According to the manufacturer’s instructions, in short, the treated cells were washed with PBS and then resuspended in 100 μL binding buffer and incubated with 5 μL Annexin V-FITC for 10 min and 10 μL PI immediately in the dark. Collect at least 2 × 10^4^ live gingle cells on a flow cytometer (Cytoflex LX).

### Protein extraction and immunoblot analysis

Cells in culture were washed with ice-cold PBS twice to completely remove residual medium. RIPA lysis buffer (Beyotime Biotech, P0013) supplemented with protease inhibitor and phosphatase inhibitor (Beyotime Biotech, P1045) was directly added to cell layers and scraped on ice. Cell lysates were transferred to small tubes and lysed on ice for 15 min with occasional vortex before being cleared by centrifugation (20,000 × *g*) at 4 °C. Protein concentrations in lysates were measured by BCA protein quantification assay (Beyotime Biotech, P0011) and adjusted equally between samples, followed by the addition of SDS-PAGE loading buffer (5×) (ABclonal, RM00001) and boiling at 100 °C for 5 min. Equal volume and equal quantity of protein samples were subjected to SDS-PAGE and transferred to a PVDF membrane (ABclonal, RM00018). The membrane was blocked in 5% milk at room temperature for 1 h and incubated with appropriate antibodies at 4 °C overnight. On the next day, the membrane was washed with TBST buffer three times and incubated with appropriate secondary HRP antibodies (Proteintech, SA00001) (diluted in 1:5000) in 5% milk at room temperature for 1 h. The membrane was washed again with TBST buffer three times and ECL (ABclonal, RM00021P) was applied for film development.

### Immunofluorescence and confocal microscopy

For immunofluorescence imaging of treated cells, after washing twice with PBS, cells were fixed with 4% paraformaldehyde for 30 min, washed three times with PBS before permeabilization with 0.5% Triton X-100 for 10 min, followed by three washes with PBS. After blocking with 5% BSA (BioFroxx, 4240GR100) for 30 min at RT, cells were washed twice with PBS and then incubated with primary antibodies diluted in PBS containing 3% BSA overnight at 4 °C. On the following day, after washes in PBS buffer three times, cells were incubated with Alexa Flour secondary antibodies diluted in PBS containing 3% BSA (1:500) at RT for 1 h. Cells were then rinsed in TBST three times and stained with DAPI (Beyotime Biotech, P0131) for 10 min at RT. Images were captured by a confocal laser scanning microscopy (Leica TCS SP8). The puncta and colocalization were analyzed using Image J software.

### RNA interference and plasmid transfection

For RNA interference mediated knockdown, the scrambled, TMED3 and ATG7 siRNAs were synthesized by GenePharma (Shanghai, China) and resuspended according to the manufacturer’s instructions.

siRNA sequences are listed as follows:

si*TMED3*: 5’-GCUGAAGUCAAGGGCGUUUTT-3’;

si*ATG7*: 5’-CAGUGGAUCUAAAUCUCAAACUGAU-3’;

si*IRE1α*: 5’-GGACGUGAGCGACAGAAUA-3’;

si*BAP18*: 5’-GGGACGAUCUUAAUCACAUTT-3’.

The cells were transfected at approximately 50% confluency using Lipofectamine 3000 reagent (Invitrogen, L3000008) according to the instructions. The siRNA knockdown efficiency was detected by western blot after 48 h, and subsequent experiments were conducted after 48 h as well. For autophagic flux detection, the tandem mRFP-GFP-LC3 reporters were transiently transfected into cells using Lipofectamine 3000 followed by chemical agents treatment 48 h later. After treatment, the cells were fixed with 4% paraformaldehyde and stained with DAPI before observation under confocal microscopy.

### The establishment of stable cell lines

For CRISPR-Cas9-mediated gene knockout, the plasmid carrying the gRNA targeting human *TMED3* was purchased from MIAOLING PLASMID. Plasmids are extracted and purified according to the manufacturer’s (Omega Bio-Tek, Endo-Free Plasmid DNA Maxi Kit) instructions. Lentivirus carrying pLV3-U6-puromycin^+^ plasmid was produced by co-transfecting HEK293T cells with two helper plasmids (psPAX2, pMD2.G) and by harvesting viral supernatant after 72 h by passing through a 0.45 µm filter. An appropriate amount of 5×PEG8000 was added to the virus supernatant and concentrated on a shaker at 4 °C for 24 h, followed by centrifugation at 4000 rpm for 30 min. Subsequently, discarded the supernatant and added complete medium supplemented with 10 µg/ml polybrene (Beyotime Biotech, C0351) to the precipitate. The original medium was replaced with the supplemented one and the cells were infected for 24 h and then replaced with fresh medium. Infected cells were selected and expanded with puromycin (Beyotime Biotech, ST551) at 2 µg/ml for 7 days before being used for subsequent assays. For the TMED3 rescue experiment, the pLV3-CMV-hygromycin^+^ plasmid carrying *3×FLAG-TMED3 WT/R20A/E123A/P128A* was stably transfected into cells using the aforementioned method and selected with hygromycin (Beyotime Biotech, ST1389) at 500 µg/ml. The effects of gene knockout and overexpression were confirmed by Western Blot assays.

### The native-gel electrophoresis assay

The protein extraction basically followed the aforementioned method, but with the addition of 5× non-denaturing and non-reducing loading buffer (Beyotime Biotech, P0016N) and without heating to boiling. Then, the protein sample was subjected to immunoblot analysis using non-denaturing electrophoresis buffer without SDS (ABclonal, BR00006) and pre-cast native-gel (ABclonal, BRK0020P).

### Drug affinity responsive target stability

Cells cultured in 100-mm dishes were treated with CHR (20 μM) or DMSO control for 12 h. Cells were harvested, lysed using RIPA buffer and then divided into 4 aliquots. The protein concentrations were measured by BCA protein quantification assay. The pronase (Sigma-Aldrich, 10165921001) was added for proteolysis at RT for 1 h. The ratio of pronase: total protein was 1:200, 1:400, 1:800 and 1:1600 in each tube. Subsequently, the samples were then mixed with the loading buffer and analyzed by immunoblotting with TMED3 antibody.

### Cellular thermal shift assay

Cells cultured in 100-mm dishes to 80% confluency were treated with CHR (20 μM) or DMSO control for 12 h. Cells were harvested by trypsin, resuspended with PBS, and then divided into 6 aliquots, each of which was heated at 48, 53, 58, 63, 68, 73 °C for 5 min. Soluble fractions were then extracted by 3 cycles of freeze-thawing with liquid nitrogen, followed by centrifugation at 20,000 × *g* for 15 min in 4 °C. Finally, samples were analyzed by immunoblotting with TMED3 antibody.

### Bio-layer interferometry assay

BLI assay was performed by the ForteBio Octet Red system. Recombinant human wild‑type and mutant TMED3 proteins (R20A, E123A, P128A, His‑tagged, >80% purity) used for BLI were purchased from a commercial vendor (OriGene China, TP760290, TP810497, TP810498 and TP810499). These TMED3 proteins were first biotinylated using Biotin Protein Conjugation Kit (Beyotime Biotech, P0632) according to the instructions and then immobilized onto the Super Streptavidin (SSA) biosensors (Sartorius, 18-5056) in 200 μL loading buffer (20 mM HEPES, 150 mM NaCl, 2 mM CaCl_2_, 0.1% BSA, pH 7.5). Then, the tip of the sensors were immersed into the kinetic buffer (PBS containing 0.1% BSA, 0.02% Tween 20, and 5% DMSO) to measure the baseline signal for 10 min. Afterwards, the kinetic buffer and different concentrations of CHR were added to each well of the all-black 96-well plates. After the baseline measurement was completed, the sensors were immersed into the buffer containing CHR for a 10 min measurement. Subsequently, wash with buffer for 5 min to allow the CHR molecules to dissociate from the sensors and prepare for the next binding. Data analysis was performed with the Octet (v.6.4) software.

### Molecular docking and dynamics simulation

AutoDock Vina is a widely used molecular docking software. The crystal structure data of the TMED3 protein for docking were obtained from the PDB database, while the three-dimensional structure of CHR was retrieved from the PubChem database. Molecular energy minimization was performed using the MMFF94 force field. Subsequently, molecular docking was conducted using AutoDock Vina 1.2 software. The resulting CHR-TMED3 complex from the docking was used as the initial structure for all-atom molecular dynamics (MD) simulations, which were performed using the AMBER 20 software. The small molecule was described using the GAFF2 force field, while the protein was modeled with the ff14SB force field. Following this, the MM/GBSA method was employed to calculate the binding free energy between the TMED3 and the CHR, and alanine scanning was performed. For this study, MD trajectories of 90–100 nanoseconds were selected for the calculations, as excessively long simulation times may affect the accuracy of the MM/GBSA results.

### RNA extraction, RNA-seq and data processing

Total RNA was extracted from DMSO- or CHR-treated T3M4 and Miapaca-2 cells for 24 h using Trizol Reagent (ABclonal, RK30129) and RNA quality was determined and quantified using an spectrophotometer (Thermo Fisher Scientific, NanoDrop One). High-throughput sequencing was performed on the Illumina NovaSeq 6000 platform (Illumina, San Diego, CA, USA) using a paired-end 2 × 150 bp configuration. Reads were aligned and quantified against the GRCh38 human reference genome (GENCODE release 44 annotations) using Salmon (v1.9.0) with transcriptome-specific indices. Raw read counts were aggregated into a gene-level matrix using the tximport function from tximport R package (v1.30.0) in R (v4.3.0). Differentially expressed genes (DEGs) were identified using DESeq2 (v1.42.0) with a significance threshold of adjusted *P* value (Benjamini–Hochberg method) <0.05 and absolute log2 fold change >1. Count data were normalized via variance stabilizing transformation (VST) varianceStabilizingTransformation function for downstream analyses. Gene set enrichment analysis (GSEA) was performed using Gene Ontology (GO) terms from the MSigDB human collection (v2023.1) via the clusterProfiler package (v4.0). Heatmaps of core genes in selected pathways were generated using the ComplexHeatmap package (v2.20.0).

### Proteomics

Proteomic analysis was conducted by PTMbio (Hangzhou, China). Briefly, tryptic peptides were separated using a nanoElute UHPLC system (Bruker Daltonics) with a home-made reversed-phase column (25 cm × 100 μm i.d.) and a gradient of 6–80% acetonitrile (0.1% formic acid) over 20 min at 500 nL/min. The platform employed a Bruker timsTOF Pro system equipped with parallel accumulation-serial fragmentation (PASEF) technology, operating in dia-PASEF mode with the following parameters: MS1 range 300–1500 *m*/*z*, MS2 range 400–850 *m*/*z*, 20 PASEF scans/cycle, and a 7 *m*/*z* isolation window. Electrospray ionization was applied at 1.75 kV. For protein identification and quantification, raw data were processed through DIA-NN v1.8 using a human UniProt database (20,429 entries) with trypsin/P digestion (1 missed cleavage allowed), carbamidomethylation (C) as a fixed modification, and a 1% FDR threshold. Statistical significance was set at adjusted *P* value < 0.05, and proteins exhibiting expression variations exceeding 1.5-fold were classified as differentially expressed.

### Xenograft mouse model

Male nude mice (BALB/c nude, 7 weeks old) were purchased from GemPharmatech (Jiangsu, China) and used for the establishment of PC xenograft model. All in vivo experiments were conducted under the institutional ethical guidelines on animal care and were approved by the Institute of Animal Care and Use Committee at The First Affiliated Hospital, Zhejiang University School of Medicine (Reference Number: 2025-实动第109号). All mice were housed in specific pathogen-free (SPF) environment in cages of up to five animals and maintained under a 12 h light/12 h dark cycle with water and food provided ad libitum. For the PDX model, tumor tissues were obtained from two patients diagnosed with PC who underwent radical tumor resection in the Department of Hepatobiliary and Pancreatic Surgery, The First Affiliated Hospital, Zhejiang University School of Medicine. These tumor tissues were washed and cut into 3–4 mm pieces in ice-cold PBS containing FBS and penicillin/streptomycin, and subcutaneously transplanted into the right axilla of nude mice using hollow needles and then the skin was disinfected. For the subcutaneous tumor model, T3M4 and Miapaca-2 cells (1 × 10^7^ cells/mouse) were subcutaneously injected into the right flanks of the nude mice. Measure tumor volume every 2 days by a microcaliper and calculated by the following formula: volume (mm^3^) = ½ × length × width^2^. When the tumor volume reached ~100 mm^3^, the mice were randomly divided into different groups. In Fig. 1I and Appendix Fig. S1B,G, group 1 received 200 μL of vehicle, and group 2 received 25 mg/kg CHR. Vehicle or CHR was administered every 2 days for 12 days. In Fig. 7C, mice bearing PDAC xenografts were randomly divided into 6 groups and received the following treatments every 2 days: (i) vehicle; (ii) 25 mg/kg CHR; (iii) 25 mg/kg CQ; (iv) 25 mg/kg CHR + 25 mg/kg CQ; (v) 15 mg/kg Gem; (vi) 25 mg/kg CHR + 15 mg/kg Gem. CHR was administered via gavage, while CQ and GEM were administered intraperitoneally. The mouse weight and tumor volume were monitored every 2 days and were sacrificed 12 days post-treatment. The tumor tissues or organs were isolated for further analysis. The blood was collected for liver and kidney function analysis using the corresponding reagent kit purchased from Jiancheng Bioengineering Institute (Jiangsu, China) (ALT - C009-2-1, AST - C010-2-1, LDH - A020-2-2, ALP - A059-2-2, CREA - C011-2-1, T-Bil - C019-1-1). No blinding was performed during animal treatment, sample collection, and image quantification analysis, due to the distinguishable administration routes and treatment regimens in different experimental groups. Inclusion and exclusion criteria: Pre-established inclusion criteria included healthy mice with normal feeding and behavioral status and successful tumor engraftment. Pre-defined exclusion criteria included severe weight loss, deteriorated mental status, tumor ulceration, or unexpected death. No animals or data points were excluded or lost to attrition during the experiment; all collected data were included in the final analysis.

### H&E staining and immunohistochemistry

Tumor or organ tissues were collected and fixed in 4% paraformaldehyde and embedded in paraffin. Paraffin sections (4 µm) were cut using a microtome (Leica HistoCore MULTICUT) and stained with H&E or processed for immunohistochemistry following standard protocols. For the clinical pancreatic cancer tissue microarray cohort, commercially prepared and pre-constructed formalin-fixed, paraffin-embedded tissue microarray slides were used for immunohistochemistry staining. All participants provided informed consent before sample collection. All human-related procedures complied with the principles of the WMA Declaration of Helsinki and the Department of Health and Human Services Belmont Report, and were approved in accordance with the ethical regulations for human biomedical research in China. All participant information (including sex and age) of the tissue microarray cohort was listed as follows. High expression: #1 female 67, #2 male 62, #3 male 77, #4 male 62, #5 male 53, #6 male 79, #7 male 65, #8 male 69, #9 male 62, #10 female 62, #11 female 61, #12 male 66, #13 female 57, #14 female 53, #15 male 66, #16 male 62, #17 male 45, #18 female 63, #19 female 64, #20 male 64, #21 male 77, #22 female 60; Low expression: #1 male 64, #2 male 65, #3 female 62, #4 female 55, #5 female 79, #6 male 68, #7 female 87, #8 male 70, #9 male 90, #10 male 75, #11 female 71, #12 male 60, #13 female 74, #14 male 65, #15 female 64, #16 female 45, #17 female 57, #18 female 66, #19 female 60. For immunohistochemistry staining, after dewaxing and inactivation of endogenous peroxidases (3% hydrogen peroxide in PBS), antibody-specific heat-mediated antigen retrieval was performed using the electric cooker (boil for 10 min and then keep it warm for 30 min). After cooling at RT, sections were blocked with PBS containing 3% BSA at RT for 30 min and then incubated with primary antibodies diluted in PBS containing 3% BSA overnight at 4 °C in a wet box. The next day, sections were washed three times with TBST buffer and stained with HRP-labeled secondary antibody (1:200) at RT for 1 h. Finally, sections were stained using diaminobenzidine (MXB Biotech, DAB-0031) and counterstained with Mayer’s hematoxylin (MXB Biotech, CTS-1096). Slides were scanned using a scanner (Akoya Vectra Polaris) and representative images were taken. The quantitative score (range < 300) was calculated as A × B, where A was the percentage of stain-positive cell area and B denoted immunostaining intensity (0 = negative, 1 = weak, 2 = moderate, 3 = strong).

### Statistical analysis

Statistical analysis was performed with GraphPad Prism v8.0.2 (La Jolla, CA, USA; https://www.graphpad.com). Statistical significance was determined using Student’s t-test (two groups) or one/two-way ANOVA (multiple groups). Prior to parametric analysis, the Shapiro–Wilk test and Levene’s test were applied to confirm normal distribution and homogeneity of variance. All analyzed data met the assumptions of parametric tests with comparable variance across groups. Data are presented as mean ± standard deviation (SD) or standard error of the mean (SEM) as appropriate, indicating intra-group variation.

### Graphics

Some of the graphics, including Fig. 5A and synopsis, were created with BioRender.com.

All animal experiments were performed in compliance with the ARRIVE guidelines. All human sample-related studies were conducted in accordance with the ICMJE recommendations and relevant ethical regulations for biomedical research.

Randomization statement: In this study, randomization was applied during cell and organoid seeding, drug treatment assignment, and in vivo model grouping. All experimental samples and animal subjects were randomly allocated to control and experimental groups to eliminate selection bias and ensure unbiased data acquisition.

## Supplementary information

Appendix

Peer Review File

Dataset EV1

Source data Fig. 1

Source data Fig. 2

Source data Fig. 3

Source data Fig. 4

Source data Fig. 5

Source data Fig. 6

Source data Fig. 7

Figure EV1 Source Data

Figure EV2 Source Data

Figure EV3 Source Data

Figure EV4 Source Data

Figure EV5 Source Data

Appendix Figure Source Data

Expanded View Figures

## Data Availability

All omics data generated in this study were deposited in databases hosted by the National Genomics Data Center (NGDC, CNCB; https://ngdc.cncb.ac.cn/). RNA-seq data were available under accession HRA019436 (GSA-Human; https://ngdc.cncb.ac.cn/gsa-human/browse/HRA019436), and proteomic data under OMIX018324 (OMIX; https://ngdc.cncb.ac.cn/omix/release/OMIX018324). The source data of this paper are collected in the following database record: biostudies:S-SCDT-10_1038-S44321-026-00506-5.
