# Supplementary material for: Chrysosplenetin-induced TMED3 aggregation triggers unfolded protein response in pancreatic cancer
Source: EMBO Mol Med. 2026 Aug 13;18(9):3790–823. doi: 10.1038/s44321-026-00506-5 (PMC13562749; doi:10.1038/s44321-026-00506-5)
Supplement: Supplementary file 1 [file 44321_2026_506_MOESM17_ESM.pdf]

Expanded View Figures

Figure EV1. CHR triggers ER stress in PC cells.

(**A**) Volcano plot of differentially expressed genes in Miapaca-2 cells treated with CHR (20 μM) versus DMSO. Upregulated (red) and downregulated (blue) genes are highlighted. Wald test was used to analyze statistical differences. The Benjamini–Hochberg procedure was used to adjust. *N* = 3 independent biological replicates. (**B**) Pathway enrichment analysis of upregulated and downregulated pathways in Miapaca-2 cells treated with CHR. Permutation test was used to analyze statistical differences. The Benjamini–Hochberg procedure was used to adjust. (**C**) Gene Set Enrichment Analysis of the ER UPR pathway in Miapaca-2 cells treated with CHR versus DMSO. (**D**) Heatmap of ER UPR-associated gene expression in Miapaca-2 cells treated with CHR versus DMSO. (**E**, **F**) Immunoblotting analysis of ATF6 (**E**) and XBP1s (**F**) in T3M4 and Miapaca-2 cells treated with indicated concentrations of CHR for 48 h. (**G**, **H**) Immunoblotting analysis of ATF6 (**G**) and XBP1s (**H**) in T3M4 and Miapaca-2 cells treated with CHR (10 μM) in the presence or absence of 4-PBA (2 mM) for 48 h. (**I**) Immunoblotting analysis of XBP1s in T3M4 and Miapaca-2 cells treated with CHR in the presence or absence of 4μ8C (5 μM) for 48 h. (**J**) Immunoblotting analysis of IRE1α and XBP1s in T3M4 and Miapaca-2 cells treated with CHR for 48 h after transfected with si*IRE1α* or si*Scramble*. (**K**) Immunoblotting analysis of PERK and CHOP in T3M4 and Miapaca-2 cells treated with CHR in the presence or absence of GSK2606414 (5 μM) for 48 h. (**L–O**) Representative images (**L**, **N**) and quantification (**M**, **O**) of immunohistochemical staining for p-IRE1α of T3M4 and Miapaca-2 xenografts treated with vehicle or CHR, scale bar: 20 μm. Student's t test was used to analyze statistical differences in (**M**, **O**). *N* = 5 independent mice. Source data are available online for this figure.

 © The Author(s)

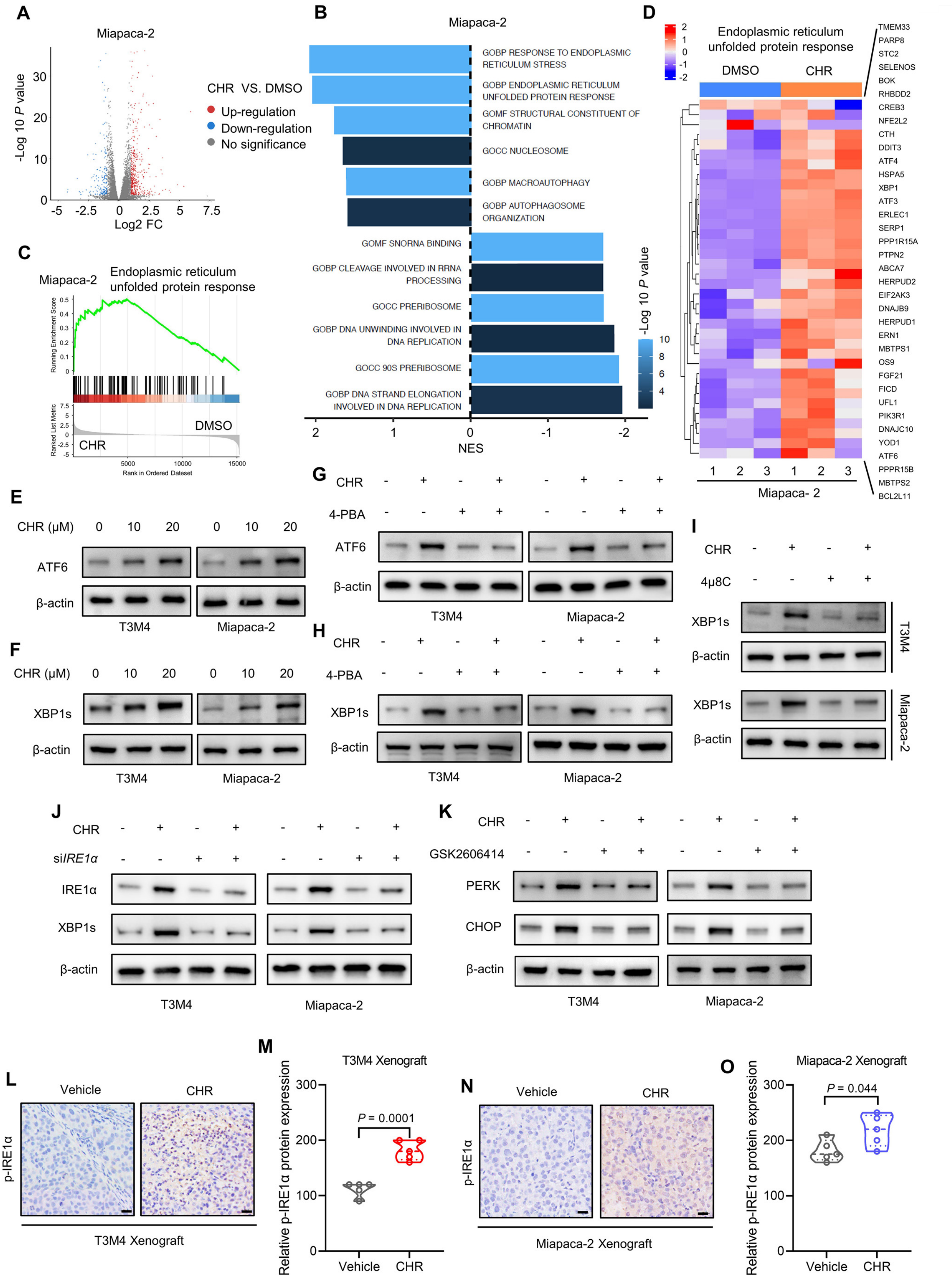
A
Miapaca-2
CHR VS. DMSO
Up-regulation
Down-regulation
No significance
-Log 10 P value
Log2 FC
B
Miapaca-2
GOBP RESPONSE TO ENDOPLASMIC RETICULUM STRESS
GOBP ENDOPLASMIC RETICULUM UNFOLDED PROTEIN RESPONSE
GOMF STRUCTURAL CONSTITUENT OF CHROMATIN
GOCC NUCLEOSOME
GOBP MACROAUTOPHAGY
GOBP AUTOPHAGOSOME ORGANIZATION
GOMF SNORNA BINDING
GOBP CLEAVAGE INVOLVED IN RRNA PROCESSING
GOCC PRERIBOSOME
GOBP DNA UNWINDING INVOLVED IN DNA REPLICATION
GOCC 90S PRERIBOSOME
GOBP DNA STRAND ELONGATION INVOLVED IN DNA REPLICATION
NES
-Log 10 P value
C
Miapaca-2
Endoplasmic reticulum unfolded protein response
Running Enrichment Score
Ranked List Metric
Rank in Ordered Dataset
DMSO
CHR
D
Endoplasmic reticulum unfolded protein response
DMSO
CHR
Miapaca- 2
TMEM33
PARP8
STC2
SELENOS
BOK
RHBDD2
CREB3
NFE2L2
CTH
DDIT3
ATF4
HSPA5
XBP1
ATF3
ERLEC1
SERP1
PPP1R15A
PTPN2
ABCA7
HERPUD2
EIF2AK3
DNAJB9
HERPUD1
ERN1
MBTPS1
OS9
FGF21
FICD
UFL1
PIK3R1
DNAJC10
YOD1
ATF6
PPPR15B
MBTPS2
BCL2L11
E
CHR (μM) 0 10 20 0 10 20
ATF6
β-actin
T3M4 Miapaca-2
F
CHR (μM) 0 10 20 0 10 20
XBP1s
β-actin
T3M4 Miapaca-2
G
CHR
4-PBA
ATF6
β-actin
T3M4 Miapaca-2
H
CHR
4-PBA
XBP1s
β-actin
T3M4 Miapaca-2
I
CHR
4μ8C
XBP1s
β-actin
T3M4
Miapaca-2
J
CHR
siIRE1α
IRE1α
XBP1s
β-actin
T3M4 Miapaca-2
K
CHR
GSK2606414
PERK
CHOP
β-actin
T3M4 Miapaca-2
L
Vehicle CHR
p-IRE1α
T3M4 Xenograft
M
T3M4 Xenograft
P = 0.0001
Relative p-IRE1α protein expression
Vehicle CHR
N
Vehicle CHR
p-IRE1α
Miapaca-2 Xenograft
O
Miapaca-2 Xenograft
P = 0.044
Relative p-IRE1α protein expression
Vehicle CHR

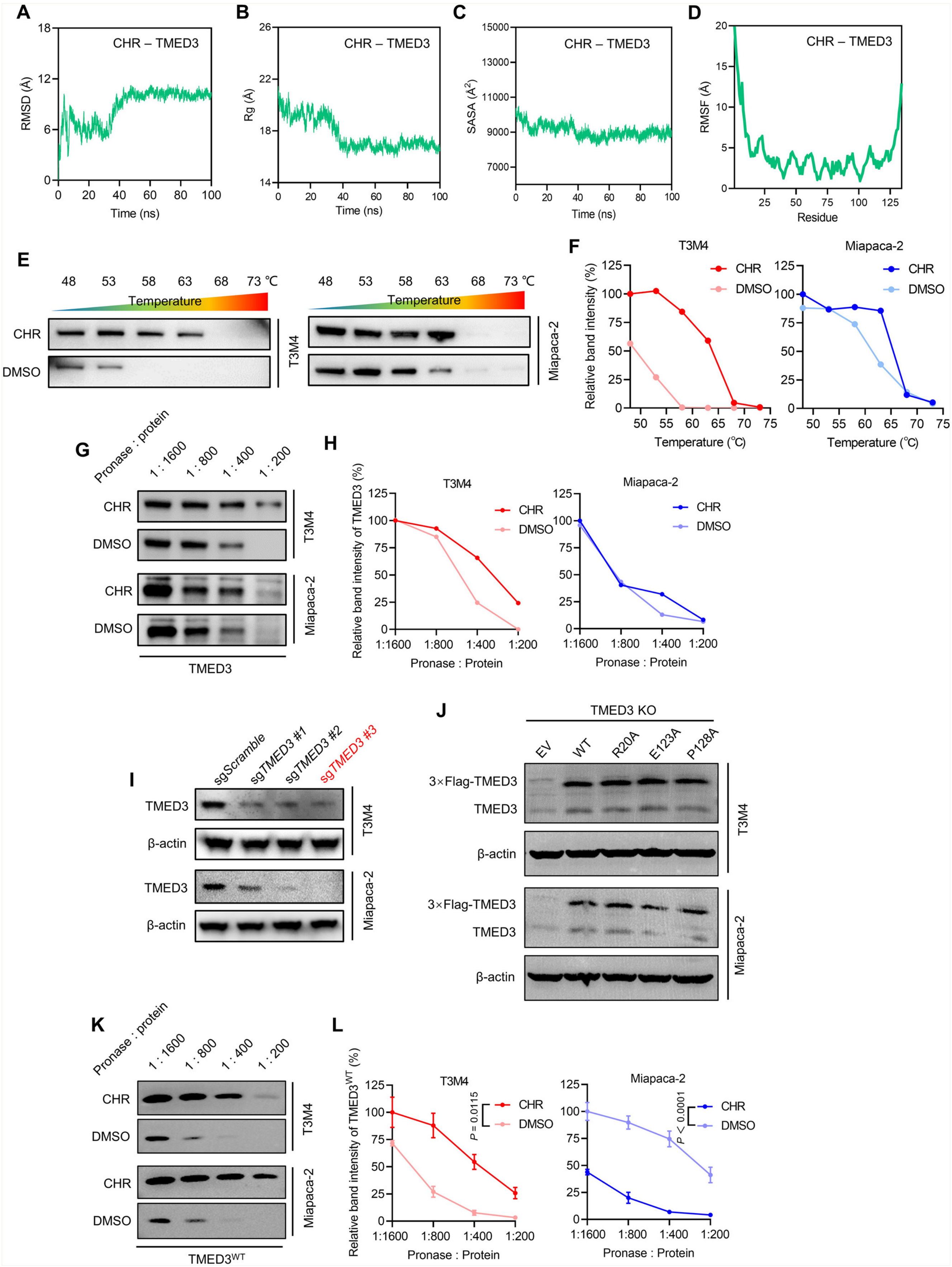
A
CHR – TMED3
RMSD (Å)
Time (ns)
B
CHR – TMED3
Rg (Å)
Time (ns)
C
CHR – TMED3
SASA (Å²)
Time (ns)
D
CHR – TMED3
RMSF (Å)
Residue
E
48 53 58 63 68 73 ℃
Temperature
CHR
DMSO
T3M4
Miapaca-2
F
T3M4
Miapaca-2
CHR
DMSO
Relative band intensity (%)
Temperature (℃)
G
Pronase : protein
1 : 1600 1 : 800 1 : 400 1 : 200
CHR
DMSO
T3M4
Miapaca-2
TMED3
H
T3M4
Miapaca-2
Relative band intensity of TMED3 (%)
Pronase : Protein
I
sgScramble
sgTMED3 #1
sgTMED3 #2
sgTMED3 #3
TMED3
β-actin
T3M4
Miapaca-2
J
TMED3 KO
EV WT R20A E123A P128A
3×Flag-TMED3
TMED3
β-actin
T3M4
Miapaca-2
K
Pronase : protein
1 : 1600 1 : 800 1 : 400 1 : 200
CHR
DMSO
T3M4
Miapaca-2
TMED3WT
L
T3M4
Miapaca-2
P = 0.0115
P < 0.0001
Relative band intensity of TMED3WT (%)
Pronase : Protein

 © The Author(s)

◀ **Figure EV2. Validation of direct interaction between CHR and TMED3.**

(**A**) Molecular dynamics simulation showing root-mean-square deviation (RMSD) of the CHR-TMED3 complex. (**B**) Radius of gyration (Rg) plot of CHR-TMED3 complex over 100 ns. (**C**) Solvent accessible surface area (SASA) of the CHR-TMED3 complex during the 100 ns simulation. (**D**) Root mean square fluctuation (RMSF) analysis of each residue in the CHR-TMED3 complex. (**E**, **F**) Immunoblotting analysis (**E**) and quantification of relative band intensity (**F**) of cellular thermal shift assay (CETSA) for CHR binding to TMED3 in T3M4 and Miapaca-2 cells. (**G**, **H**) Immunoblotting analysis (**G**) and quantification of relative band intensity (**H**) of drug affinity responsive target stability (DARTS) for CHR binding to TMED3 in T3M4 and Miapaca-2 cells. (**I**) Immunoblotting analysis verifying TMED3 knockout efficiency in T3M4 and Miapaca-2 cells generated using three independent sgRNAs targeting *TMED3*. (**J**) Immunoblotting analysis verifying TMED3 expression in TMED3-KO T3M4 and Miapaca-2 cells (generated with sgRNA#3) upon re-expression of wild-type TMED3 or TMED3 point mutants (R20A, E123A, P128A). (**K**, **L**) Immunoblotting analysis (**K**) and quantification of relative band intensity (**L**) of DARTS for CHR binding to wild-type TMED3 proteins in TMED3-KO cells. Two-way ANOVA was used to analyze statistical differences in (**L**). Mean with ± SD. $N = 3$ independent biological replicates. Source data are available online for this figure.

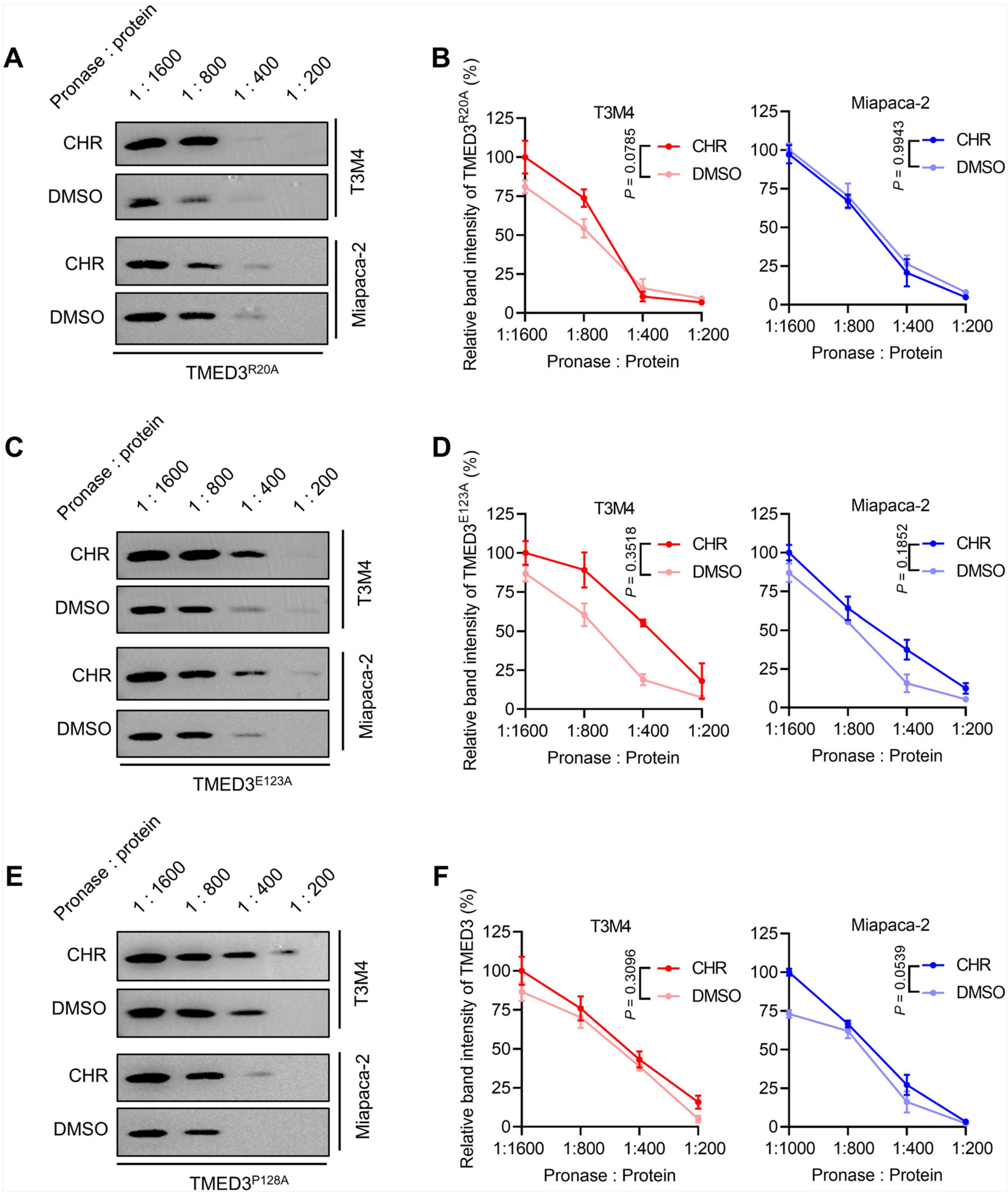

Figure EV3. Identification of key binding residues mediating direct interaction between CHR and TMED3.

(**A**, **B**) Immunoblotting analysis (**A**) and quantification of relative band intensity (**B**) of DARTS for CHR binding to TMED3 R20A mutant proteins in TMED3-KO cells. Two-way ANOVA was used to analyze statistical differences in (**B**). Mean with ± SD. $N = 3$ independent biological replicates. (**C**, **D**) Immunoblotting analysis (**C**) and quantification of relative band intensity (**D**) of DARTS for CHR binding to TMED3 E123A mutant proteins in TMED3-KO cells. Two-way ANOVA was used to analyze statistical differences in (**D**). Mean with ± SD. $N = 3$ independent biological replicates. (**E**, **F**) Immunoblotting analysis (**E**) and quantification of relative band intensity (**F**) of DARTS for CHR binding to TMED3 P128A mutant proteins in TMED3-KO cells. Two-way ANOVA was used to analyze statistical differences in (**F**). Mean with ± SD. $N = 3$ independent biological replicates. Source data are available online for this figure.

© The Author(s)

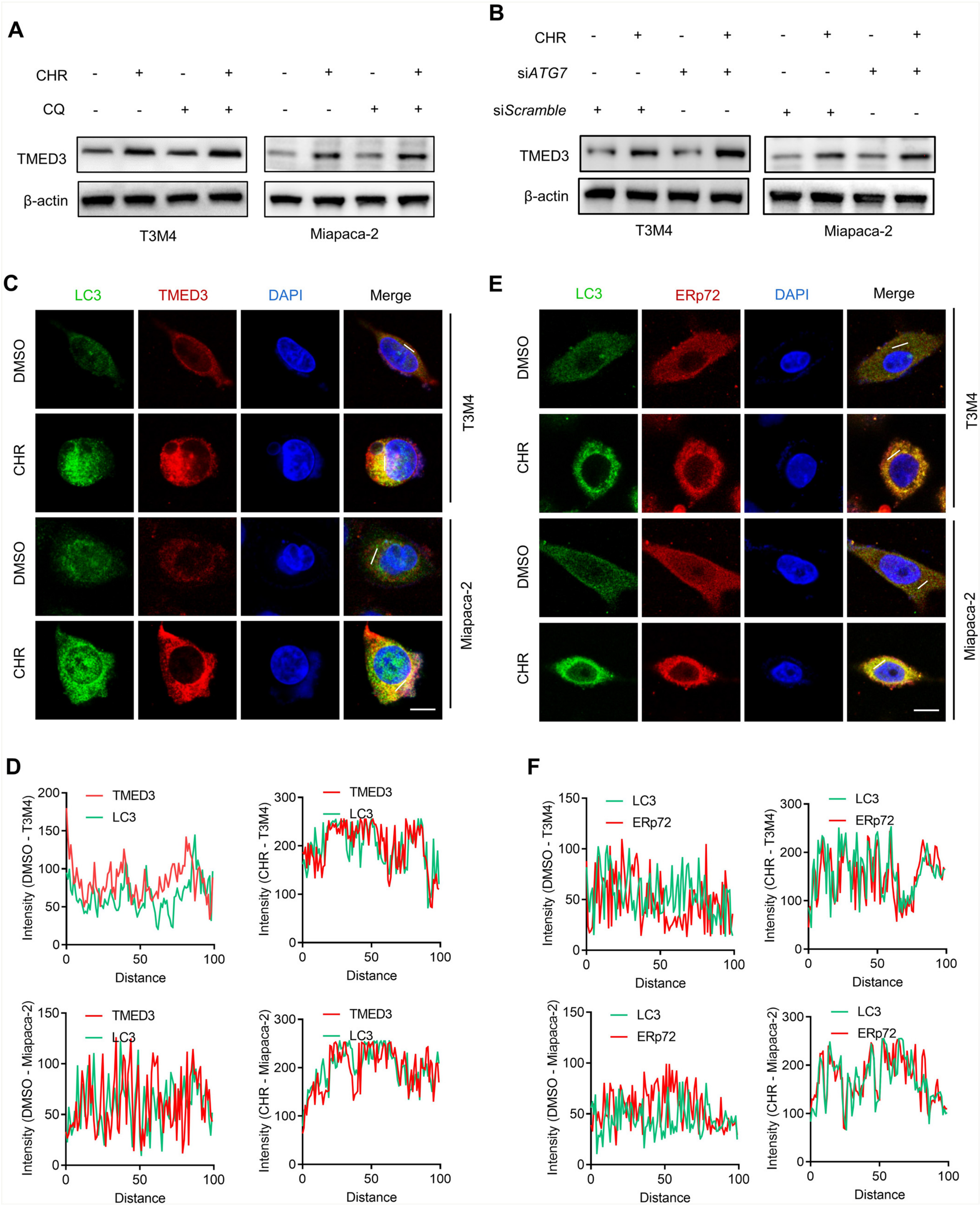
A
CHR
CQ
TMED3
β-actin
T3M4
Miapaca-2
B
CHR
siATG7
siScramble
TMED3
β-actin
T3M4
Miapaca-2
C
LC3
TMED3
DAPI
Merge
DMSO
CHR
T3M4
Miapaca-2
E
LC3
ERp72
DAPI
Merge
D
TMED3
LC3
Intensity (DMSO - T3M4)
Intensity (CHR - T3M4)
Intensity (DMSO - Miapaca-2)
Intensity (CHR - Miapaca-2)
Distance
F
LC3
ERp72

Figure EV4. CHR-mediated autophagy may be in response to TMED3 protein accumulation.

(**A**, **B**) Immunoblotting analysis of TMED3 expression in T3M4 and Miapaca-2 cells treated with CHR (10 μM) in presence or absence of CQ (10 μM) (**A**), or after transfected with si*ATG7* or si*Scramble* (B). (**C**, **D**) Representative images (**C**) and colocalization analysis of LC3 and TMED3 (**D**) of immunofluorescent staining for LC3 (green), TMED3 (red) and DAPI (blue) in T3M4 and Miapaca-2 cells treated with DMSO or CHR, scale bar: 10 μm. (**E**, **F**) Representative images (**E**) and colocalization analysis of LC3 and ERp72 (**F**) of immunofluorescent staining for LC3 (green), ERp72 (red) and DAPI (blue) in T3M4 and Miapaca-2 cells treated with DMSO or CHR, scale bar: 10 μm. Source data are available online for this figure.

 © The Author(s)

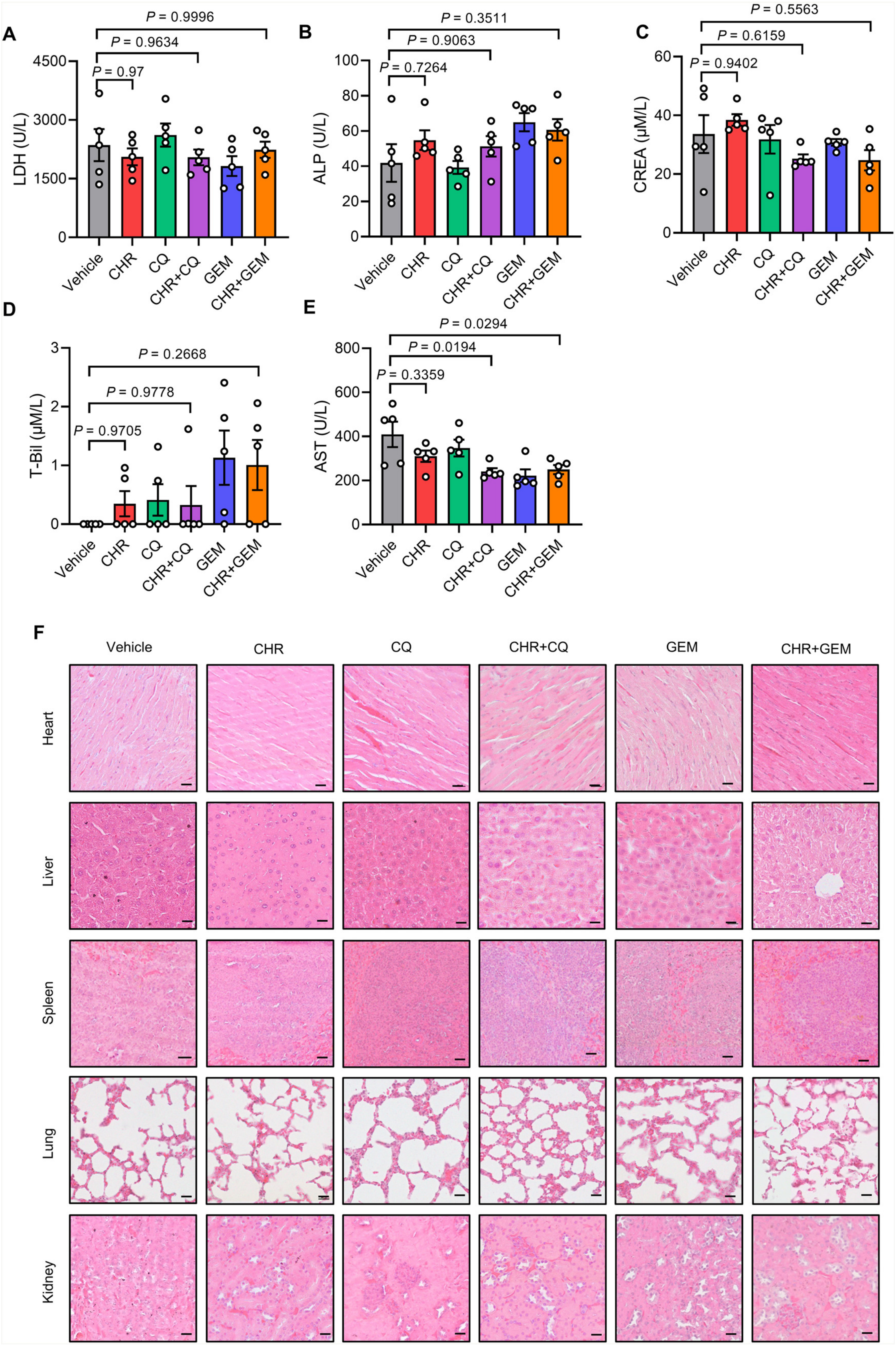
A
B
C
D
E
F
P = 0.9996
P = 0.9634
P = 0.97
LDH (U/L)
P = 0.3511
P = 0.9063
P = 0.7264
ALP (U/L)
P = 0.5563
P = 0.6159
P = 0.9402
CREA (μM/L)
P = 0.2668
P = 0.9778
P = 0.9705
T-Bil (μM/L)
P = 0.0294
P = 0.0194
P = 0.3359
AST (U/L)
Vehicle
CHR
CQ
CHR+CQ
GEM
CHR+GEM
Heart
Liver
Spleen
Lung
Kidney

Figure EV5. CHR-based combination therapy has no obvious toxic side effects.

(**A–E**) Serum levels of LDH (**A**), ALP (**B**), CREA (**C**), T-Bil (**D**) and AST (**E**) in PDX #2 xenografts treated with vehicle, CHR (25 mg/kg), CQ (25 mg/kg), CHR (25 mg/kg) + CQ (25 mg/kg), GEM (15 mg/kg), CHR (25 mg/kg) + GEM (15 mg/kg) for 12 days. One-way ANOVA was used to analyze statistical differences. Mean with ± SEM. $N = 5$ independent mice. (**F**) Representative H&E staining images of major organs (heart, liver, spleen, lung, and kidney) from mice treated with the indicated treatments, scale bars: 20 μm. Source data are available online for this figure.

 © The Author(s)
