# Supplementary material for: Chrysosplenetin-induced TMED3 aggregation triggers unfolded protein response in pancreatic cancer
Source: EMBO Mol Med. 2026 Aug 13;18(9):3790–823. doi: 10.1038/s44321-026-00506-5 (PMC13562749; doi:10.1038/s44321-026-00506-5)
Supplement: Supplementary file 2 [file 44321_2026_506_MOESM1_ESM.pdf]

Appendix for:

Chrysosplenetin-induced TMED3 aggregation triggers unfolded protein response in pancreatic cancer

The appendix include: Appendix Figure S1-S16 and Figure Legends

Contents

Appendix Figure S1. Drug library screening identified and validated the anti-PC activity of CHR *in vivo* and *in vitro*. **(Page 4-5)**

Appendix Figure S2. CHR treatment shows no obvious systemic toxicity *in vivo*. **(Page 6-7)**

Appendix Figure S3. CHR induces apoptosis in PC cells. **(Page 8-9)**

Appendix Figure S4. Inhibition of ER stress reverses CHR-mediated anti-PC effects. **(Page 10)**

Appendix Figure S5. CHR triggers ER stress-autophagy-apoptosis cascade in PC cells. **(Page 11)**

Appendix Figure S6. ER stress acts upstream of autophagy in CHR-treated PC cells. **(Page 12-13)**

Appendix Figure S7. Pharmacological inhibition of autophagy enhances CHR-induced cytotoxicity in PC cells. **(Page 14-15)**

Appendix Figure S8. Inhibition of autophagy enhances CHR-induced apoptosis in PC cells. **(Page 16-17)**

Appendix Figure S9. CHR-induced proteomic alterations in cellular components and protein subcellular localization. **(Page 18)**

Appendix Figure S10. Ubiquitin independent TMED3 accumulation contributes to CHR anti PC activity. **(Page 19-20)**

Appendix Figure S11. TMED3 is upregulated in CHR-treated PC cells *in vitro* and *in vivo*. **(Page 21)**

Appendix Figure S12. CHR enhances TMED3 colocalization with endoplasmic reticulum- and Golgi-resident markers. **(Page 22-23)**

Appendix Figure S13. CHR does not directly interact with ER- or Golgi-resident proteins other than TMED3. **(Page 24)**

Appendix Figure S14. The binding affinity between CHR and TMED3 modulates CHR-induced cytotoxicity. **(Page 25)**

Appendix Figure S15. The combination therapy of CHR and GEM synergistically kills PC cells. **(Page 26)**

Appendix Figure S16. CHR-related phenotypic pathways are observed in the PDX model. **(Page 27)**

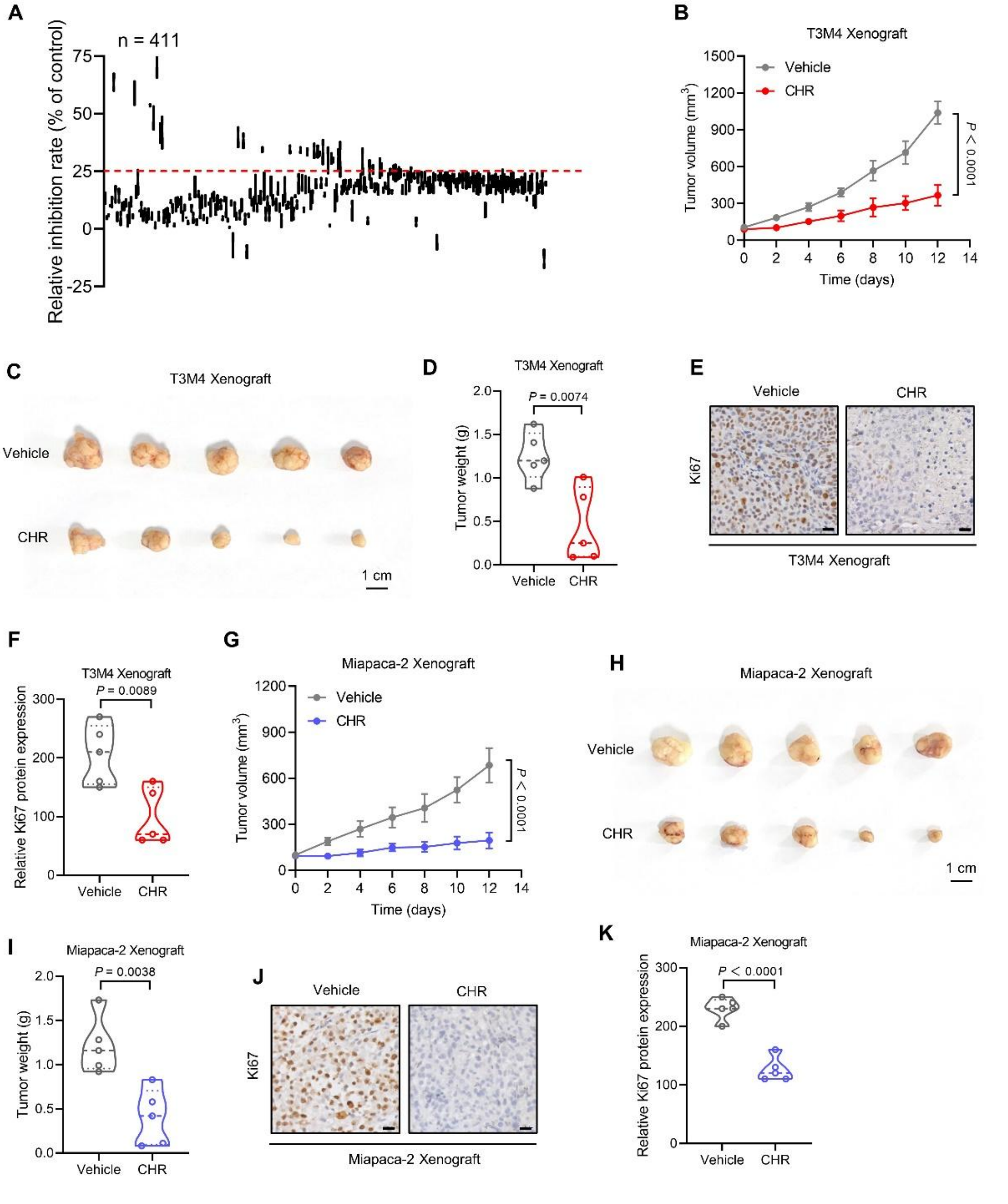

Appendix Figure S1. Drug library screening identified and validated the anti-PC activity of CHR *in vivo* and *in vitro*. (A) Relative inhibition rate of 411 natural compounds from high-throughput screening on Miapaca-2 cells. Compounds with inhibition rates exceeding 25% are shown in Figure 1A. **(B-D)** Tumor volumes (B), images (C) and weights (D) of T3M4 xenografts treated with vehicle or CHR (25 mg/kg) for 12 days. Two-way ANOVA was used to analyze statistical differences in (B). Mean

with ± SEM. Student's t test was used to analyze statistical differences in (D). N = 5 independent mice. **(E-F)** Representative images (E) and quantification (F) of immunohistochemical staining for Ki67 of T3M4 xenografts, scale bar: 20 μm. Student's t test was used to analyze statistical differences in (F). N = 5 independent mice. **(G-I)** Tumor volumes (G), images (H) and weights (I) of Miapaca-2 xenografts treated with vehicle or CHR (25 mg/kg) for 12 days. Two-way ANOVA was used to analyze statistical differences in (G). Mean with ± SEM. Student's t test was used to analyze statistical differences in (I). N = 5 independent mice. **(J-K)** Representative images (J) and quantification (K) of immunohistochemical staining for Ki67 of Miapaca-2 xenografts, scale bar: 20 μm. Student's t test was used to analyze statistical differences in (K). N = 5 independent mice.

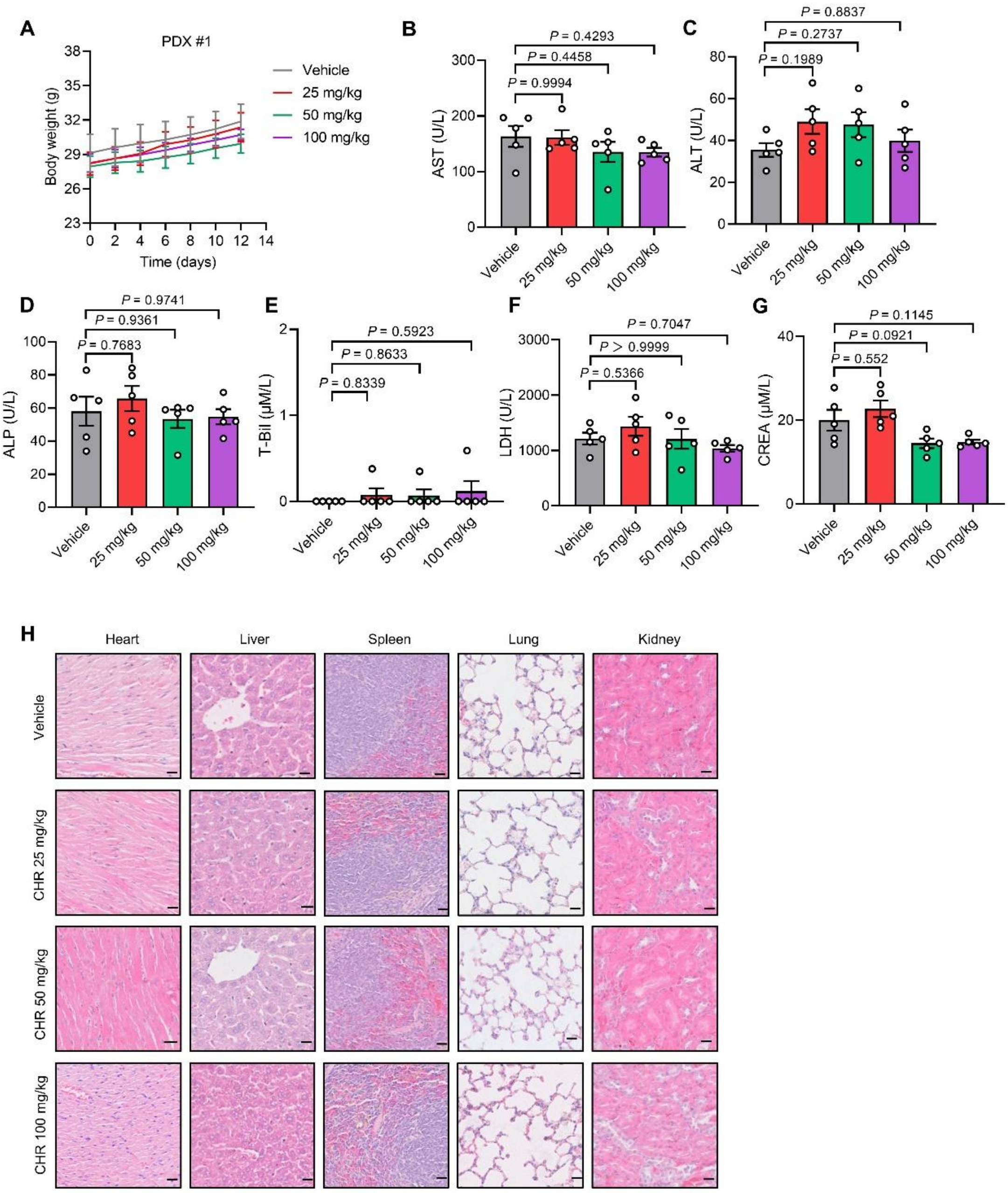

Appendix Figure S2. CHR treatment shows no obvious systemic toxicity *in vivo*.

(A) Body weight changes of PDX#1 xenograft mice treated with vehicle or gradient doses of CHR (25, 50, 100 mg/kg) for 12 days. Mean with ± SEM. N = 5 independent mice. **(B-G)** Serum levels of AST(B), ALT (C), ALP (D), T-Bil (E), LDH (F) , and CREA (G) in PDX #1 xenografts receiving the above-indicated CHR treatments. One-way ANOVA was used to analyze statistical differences. Mean with ± SEM. N = 5

independent mice. **(H)** Representative H&E staining images of major organs (heart, liver, spleen, lung, and kidney) from mice under different CHR dose treatments, scale bars: 20 μm.

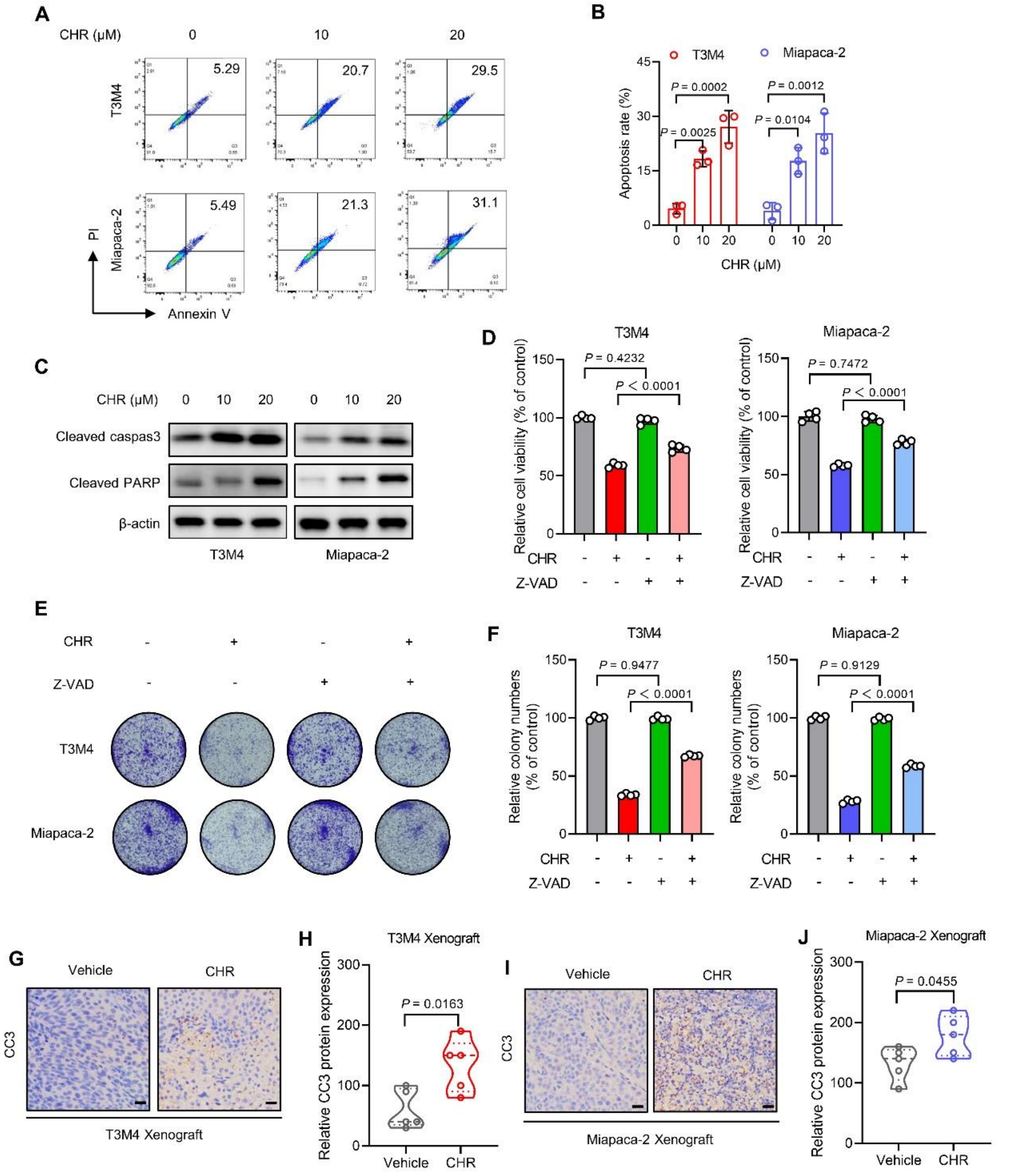

Appendix Figure S3. CHR induces apoptosis in PC cells. (A-B) Flow cytometric analysis (A) and quantification (B) of apoptosis rate in T3M4 and Miapaca-2 cells treated with indicated concentrations of CHR for 48 h. One-way ANOVA was used to analyze statistical differences in (B). Mean with ± SD. N = 3 independent biological replicates. **(C)** Immunoblotting analysis of CC3 and cleaved PARP in CHR-treated T3M4 and Miapaca-2 cells. **(D)** Cell viability of T3M4 and Miapaca-2 cells treated with

CHR (10 μM) in the presence or absence of Z-VAD (10 μM). One-way ANOVA was used to analyze statistical differences. Mean with ± SD. N = 4 independent biological replicates. **(E-F)** Representative images (E) and quantification of colony numbers (F) of T3M4 and Miapaca-2 cells treated with CHR in the presence or absence of Z-VAD. One-way ANOVA was used to analyze statistical differences in (F). Mean with ± SD. N = 4 independent biological replicates. **(G-J)** Representative images (G, I) and quantification (H, J) of immunohistochemical staining for CC3 of T3M4 and Miapaca-2 xenografts treated with vehicle or CHR, scale bar: 20 μm. Student's t test was used to analyze statistical differences in (H, J). N = 5 independent mice.

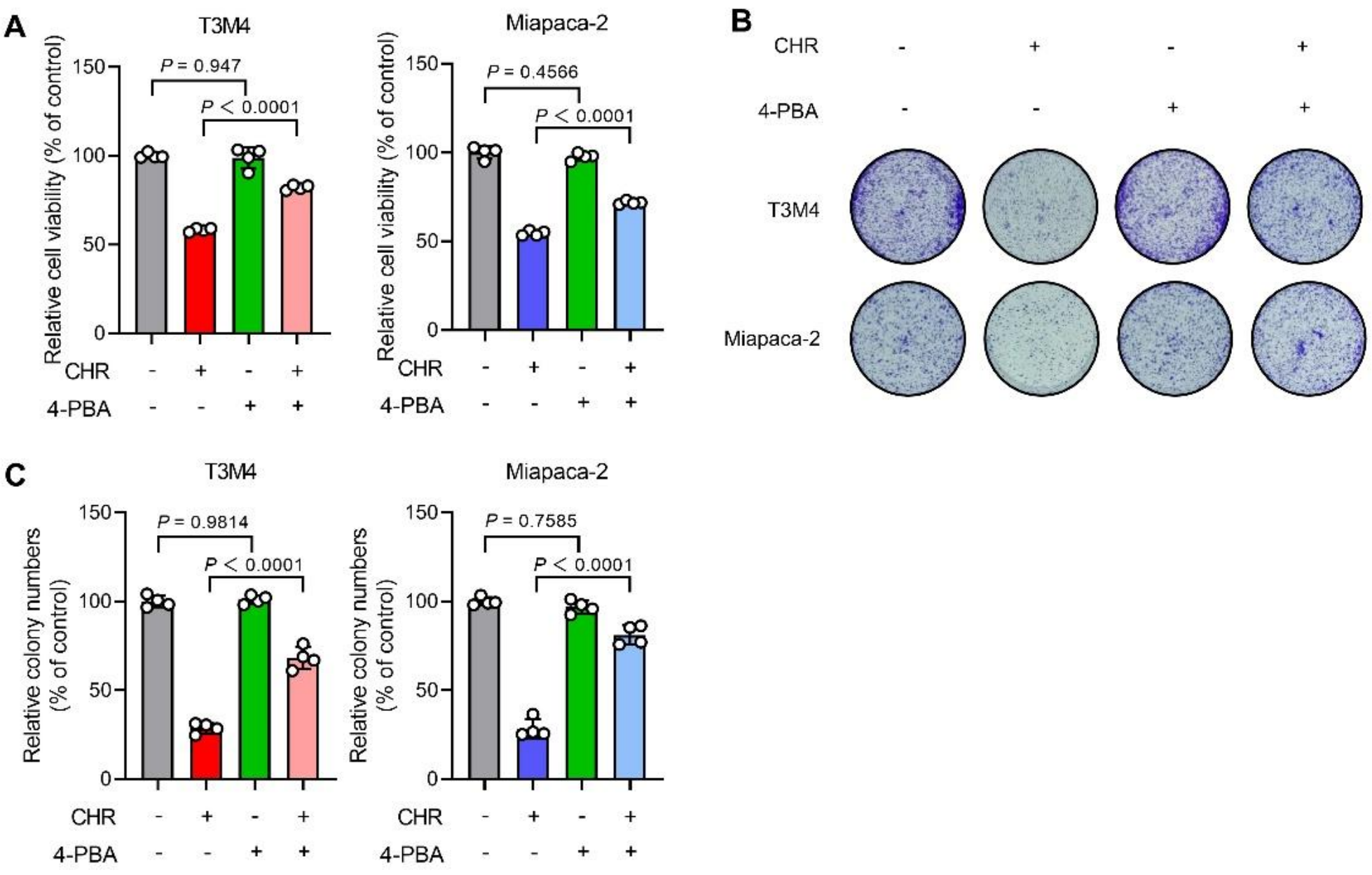

Appendix Figure S4. Inhibition of ER stress reverses CHR-mediated anti-PC effects. (A) Cell viability of T3M4 and Miapaca-2 cells treated with CHR (10 μM) in the presence or absence of 4-PBA (2 mM). One-way ANOVA was used to analyze statistical differences. Mean with ± SD. N = 4 independent biological replicates. **(B-C)** Representative images (B) and quantification of colony numbers (C) of T3M4 and Miapaca-2 cells treated with CHR in the presence or absence of 4-PBA. One-way ANOVA was used to analyze statistical differences in (C). Mean with ± SD. N = 4 independent biological replicates.

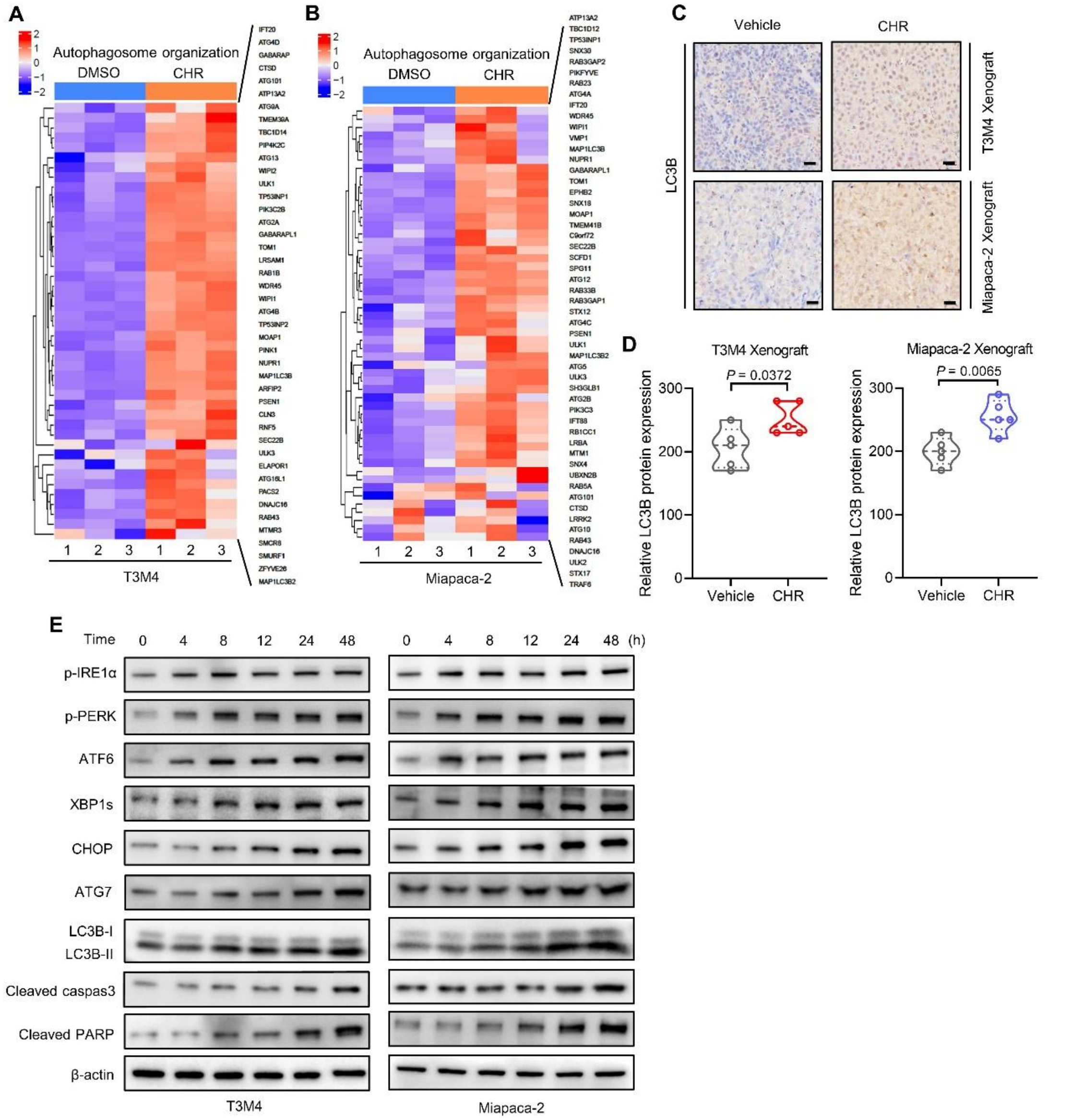

Appendix Figure S5. CHR triggers ER stress-autophagy-apoptosis cascade in PC cells. (A-B) Heatmaps of the autophagosome organization gene expression in T3M4 (A) and Miapaca-2 (B) cells treated with DMSO or CHR. **(C-D)** Representative images (C) and quantification (D) of immunohistochemical staining for LC3B of T3M4 and Miapaca-2 xenografts treated with vehicle or CHR, scale bar: 20 μm. Student's t test was used to analyze statistical differences in (D). N = 5 independent mice. **(E)** Immunoblotting analysis of ER stress, autophagy, and apoptosis markers in T3M4 and Miapaca-2 cells treated with CHR (20 μM) for indicated time durations.

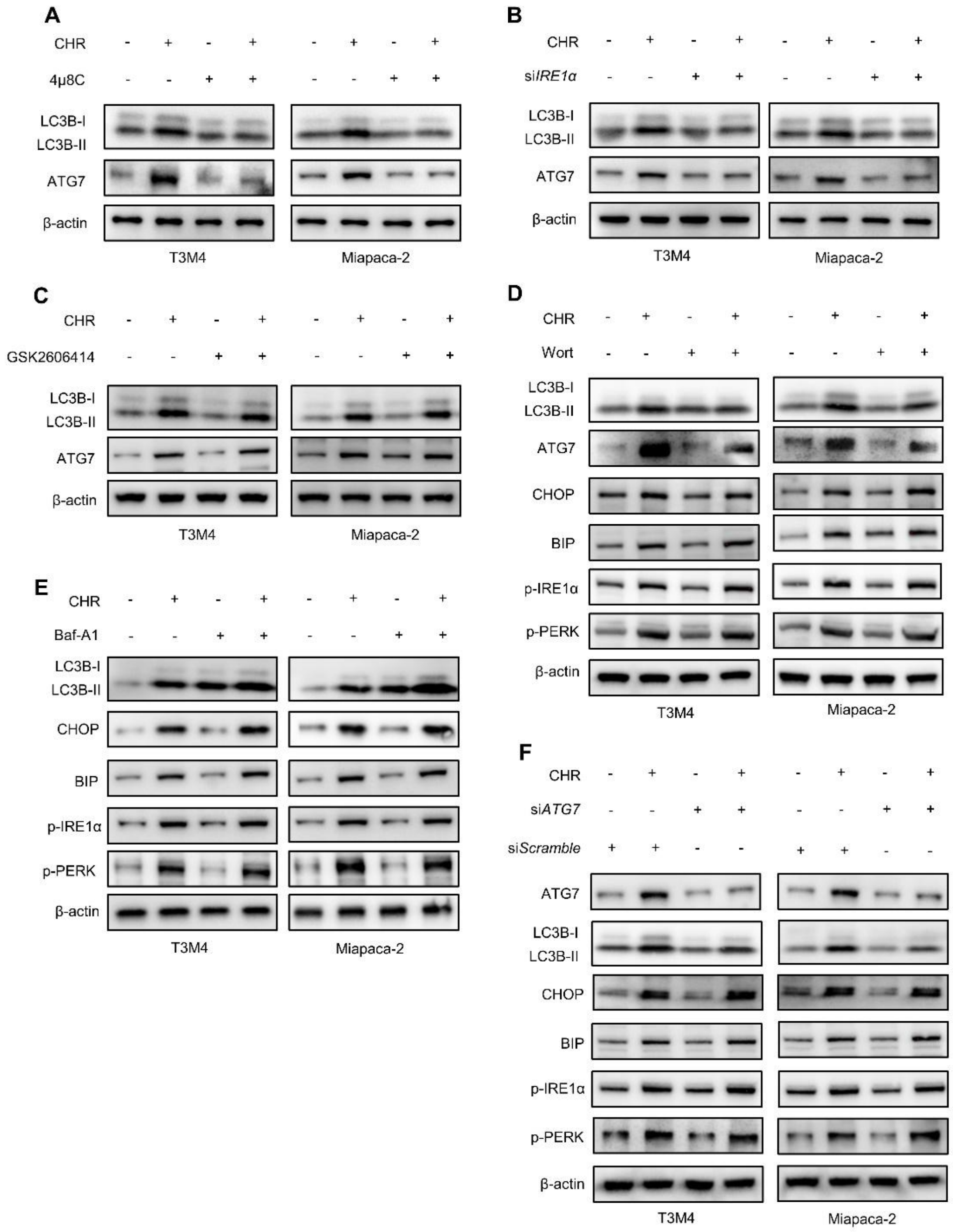

Appendix Figure S6. ER stress acts upstream of autophagy in CHR-treated PC cells. (A) Immunoblotting analysis of autophagy markers in T3M4 and Miapaca-2 cells treated with CHR (10 μM) in the presence or absence of 4μ8C (5 μM) for 48 h. **(B)** Immunoblotting analysis of autophagy markers in T3M4 and Miapaca-2 cells treated with CHR for 48 h after transfected with si*IRE1α* or si*Scramble*. **(C)** Immunoblotting analysis of autophagy markers in T3M4 and Miapaca-2 cells treated with CHR in the presence or absence of GSK2606414 (5 μM) for 48 h. **(D)** Immunoblotting analysis of

autophagy and ER stress markers in T3M4 and Miapaca-2 cells treated with CHR for 48 h in the presence or absence of Wort (1 μM). **(E)** Immunoblotting analysis of autophagy and ER stress markers in T3M4 and Miapaca-2 cells pretreated with Baf-A1 (100 nM) for 6 h, followed by CHR treatment for 48 h. **(F)** Immunoblotting analysis of autophagy and ER stress markers in T3M4 and Miapaca-2 cells treated with CHR for 48 h after transfected with si*ATG7* or si*Scramble*.

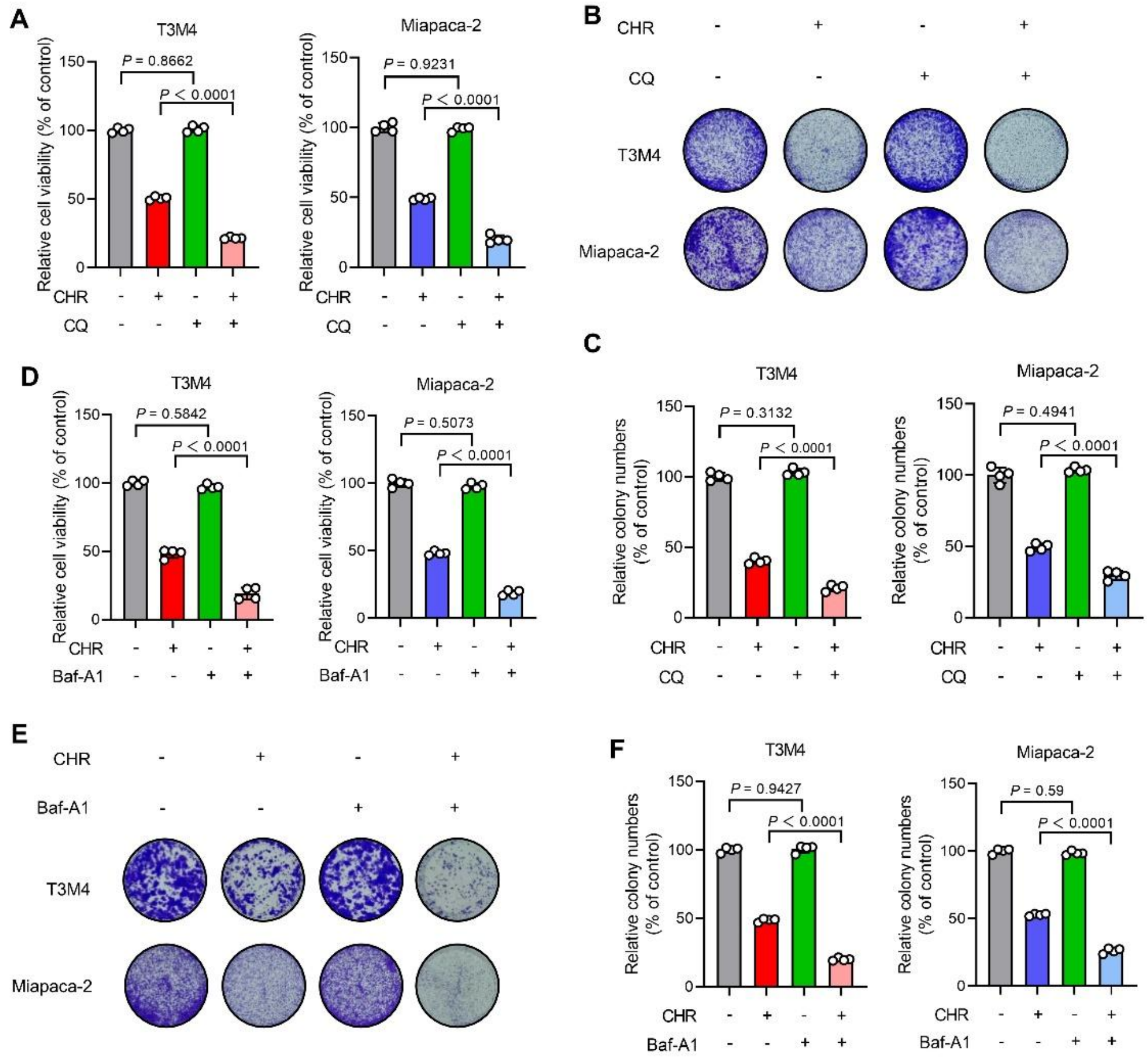

Appendix Figure S7. Pharmacological inhibition of autophagy enhances CHR-induced cytotoxicity in PC cells. (A) Cell viability of T3M4 and Miapaca-2 cells treated with CHR (10 μM) for 48 h in the presence or absence of CQ (10 μM). One-way ANOVA was used to analyze statistical differences. Mean with ± SD. N = 4 independent biological replicates. **(B-C)** Representative images (B) and quantification of colony numbers (C) of T3M4 and Miapaca-2 cells treated with CHR in the presence or absence of CQ. One-way ANOVA was used to analyze statistical differences in (C). Mean with ± SD. N = 4 independent biological replicates. **(D)** Cell viability of T3M4 and Miapaca-2 cells pretreated with Baf-A1 (100 nM) for 6 h, followed by CHR treatment for 48 h. One-way ANOVA was used to analyze statistical differences. Mean with ± SD. N = 4 independent biological replicates. **(E-F)** Representative images (E) and quantification of colony numbers (F) of T3M4 and Miapaca-2 cells treated with

CHR in the presence or absence of Baf-A1. One-way ANOVA was used to analyze statistical differences in (F). Mean with ± SD. N = 4 independent biological replicates.

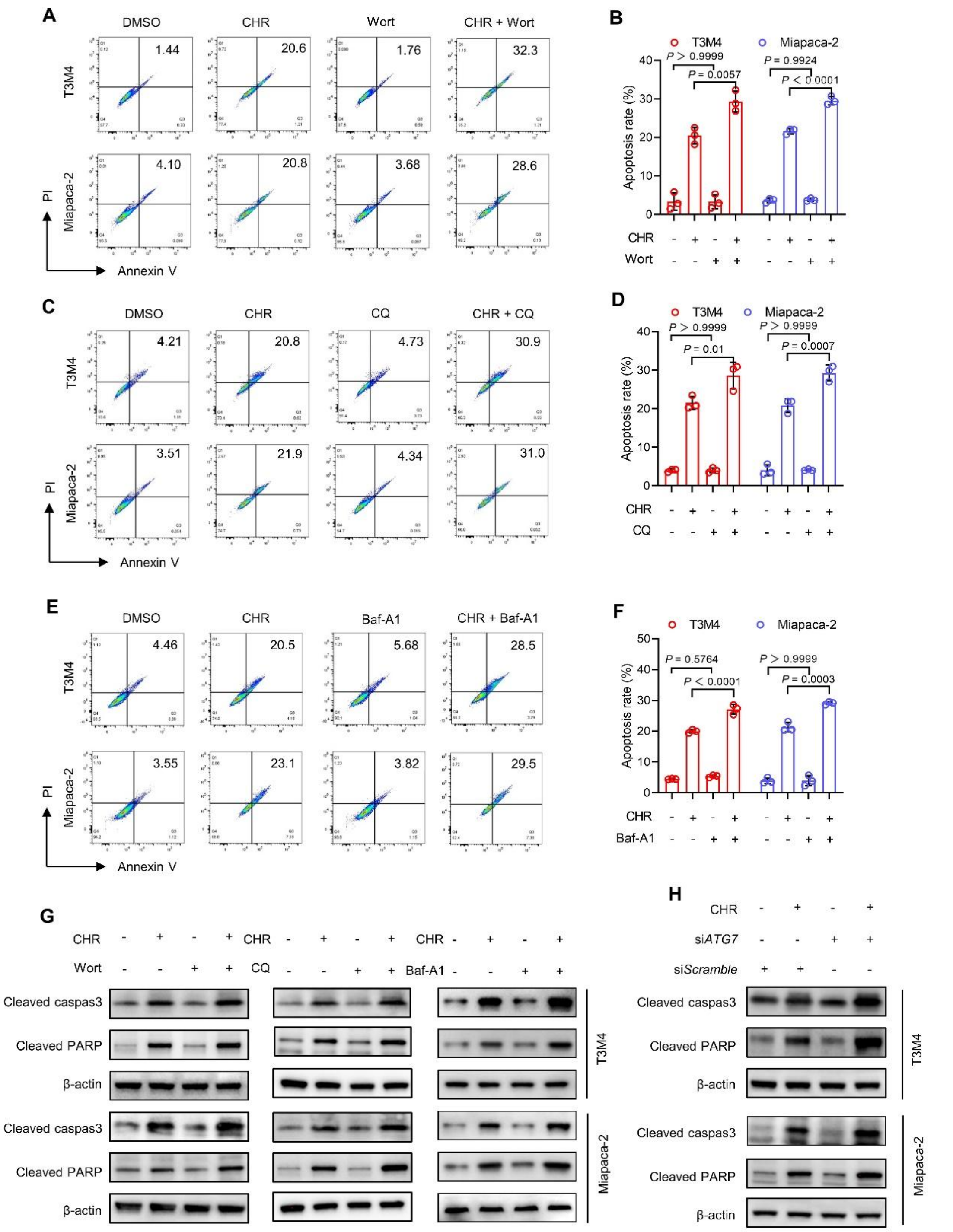

Appendix Figure S8. Inhibition of autophagy enhances CHR-induced apoptosis in PC cells. (A-B) Flow cytometric analysis (A) and quantification (B) of apoptosis rate in T3M4 and Miapaca-2 cells treated with CHR (10 μM) for 48 h in the presence or absence of Wort (1 μM). One-way ANOVA was used to analyze statistical differences in (B). Mean with ± SD. N = 3 independent biological replicates. **(C-D)** Flow

cytometric analysis (C) and quantification (D) of apoptosis rate in T3M4 and Miapaca-2 cells treated with CHR for 48 h in the presence or absence of CQ (10 μM). One-way ANOVA was used to analyze statistical differences in (D). Mean with ± SD. N = 3 independent biological replicates. **(E-F)** Flow cytometric analysis (E) and quantification (F) of apoptosis rate in T3M4 and Miapaca-2 cells pretreated with Baf-A1 (100 nM) for 6 h, followed by CHR treatment for 48 h. One-way ANOVA was used to analyze statistical differences in (F). Mean with ± SD. N = 3 independent biological replicates. **(G)** Immunoblotting analysis of CC3 and cleaved PARP in T3M4 and Miapaca-2 cells treated with CHR for 48 h in the presence or absence of Wort, CQ, or pretreated with Baf-A1 (100 nM) for 6 h, followed by CHR treatment for 48 h. **(H)** Immunoblotting analysis of CC3 and cleaved PARP in T3M4 and Miapaca-2 cells treated with CHR for 48 h after transfected with si*ATG7* or si*Scramble*.

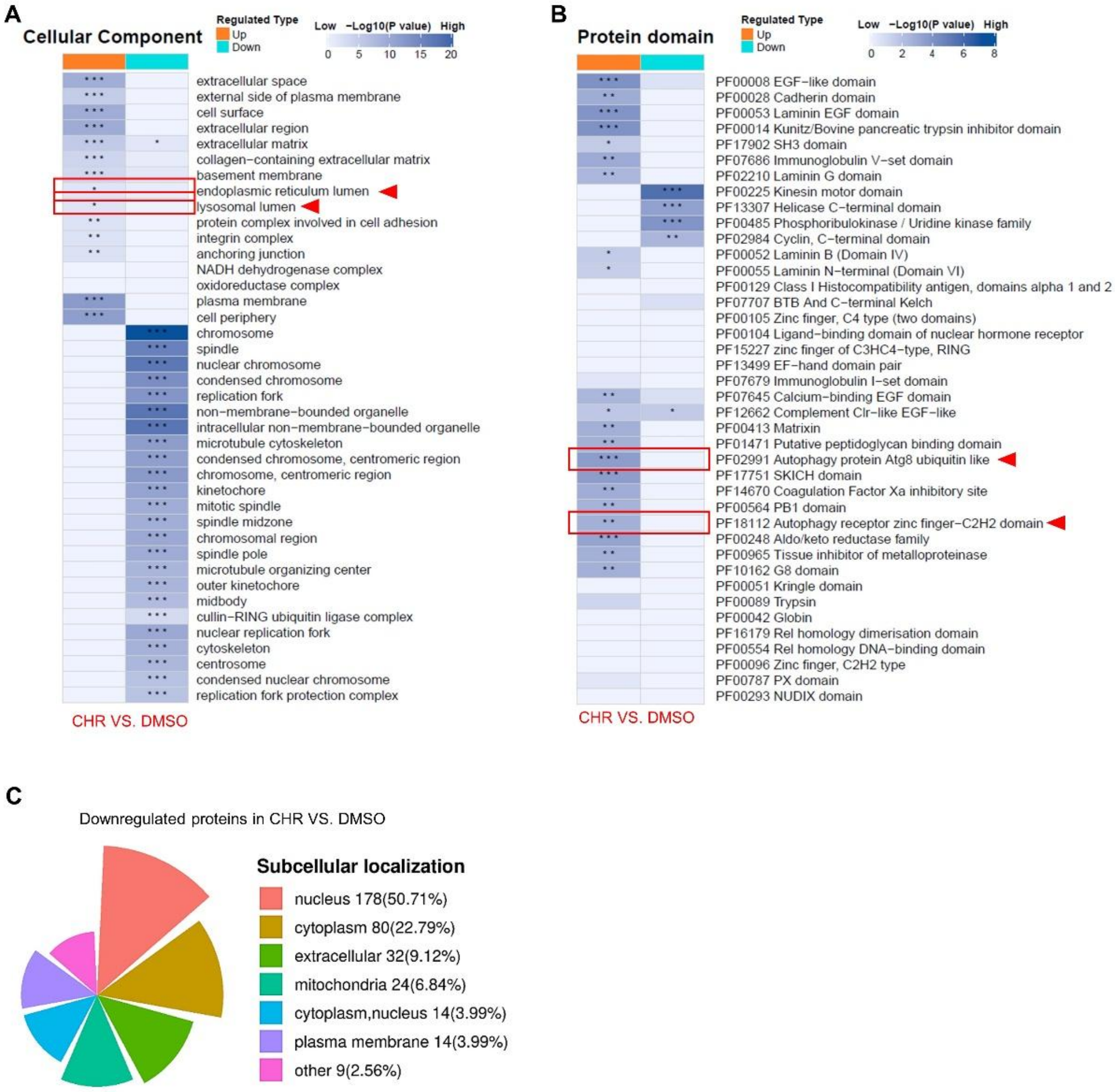

Appendix Figure S9. CHR-induced proteomic alterations in cellular components and protein subcellular localization. (A-B) Gene Ontology analysis of significantly up- and down-regulated cellular components (A) and protein domains (B) in cells treated with CHR versus DMSO. **(C)** Subcellular localization distribution of downregulated proteins in CHR versus DMSO group.

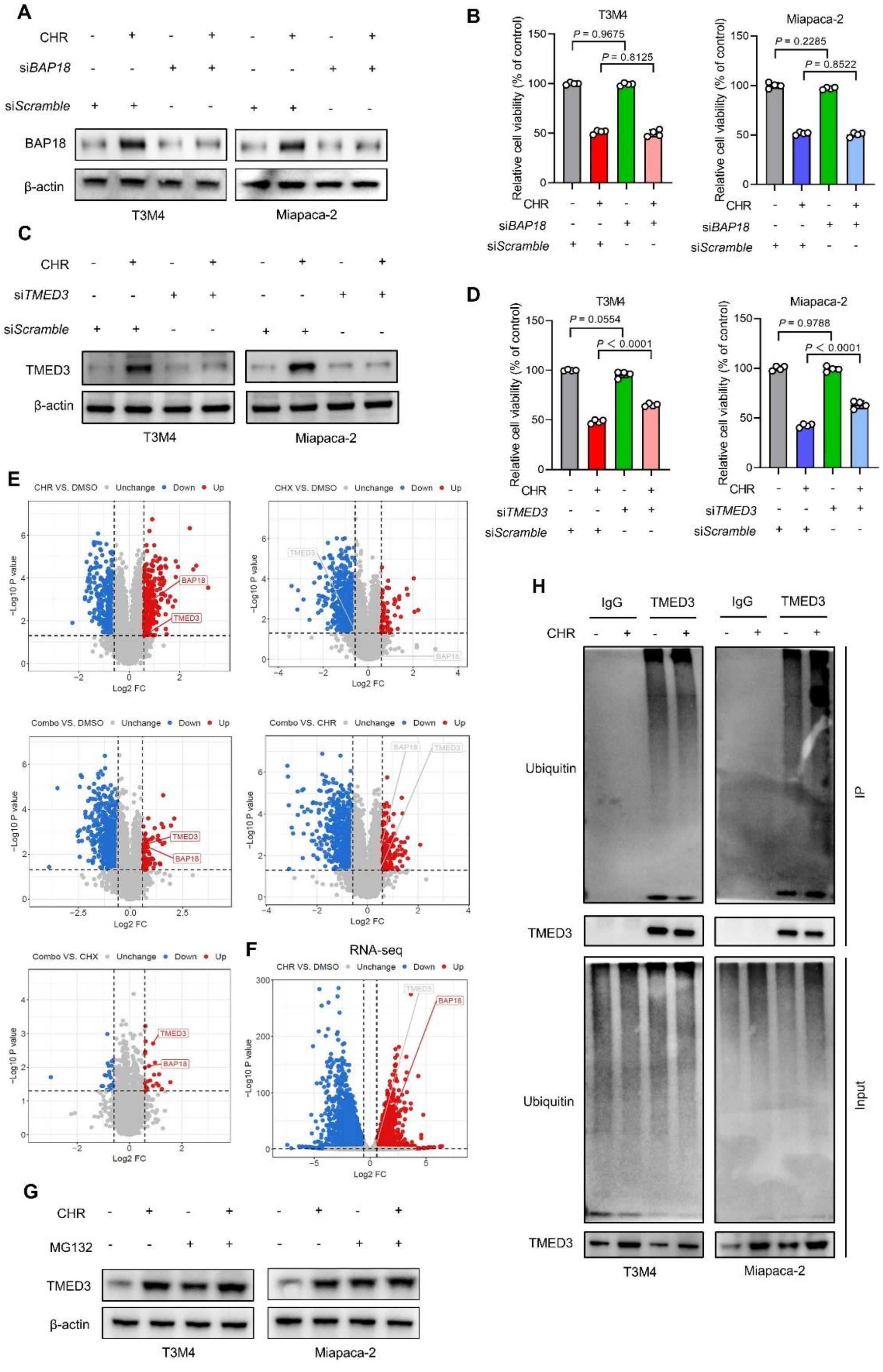

Appendix Figure S10. Ubiquitin-independent TMED3 accumulation contributes to CHR anti-PC activity. (A) Immunoblotting analysis of BAP18 in T3M4 and Miapaca-2 cells treated with CHR (10 μM) for 48 h after transfected with si*BAP18* or si*Scramble*. **(B)** Cell viability of T3M4 and Miapaca-2 cells treated with CHR for 48 h

after transfected with si*BAP18* or si*Scramble*. One-way ANOVA was used to analyze statistical differences. Mean with ± SD. N = 4 independent biological replicates. **(C)** Immunoblotting analysis of TMED3 in T3M4 and Miapaca-2 cells treated with CHR for 48 h after transfected with si*TMED3* or si*Scramble*. **(D)** Cell viability of T3M4 and Miapaca-2 cells treated with CHR for 48 h after transfected with si*TMED3* or si*Scramble*. One-way ANOVA was used to analyze statistical differences. Mean with ± SD. N = 4 independent biological replicates. **(E)** Volcano plots of differentially expressed proteins in cells under different treatment comparisons from proteomics sequencing. **(F)** Volcano plot of differentially expressed genes in cells treated with CHR versus DMSO from RNA sequecing. **(G)** Immunoblotting analysis of TMED3 in T3M4 and Miapaca-2 cells pretreated with MG132 (5 μM) for 24 h, followed by CHR treatment for 48 h. **(H)** Co-immunoprecipitation analysis verifying the interaction between TMED3 and ubiquitin in T3M4 and Miapaca-2 cells following 48 h CHR treatment.

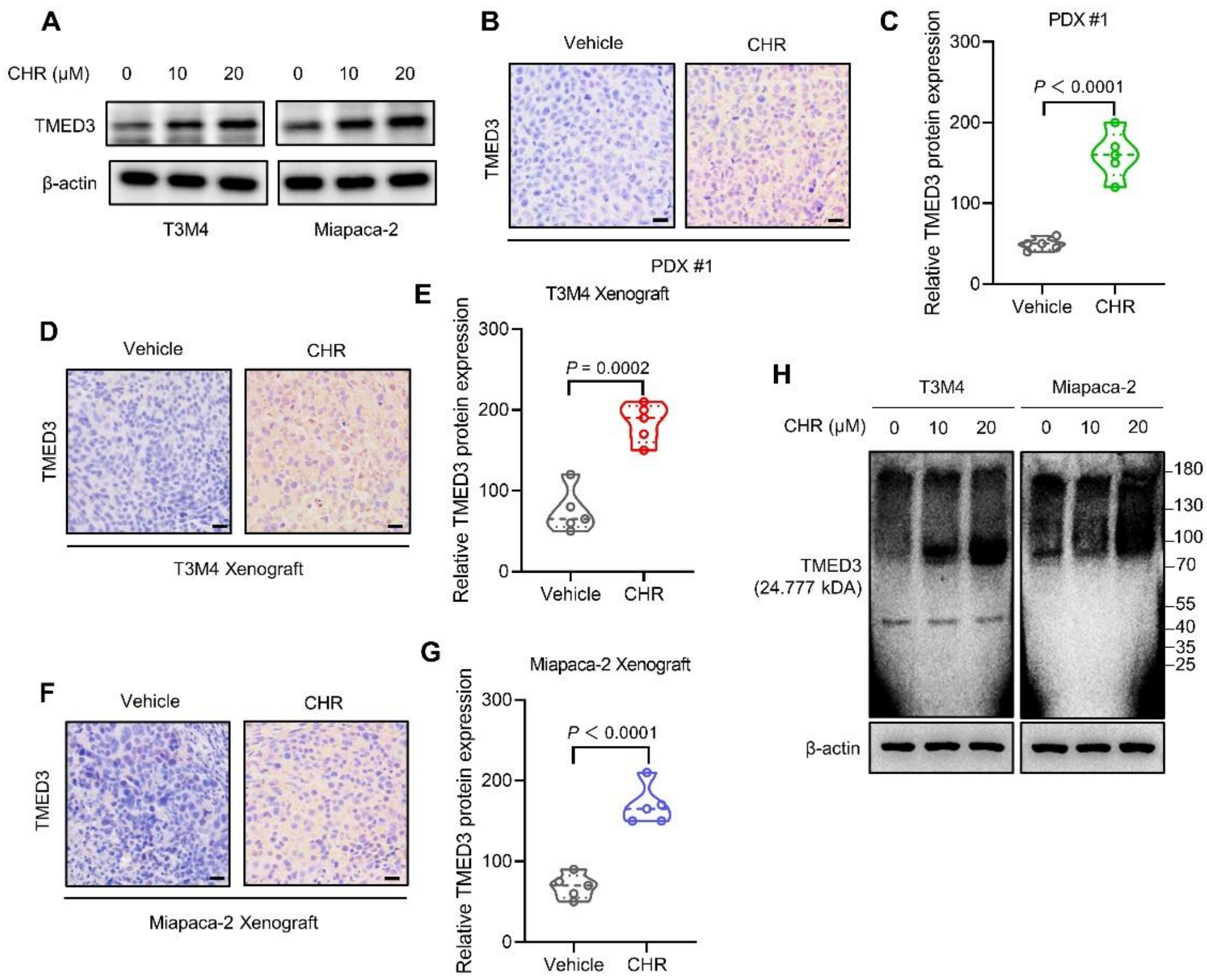

Appendix Figure S11. TMED3 is upregulated in CHR-treated PC cells *in vitro* and *in vivo*. (A) Immunoblotting analysis of TMED3 expression in T3M4 and Miapaca-2 cells treated with indicated concentrations of CHR for 48 h. **(B-G)** Representative images (B, D, F) and quantification (C, E, G) of immunohistochemical staining for TMED3 of PDX #1, T3M4 and Miapaca-2 xenografts treated with vehicle or CHR, scale bar: 20 μm. Student's t test was used to analyze statistical differences in (C, E, G). N = 5 independent mice. **(H)** Native gel electrophoresis analysis of CHR-induced TMED3 oligomerization in T3M4 and Miapaca-2 cells. β-actin (reducing gel) as the quantitative loading control.

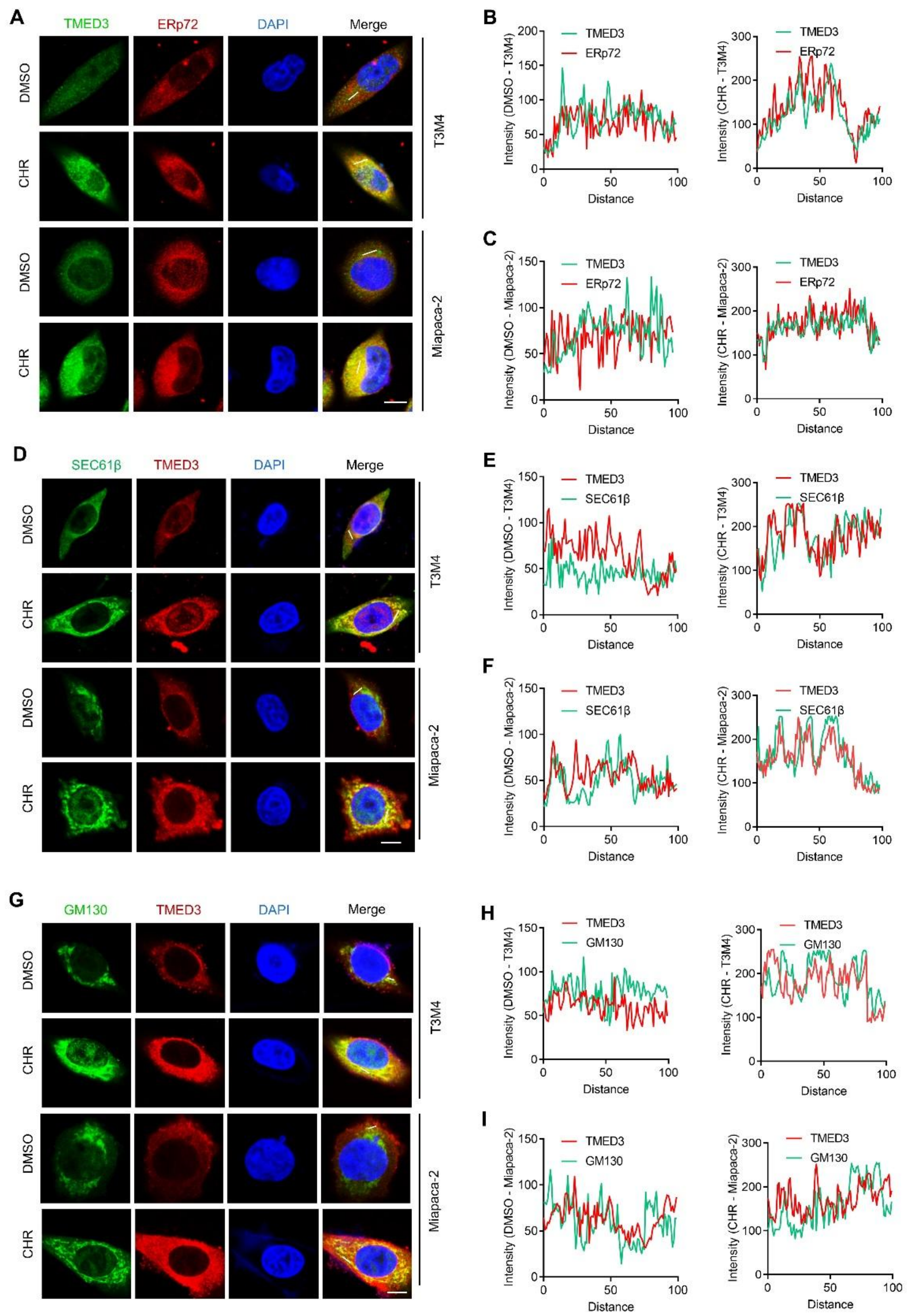

Appendix Figure S12. CHR enhances TMED3 colocalization with endoplasmic reticulum- and Golgi-resident markers. (A-C) Representative images (A) and colocalization analysis of TMED3 and ERp72 (B-C) of immunofluorescent staining for TMED3 (green), ERp72 (red) and DAPI (blue) in T3M4 and Miapaca-2 cells treated

with DMSO or CHR, scale bar: 10 μm. **(D-F)** Representative images (D) and colocalization analysis of SEC61β and TMED3 (E-F) of immunofluorescent staining for SEC61β (green), TMED3 (red) and DAPI (blue) in T3M4 and Miapaca-2 cells treated with DMSO or CHR, scale bar: 10 μm. **(G-I)** Representative images (G) and colocalization analysis of GM130 and TMED3 (H-I) of immunofluorescent staining for GM130 (green), TMED3 (red) and DAPI (blue) in T3M4 and Miapaca-2 cells treated with DMSO or CHR, scale bar: 10 μm.

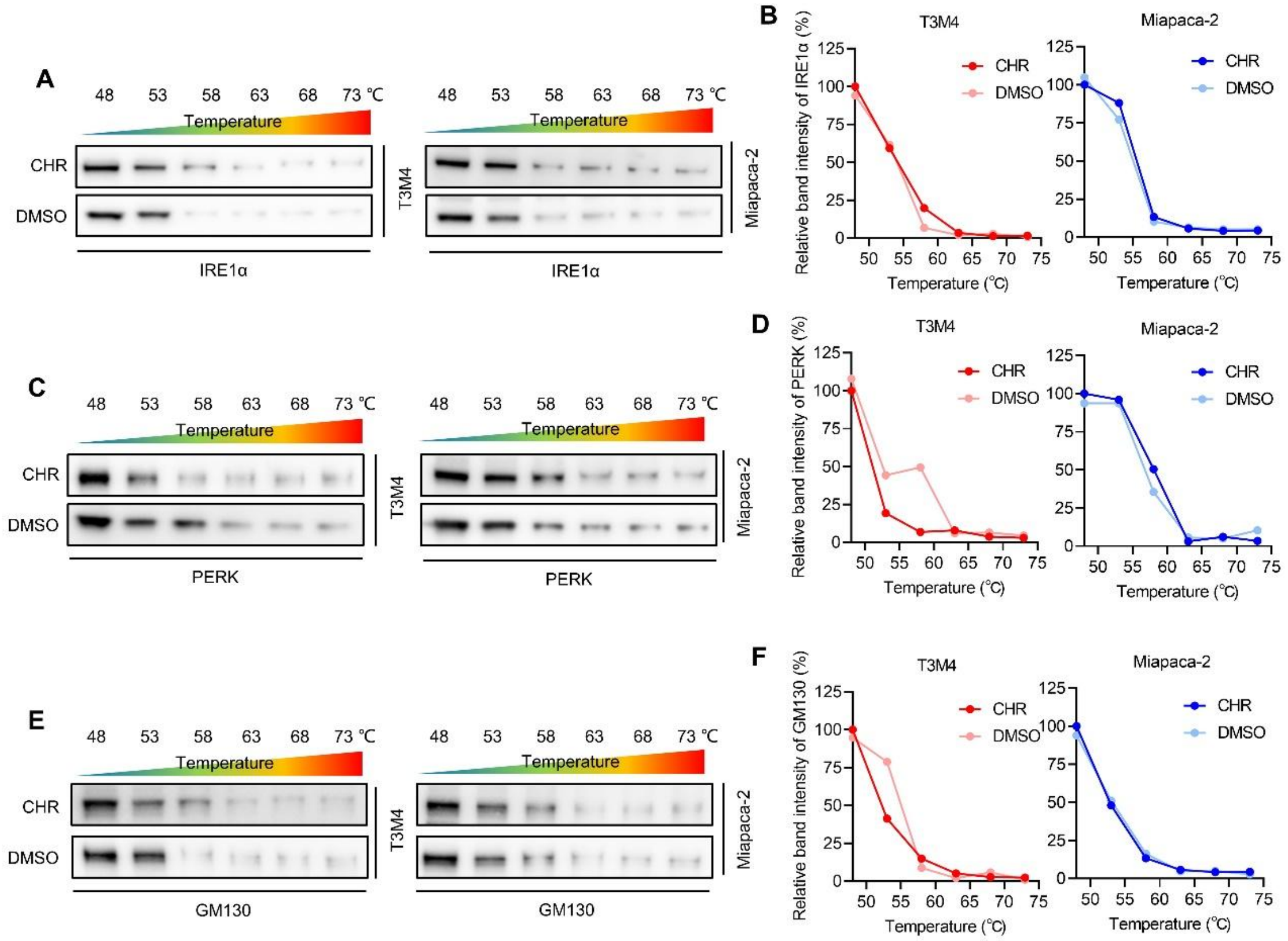

Appendix Figure S13. CHR does not directly interact with ER- or Golgi-resident proteins other than TMED3. (A-B) Immunoblotting analysis (A) and quantification of relative band intensity (B) of CETSA for CHR binding to IRE1α in T3M4 and Miapaca-2 cells. **(C-D)** Immunoblotting analysis (C) and quantification of relative band intensity (D) of CETSA for CHR binding to PERK in T3M4 and Miapaca-2 cells. **(E-F)** Immunoblotting analysis (E) and quantification of relative band intensity (F) of CETSA for CHR binding to GM130 in T3M4 and Miapaca-2 cells.

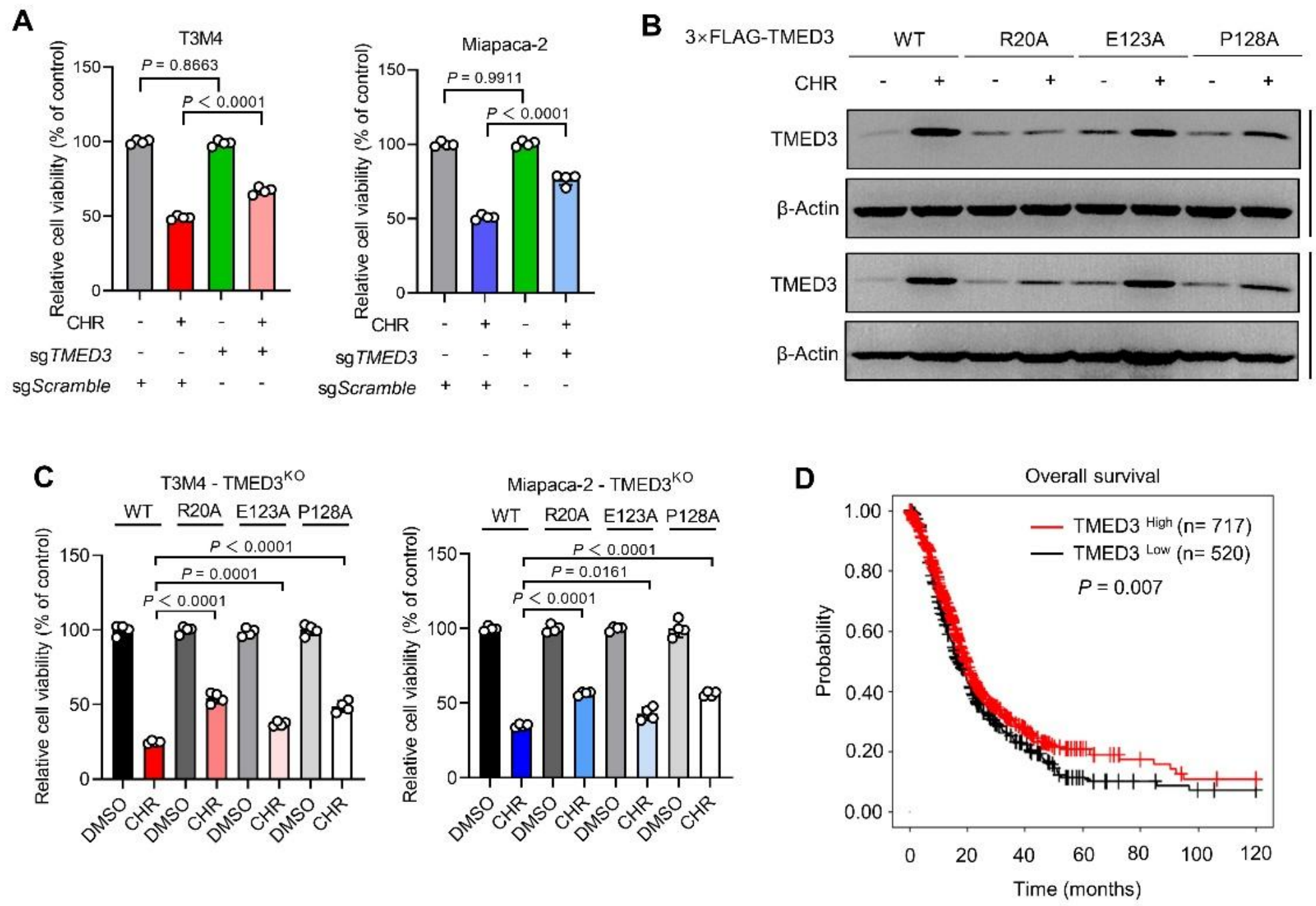

Appendix Figure S14. The binding affinity between CHR and TMED3 modulates CHR-induced cytotoxicity. (A) Cell viability of wild-type and TMED3-KO T3M4 and Miapaca-2 cells treated with CHR for 48 h. One-way ANOVA was used to analyze statistical differences. Mean with ± SD. N = 4 independent biological replicates. **(B)** Immunoblotting for TMED3 in TMED3-KO T3M4 and Miapaca-2 cells re-expressing wild-type TMED3 or its mutants (R20A, E123A, P128A) after 48 h of CHR or DMSO treatment. **(C)** Cell viability of wild-type, R20A, E123A, and P128A mutant-reconstituted TMED3-KO T3M4 and Miapaca-2 cells after 48 h of CHR (20 μM) or DMSO treatment. One-way ANOVA was used to analyze statistical differences. Mean with ± SD. N = 4 independent biological replicates. **(D)** Survival analysis of PC patients according to TMED3 expression via the online Kaplan-Meier Plotter platform.

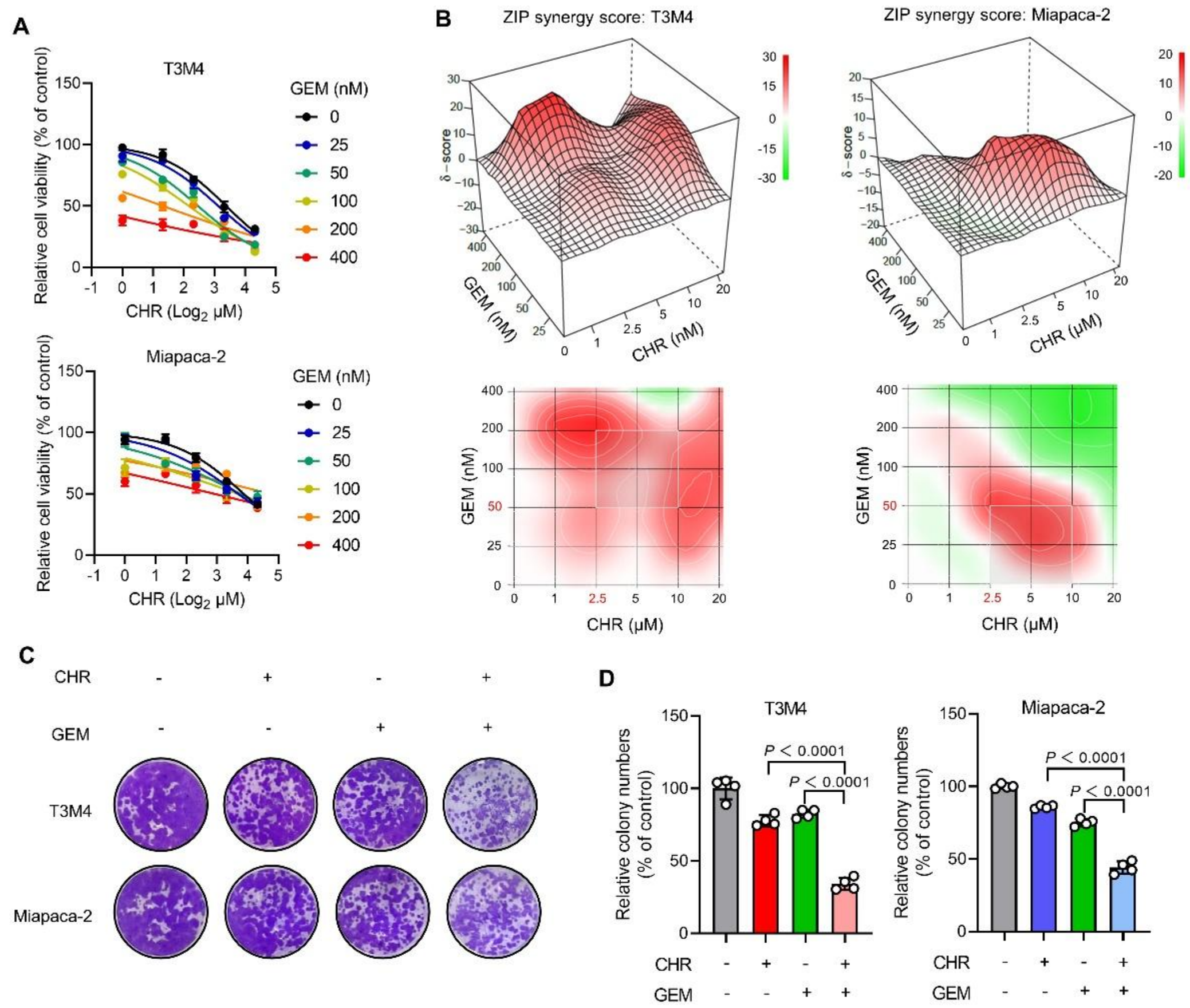

Appendix Figure S15. The combination therapy of CHR and GEM synergistically kills PC cells. (A) Dose-response curves showing relative cell viability of T3M4 and Miapaca-2 cells after 72 h treatment with increasing concentrations of CHR in the presence of escalating doses of GEM. Mean with ± SD. N = 3 independent biological replicates. **(B)** ZIP synergy analysis of CHR and GEM combination in T3M4 and Miapaca-2 cells. Top: 3D synergy surface plots; bottom: 2D synergy heatmaps. **(C-D)** Representative images (C) and quantification of colony numbers (D) of T3M4 and Miapaca-2 cells treated with CHR alone, GEM alone or combination. One-way ANOVA was used to analyze statistical differences in (D). Mean with ± SD. N = 4 independent biological replicates.

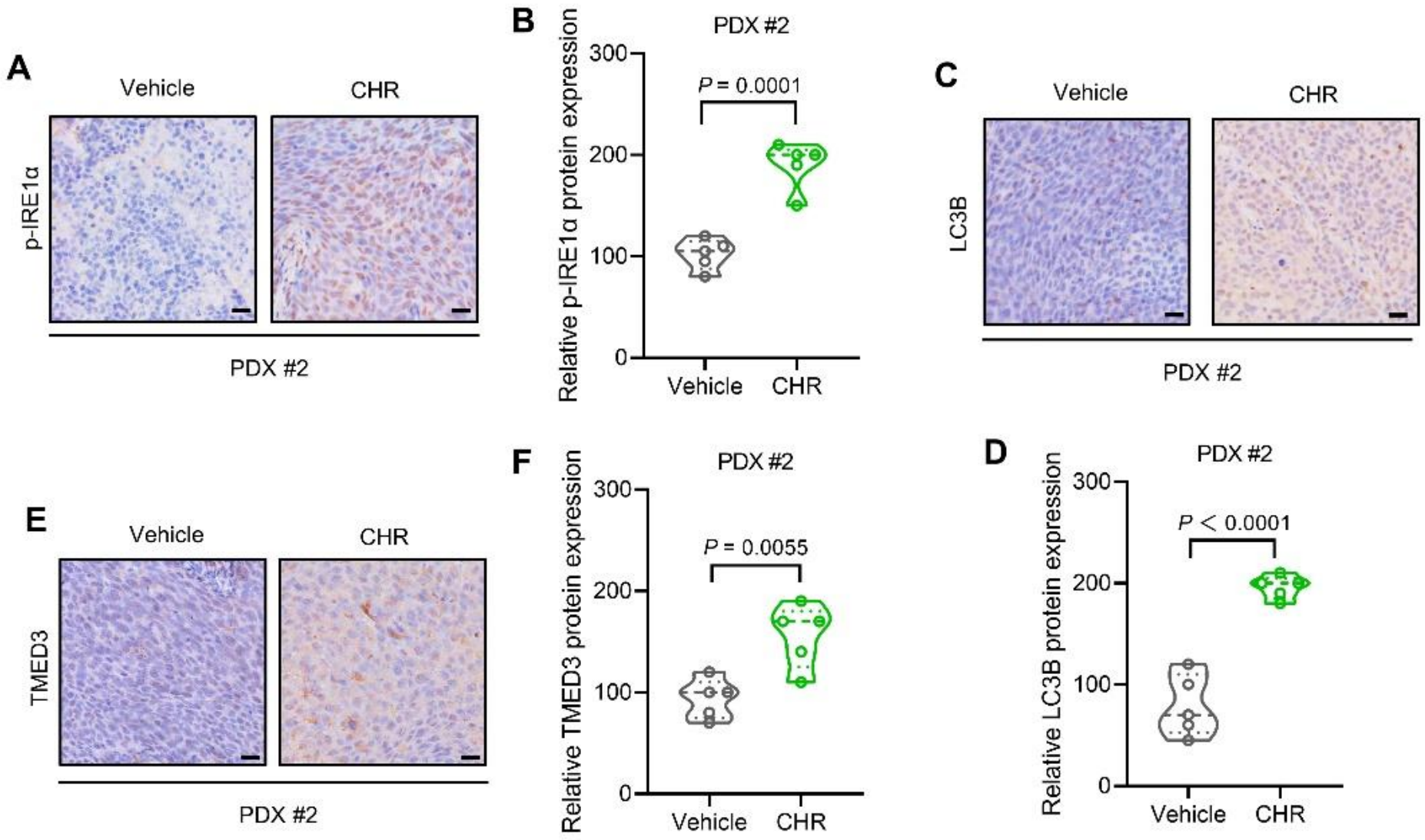

Appendix Figure S16. CHR-related phenotypic pathways are observed in the PDX model. (A-F) Representative images (A, C, E) and quantification (B, D, F) of immunohistochemical staining for p-IRE1α, LC3B and TMED3 of PDX #2 xenografts treated with vehicle or CHR for 12 days, scale bar = 20 μm. Student's t test was used to analyze statistical differences in (B, D, F). N = 5 independent mice.
