# Supplementary material for: Chrysosplenetin-induced TMED3 aggregation triggers unfolded protein response in pancreatic cancer
Source: EMBO Mol Med. 2026 Aug 13;18(9):3790–823. doi: 10.1038/s44321-026-00506-5 (PMC13562749; doi:10.1038/s44321-026-00506-5)
Supplement: Supplementary file 3 [file 44321_2026_506_MOESM2_ESM.pdf]

EMBO Molecular Medicine

Chrysosplenetin-induced TMED3 aggregation triggers unfolded protein response in pancreatic cancer

Zhe Zhang, Ningna Weng, Xuanhao Gu, Yi Zang, Qitai Chen, Rujia Zheng, Xishan Yang, Gubu Amu, Danyang Zhao, Zhihao Ma, Jinyan Huang, Tingbo Liang, and Qi Zhang

Corresponding authors: Zhe Zhang (scuzz@zju.edu.cn) , Tingbo Liang (liangtingbo@zju.edu.cn), Qi Zhang (qi.zhang@zju.edu.cn)

Review Timeline:

Submission Date:	8th Sep 25
Editorial Decision:	23rd Sep 25
Revision Received:	1st Jun 26
Editorial Decision:	29th Jun 26
Revision Received:	5th Jul 26
Editorial Decision:	17th Jul 26
Revision Received:	21st Jul 26
Accepted:	30th Jul 26

Editor: Lise Roth

Transaction Report:

(Note: With the exception of the correction of typographical or spelling errors that could be a source of ambiguity, letters and reports are not edited. Depending on transfer agreements, referee reports obtained elsewhere may or may not be included in this compilation. Referee reports are anonymous unless the Referee chooses to sign their reports.)

23rd Sep 2025

Dear Dr. Zhang,

Thank you for the submission of your manuscript to EMBO Molecular Medicine. We have now received feedback from the three reviewers who agreed to evaluate your manuscript.

As you will see from the enclosed reports, the reviewers agree that the findings are of potential interest, but they also raise major and partially overlapping concerns related to the weak mechanistic insight and overall limited experimental rigor. Based on the nature of the concerns and the amount of time and work that would be needed to address them, considering also that at EMBO Press we encourage a single round of revisions in a limited time frame, we prefer to return the manuscript to you at this point with the decision that we cannot offer to publish it.

However, given the potential interest of the findings, we would welcome a new manuscript on the same topic if you obtained data that would address the referees' concerns in full.

To be completely clear, however, I would like to stress that if you were to send a revised manuscript, this would be treated as a new submission rather than a revision and would be reviewed afresh, in particular with respect to the literature and the novelty of your findings at the time of resubmission. If you decide to follow this route, please make sure you nevertheless upload a letter of response to the referees' comments.

I am sorry that I could not bring better news this time and hope that the referee comments are helpful in your continued work in this area.

With kind regards,

Lise Roth

Lise Roth, Ph.D
Senior Editor
EMBO Molecular Medicine

***** Reviewer's comments *****

Referee #1 (Comments on Novelty/Model System for Author):

Limited in vivo efficacy : 1/ The antitumor effect of CHR in PDX models is modest: no more than ~50% tumor volume reduction at a relatively high dose (25 mg/kg); 2/ The reported tumor volumes in PDX models show very low variability, which is unusual for these heterogeneous systems; 3/ Many natural and synthetic compounds have demonstrated similar or stronger preclinical efficacy but almost never advanced to the clinic.
Questionable in vitro potency : 1/ The IC50 values range from just above 10 µM to over 30 µM, indicating low cytotoxic efficiency; 2/ For a compound with clinical aspirations, one would expect more consistent potency in the low micromolar or nanomolar range.
Weak evidence for TMED3 as a target : 1/ The siRNA-mediated knockdown of TMED3 is relatively inefficient, as shown by the incomplete reduction in the western blot (Figure 6A); 2/ Even with this knockdown, the effects on ER stress markers are minimal, suggesting that TMED3 is not an essential driver of the phenotype induced by CHR; 3/ If TMED3 were truly the critical target, its genetic inhibition should mimic (or at least approximate) the biological effects of CHR, which is not the case; 4/ More studies with the siTMED3 must be performed to validate TMED3 as an essential target.
Mechanistic shortcomings : 1/ The authors propose that CHR stabilizes TMED3 and triggers its aggregation in the ER, but they do not show whether CHR destabilizes or alters TMED3 turnover (e.g., ubiquitination, proteasomal degradation); 2/ The observed activation of UPR and apoptosis is consistent with what has already been reported for many other flavonoids and natural products. Therefore, the mechanism is not conceptually novel, but rather a reiteration of a well-documented phenomenon.
Overstated clinical relevance : 1/ CHR's activity is neither exceptional in vitro nor in vivo; 2/ The reported binding affinity (Kd ~16 µM) is relatively weak, and the relationship between TMED3 expression and patient survival in pancreatic cancer is not coherently addressed; 3/ Although no major toxicity was detected in mice, this does not offset the limited antitumor efficacy; 4/

Overall, the clinical interest of CHR as a stand-alone or combination therapy is minimal.

Referee #1 (Remarks for Author):

The study presents chrysosplenetin (CHR) as a natural compound with potential anti-pancreatic cancer activity through induction of endoplasmic reticulum (ER) stress, mediated by aggregation of TMED3. While the experimental scope is broad (screening, cell lines, PDOs, PDXs, proteomics, docking, DARTS/CETSA/BLI), several conceptual and methodological limitations considerably reduce the translational relevance of the findings.
Major Comments
Limited in vivo efficacy : 1/ The antitumor effect of CHR in PDX models is modest: no more than ~50% tumor volume reduction at a relatively high dose (25 mg/kg); 2/ The reported tumor volumes in PDX models show very low variability, which is unusual for these heterogeneous systems; 3/ Many natural and synthetic compounds have demonstrated similar or stronger preclinical efficacy but almost never advanced to the clinic.
Questionable in vitro potency : 1/ The IC50 values range from just above 10 µM to over 30 µM, indicating low cytotoxic efficiency; 2/ For a compound with clinical aspirations, one would expect more consistent potency in the low micromolar or nanomolar range.
Weak evidence for TMED3 as a target : 1/ The siRNA-mediated knockdown of TMED3 is relatively inefficient, as shown by the incomplete reduction in the western blot (Figure 6A); 2/ Even with this knockdown, the effects on ER stress markers are minimal, suggesting that TMED3 is not an essential driver of the phenotype induced by CHR; 3/ If TMED3 were truly the critical target, its genetic inhibition should mimic (or at least approximate) the biological effects of CHR, which is not the case; 4/ More studies with the siTMED3 must be performed to validate TMED3 as an essential target.
Mechanistic shortcomings : 1/ The authors propose that CHR stabilizes TMED3 and triggers its aggregation in the ER, but they do not show whether CHR destabilizes or alters TMED3 turnover (e.g., ubiquitination, proteasomal degradation); 2/ The observed activation of UPR and apoptosis is consistent with what has already been reported for many other flavonoids and natural products. Therefore, the mechanism is not conceptually novel, but rather a reiteration of a well-documented phenomenon.
Overstated clinical relevance : 1/ CHR's activity is neither exceptional in vitro nor in vivo; 2/ The reported binding affinity (Kd ~16 µM) is relatively weak, and the relationship between TMED3 expression and patient survival in pancreatic cancer is not coherently addressed; 3/ Although no major toxicity was detected in mice, this does not offset the limited antitumor efficacy; 4/ Overall, the clinical interest of CHR as a stand-alone or combination therapy is minimal.
Final Conclusion
This manuscript provides a wide set of experimental data, but the results do not convincingly support TMED3 as a solid therapeutic target or CHR as a viable clinical candidate. The proposed mechanism (ER stress-UPR-apoptosis) is already well established and lacks true novelty. Given the modest in vivo efficacy, limited in vitro potency, weak evidence for TMED3 as the critical mediator, and the existence of numerous compounds with similar preclinical effects that never reached the clinic, the translational value of this study is very limited.

Referee #2 (Comments on Novelty/Model System for Author):

The manuscript does not clearly indicate whether appropriate ethical approval was obtained for the collection and use of human tumor samples. This requires explicit clarification to ensure the integrity of the work.

Referee #2 (Remarks for Author):

In this manuscript, Zhang et al. investigate chrysosplenetin (CHR) as a potential therapeutic agent in pancreatic cancer (PC), proposing TMED3 as its direct target and linking TMED3 aggregation to ER stress, UPR activation, and apoptosis. The study employs a broad range of experimental approaches, and the identification of TMED3 as a possible novel target in PC is of potential interest. However, several aspects of the mechanistic interpretation remain insufficiently developed, and the manuscript would benefit from substantial revision before it can be considered for publication.
Major Concerns:
1. A central concern is the mechanistic evidence. The authors suggest that CHR induces the UPR through aggregation of TMED3, but the precise mechanism by which TMED3, an ER-localized protein, mediates CHR-induced ER stress and triggers UPR signaling and cell death is not clearly demonstrated. Closely related to this, the causal link between CHR binding to TMED3 and the selective activation of UPR pathways is not fully established.
2. The analysis of UPR signaling is incomplete: while the IRE1 and PERK branches are examined, the ATF6 pathway is not addressed, leaving the overall picture of UPR activation only partially resolved.
3. The treatment of IRE1α, as one of the key mediators of the UPR, requires further detail. Demonstration of IRE1α phosphorylation alone is not sufficient to establish a functional role, and assays such as XBP1 splicing or RIDD activity would strengthen the conclusions.
4. The interpretation of autophagy also requires clarification. The authors conclude that autophagy is cytoprotective in this setting, but it remains unclear whether this response is mediated by IRE1α signaling or reflects a more general stress response.

5. Another important point concerns the reported synergy between CHR and gemcitabine. It is not clear whether the observed effect represents true synergy or a merely additive effect. The criteria for defining synergy are not clearly stated, nor is the type of synergy discussed. Without rigorous quantification, the claim of synergy remains uncertain.
6. Finally, the manuscript does not clearly indicate whether appropriate ethical approval was obtained for the collection and use of human tumor samples. This requires explicit clarification to ensure the integrity of the work.

Minor concerns:
7. Beyond these scientific issues, there are additional weaknesses in the manuscript's structure and compliance with basic standards. The introduction contains material that belongs in the results section, resulting in a lack of clarity and coherence. A stricter separation of introduction, results, and discussion is necessary.
8. Essential prior work on IRE1 and UPR signaling in cancer is not cited, weakening the contextualization of the study.

Referee #3 (Comments on Novelty/Model System for Author):

The reasons are described in the comments to the authors.

Referee #3 (Remarks for Author):

The authors screened a natural product library and identified chrysosplenetin (CHR) as a cytotoxic compound inducing cell death, proliferation arrest, and apoptosis in pancreatic cancer cells in vitro and in vivo. They show that CHR triggers endoplasmic reticulum (ER) stress and autophagy, primarily through specific interaction with the TMED3 protein, an ER/golgi shuttling transmembrane protein, as demonstrated by various molecular and cellular techniques (proteomic analysis, in silico structural modelling, thermal stability shift assay and Biolayer Interferometry). TMED3 silencing reduces CHR impact on ER stress, autophagy and cell death. Finally, authors describe the potential synergistic effect of CHR with chemotherapy or autophagy inhibition for the induction of cancer cell death.

This study's principal novelty lies in characterizing the mechanism of CHR as a TMED3 binder and elucidating its role in ER stress-induced apoptosis in pancreatic cancer, a mode of action so far not described. However, CHR's anti-cancer properties were previously reported for other targets and cancers, which slightly lowers the overall novelty.

Major limitations:
While the manuscript presents an hypothesis regarding the role of CHR, the overall robust-ness of these conclusions remain inadequately supported by the data. The identification of TMED3 as a bona fide target of CHR is quite weak. The rationale behind the Venn diagram used for target exclusion (especially BAP18) is not sufficiently explained, and in silico model-ing of the CHR-TMED3 interaction is not accompanied by any experimental validation (site-directed mutagenesis or biochemical mapping of the binding interface). The use of a so-called "purified TMED3 protein" for affinity measurements is problematic: no source or characterization is presented, making the fundamental evidence for TMED3 targeting am-biguous.
Moreover, siRNAs targeting TMED3 or ATG7 result in only marginal reduction in protein levels and fail to robustly modulate the core readouts (cell viability, autophagy, ER stress marker expression). These limited knockdown efficiency weakens all downstream interpreta-tions. The use of non-specific pharmacological modulators such as wortmannin (a PI3K in-hibitor) and PBA (a chemical chaperone with broad cellular effects) instead of highly selec-tive pathway inhibitors (such as 4uC8, Perki for ER stress; bafilomycin for autophagy) further undermines the specificity of mechanistic claims made for CHR action.
Taken together, while CHR demonstrably induces ER stress, autophagy, and cancer cell death, the data suggest these effects may involve broadly cytotoxic mechanisms similar to those observed with established chemotherapeutics such as gemcitabine or paclitaxel, both of which are known to trigger ER stress responses and apoptosis without direct engagement of ER-specific proteins [including Palam CDD 2015, Xu Cancer Res Treat 2014 among others]
Notably, the manuscript's central claim that CHR induces TMED3 aggregation leading to UPR activation, remains without any direct experimental evidence. Finally, clinical correla-tive analyses (TMED3 expression vs overall survival) are limited to a single, unreferenced cohort with small differences, rendering translational significance highly speculative at this stage.

Major points

Requirement for Robust Genetic Validation of TMED3 Specificity
Demonstrating the specificity of CHR's anticancer effect through TMED3 requires rigorous genetic tools beyond the modest siRNA knockdowns presented. The authors must conduct CRISPR-Cas9 or shRNA-mediated knockdown/knockout of TMED3 with multiple independent constructs, confirming efficient protein depletion at the western blot level. Furthermore, re-expression of TMED3 mutants rendered resistant to CHR binding, specifically point mutants at Arg20, Glu123, or Pro128, must be performed to show functional rescue. These experi-ments are essential to prove that TMED3 is the critical binding partner mediating CHR ac-tion rather than a secondary or off-target effect. The use of such recombinant TMED3 is mandatory to repeat fig 5J, L and N. This would actually demonstrate the CHR direct binding to TMED3 is sufficient to induce conformation changes, increased thermal stability, re-sistance to pronase degradation and allow the identification of accurate Kd.

Critical Evaluation of Target Identification Approaches and Consideration of Other Can-didate Targets
The identification of TMED3 as the sole target via a Venn diagram approach is inadequately justified. Notably, BAP18 was excluded based on in silico analyses and transcriptomic data affected by a 12-hour cycloheximide (CHX) treatment, that will mask transcriptomic changes and proteins with half-lives longer than 12 hours, are unlikely to show altered levels during such treatment. This warrants a broader exploration of other putative targets, such as BAP18, through functional assays to determine compound specificity and off-target effects.

Comprehensive Biophysical Characterization Using Recombinant TMED3 Protein
The manuscript's claims regarding direct CHR binding to TMED3 and its consequent effects on protein conformation, thermal stability, and resistance to proteolytic digestion (as pre-sented in figure 5J, L, N) must be substantiated using fully characterized, recombinant TMED3 protein, both wildtype and the aforementioned point mutants. Binding curves allow-ing accurate determination of dissociation constants (Kd) are required. Parallel testing on other ER-resident transmembrane proteins (PERK, IRE1, SEC61, GM130) is necessary as con-trols to decisively demonstrate that observed effects are TMED3-specific and not due to generalized ER protein perturbation, as commonly seen with other ER stress inducers such as DTT, thapsigargin, or tunicamycin

Temporal Resolution of ER Stress and Autophagy Response
Current data are limited to dose-responses without adequate kinetic analysis. A detailed time-course dissection of CHR-mediated activation of key ER stress sensors (IRE1, PERK) and autophagy markers (e.g., LC3 conversion) must be conducted, including assays using selec-tive pharmacological inhibitors such as 4uC8 (IRE1 inhibitor), Perk inhibitor, and bafilomycin (autophagy inhibitor). This would clearly define the sequence and interdependencies of cel-lular pathways activated by CHR and distinguish primary mechanism from downstream ef-fects.
Direct Evidence of TMED3 Aggregation
The hypothesis that CHR induces TMED3 aggregation, which triggers UPR and subsequent apoptosis, is a central mechanistic claim that lacks direct experimental support. Rigorous validation of TMED3 aggregation, via biochemical assays (native gels), advanced microscopy, or biophysical techniques, is imperative to substantiate this claim. Moderate the conclusions on TMED3 aggregate if these experiments fails to demonstrate this event.

Minor Points

Experimental Validation and Genetic Tools
Current siRNA-mediated silencing of TMED3 and ATG7 is weak and insufficient to support mechanistic conclusions. Use several independent siRNA or shRNA sequences with confirmed knockdown efficiency, validated at the protein level. Provide thorough knockdown validation for reliability.Colony formation and autophagy experiments require repetition with the above-mentioned silencing tools to confirm robustness and reproducibility
Effects of siATG7 on apoptosis are weak and inconsistent with pharmacologic inhibitors like chloroquine or wortmannin; conclusions about autophagy's role should be moderated or fur-ther supported.

Biochemical and Molecular Methods
The nature, origin, and purification details of TMED3 protein used in binding and stability (Figures 5M-N) are not provided; this should be clearly detailed in methods.
The use of wortmannin as an autophagy inhibitor is misleading since it targets PI3K; authors should justify its use or substitute with more specific autophagy modulators like bafilomycin.
Subcellular localization of TMED3 (Figure 5E) should be validated by additional markers for ER (PERK, IRE1, Sec61) and Golgi (GM130) to ensure precise compartment assignment.

Imaging and Data Presentation
Immunohistochemistry images, especially for caspase-3 cleavage, are blurry with unspecific staining ; higher quality and better characterized images are needed to substantiate apopto-sis claims.
Co-immunofluorescence figures show labeling inconsistencies; e.g., Figure S9E's text men-tions TMED3/ERp72 colocalization but the image depicts LC3/ERp72.
FACS panels are too small, poorly labeled, and hard to interpret; larger, well-annotated plots are necessary for transparency.
Supplementary table (end of main PDF file) lacks proper formatting, impeding readability; they should be reformat-ted with clear alignment, headings, and explanations.

Data Analysis and Interpretation
Differential sensitivity of pancreatic cancer lines to CHR (mild effect in Miapaca vs. T3M4) should be explained to characterize variability in response.
A systematic analysis of proteins downregulated by CHR (Figure 5 mass spectrometry) should identify how many localize to ER, Golgi, and mitochondria to determine whether CHR's ac-tions are specific or broadly affect organellar function.
All antibodies and reagents used in the study need detailed description with catalog numbers and RRIDs to ensure reproducibility and facilitate future studies.

As a service to authors, EMBO provides authors with the possibility to transfer a manuscript that one journal cannot offer to publish to another EMBO publication. The full manuscript and if applicable, reviewers reports are automatically sent to the receiving journal to allow for fast handling and a prompt decision on your manuscript. For more details of this service, and to transfer your manuscript to another EMBO title please click on Link Not Available

Please do not share this URL as it will give anyone who clicks it access to your account.

Point-by-point response

The revised sections of the manuscript are marked in blue.

Referee #1 (Remarks for Author):

The study presents chrysosplenetin (CHR) as a natural compound with potential anti-pancreatic cancer activity through induction of endoplasmic reticulum (ER) stress, mediated by aggregation of TMED3. While the experimental scope is broad (screening, cell lines, PDOs, PDXs, proteomics, docking, DARTS/CETSA/BLI), several conceptual and methodological limitations considerably reduce the translational relevance of the findings.

Major Comments

Limited in vivo efficacy :

1/ The antitumor effect of CHR in PDX models is modest: no more than ~50% tumor volume reduction at a relatively high dose (25 mg/kg);

Re: We appreciate the reviewer's critical assessment regarding the observed single-agent efficacy of CHR in our PDX models. Indeed, at a dose of 25 mg/kg, CHR monotherapy induces a tumor growth inhibition of approximately 50%, which may be viewed as a moderate effect. However, we want to contextualize this finding within the broader scope of the study and provide additional data that support the translational rationale for CHR.

It is important to note that the *in vitro* IC_{50} of CHR in pancreatic cancer cell lines ranges around 15 μM **(Fig. 1D in the original version)**. Based on the pharmacokinetic characteristics of CHR and the principle of *in vivo*-to-*in vitro* concentration conversion (PMID: 26537009; PMID: 37930506), the 25 mg/kg dose administered in mice corresponds to a concentration that is pharmacodynamically consistent with the

effective concentrations observed in cell-based assays. Thus, the antitumor effect seen *in vivo* aligns well with the compound's intrinsic potency. To further address the dose-response relationship, we conduct an additional PDX experiment evaluating CHR at 0, 25, 50, and 100 mg/kg. The results demonstrate a clear dose-dependent suppression of tumor growth, with higher doses achieving more pronounced inhibition **(Fig[PbP-R1]. 1)**. Notably, even at the highest dose tested (100 mg/kg), we observe no significant toxicity based on the comprehensive assessments, including body weight monitoring, serum biochemistry profiles and H&E staining **(Fig[PbP-R1]. 2)**. These data indicate that CHR exhibits a favorable safety window, and its antitumor activity can be enhanced by dose escalation without incurring overt systemic toxicity. In drug development, especially for aggressive malignancies like pancreatic cancer, the absence of significant toxicity at efficacious doses is often a more critical determinant of translational potential than the absolute dose magnitude itself.

Moreover, the primary objective of this study is not to present CHR as a stand-alone therapeutic agent with maximal single-agent potency, but rather to identify a novel drug-target interaction and demonstrate its potential for use in combination therapy. In summary, we have incorporated the dose-escalation data into the revised manuscript and expanded the discussion to better emphasize these points. We believe these additions strengthen the conclusion that CHR warrants further investigation as a component of rational combination regimens for pancreatic cancer.

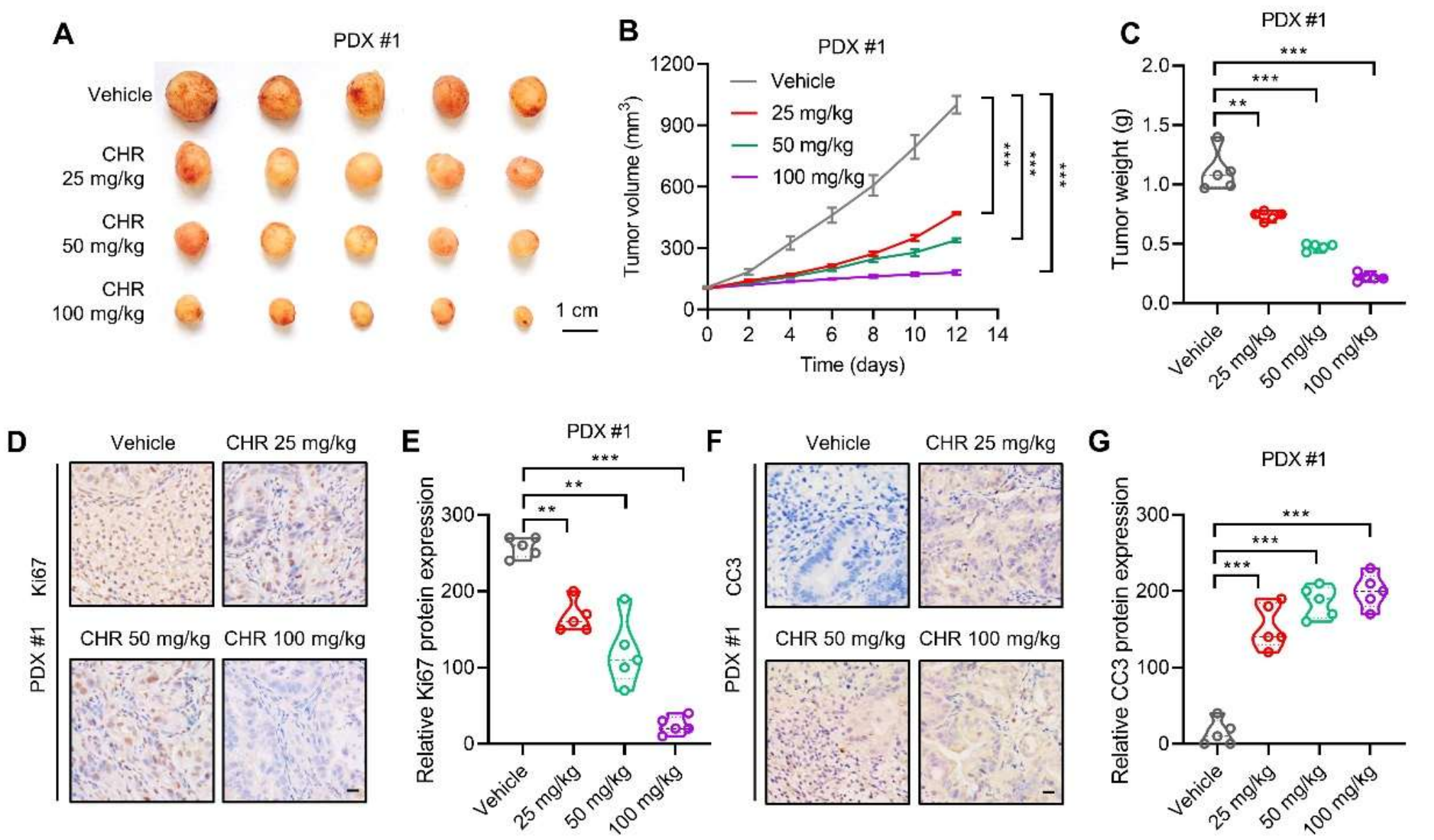

Fig[PbP-R1]. 1 (A-C) Tumor volumes (A), images (B) and weights (C) of PDX #1 xenografts treated with vehicle (n = 5) or CHR (25 mg/kg, 50 mg/kg, or 100 mg/kg, n = 5) for 12 days. **(D-G)** Representative images (D, F) and quantification (E, G) of immunohistochemical staining for Ki67 and CC3 of PDX #1 xenografts treated with vehicle or CHR, scale bar: 20 μm. Tumor volumes are presented as mean ± SEM. Other data are presented as mean ± SD, n=5; $^{**}P < 0.01$, $^{***}P < 0.001$.

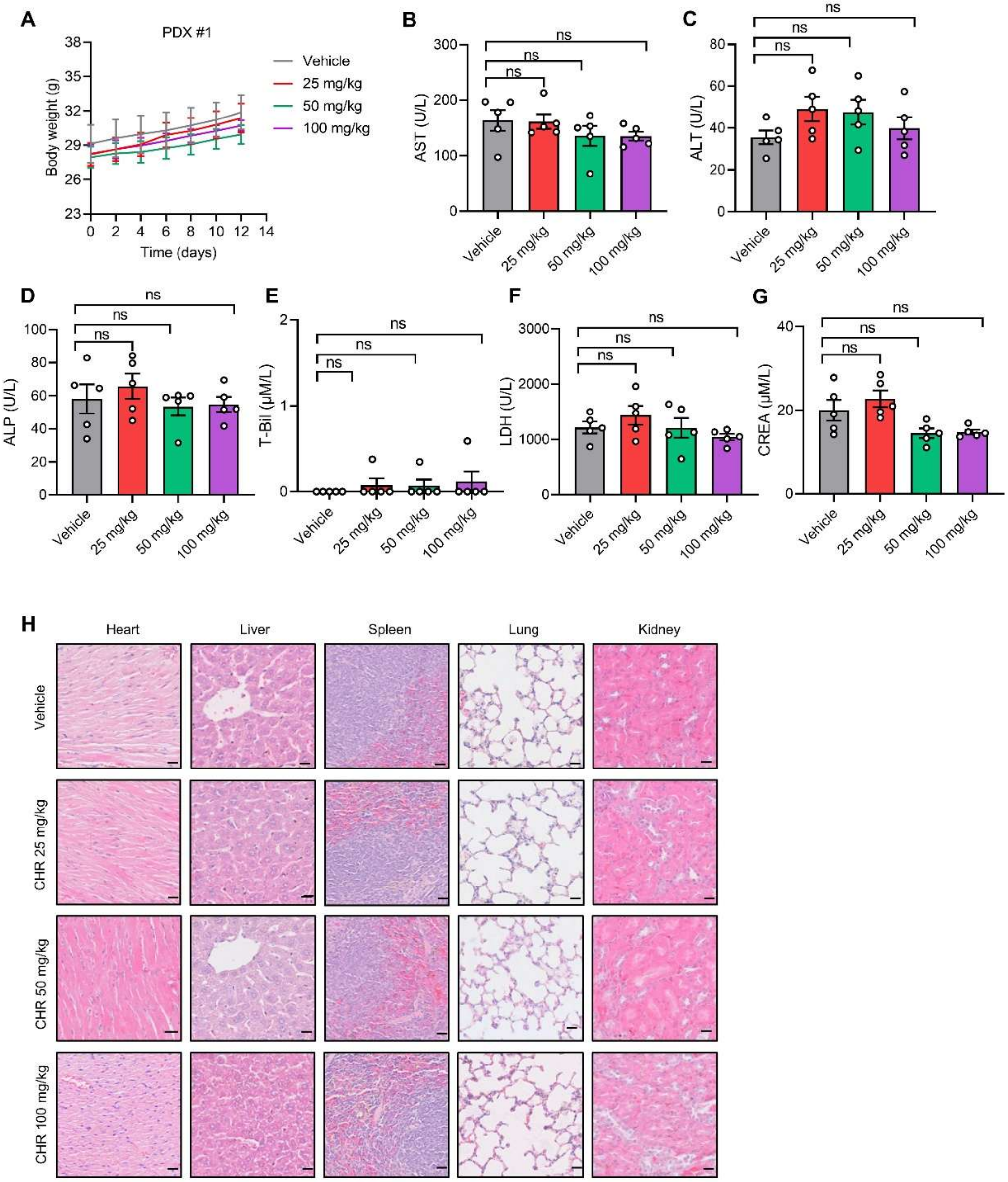

Fig[PbP-R1]. 2 (A) Body weight changes of PDX#1 xenograft mice treated with vehicle or gradient doses of CHR (25, 50, 100 mg/kg) for 12 days. **(B-G)** Serum levels of AST(B), ALT (C), ALP (D), T-Bil (E), LDH (F) , and CREA (G) in PDX #1 xenografts receiving the above-indicated CHR treatments. **(H)** Representative H&E staining images of major organs (heart, liver, spleen, lung, and kidney) from mice under different CHR dose treatments, scale bars: 20 µm. Data are presented as mean ± SEM, n=5; ns, not significant.

2/ The reported tumor volumes in PDX models show very low variability, which is unusual for these heterogeneous systems;

Re: We thank the reviewer for raising this important point regarding data presentation. The reviewer is correct that patient-derived xenograft (PDX) models can exhibit substantial biological variability. In our results depicting tumor growth over time **(Fig. 1I, 7C in the original version)**, we present the data as the mean ± SEM, which visually reduces the apparent spread of individual data points compared to showing the SD, since SEM = SD/√n. Notably, the low intra-group variability in our PDX studies is a deliberate outcome of our highly standardized protocols, designed to minimize confounding variables and enhance the reproducibility of therapeutic response assessment.

3/ Many natural and synthetic compounds have demonstrated similar or stronger preclinical efficacy but almost never advanced to the clinic.

RE: We thank the reviewer for this realistic perspective. We entirely agree that the historical gap between promising preclinical compounds and clinical success is vast, often due to unforeseen toxicity, insufficient efficacy, or unclear mechanisms, hurdles that indeed require years of collaborative effort to overcome.

The primary goal of our study is not to present CHR as a direct clinical candidate, but to provide a rigorous, mechanistic foundation for future development. We have systematically addressed two key early-stage barriers: we move beyond phenotypic screening to identify TMED3 as a novel, direct target, and delineate its role in triggering lethal ER stress; we demonstrate that targeting this pathway (via CHR) significantly enhances the antitumor efficacy of standard care (gemcitabine) in pancreatic cancer patient-derived models, outlining a clear and low-risk combinatorial clinical strategy.

Together, we view CHR not as an endpoint, but as a starting point. Its true value lies in validating TMED3/ER stress as a druggable axis in pancreatic cancer, providing a structural basis for medicinal chemists to design more potent and selective analogs, and defining a mechanistic biomarker (TMED3 aggregation/ER stress) for potential patient stratification in future trials. By openly sharing our studies, we hope to invite further investigation from the scientific community, including necessary safety assessments, structural optimization, and clinical trial design, to collectively explore the translational potential of this pathway, whether through CHR or its optimized derivatives.

Questionable in vitro potency :

1/ The IC50 values range from just above 10 μM to over 30 μM, indicating low cytotoxic efficiency;

Re: We respectfully disagree with the assessment of "low cytotoxic efficiency." In drug development, efficacy is defined by the therapeutic index (safety vs. effect), not by in vitro IC_{50} alone.

Of note, IC_{50} values in the μM range are common among clinically successful and promising agents. Widely used chemotherapy drugs such as 5-fluorouracil and oxaliplatin exhibit in vitro IC_{50} in the μM range (PMID: 41423446). Similarly, potent natural products like quercetin are active at 25-100 μM concentrations (PMID: 21610320). Thus, μM-level potency is fully compatible with significant biological and therapeutic activity. In our study, CHR demonstrates dose-dependent efficacy *in vivo* with a safety profile. Our newly provided dose-escalation data (0, 25, 50, 100 mg/kg) confirm a clear dose-dependent inhibition of tumor growth in PDX models. Critically, even at the highest dose (100 mg/kg), CHR treatment does not induce significant

toxicity, as evidenced by stable body weight and normal organ function **(Fig[PbP-R1]. 2)**. This wide therapeutic window is the key translational metric, not the absolute *in vitro* IC_{50}.

2/ For a compound with clinical aspirations, one would expect more consistent potency in the low micromolar or nanomolar range.

Re: We appreciate the reviewer's comment. However, the assumption that clinical-stage compounds must uniformly possess low nanomolar potency is not supported by clinical or pharmacological reality. Firstly, potency (IC_{50}) is distinct from efficacy and therapeutic index. Many clinically successful drugs, especially natural product-derived or cytotoxic chemotherapies, have modest IC_{50} values yet remain first-line treatments due to their *in vivo* efficacy and manageable safety profile. Examples include 5-fluorouracil, cisplatin, and paclitaxel in its early formulations, as well as potent natural leads like quercetin. The relevant benchmark is the *in vivo* therapeutic window, not the absolute dose. Secondly, CHR meets this critical benchmark. It achieves significant, dose-dependent tumor growth inhibition in PDX models at doses that show no evidence of systemic toxicity, demonstrating a favorable *in vivo* therapeutic index. Its novel, well-characterized mechanism (TMED3-mediated ER stress) and potential for combination therapy with standard regimens further enhance its therapeutic prospects. Thus, CHR's pharmacological profile is consistent with numerous effective anticancer agents.

Weak evidence for TMED3 as a target :

1/ The siRNA-mediated knockdown of TMED3 is relatively inefficient, as shown by the incomplete reduction in the western blot (Figure 6A);

RE: We thank the reviewer for this helpful observation. To provide more definitive genetic evidence, we have generated stable TMED3-knockout (TMED3-KO) cell lines via CRISPR/Cas9, screening three independent sgRNA sequences **(Fig$^{PbP\text{-}R1}$. 3)**. Then, we select the clone with the most complete knockout efficiency for all subsequent phenotypic analyses.

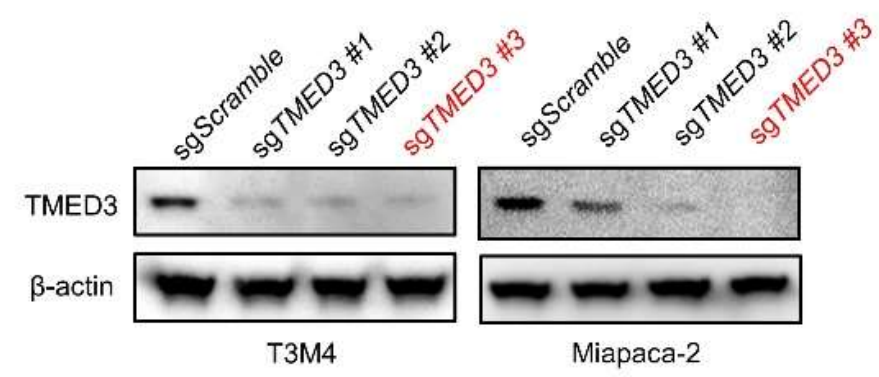

Fig$^{PbP\text{-}R1}$. 3 Immunoblotting analysis verifying TMED3 knockout efficiency in T3M4 and Miapaca-2 cells generated using three independent sgRNAs targeting *TMED3*.

2/ Even with this knockdown, the effects on ER stress markers are minimal, suggesting that TMED3 is not an essential driver of the phenotype induced by CHR;

RE: We thank the reviewer for raising this point. We agree that in our initial siRNA experiments, the partial knockdown may have limited the observable phenotypic effect. To conclusively address this, we perform the key phenotypic assays in our newly generated TMED3-KO cell lines. The results, presented in **Fig$^{PbP\text{-}R1}$. 4**, demonstrate a clear phenotypic rescue upon complete TMED3 ablation. For example, TMED3-KO cells show a marked attenuation in the upregulation of key ER stress markers and autophagy markers in response to CHR treatment. Furthermore, TMED3-KO cells are significantly more resistant to CHR-induced cytotoxicity in viability assays and exhibit a rescued clonogenic survival capacity. Flow cytometry analysis confirms a substantial reduction in CHR-induced apoptotic cell death in the TMED3-KO cells. These data from a complete genetic knockout model confirm that TMED3 is an essential mediator of the CHR-induced ER stress, autophagy, cytotoxicity, and apoptotic phenotypes. We

have updated the relevant sections of the manuscript with these new data.

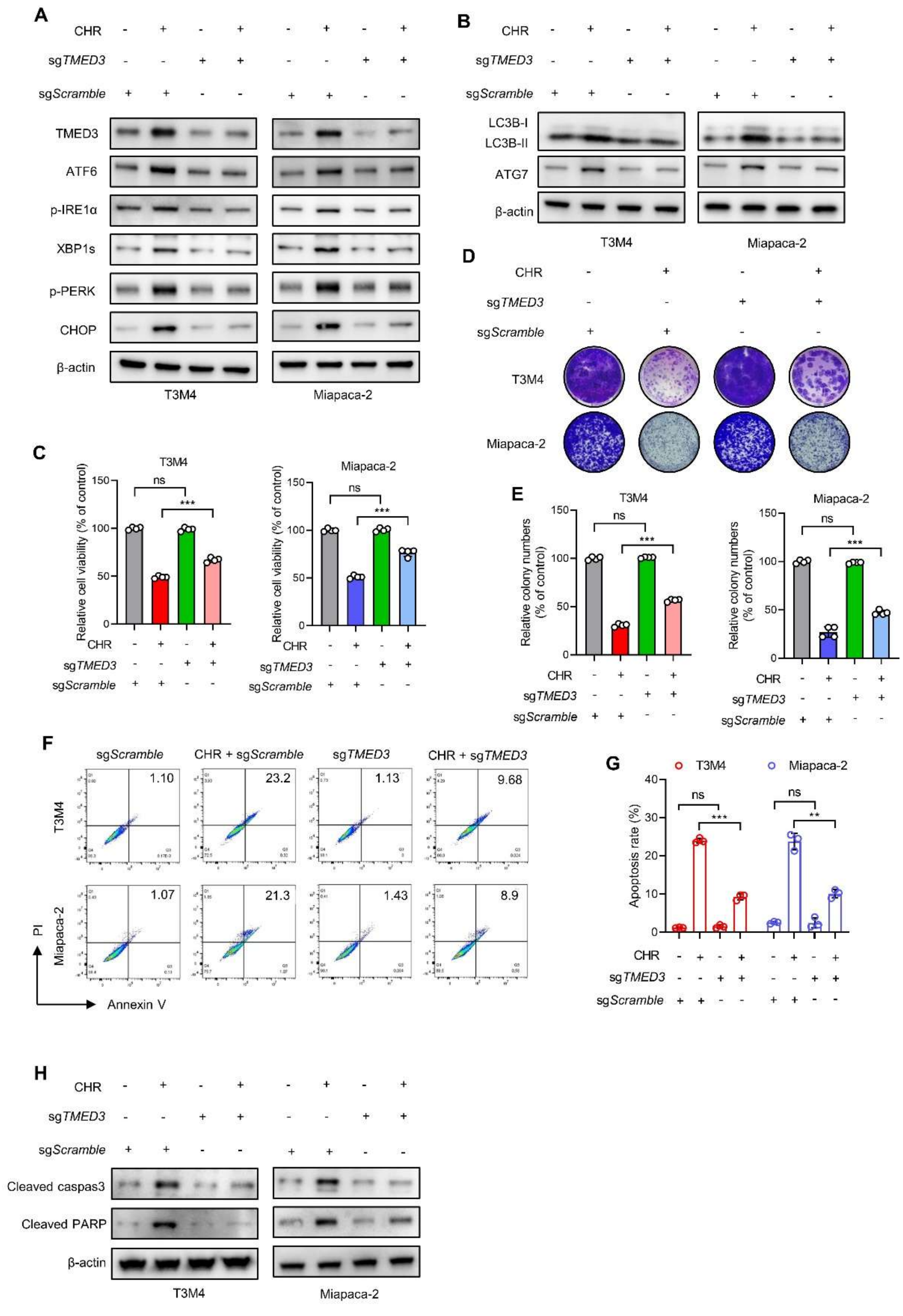

Fig[PbP-R1]. 4 (A-B) Immunoblotting analysis of ER stress markers (A) and autophagy markers (B) in wild-type and TMED3-KO T3M4 and Miapaca-2 cells treated with CHR (10 μM) for 48 h. **(C)** Cell viability of wild-type and TMED3-KO T3M4 and Miapaca-2 cells treated with CHR for 48 h. **(D-E)** Representative images (D) and quantification of colony numbers (E) of wild-type and

TMED3-KO T3M4 and Miapaca-2 cells treated with CHR. **(F-G)** Flow cytometric analysis (F) and quantification (G) of apoptosis rate in wild-type and TMED3-KO T3M4 and Miapaca-2 cells treated with CHR for 48 h. **(H)** Immunoblotting analysis of CC3 and cleaved PARP in wild-type and TMED3-KO T3M4 and Miapaca-2 cells treated with CHR for 48 h. Data are presented as mean ± SD, n=3/4; $^{**}P < 0.01$, $^{***}P < 0.001$, ns, no significant.

3/ If TMED3 were truly the critical target, its genetic inhibition should mimic (or at least approximate) the biological effects of CHR, which is not the case;

RE: Thank you very much for your comment. As detailed in our response to the related prior comment, we have now performed genetic validation using CRISPR/Cas9-mediated TMED3 knockout cells. The data confirm that complete ablation of TMED3 significantly attenuates the key biological effects of CHR, including UPR and autophagy activation, cytotoxicity, and apoptosis, which robustly supports TMED3's role as a critical mediator of CHR's action.

4/ More studies with the siTMED3 must be performed to validate TMED3 as an essential target.

RE: Thank you. Please refer to the previous reply.

Mechanistic shortcomings :

5/ The authors propose that CHR stabilizes TMED3 and triggers its aggregation in the ER, but they do not show whether CHR destabilizes or alters TMED3 turnover (e.g., ubiquitination, proteasomal degradation);

RE: We thank the reviewer for this insightful suggestion. In response, we have performed new experiments to investigate this. As shown in **Fig$^{\text{PbP-R1}}$. 5**, co-treatment with the proteasome inhibitor MG132 does not result in a further accumulation of TMED3 beyond the level induced by CHR alone. Consistently, immunoprecipitation

analysis shows that CHR treatment does not significantly alter the poly-ubiquitination status of TMED3. These findings indicate that the CHR-induced stabilization and subsequent aggregation of TMED3 in the ER are not primarily mediated by inhibition of its ubiquitin-mediated proteasomal degradation. We have integrated these results and their interpretation into the revised manuscript.

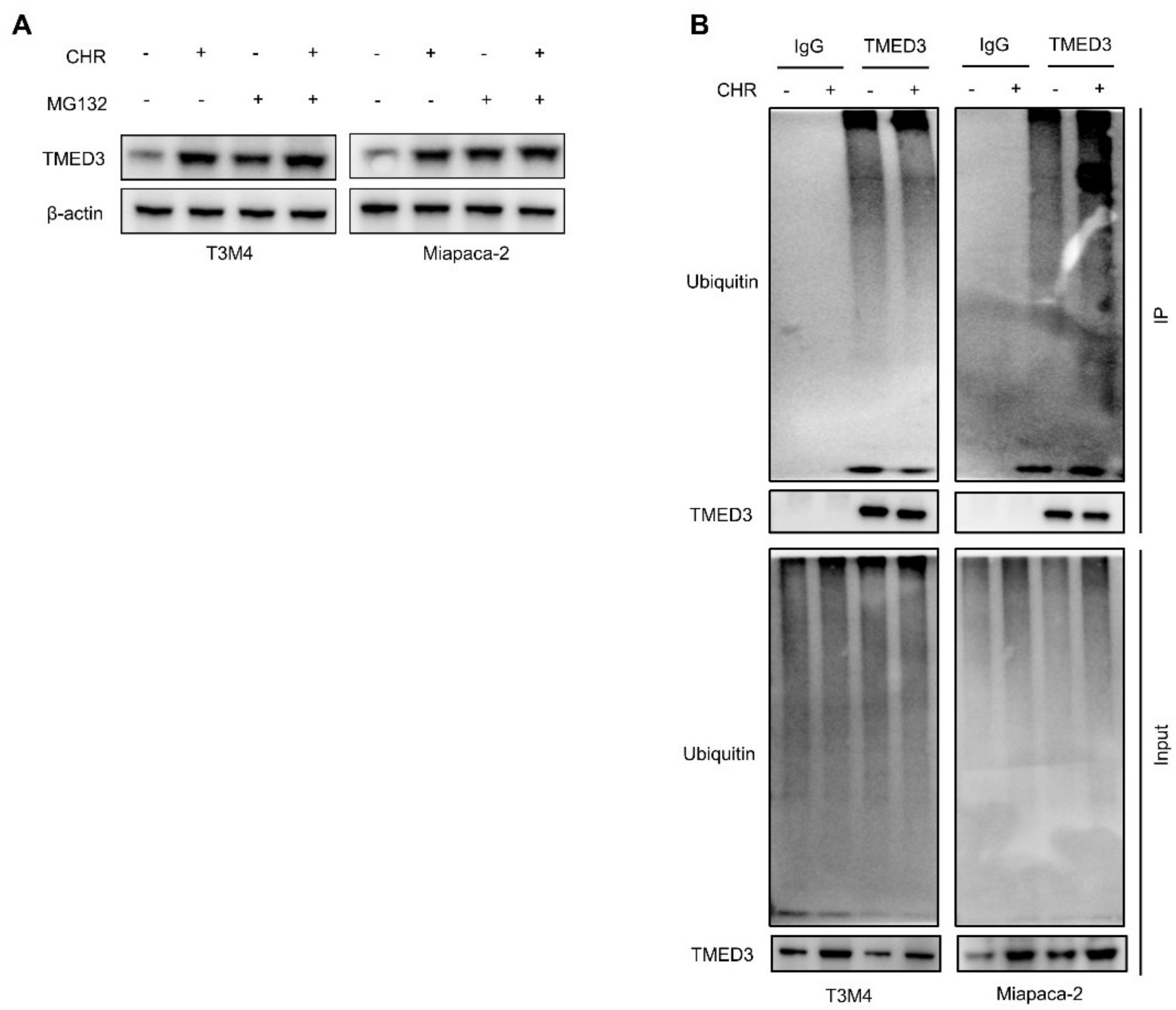

Fig^PbP-R1^. 5 (A) Immunoblotting analysis of TMED3 in T3M4 and Miapaca-2 cells pretreated with MG132 (5 μM) for 24 h, followed by CHR treatment for 48 h. **(B)** Co-immunoprecipitation analysis verifying the interaction between TMED3 and ubiquitin in T3M4 and Miapaca-2 cells following 48 h CHR treatment.

6/ The observed activation of UPR and apoptosis is consistent with what has already been reported for many other flavonoids and natural products. Therefore, the mechanism is not conceptually novel, but rather a reiteration of a well-documented phenomenon.

RE: We thank the reviewer for raising this point. We agree that the induction of ER

stress/UPR, apoptosis, and autophagy represents a common phenotypic outcome for many anticancer agents, including various flavonoids. Indeed, these are conserved cellular response pathways rather than novel biological discoveries.

The conceptual novelty of our work lies not in the observation of these downstream phenotypes, but in the identification of the precise molecular target and initiating event that triggers them. While many compounds may converge on similar cellular outcomes, their specific molecular targets and upstream mechanisms often remain elusive, which critically limits mechanistic understanding and rational drug development. In this study, we have gone beyond phenotypic characterization to identify TMED3 as a direct binding target of CHR using an integrated approach (BLI, CETSA, DARTS, molecular docking). To further confirm this result, we provide new genetic evidence in this revised manuscript, through mutagenesis of key TMED3 residues (*e.g.*, Arg20, Glu123, Pro128). Mutational analysis of critical TMED3 residues, especially Arg20, markedly disrupts CHR interaction and subsequent cell death progression **(Fig[PbP-R1]. 6-7)**. These data strongly reinforce the specificity of the CHR-TMED3 interaction. To our knowledge, this is the first report identifying TMED3 as a druggable target whose ligand-induced aggregation triggers lethal ER stress in pancreatic cancer cells. This mechanism distinguishes CHR from other flavonoids and provides a novel, target-driven rationale for its anti-tumor activity. We have clarified this point in the revised discussion to better highlight the mechanistic advance beyond the well-documented downstream phenotypes.

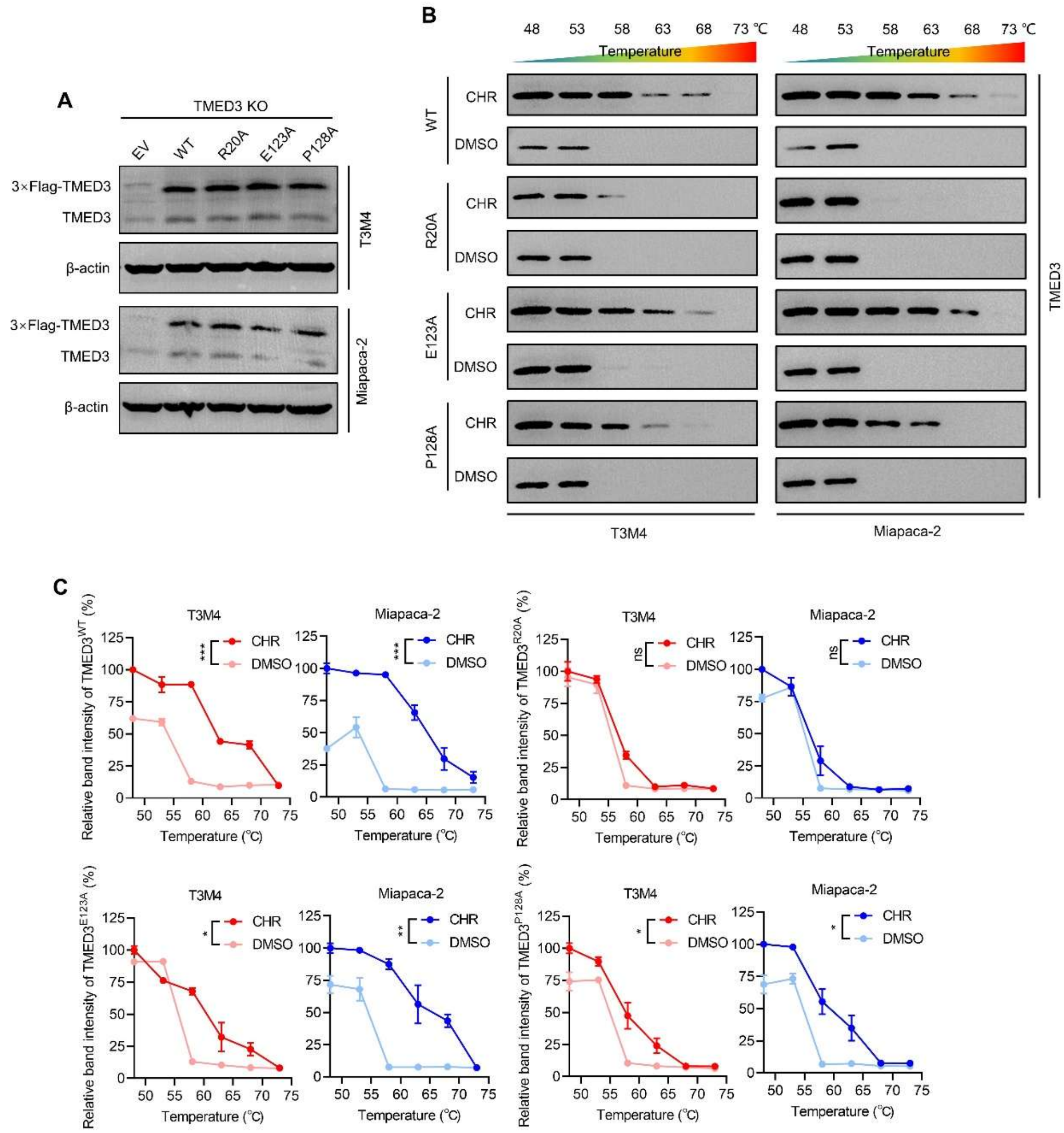

Fig[PbP-R1]. 6 (A) Immunoblotting analysis verifying TMED3 expression in TMED3-KO T3M4 and Miapaca-2 cells (generated with sgRNA#3) upon re-expression of wild-type TMED3 or TMED3 point mutants (R20A, E123A, P128A). **(B-C)** Immunoblotting analysis (B) and quantification of relative band intensity (C) of CETSA for CHR binding to wild-type TMED3, TMED3 R20A, E123A and P128A mutant proteins in TMED3-KO cells. Data are presented as mean ± SD, n=3; $^{*}P < 0.05$, $^{**}P < 0.01$, $^{***}P < 0.001$, ns, no significant.

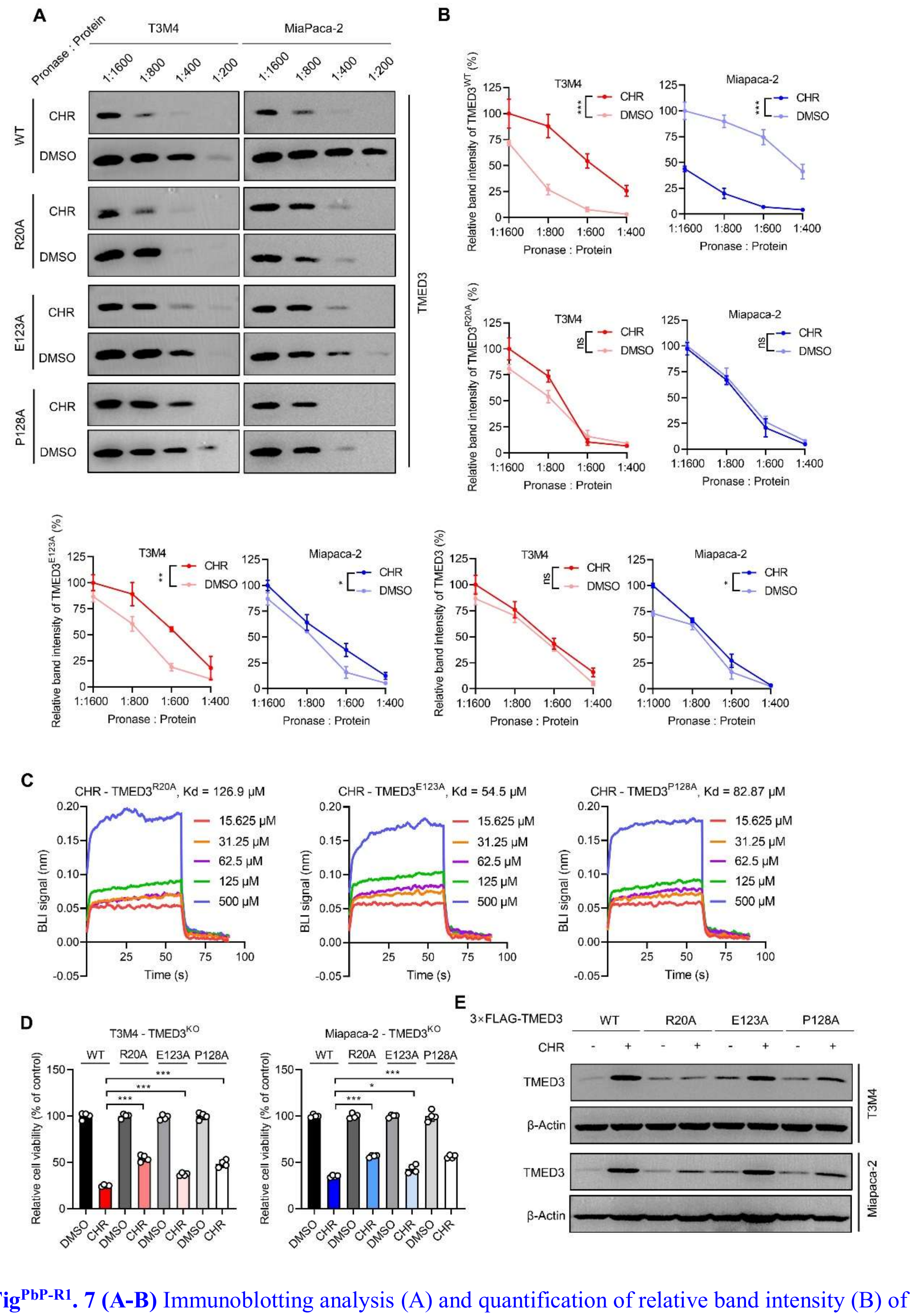

Fig[PbP-R1]. 7 (A-B) Immunoblotting analysis (A) and quantification of relative band intensity (B) of DARTS for CHR binding to wild-type TMED3, TMED3 R20A, E123A and P128A mutant proteins in TMED3-KO cells. **(C)** Bio-layer interferometry sensorgrams indicating direct binding of CHR to TMED3 R20A, E123A and P128A mutant proteins with corresponding Kd values. **(D)** Cell viability of wild-type, R20A, E123A, and P128A mutant-reconstituted TMED3-KO T3M4 and Miapaca-2 cells after 48 h of CHR (20 μM) or DMSO treatment. **(E)** Immunoblotting for TMED3 in TMED3-

KO T3M4 and Miapaca-2 cells re-expressing wild-type TMED3 or its mutants (R20A, E123A, P128A) after 48 h of CHR or DMSO treatment. Data are presented as mean ± SD, n=3/4; $^{*}P < 0.05$, $^{**}P < 0.01$, $^{***}P < 0.001$, ns, no significant.

Overstated clinical relevance:

1/ CHR's activity is neither exceptional in vitro nor in vivo;

RE: We thank the reviewer for their rigorous evaluation. We respectfully disagree that CHR's activity is not exceptional. Its preclinical profile is not defined by a single parameter but by a compelling combination of attributes that collectively support its translational promise. Importantly, we emphasize that CHR's anti-cancer activity is robust and entirely in line with established standards in oncology drug discovery. As a natural product lead, its *in vitro* potency (IC_{50} in the μM range) and *in vivo* efficacy are fully comparable to, and in some cases, surpass the preclinical profiles of several clinically successful chemotherapies, such as 5-fluorouracil and oxaliplatin, which are well-known to exhibit μM-level *in vitro* activity (PMID: 41423446). Moreover, CHR does not need to be the most potent compound ever discovered to be exceptional. It is a well-characterized, target-driven agent with validated efficacy, a good safety profile, and a clear route to clinical application. This combination makes it a highly promising candidate for further development.

2/ The reported binding affinity (Kd ~16 μM) is relatively weak, and the relationship between TMED3 expression and patient survival in pancreatic cancer is not coherently addressed;

RE: Thank you. We address each point below with new data that strengthens our conclusions.

Firstly, we agree that a Kd in the μM range may be considered moderate in

absolute terms. However, it is entirely consistent with the functional activity and cellular potency of CHR. Many successful natural product-derived drugs and bioactive compounds (*e.g.,* metformin, Ainsliadimer A) exhibit effective concentrations and target affinities in this range (PMID: 35197629; PMID: 36889312). The significance of the interaction is validated by its functional consequences. Direct binding is confirmed across multiple orthogonal methods (CETSA, DARTS, BLI). Furthermore, critical residues for binding are identified in this revision **(Fig$^{\text{PbP-R1}}$. 6-7)**. Together, the moderate binding affinity is functionally relevant and mechanistically validated by mutagenesis studies.

Secondly, the survival analysis in the initial submission is based on clinical data from 16 databases (PMID: 37260110). To address this more comprehensively, we have now performed a validation study using an independent tissue microarray cohort of pancreatic cancer patients. The analysis also confirms that low TMED3 expression is significantly associated with worse overall survival **(Fig$^{\text{PbP-R1}}$. 8)**.

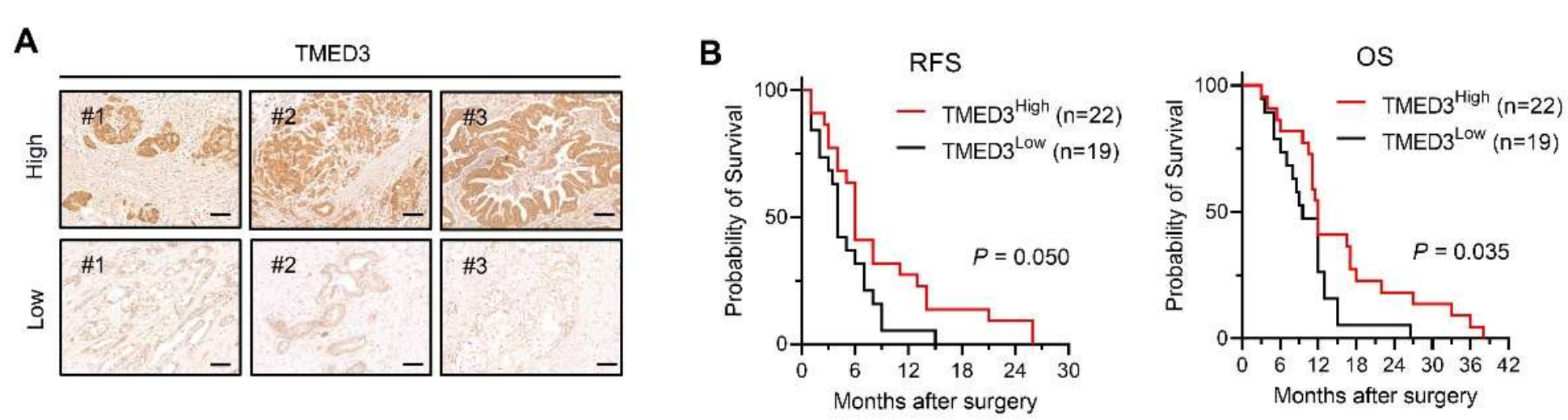

Fig$^{\text{PbP-R1}}$. 8 (A) Representative images of immunohistochemical staining for TMED3 in PDAC patient tumor tissueswith three representative cases each for high- and low-TMED3 expression groups. Scale bar: 100 µm. **(B)** Kaplan-Meier analysis of PDAC patients based on TMED3 expression levels derived from tissue microarray (A).

3/ Although no major toxicity was detected in mice, this does not offset the limited antitumor efficacy;

RE: Thank you again. We respectfully argue that the preclinical therapeutic potential of

a compound is fundamentally defined by its therapeutic window, the balance between efficacy and tolerability, rather than by either metric in isolation. Our study demonstrates that CHR exhibits a favorable and exceptionally wide therapeutic window, which is a key translational strength. Notably, in our revised manuscript, we include a dose-response experiment (25, 50, and 100 mg/kg) in PDX models. The results shows a clear, dose-dependent increase in anti-tumor activity. At the highest dose tested (100 mg/kg), CHR monotherapy achieves highly significant tumor growth suppression. Crucially, no dose-limiting toxicity is observed even at 100 mg/kg, as evidenced by stable body weight, normal serum biochemistry, and unremarkable histopathology of major organs **(Fig[PbP-R1]. 1-2)**.

4/ Overall, the clinical interest of CHR as a stand-alone or combination therapy is minimal.

RE: Thank you for your comments. We respectfully but firmly disagree with the reviewer's broad conclusion regarding the clinical potential of CHR. As we have repeatedly mentioned before, CHR represents more than a compound with standalone activity. It is a targeted therapy candidate with a novel mechanism, a ready-made and rational combination strategy (with gemcitabine), and a safety profile that supports this approach. This integrated profile provides a strong, mechanism-driven foundation for clinical investigation, distinguishing it from agents with efficacy but undefined targets or narrow therapeutic indices.

Final Conclusion

This manuscript provides a wide set of experimental data, but the results do not convincingly support TMED3 as a solid therapeutic target or CHR as a viable clinical

candidate. The proposed mechanism (ER stress-UPR-apoptosis) is already well established and lacks true novelty. Given the modest in vivo efficacy, limited in vitro potency, weak evidence for TMED3 as the critical mediator, and the existence of numerous compounds with similar preclinical effects that never reached the clinic, the translational value of this study is very limited.

RE: We thank the reviewer for the comprehensive evaluation. We respectfully maintain that the study provides a novel and translationally relevant contribution. The novelty lies in identifying TMED3 as the direct molecular target, a mechanism distinct from other ER stress inducers. Our functional validation, now strengthened by CRISPR knockout and binding-site mutagenesis, solidly supports TMED3 as a critical mediator. While CHR's *in vitro* IC_{50} is in the μM range, common among clinically used drugs, its true strength is its excellent *in vivo* therapeutic index (dose-dependent efficacy with no observed toxicity) and proven combination effect with gemcitabine in PDX models. This combination of a novel target, safety, and clear combinatorial strategy provides a concrete, de-risked path for clinical translation that many preclinical candidates lack.

Referee #2 (Remarks for Author):

In this manuscript, Zhang et al. investigate chrysosplenetin (CHR) as a potential therapeutic agent in pancreatic cancer (PC), proposing TMED3 as its direct target and linking TMED3 aggregation to ER stress, UPR activation, and apoptosis. The study employs a broad range of experimental approaches, and the identification of TMED3 as a possible novel target in PC is of potential interest. However, several aspects of the mechanistic interpretation remain insufficiently developed, and the manuscript would benefit from substantial revision before it can be considered for publication.

Major Concerns:

1. A central concern is the mechanistic evidence. The authors suggest that CHR induces the UPR through aggregation of TMED3, but the precise mechanism by which TMED3, an ER-localized protein, mediates CHR-induced ER stress and triggers UPR signaling and cell death is not clearly demonstrated. Closely related to this, the causal link between CHR binding to TMED3 and the selective activation of UPR pathways is not fully established.

RE: We thank the reviewer for this insightful comment. In this revision, we have conducted several key experiments to directly address this causality and deepen the mechanistic understanding.

Firstly, complete loss of TMED3 profoundly attenuates CHR-induced UPR activation and apoptosis **(Fig$^{PbP\text{-}R2}$. 1)**. Our new data with TMED3 point mutants (Arg20, Glu123, Pro128) demonstrate that disrupting the CHR-binding interface significantly blunts CHR-induced cell death **(Fig$^{PbP\text{-}R2}$. 2-3)**. This establishes a direct molecular and functional link between specific binding to TMED3 and the downstream apoptotic phenotypes.

To investigate the mechanistic link between CHR-induced TMED3 aggregation

and ER stress, we performed an unbiased TMED3 interactome analysis following CHR treatment in Miapaca-2 and T3M4 cells. The analysis was conducted under stringent conditions for each line, comparing an experimental group (CHR + anti-TMED3) against baseline (DMSO + anti-TMED3) and negative (DMSO + IgG) controls. High-confidence interactors were identified by applying dual filters of Protein FDR Confidence (High) and an experiment-wide q-value ≤ 0.01. Furthermore, we systematically excluded common contaminants (*e.g.*, proteins with "CON_" accession, keratins, histones, ribosomal proteins) and any protein detected in the IgG control, thereby defining cell line-specific sets of putative TMED3 interactors **(Fig[PbP-R2]. 4)**.

To identify the most robust changes, we focused on the common differential interactors between the two cell lines **(Fig[PbP-R2]. 4)**. This comparative analysis revealed a marked rewiring of the TMED3 interaction network upon CHR treatment. Specifically, associations with proteins involved in cytoskeletal dynamics and stress signaling (*e.g.*, EZR, S100A9) were consistently enhanced, while interactions with factors implicated in RNA metabolism and gene expression regulation (*e.g.*, SRSF1, ZMAT2) were diminished in both models.

While a full characterization of these interactions is beyond the scope of this study, we propose that CHR-induced TMED3 oligomers act as a scaffold. This scaffold actively recruits cytoskeletal and stress-responder proteins (*e.g.*, EZR, S100A9) to the ER-Golgi interface, while concurrently displacing factors involved in mRNA processing and adaptive transcription (*e.g.*, SRSF1, ZMAT2). We hypothesize that this rewiring initiates ER stress through two primary mechanisms: (1) by disrupting vesicular trafficking via aberrant cytoskeletal engagement, and (2) by creating a local pro-apoptotic signaling environment that amplifies the UPR. Furthermore, the loss of adaptive transcription factors likely cripples the cell's compensatory response,

collectively driving irreversible ER stress and cell death.

We have incorporated these points into the revised discussion, clarifying that CHR binding to TMED3 initiates a cascade likely involving the disruption of its native interactome and ER homeostasis functions, which culminates in UPR activation. This refined model strengthens the causal narrative and provides a clear direction for future mechanistic dissection.

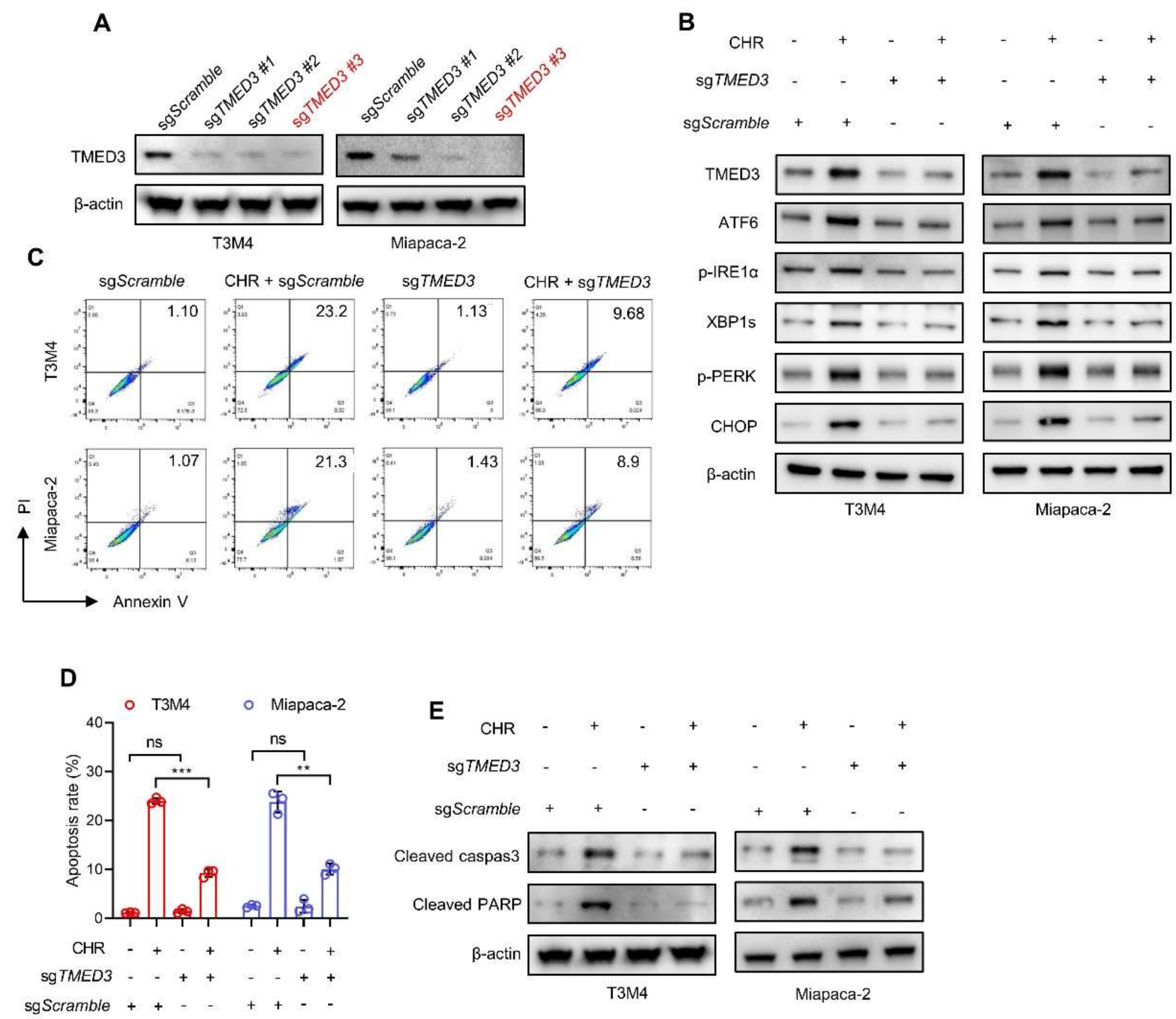

Fig[PbP-R2]. 1 (A) Immunoblotting analysis verifying TMED3 knockout efficiency in T3M4 and Miapaca-2 cells generated using three independent sgRNAs targeting *TMED3*. **(B)** Immunoblotting analysis of ER stress markers in wild-type and TMED3-KO T3M4 and Miapaca-2 cells treated with CHR (10 μM) for 48 h. **(C-D)** Flow cytometric analysis (C) and quantification (D) of apoptosis rate in wild-type and TMED3-KO T3M4 and Miapaca-2 cells treated with CHR for 48 h. **(E)** Immunoblotting analysis of CC3 and cleaved PARP in wild-type and TMED3-KO T3M4 and Miapaca-2 cells treated with CHR for 48 h.

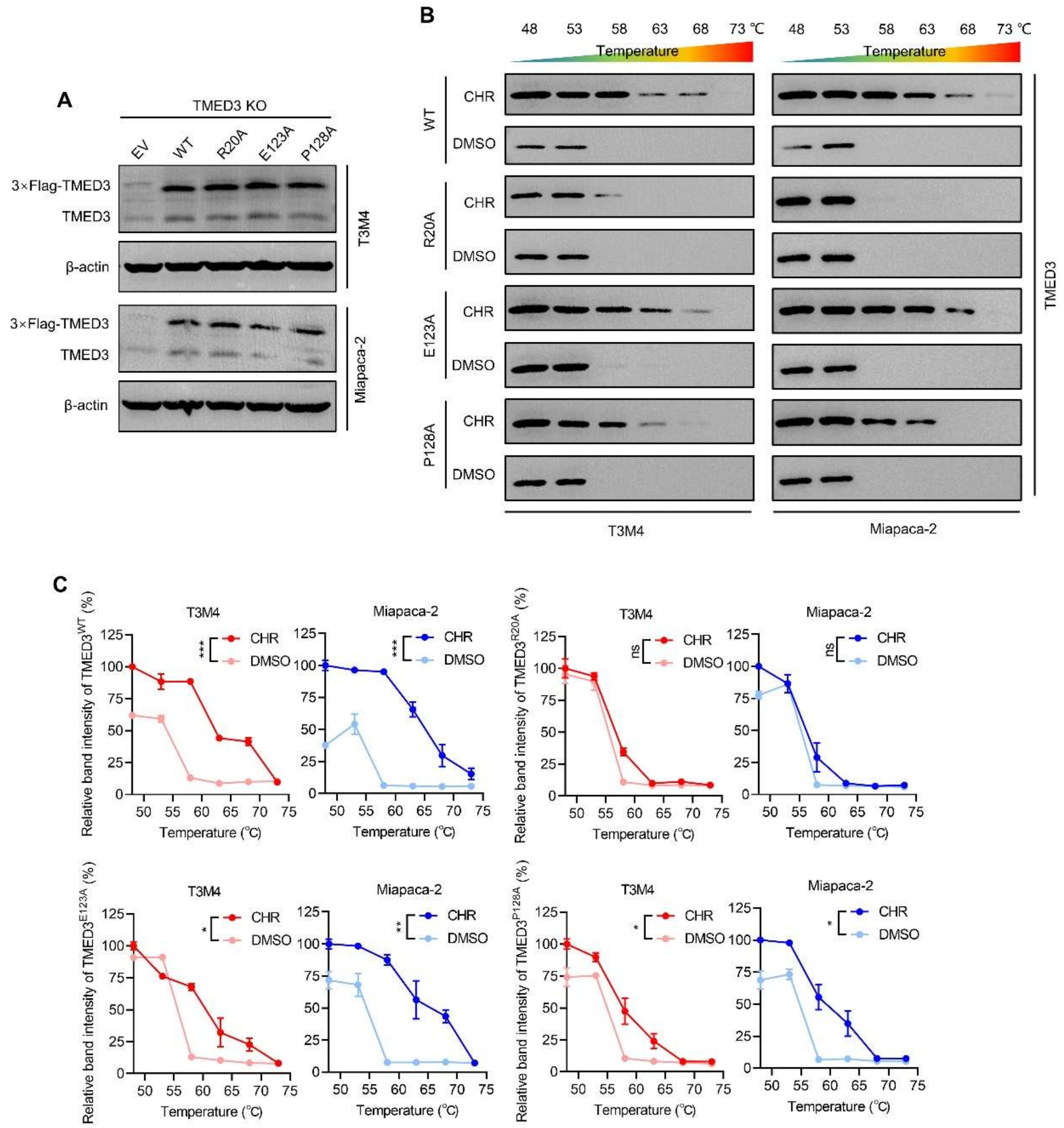

Fig[PbP-R2]. 2 (A) Immunoblotting analysis verifying TMED3 expression in TMED3-KO T3M4 and Miapaca-2 cells (generated with sgRNA#3) upon re-expression of wild-type TMED3 or TMED3 point mutants (R20A, E123A, P128A). **(B-C)** Immunoblotting analysis (B) and quantification of relative band intensity (C) of CETSA for CHR binding to wild-type TMED3, TMED3 R20A, E123A and P128A mutant proteins in TMED3-KO cells. Data are presented as mean ± SD, n=3; $^{*}P < 0.05$, $^{**}P < 0.01$, $^{***}P < 0.001$, ns, no significant.

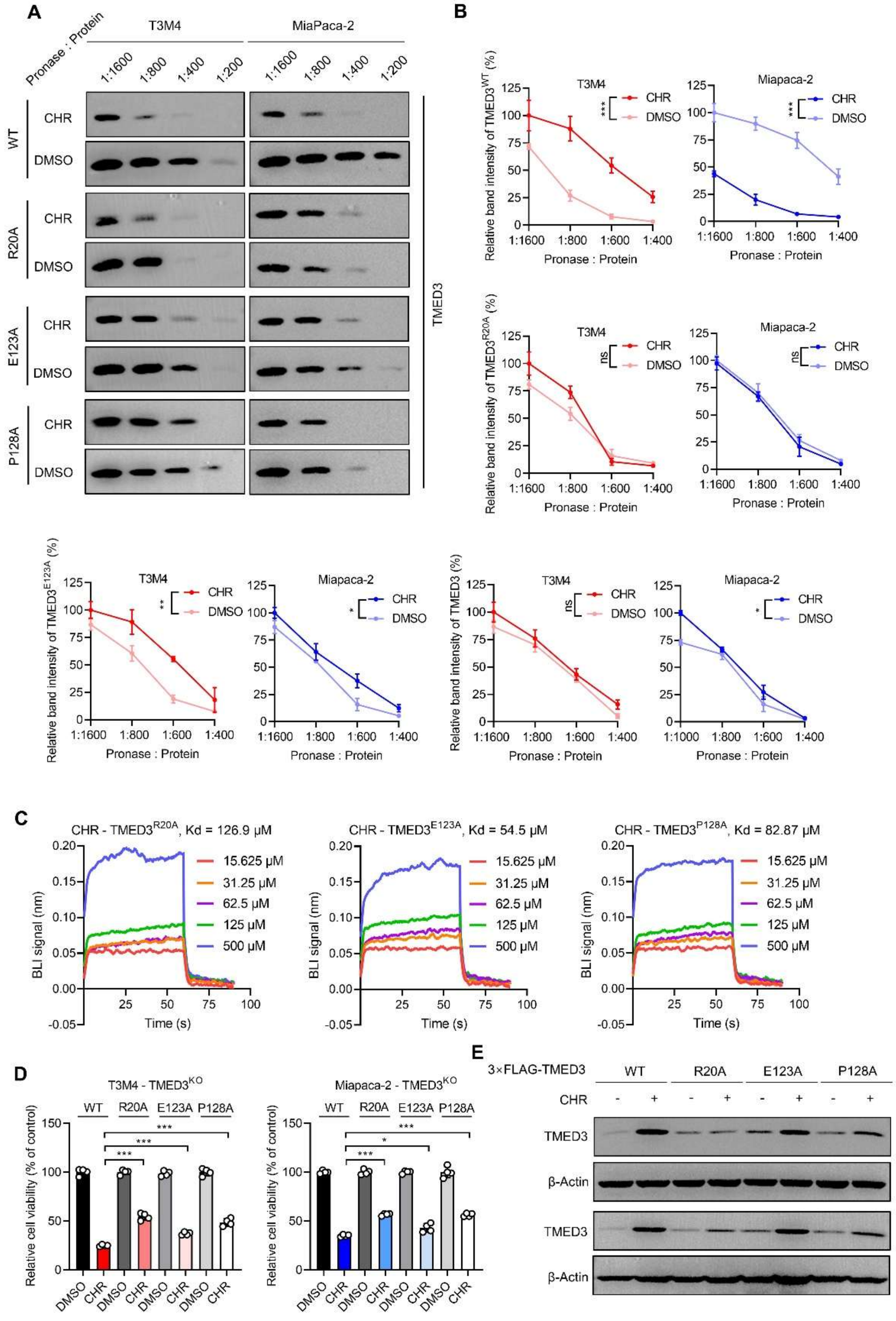

Fig[PbP-R2]. 3 (A-B) Immunoblotting analysis (A) and quantification of relative band intensity (B) of DARTS for CHR binding to wild-type TMED3, TMED3 R20A, E123A and P128A mutant proteins in TMED3-KO cells. **(C)** Bio-layer interferometry sensorgrams indicating direct binding of CHR to TMED3 R20A, E123A and P128A mutant proteins with corresponding Kd values. **(D)** Cell viability of wild-type, R20A, E123A, and P128A mutant-reconstituted TMED3-KO T3M4 and Miapaca-2 cells after 48 h of CHR (20 μM) or DMSO treatment. **(E)** Immunoblotting for TMED3 in TMED3-

KO T3M4 and Miapaca-2 cells re-expressing wild-type TMED3 or its mutants (R20A, E123A, P128A) after 48 h of CHR or DMSO treatment. Data are presented as mean ± SD, n=3/4; *$P < 0.05$, **$P < 0.01$, ***$P < 0.001$, ns, no significant.

Figure for reviewers removed

2. The analysis of UPR signaling is incomplete: while the IRE1 and PERK branches are examined, the ATF6 pathway is not addressed, leaving the overall picture of UPR activation only partially resolved.

RE: Thank you for highlighting this important aspect of UPR signaling. In the revised manuscript, we have now included analysis of the ATF6 branch, providing a more comprehensive view of CHR-induced UPR activation. As shown in **Fig[PbP-R2]. 5**, CHR treatment induces a dose-dependent upregulation of ATF6 protein expression in pancreatic cancer cells. Furthermore, this upregulation is significantly attenuated by co-treatment with the ER stress inhibitor 4-PBA and is markedly reduced upon genetic

knockdown of TMED3. These new data demonstrate that CHR activates all three major branches of the UPR (PERK, IRE1α, and ATF6) in a TMED3-dependent manner. We have integrated these findings into the relevant results section and updated the corresponding figure legends.

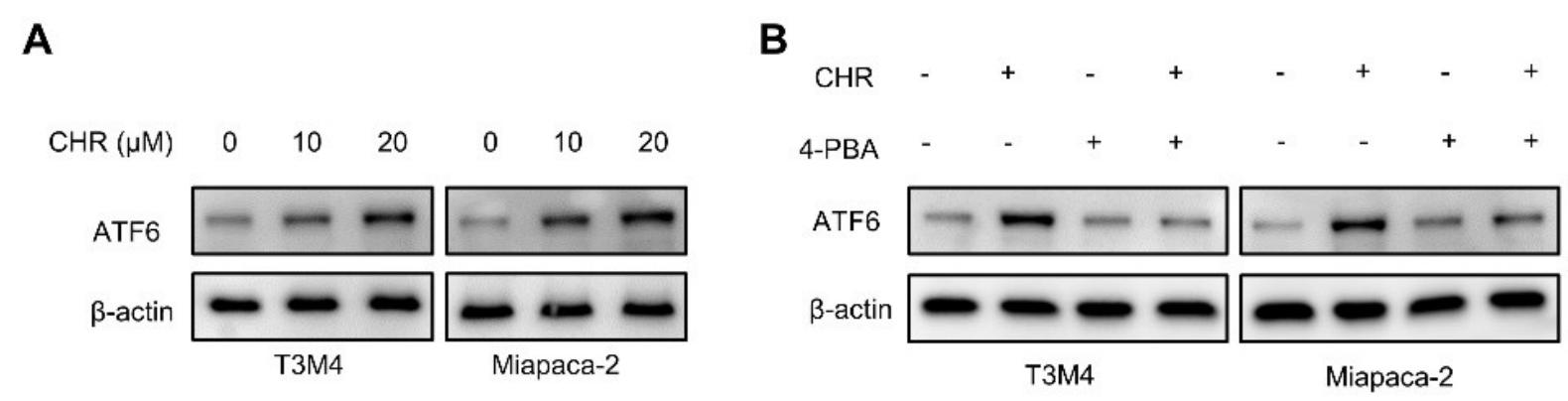

Fig[PbP-R2]. 5 **(A)** Immunoblotting analysis of ATF6 in T3M4 and Miapaca-2 cells treated with indicated concentrations of CHR for 48 h. **(B)** Immunoblotting analysis of ATF6 in T3M4 and Miapaca-2 cells treated with CHR (10 μM) in the presence or absence of 4-PBA (2 mM) for 48 h.

3. The treatment of IRE1α, as one of the key mediators of the UPR, requires further detail. Demonstration of IRE1α phosphorylation alone is not sufficient to establish a functional role, and assays such as XBP1 splicing or RIDD activity would strengthen the conclusions.

RE: Thank you very much for the good suggestion. In response, we have performed additional experiments to assess IRE1α's downstream signaling activity. As shown in **Fig[PbP-R2]. 6,** CHR treatment leads to a concentration-dependent increase in the spliced, active form of XBP1 (XBP1s) at the protein level, confirming activation of the canonical IRE1α-XBP1 signaling axis. Furthermore, genetic knockdown of *IRE1α* (siRNA) or pharmacological inhibition of IRE1α's RNase activity using 4μ8C effectively attenuated the CHR-induced upregulation of XBP1s. This establishes a direct functional link between CHR-induced IRE1α phosphorylation and its downstream transcriptional output. We have incorporated these findings into the revised manuscript and updated the corresponding figure legends.

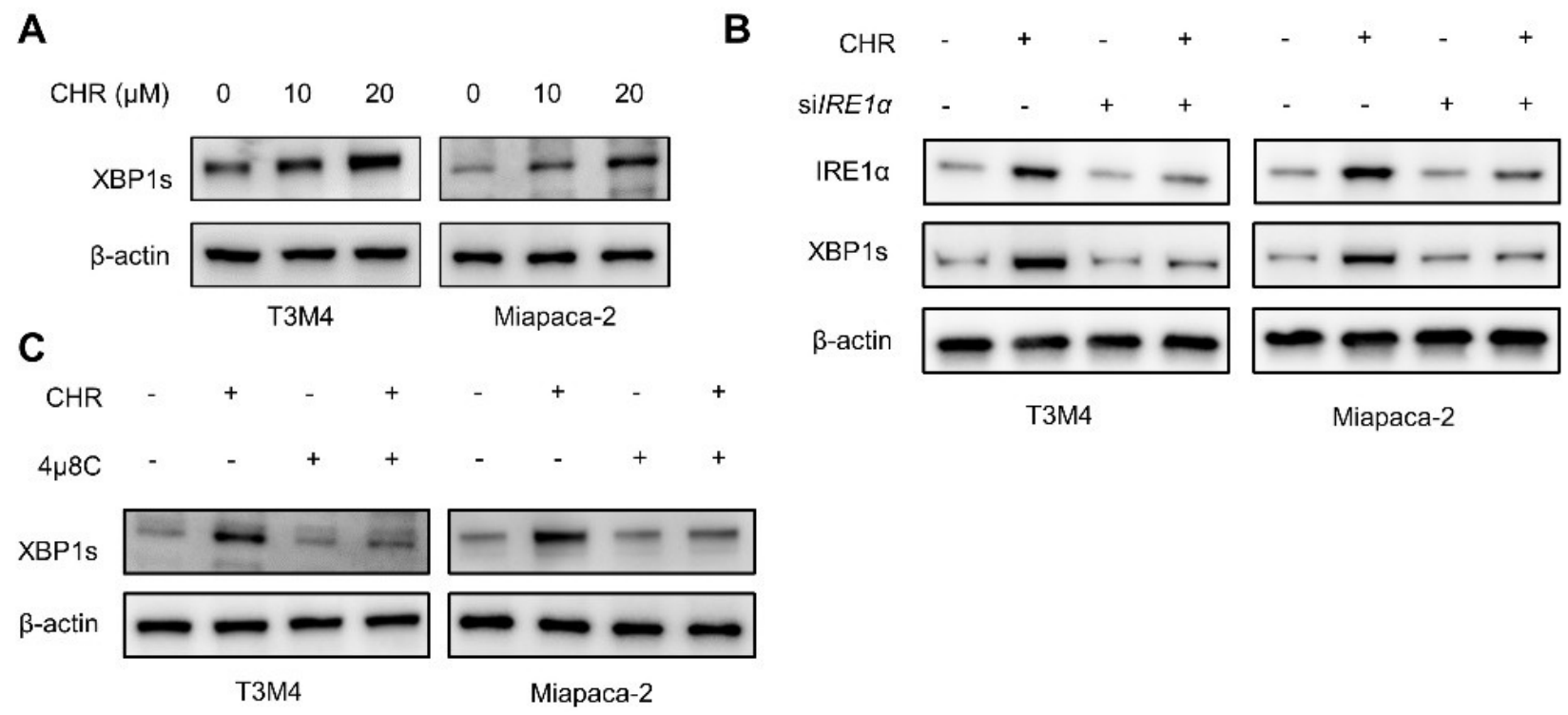

Fig[PbP-R2]. 6 (A) Immunoblotting analysis of XBP1s in T3M4 and Miapaca-2 cells treated with indicated concentrations of CHR for 48 h. **(B)** Immunoblotting analysis of IRE1α and XBP1s in T3M4 and Miapaca-2 cells treated with CHR (10 μM) for 48 h after transfected with si*IRE1α* or si*Scramble*. **(C)** Immunoblotting analysis of XBP1s in T3M4 and Miapaca-2 cells treated with CHR in the presence or absence of 4μ8C (5 μM) for 48 h.

4. The interpretation of autophagy also requires clarification. The authors conclude that autophagy is cytoprotective in this setting, but it remains unclear whether this response is mediated by IRE1α signaling or reflects a more general stress response.

RE: We thank the reviewer for raising this important point. To clarify whether the observed cytoprotective autophagy is specifically linked to the IRE1α branch of the UPR, we performed the following experiments. As shown in **Fig[PbP-R2]. 7**, we find that using siRNA against *IRE1α* or the specific inhibitor 4μ8C significantly attenuates the CHR-induced upregulation of key autophagy markers. These data indicate that the cytoprotective autophagy triggered by CHR is not merely a general stress response, but is mechanistically coupled to the activation of the IRE1α signaling branch of the UPR. We have integrated these results and their interpretation into the revised manuscript.

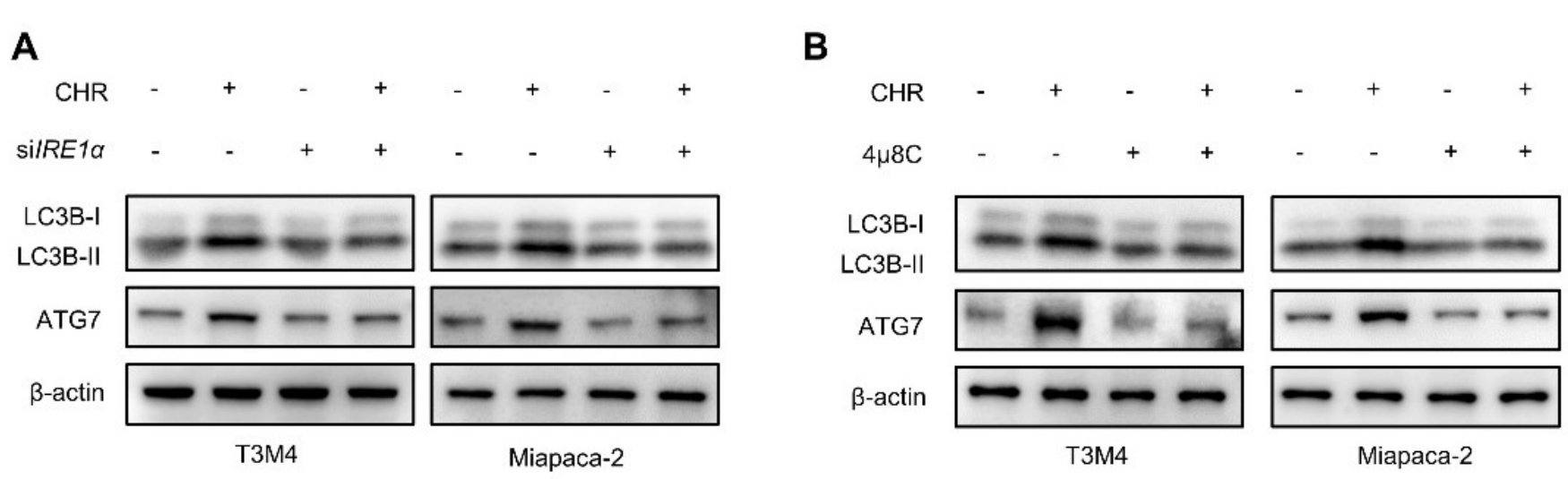

Fig[PbP-R2]. 7 (A) Immunoblotting analysis of autophagy markers in T3M4 and Miapaca-2 cells treated with CHR for 48 h after transfected with si*IRE1α* or si*Scramble*. **(B)** Immunoblotting analysis of autophagy markers in T3M4 and Miapaca-2 cells treated with CHR (10 μM) in the presence or absence of 4μ8C (5 μM) for 48 h.

5. Another important point concerns the reported synergy between CHR and gemcitabine. It is not clear whether the observed effect represents true synergy or a merely additive effect. The criteria for defining synergy are not clearly stated, nor is the type of synergy discussed. Without rigorous quantification, the claim of synergy remains uncertain.

RE: We thank the reviewer for this critical comment. We agree that the term "synergy" should be used with rigorous quantitative support. In response, we have refined our analysis and language in the revised manuscript to enhance precision.

Indeed, we perform a formal dose-matrix analysis using the Zero Interaction Potency (ZIP) model, a reference model that accounts for both potency and efficacy shifts. The analysis confirms that specific concentration pairs, notably 2.5 μM CHR + 50 nM gemcitabine, yield a ZIP synergy score > 10, indicating a significant synergistic interaction. This concentration combination is used in subsequent *in vitro* clonogenic assays, which confirmed the enhanced anti-proliferative effect **(Fig. S10 in the original version)**. However, we do not perform similar analyses in our *in vivo* model. Therefore, we have updated the text accordingly, specifying the *in vitro* synergistic interaction by ZIP scoring and describing the *in vivo* outcome as a "therapeutically enhanced combination effect." We believe this revision provides appropriate rigor and clarity.

6. Finally, the manuscript does not clearly indicate whether appropriate ethical approval was obtained for the collection and use of human tumor samples. This requires explicit clarification to ensure the integrity of the work.

RE: Thank you for your reminder. The collection and use of all patient-derived tumor samples in this study are conducted in strict accordance with ethical standards and are approved by the appropriate institutional review board. The study protocol involving human samples is reviewed and approved by the Clinical Research Ethics Committee of the First Affiliated Hospital, Zhejiang University School of Medicine (Approval Number: [2025B] IIT Ethics Approval No. 1019). We have added this ethical statement in the materials and methods for clarity.

Minor concerns:

7. Beyond these scientific issues, there are additional weaknesses in the manuscript's structure and compliance with basic standards. The introduction contains material that belongs in the results section, resulting in a lack of clarity and coherence. A stricter separation of introduction, results, and discussion is necessary.

RE: Thank you very much. In the revised manuscript, we have thoroughly restructured the text to ensure a clear and logical separation of sections.

8. Essential prior work on IRE1 and UPR signaling in cancer is not cited, weakening the contextualization of the study.

RE: Thank you again. We have incorporated the relevant content into the revised manuscript.

Referee #3 (Remarks for Author):

The authors screened a natural product library and identified chrysosplenetin (CHR) as a cytotoxic compound inducing cell death, proliferation arrest, and apoptosis in pancreatic cancer cells in vitro and in vivo. They show that CHR triggers endoplasmic reticulum (ER) stress and autophagy, primarily through specific interaction with the TMED3 protein, an ER/golgi shuttling transmembrane protein, as demonstrated by various molecular and cellular techniques (proteomic analysis, in silico structural modelling, thermal stability shift assay and Biolayer Interferometry). TMED3 silencing reduces CHR impact on ER stress, autophagy and cell death. Finally, authors describe the potential synergistic effect of CHR with chemotherapy or autophagy inhibition for the induction of cancer cell death. This study's principal novelty lies in characterizing the mechanism of CHR as a TMED3 binder and elucidating its role in ER stress-induced apoptosis in pancreatic cancer, a mode of action so far not described. However, CHR's anti-cancer properties were previously reported for other targets and cancers, which slightly lowers the overall novelty.

Major limitations:

1. While the manuscript presents an hypothesis regarding the role of CHR, the overall robust-ness of these conclusions remain inadequately supported by the data. The identification of TMED3 as a bona fide target of CHR is quite weak. The rationale behind the Venn diagram used for target exclusion (especially BAP18) is not sufficiently explained, and in silico model-ing of the CHR-TMED3 interaction is not accompanied by any experimental validation (site-directed mutagenesis or biochemical mapping of the binding interface). The use of a so-called "purified TMED3 protein" for affinity measurements is problematic: no source or characterization is presented, making the fundamental evidence for TMED3 targeting ambiguous.

RE: We thank the reviewer for the detailed critique regarding the robustness of TMED3 as a direct target. We have addressed each concern with substantial new experimental data to solidify our conclusions.

The Venn diagram identifies two candidate proteins, TMED3 and BAP18. We perform functional validation for both. As shown in **Fig[PbP-R3]. 1**, siRNA-mediated knockdown of *BAP18* does not rescue CHR-induced cytotoxicity, effectively excluding it as a primary functional target. In contrast, as now demonstrated using more rigorous genetic models, TMED3 disruption consistently rescues CHR's effects. This functional prioritization is now clearly stated.

Furthermore, we agree that *in silico* modeling requires experimental support. We have now performed site-directed mutagenesis of key TMED3 residues (Arg20, Glu123, Pro128) predicted to interact with CHR. These TMED3 mutants show significantly attenuated binding to CHR and are markedly resistant to CHR-induced and cell death **(Fig[PbP-R3]. 2-3)**. This provides direct experimental validation of the predicted binding interface and its functional necessity.

Recombinant human wild-type and mutant TMED3 proteins (R20A, E123A, P128A, His-tagged, >80% purity) used fo BLI are purchased from a commercial vendor (OriGene China, TP760290, TP810497, TP810498 and TP810499).We have added this source and characterization information to the materials and methods section.

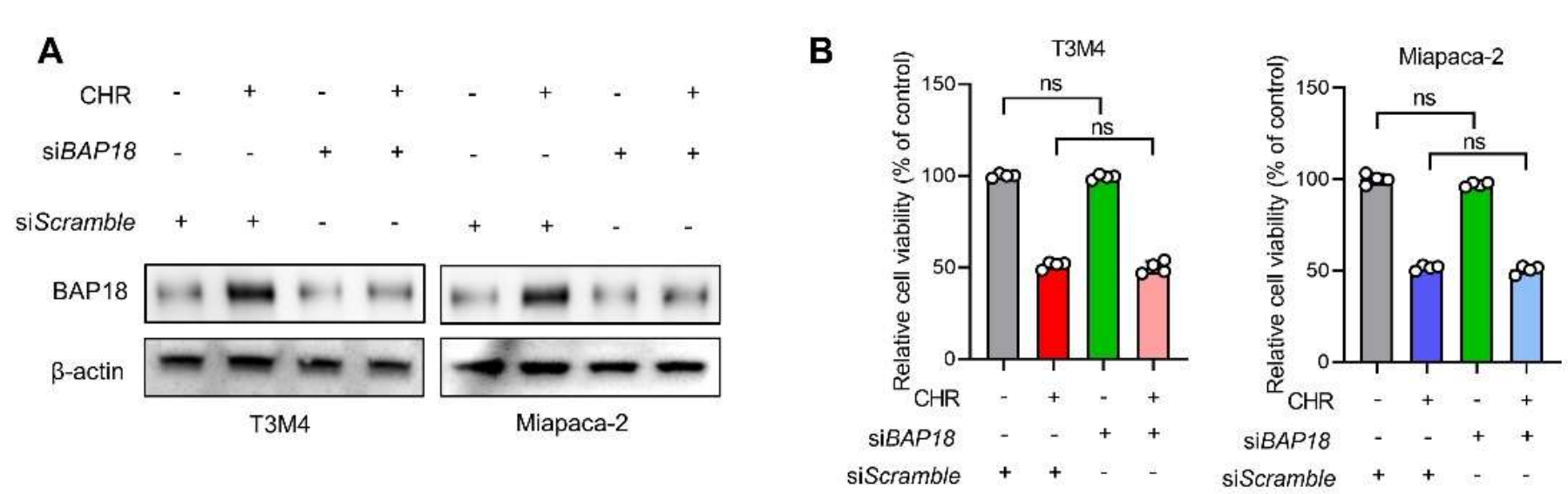

Fig[PbP-R3]. 1 (A) Immunoblotting analysis of BAP18 in T3M4 and Miapaca-2 cells treated with CHR (10 μM) for 48 h after transfected with si*BAP18* or si*Scramble*. **(B)** Cell viability of T3M4 and

Miapaca-2 cells treated with CHR for 48 h after transfected with si*BAP18* or si*Scramble*.

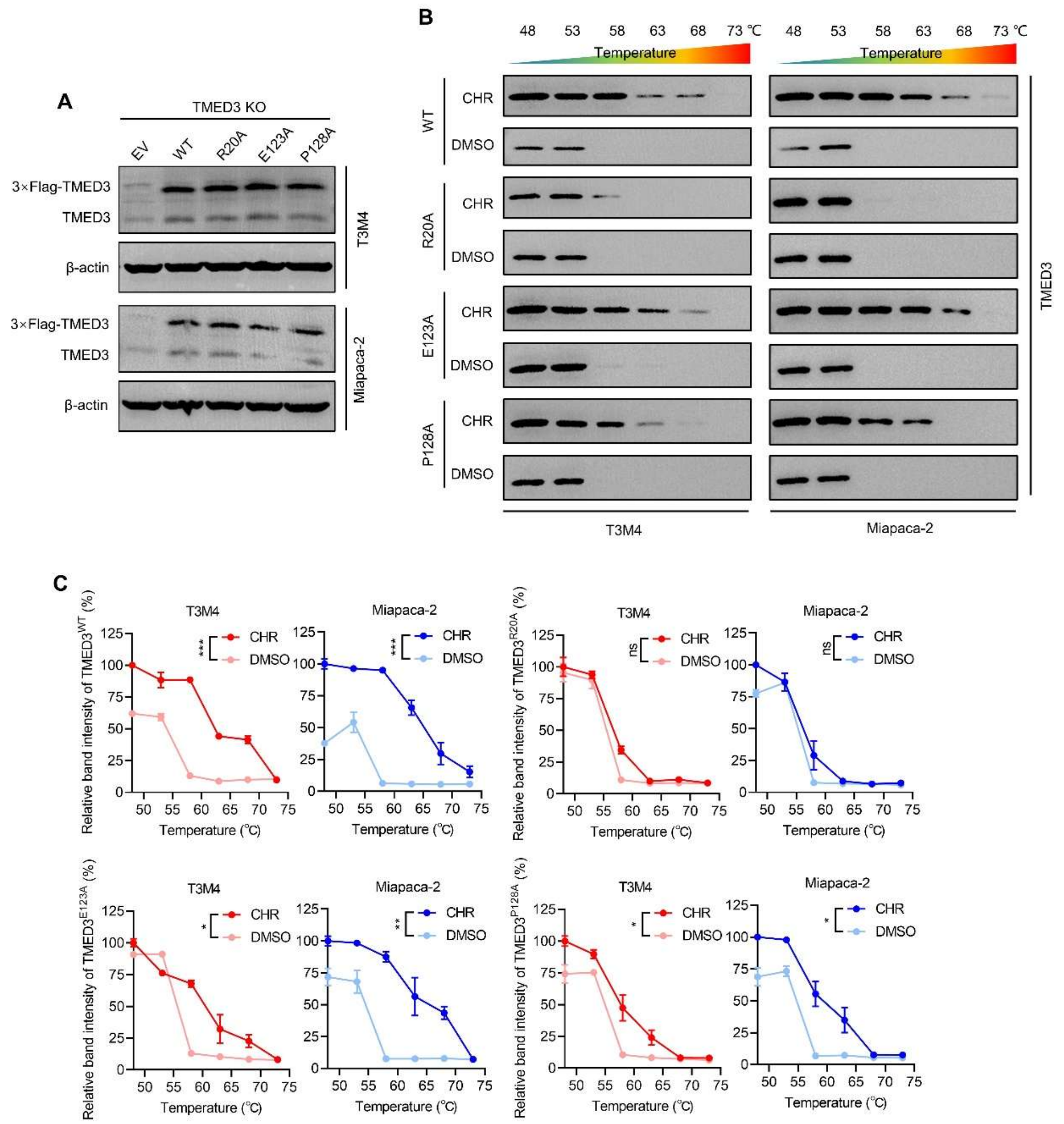

Fig[PbP-R3]. 2 (A) Immunoblotting analysis verifying TMED3 expression in TMED3-KO T3M4 and Miapaca-2 cells (generated with sgRNA#3) upon re-expression of wild-type TMED3 or TMED3 point mutants (R20A, E123A, P128A). **(B-C)** Immunoblotting analysis (B) and quantification of relative band intensity (C) of CETSA for CHR binding to wild-type TMED3, TMED3 R20A, E123A and P128A mutant proteins in TMED3-KO cells. Data are presented as mean ± SD, n=3; $^{*}P < 0.05$, $^{**}P < 0.01$, $^{***}P < 0.001$, ns, no significant.

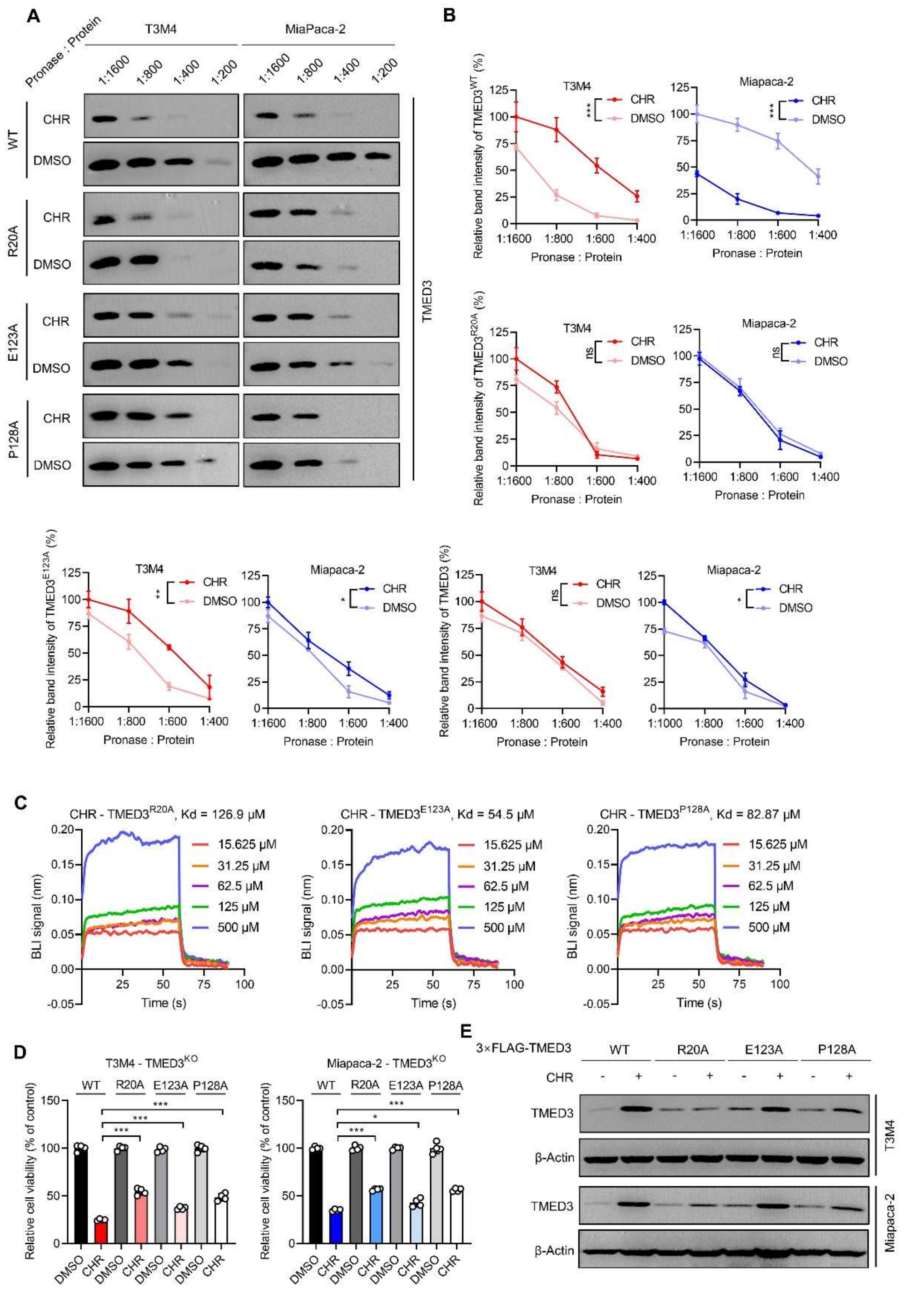

Fig[PbP-R3]. 3 (A-B) Immunoblotting analysis (A) and quantification of relative band intensity (B) of DARTS for CHR binding to wild-type TMED3, TMED3 R20A, E123A and P128A mutant proteins in TMED3-KO cells. **(C)** Bio-layer interferometry sensorgrams indicating direct binding of CHR to TMED3 R20A, E123A and P128A mutant proteins with corresponding Kd values. **(D)** Cell viability of wild-type, R20A, E123A, and P128A mutant-reconstituted TMED3-KO T3M4 and Miapaca-2 cells after 48 h of CHR (20 μM) or DMSO treatment. **(E)** Immunoblotting for TMED3 in TMED3-

KO T3M4 and Miapaca-2 cells re-expressing wild-type TMED3 or its mutants (R20A, E123A, P128A) after 48 h of CHR or DMSO treatment. Data are presented as mean ± SD, n=3/4; $^{*}P < 0.05$, $^{**}P < 0.01$, $^{***}P < 0.001$, ns, no significant.

2. Moreover, siRNAs targeting TMED3 or ATG7 result in only marginal reduction in protein levels and fail to robustly modulate the core readouts (cell viability, autophagy, ER stress marker expression). These limited knockdown efficiency weakens all downstream interpreta-tions. The use of non-specific pharmacological modulators such as wortmannin (a PI3K in-hibitor) and PBA (a chemical chaperone with broad cellular effects) instead of highly selec-tive pathway inhibitors (such as 4uC8, Perki for ER stress; bafilomycin for autophagy) further undermines the specificity of mechanistic claims made for CHR action. Taken together, while CHR demonstrably induces ER stress, autophagy, and cancer cell death, the data suggest these effects may involve broadly cytotoxic mechanisms similar to those observed with established chemotherapeutics such as gemcitabine or paclitaxel, both of which are known to trigger ER stress responses and apoptosis without direct engagement of ER-specific proteins [including Palam CDD 2015, Xu Cancer Res Treat 2014 among others]

RE: Thank you very much for your suggestions. We have systematically addressed these concerns in the revised manuscript with new and improved experimental data.

Firstly, we agree that partial protein knockdown can limit phenotypic interpretation. To address this, we have generated and characterized CRISPR/Cas9-mediated TMED3 KO cell lines. As shown in **Fig^{PbP-R3}. 4**, these KO cells exhibit near-complete loss of TMED3 protein and display a significant attenuation of CHR-induced phenotypes, including UPR marker expression, cytotoxicity, and apoptosis.

For ATG7, we have optimized siRNA conditions to achieve more effective knockdown. The improved knockdown efficiency, confirmed by western blot, now

leads to a more pronounced enhancement of CHR-induced cell death **(Fig[PbP-R3]. 5)**.

To increase the specificity of our pathway interrogation, we have performed new experiments with highly selective inhibitors. For the IRE1α/XBP1 branch of UPR, we use the specific RNase inhibitor 4μ8C. Treatment with 4μ8C effectively inhibits CHR-induced XBP1 splicing and attenuates downstream autophagy markers **(Fig[PbP-R3]. 6)**.

For the PERK branch, we use the selective inhibitor PERKi (GSK2606414). PERKi treatment significantly reduces CHR-induced expression of CHOP **(Fig[PbP-R3]. 7)**.

For autophagy flux, we complement CQ data with Bafilomycin A1, a specific V-ATPase inhibitor that blocks autolysosome acidification. Bafilomycin A1 treatment similarly enhances CHR-induced LC3B-II accumulation and cell death, confirming the role of functional autophagy **(Fig[PbP-R3]. 8)**.

While we agree that some chemotherapies can secondarily induce ER stress, our data establish a primary, target-driven mechanism for CHR. Unlike the indirect stress caused by gemcitabine (DNA damage) or paclitaxel (microtubule disruption), CHR directly binds to and aggregates TMED3, an ER-resident protein, thereby initiating ER stress. The genetic evidence (TMED3 KO and point mutants) shows that TMED3 is required for CHR's effects, which is a criterion not met for a non-specific cytotoxic agent **(Fig[PbP-R3]. 3-4)**. This target-specific initiation differentiates CHR's action from the broad downstream consequences of conventional chemotherapy.

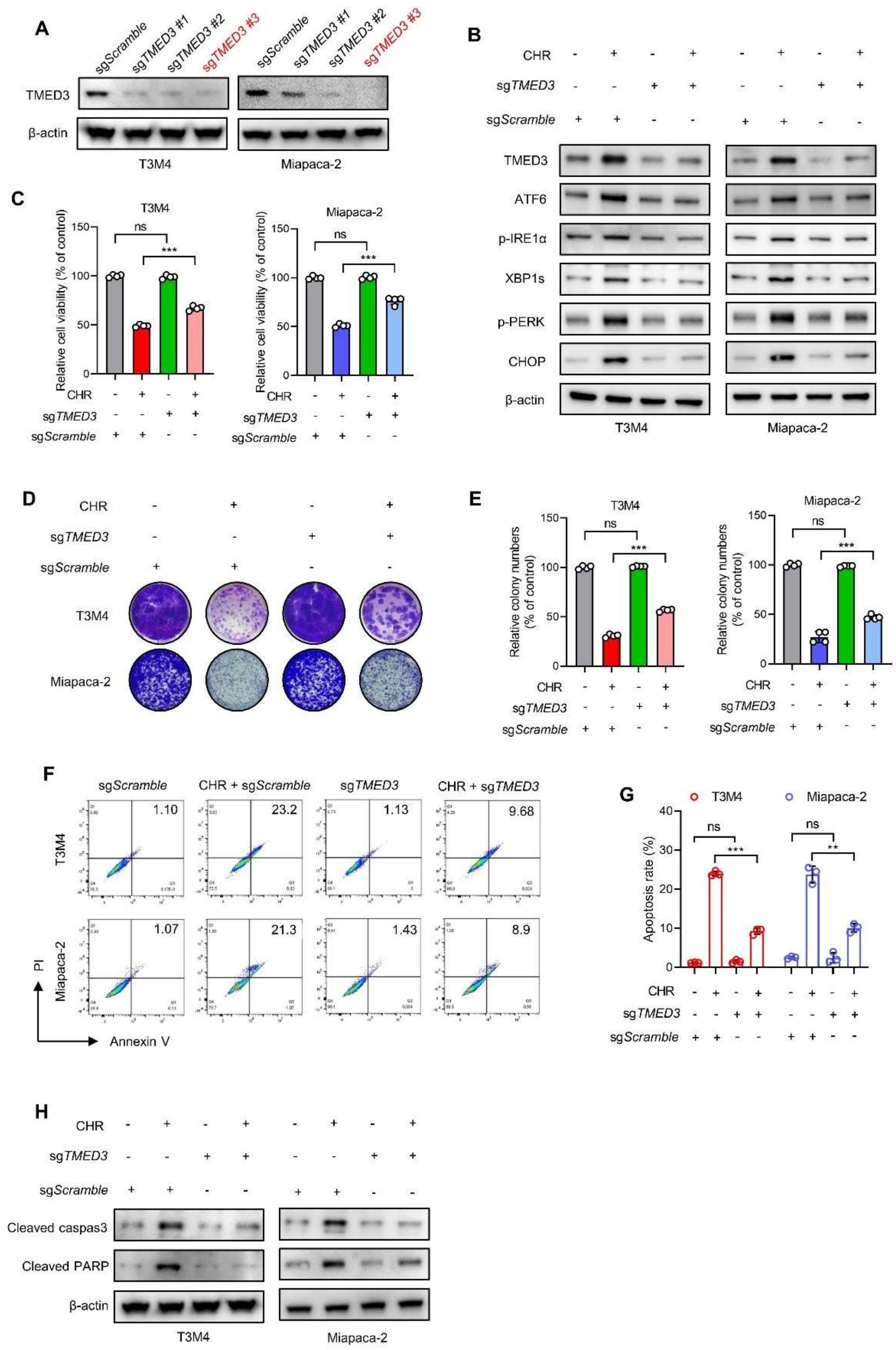

Fig[PbP-R3]**. 4 (A)** Immunoblotting analysis verifying TMED3 knockout efficiency in T3M4 and Miapaca-2 cells generated using three independent sgRNAs targeting *TMED3*. **(B)** Immunoblotting analysis of ER stress markers in wild-type and TMED3-KO T3M4 and Miapaca-2 cells treated with CHR (10 μM) for 48 h. **(C)** Cell viability of wild-type and TMED3-KO T3M4 and Miapaca-2 cells treated with CHR for 48 h. **(D-E)** Representative images (D) and quantification of colony numbers (E) of wild-type and TMED3-KO T3M4 and Miapaca-2 cells treated with CHR. **(F-G)** Flow

cytometric analysis (F) and quantification (G) of apoptosis rate in wild-type and TMED3-KO T3M4 and Miapaca-2 cells treated with CHR for 48 h. **(H)** Immunoblotting analysis of CC3 and cleaved PARP in wild-type and TMED3-KO T3M4 and Miapaca-2 cells treated with CHR for 48 h. Data are presented as mean ± SD, n=3/4; $^{**}P < 0.01$, $^{***}P < 0.001$, ns, no significant.

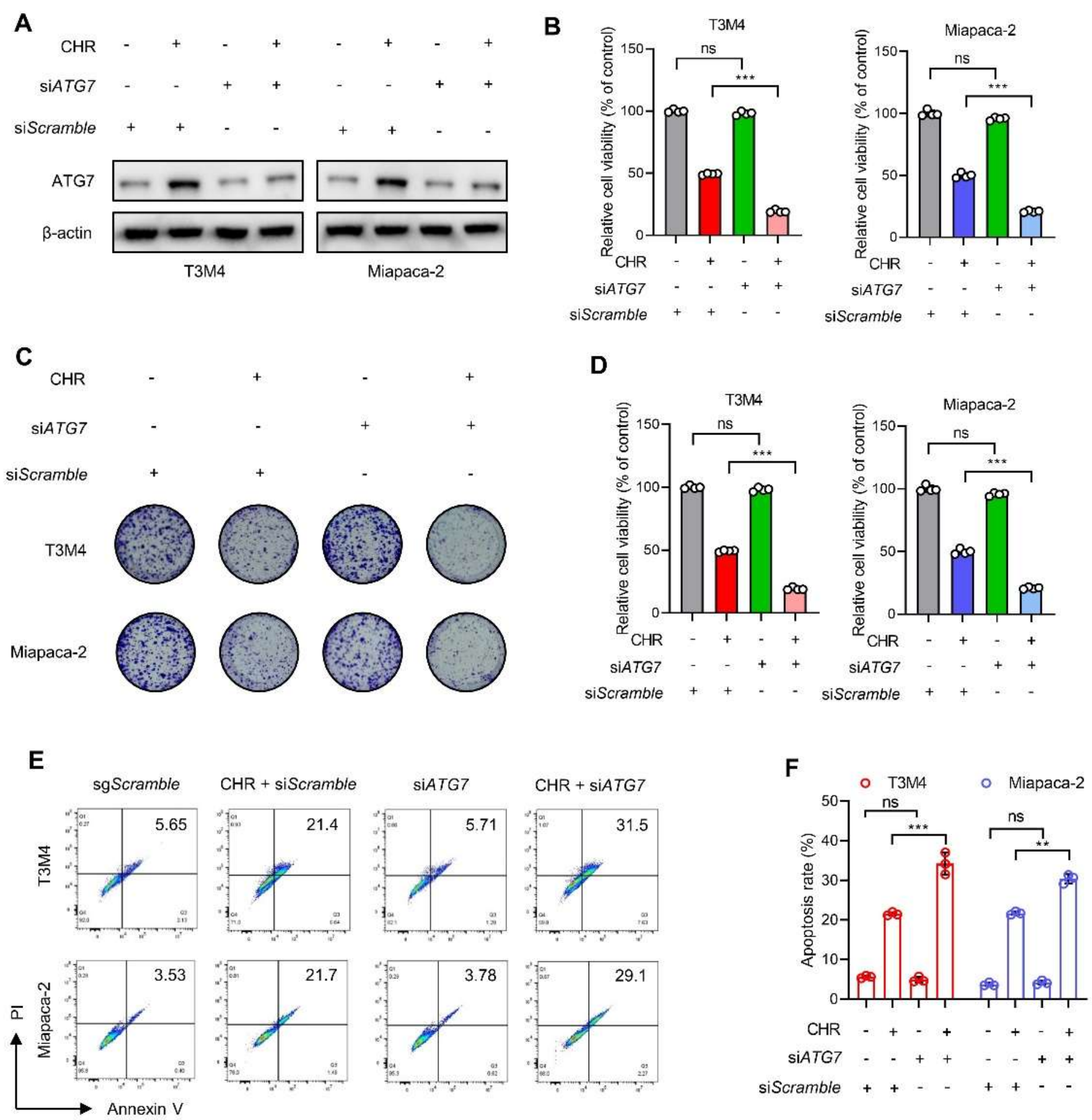

Fig[PbP-R3]. 5 (A) Immunoblotting analysis of ATG7 in T3M4 and Miapaca-2 cells treated with CHR (10 μM) for 48 h after transfected with si*ATG7* or si*Scramble*. **(B)** Cell viability of T3M4 and Miapaca-2 cells treated with CHR for 48 h after transfected with si*ATG7* or si*Scramble*. **(C-D)** Representative images (C) and quantification of colony numbers (D) of T3M4 and Miapaca-2 cells treated with CHR after transfected with si*ATG7* or si*Scramble*. **(E-F)** Flow cytometric analysis (E) and quantification (F) of apoptosis rate in T3M4 and Miapaca-2 cells treated with CHR for 48 h after transfected with si*ATG7* or si*Scramble*. Data are presented as mean ± SD, n=3/4; $^{*}P < 0.05$, $^{**}P < 0.01$, $^{***}P < 0.001$, ns, no significant.

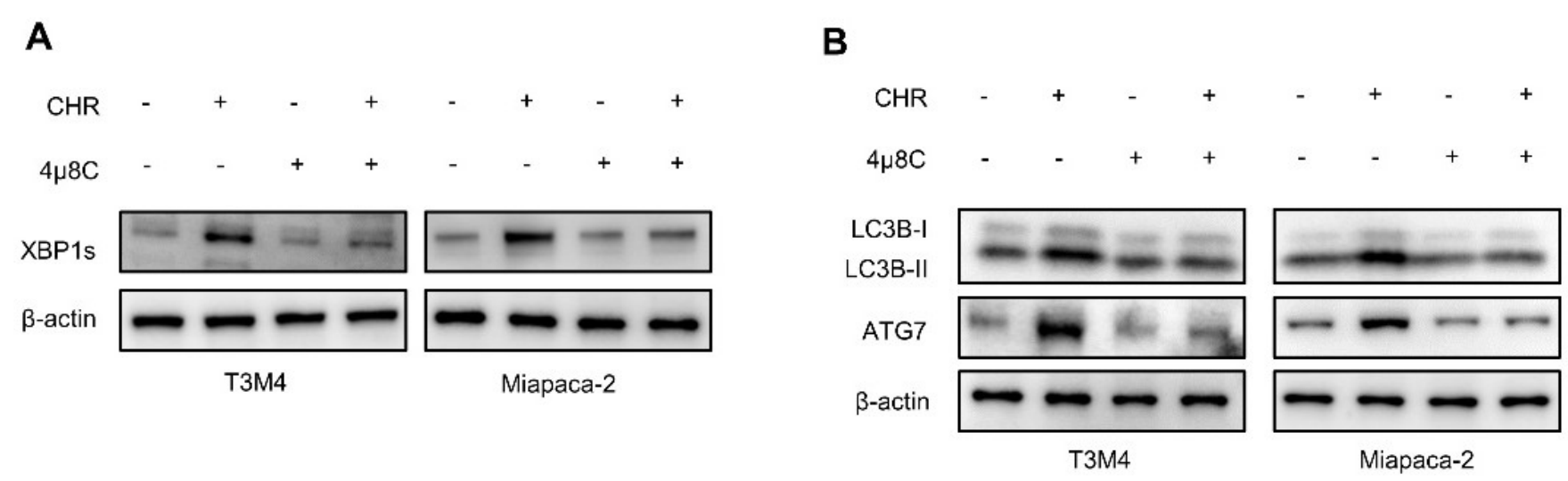

Fig[PbP-R3]. 6 (A) Immunoblotting analysis of XBP1s in T3M4 and Miapaca-2 cells treated with CHR (10 μM) in the presence or absence of 4μ8C (5 μM) for 48 h. **(B)** Immunoblotting analysis of autophagy markers in T3M4 and Miapaca-2 cells treated with CHR in the presence or absence of 4μ8C for 48 h.

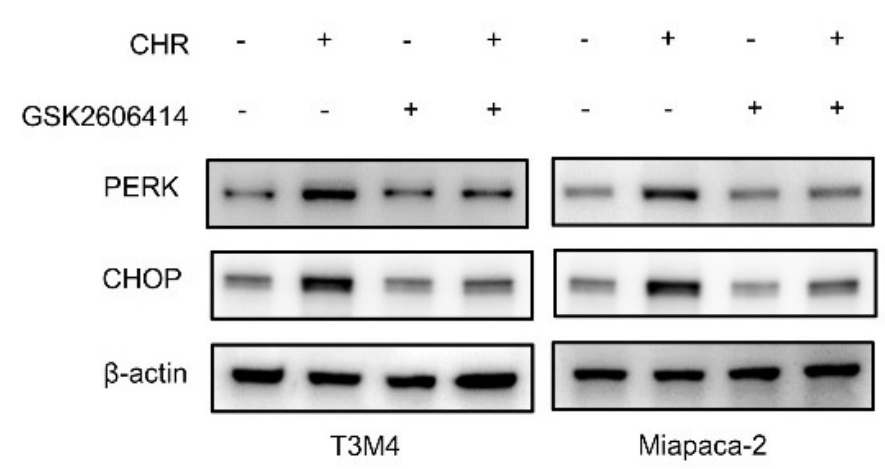

Fig[PbP-R3]. 7 Immunoblotting analysis of PERK and CHOP in T3M4 and Miapaca-2 cells treated with CHR in the presence or absence of GSK2606414 (5 μM) for 48 h.

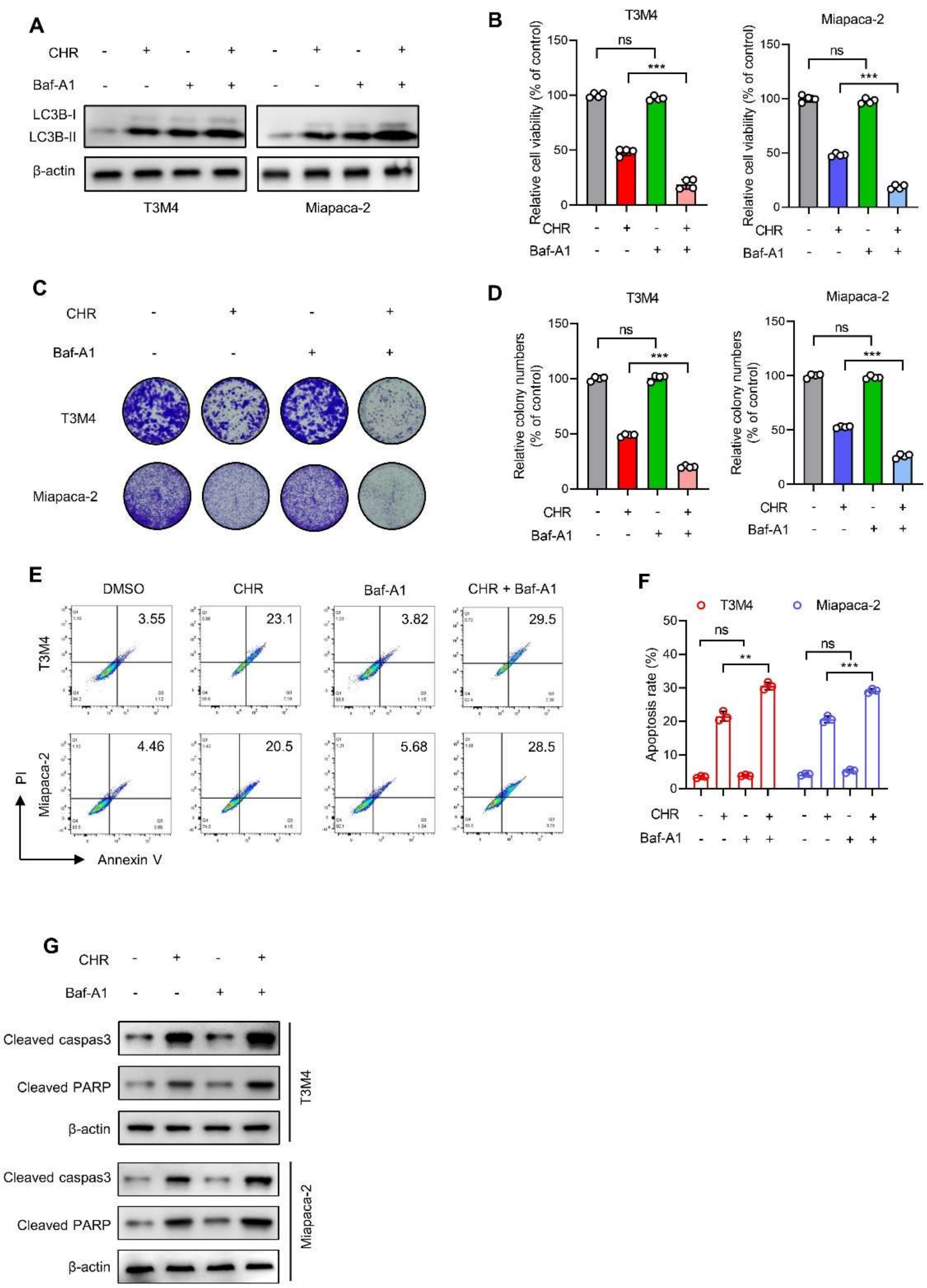

Fig[PbP-R3]. 8 (A) immunoblotting analysis of LC3B in T3M4 and Miapaca-2 cells pretreated with Baf-A1 (100 nM) for 6 h, followed by CHR treatment for 48 h. **(B)** Cell viability of T3M4 and Miapaca-2 cells pretreated with Baf-A1 (100 nM) for 6 h, followed by CHR treatment for 48 h. **(C-D)** Representative images (C) and quantification of colony numbers (D) of T3M4 and Miapaca-2 cells treated with CHR in the presence or absence of Baf-A1. **(E-F)** Flow cytometric analysis (E) and quantification (F) of apoptosis rate in T3M4 and Miapaca-2 cells pretreated with Baf-A1 (100 nM) for 6 h, followed by CHR treatment for 48 h. **(G)** Immunoblotting analysis of CC3 and cleaved PARP in T3M4 and Miapaca-2 cells pretreated with Baf-A1 (100 nM) for 6 h, followed by CHR treatment for 48 h. Data are presented as mean ± SD, n=3/4; $^{*}P < 0.05$, $^{**}P < 0.01$, $^{***}P < 0.001$, ns,

no significant.

3. Notably, the manuscript's central claim that CHR induces TMED3 aggregation leading to UPR activation, remains without any direct experimental evidence.

RE: We respectfully but firmly disagree with the reviewer’s statement that our central claim lacks direct experimental evidence. On the contrary, we have provided a multi-layered set of data that directly and mechanistically connects CHR binding to TMED3 aggregation and subsequent UPR activation.

Firstly, BLI assay demonstrates a concentration-dependent, physical interaction between purified recombinant TMED3 and CHR with a quantifiable affinity. CETSA shows that CHR treatment significantly increases the thermal stability of endogenous TMED3 in living cells, confirming target engagement in a physiologically relevant context. Furthermore, DARTS indicates that CHR binding protects endogenous TMED3 from pronase digestion, providing further evidence of direct interaction **(Fig. 5 in the original version)**. Notably, our new data show that point mutations in TMED3 predicted by docking to interact with CHR, abolish CHR-induced protein stabilization and attenuate the cell death, providing structure-function validation of the specific binding site **(Fig^{PbP-R3}. 2-3)**.

Importantly, we also performed biochemical assays under non-denaturing conditions. Under basal conditions, TMED3 migrated as a distinct band at approximately 100 kDa, consistent with its stable oligomeric form (PMID: 41364076). CHR treatment induced a marked intensification of the 100 kDa band along with the appearance of higher-molecular-weight species, indicating progressive oligomerization or aggregation **(Fig^{PbP-R3}. 9)**. Then, CRISPR/Cas9 knockout of TMED3 significantly blunted CHR-induced upregulation of UPR markers and rescued cells from CHR-mediated cytotoxicity and apoptosis **(Fig^{PbP-R3}. 4)**.

Taken together, the claim that CHR induces TMED3 aggregation leading to UPR activation is supported by direct evidence of (1) binding, (2) consequent protein stabilization and subcellular accumulation, and (3) the genetic necessity of TMED3 for initiating the downstream UPR and apoptotic response. We believe this evidence chain is robust and directly substantiates our central mechanistic model.

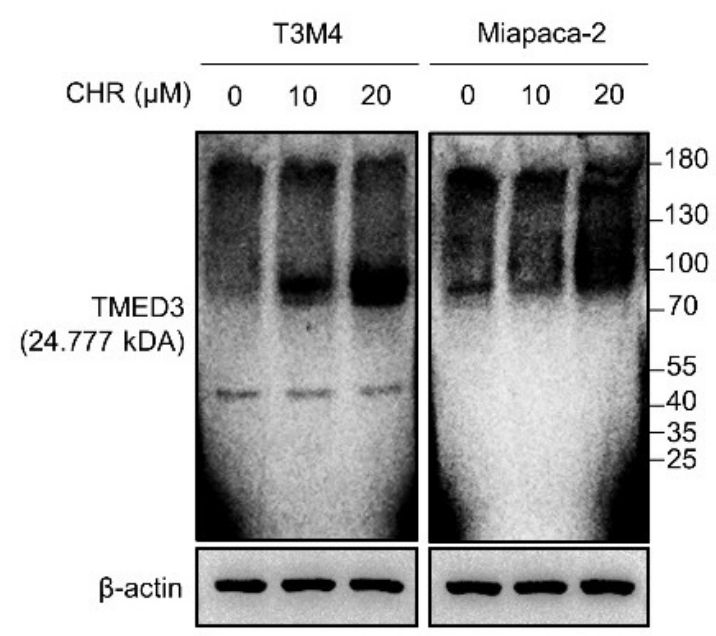

Fig^PbP-R3. 9 Native gel electrophoresis analysis of CHR-induced TMED3 oligomerization in T3M4 and Miapaca-2 cells. β-actin (reducing gel) as the quantitative loading control.

4. Finally, clinical correla-tive analyses (TMED3 expression vs overall survival) are limited to a single, unreferenced cohort with small differences, rendering translational significance highly speculative at this stage.

RE: We thank the reviewer for raising this important point regarding the clinical relevance of TMED3. In the original version, we analyze pancreatic cancer transcriptomic data from 16 datasets (PMID: 37260110). This independent, large-scale public dataset validates that lower TMED3 expression correlates significantly with poorer patient prognosis. To strengthen our findings, we also analyze an independent cohort of pancreatic cancer patient samples via tissue microarray **(Fig^PbP-R3. 10)**. These two independent lines of clinical evidence consistently support the clinical relevance of TMED3 in pancreatic cancer and strengthen the translational rationale for targeting this protein. We have updated the manuscript accordingly.

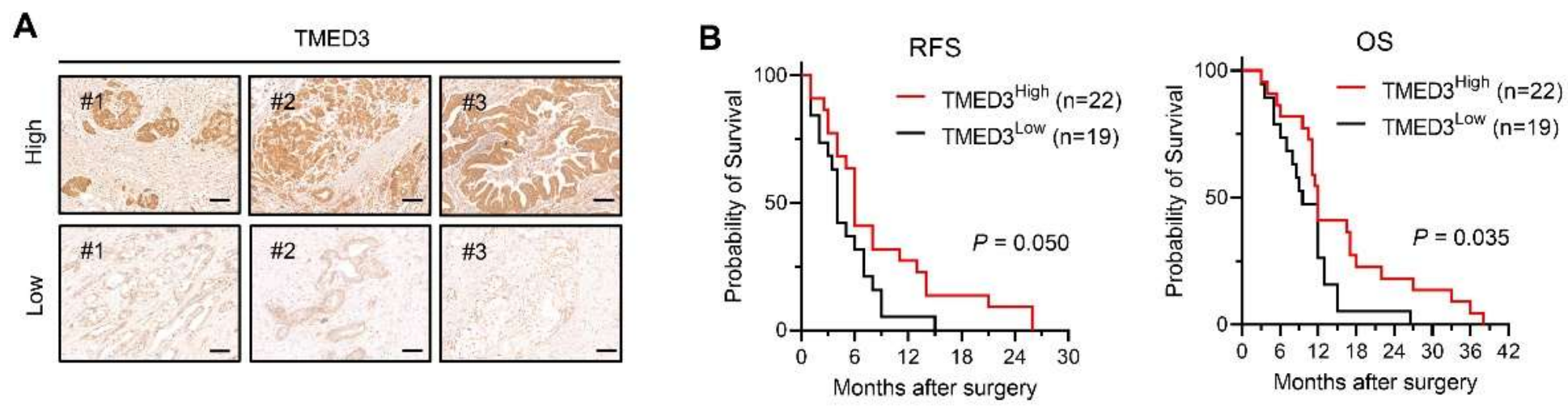

Fig^{PbP-R3}. 10 (A) Representative images of immunohistochemical staining for TMED3 in PDAC patient tumor tissueswith three representative cases each for high- and low-TMED3 expression groups. Scale bar: 100 µm. **(B)** Kaplan-Meier analysis of PDAC patients based on TMED3 expression levels derived from tissue microarray (A).

Major points

Requirement for Robust Genetic Validation of TMED3 Specificity.

1. Demonstrating the specificity of CHR's anticancer effect through TMED3 requires rigorous genetic tools beyond the modest siRNA knockdowns presented. The authors must conduct CRISPR-Cas9 or shRNA-mediated knockdown/knockout of TMED3 with multiple independent constructs, confirming efficient protein depletion at the western blot level.

RE: We agree with the reviewer that rigorous genetic validation is essential. As indicated in our previous responses, we have performed CRISPR-Cas9-mediated knockout of TMED3 using multiple sgRNAs and selected the clone with near-complete protein ablation for all functional studies. These TMED3-KO cells show a robust attenuation of CHR-induced phenotypes, including UPR activation, cytotoxicity, and apoptosis **(Fig^{PbP-R3}. 4)**. These data provide conclusive genetic evidence supporting TMED3 as a critical mediator of CHR’s action and are now included in the revised manuscript.

2. Furthermore, re-expression of TMED3 mutants rendered resistant to CHR binding,

specifically point mutants at Arg20, Glu123, or Pro128, must be performed to show functional rescue. These experiments are essential to prove that TMED3 is the critical binding partner mediating CHR action rather than a secondary or off-target effect. The use of such recombinant TMED3 is mandatory to repeat fig 5J, L and N. This would actually demonstrate the CHR direct binding to TMED3 is sufficient to induce conformation changes, increased thermal stability, resistance to pronase degradation and allow the identification of accurate Kd.

RE: We have fully addressed this essential point. As detailed in our responses and in the revised manuscript, we have generated TMED3 point mutants targeting the CHR-binding interface predicted by docking **(Fig[PbP-R3]. 2-3)**. These experiments directly prove that CHR binding to this specific site on TMED3 is required for its biological effects, eliminating explanations based on off-target or secondary effects. The corresponding data are now included in the revised manuscript.

Critical Evaluation of Target Identification Approaches and Consideration of Other Candidate Targets

1. The identification of TMED3 as the sole target via a Venn diagram approach is inadequately justified. Notably, BAP18 was excluded based on in silico analyses and transcriptomic data affected by a 12-hour cycloheximide (CHX) treatment, that will mask transcriptomic changes and proteins with half-lives longer than 12 hours, are unlikely to show altered levels during such treatment. This warrants a broader exploration of other putative targets, such as BAP18, through functional assays to determine compound specificity and off-target effects.

RE: Thank you. We have performed the functional validation suggested. SiRNA-mediated knockdown of *BAP18* does not rescue CHR-induced cytotoxicity, in contrast

to the robust rescue observed with TMED3 knockdown/knockout **(Fig[PbP-R3]. 1** and **Fig[PbP-R3]. 4)**. This functional evidence, together with the proteomic and transcriptomic data, supports the exclusion of BAP18 as a primary functional target of CHR.

Comprehensive Biophysical Characterization Using Recombinant TMED3 Protein

1. The manuscript's claims regarding direct CHR binding to TMED3 and its consequent effects on protein conformation, thermal stability, and resistance to proteolytic digestion (as presented in figure 5J, L, N) must be substantiated using fully characterized, recombinant TMED3 protein, both wildtype and the aforementioned point mutants. Binding curves allowing accurate determination of dissociation constants (Kd) are required. Parallel testing on other ER-resident transmembrane proteins (PERK, IRE1, SEC61, GM130) is necessary as controls to decisively demonstrate that observed effects are TMED3-specific and not due to generalized ER protein perturbation, as commonly seen with other ER stress inducers such as DTT, thapsigargin, or tunicamycin.

RE: Thank you very much for your suggestion. We have already thoroughly discussed the specificity of the CHR-TMED3 interaction in our previous response. As expected, no interaction is detected between CHR and other key endoplasmic reticulum-resident transmembrane proteins by CETSA, including PERK, IRE1α and SEC61 **(Fig[PbP-R3]. 11)**.

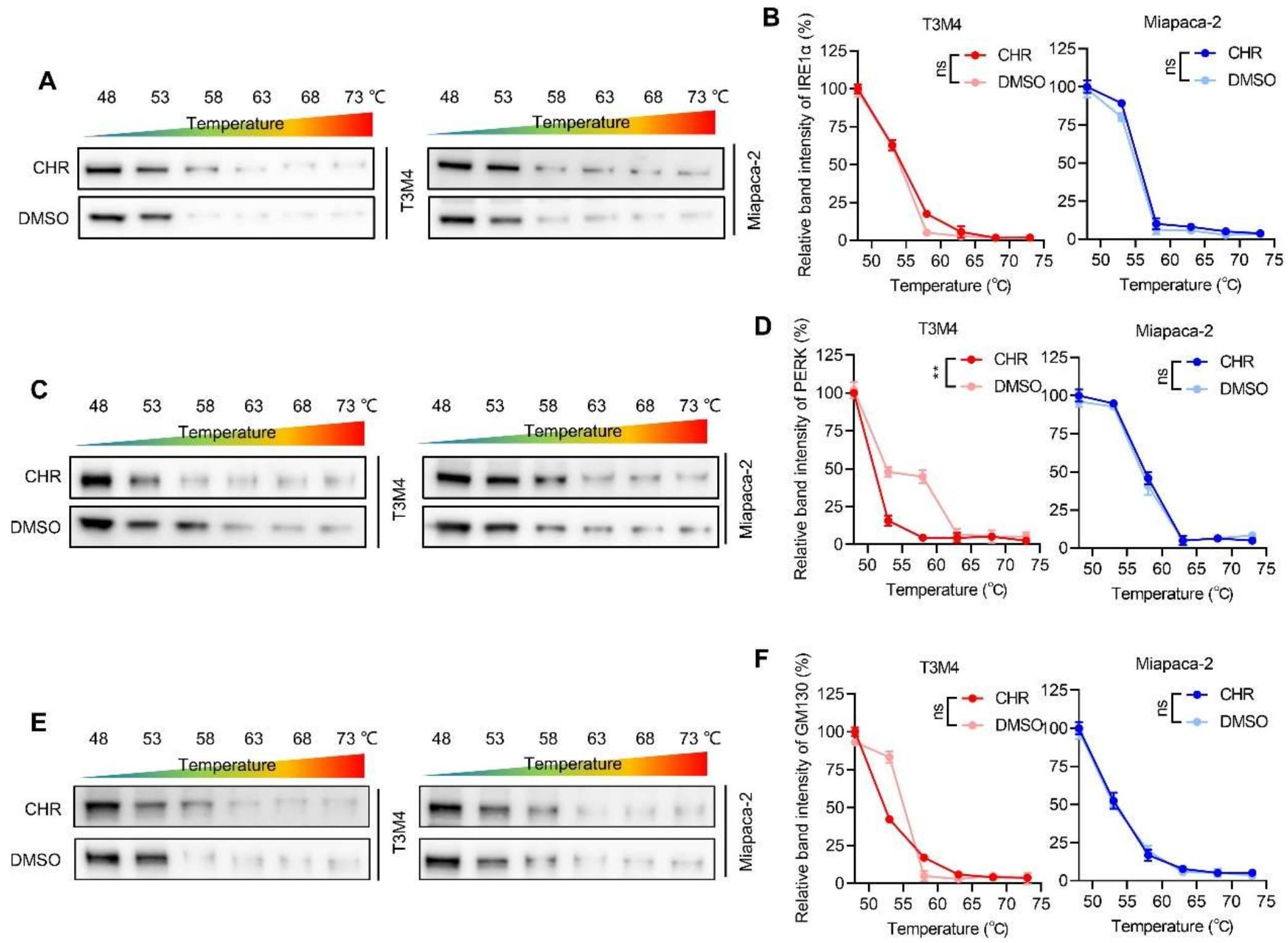

Fig[PbP-R3]. 11 (A-B) Immunoblotting analysis (A) and quantification of relative band intensity (B) of CETSA for CHR binding to IRE1α in T3M4 and Miapaca-2 cells. **(C-D)** Immunoblotting analysis (C) and quantification of relative band intensity (D) of CETSA for CHR binding to PERK in T3M4 and Miapaca-2 cells. **(E-F)** Immunoblotting analysis (E) and quantification of relative band intensity (F) of CETSA for CHR binding to GM130 in T3M4 and Miapaca-2 cells.

Temporal Resolution of ER Stress and Autophagy Response

1. Current data are limited to dose-responses without adequate kinetic analysis. A detailed time-course dissection of CHR-mediated activation of key ER stress sensors (IRE1, PERK) and autophagy markers (e.g., LC3 conversion) must be conducted, including assays using selective pharmacological inhibitors such as 4uC8 (IRE1 inhibitor), Perk inhibitor, and bafilomycin (autophagy inhibitor). This would clearly define the sequence and interdependencies of cellular pathways activated by CHR and distinguish primary mechanism from downstream effects.

RE: Thank you for this constructive suggestion. As requested, we have performed a

detailed time-course analysis using a effective concentration of CHR (20 μM). The new data clearly show the activation kinetics of key pathways over 48 hours. Firstly, ATF6 and phosphorylation of PERK and IRE1α are detectable as early as 2-4 hours post-CHR treatment, preceding the upregulation of downstream effectors like CHOP and XBP1s (evident by 8-12 hours). For autophagy induction, the conversion of LC3B-I to LC3B-II and upregulation of ATG7 begin by 8-12 hours, following the initial phosphorylation of IRE1α. This temporal sequence supports our model that autophagy is a secondary, adaptive response to CHR-induced ER stress. In addition, cleavage of caspase-3 and PARP become prominent at 24-48 hours, consistent with apoptosis being a later-stage outcome of sustained, unresolved ER stress **(Fig^{PbP-R3}. 12)**. These data, combined with our previous experiments using selective inhibitors (4μ8C, PERKi, Bafilomycin A1), provide a comprehensive mechanism.

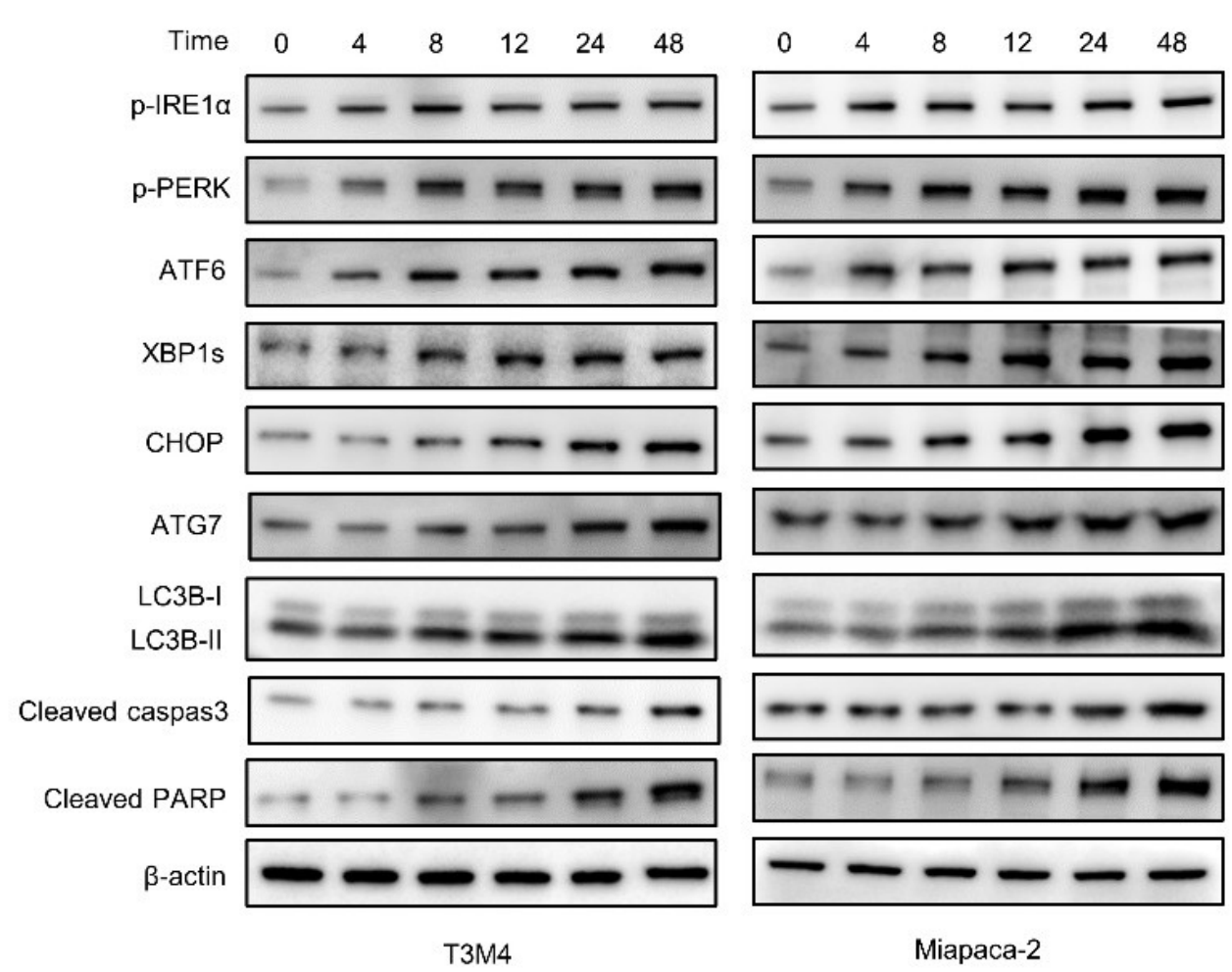

Fig^{PbP-R3}. 12 Immunoblotting analysis of ER stress, autophagy, and apoptosis markers in T3M4 and Miapaca-2 cells treated with CHR (20 μM) for indicated time durations.

Direct Evidence of TMED3 Aggregation

The hypothesis that CHR induces TMED3 aggregation, which triggers UPR and subsequent apoptosis, is a central mechanistic claim that lacks direct experimental

support. Rigorous validation of TMED3 aggregation, via biochemical assays (native gels), advanced microscopy, or biophysical techniques, is imperative to substantiate this claim. Moderate the conclusions on TMED3 aggregate if these experiments fails to demonstrate this event.

RE: Thank you again. We have provided direct biochemical evidence for CHR-induced TMED3 aggregation using non-denaturing (native) gel electrophoresis. As shown in **Fig$^{PbP\text{-}R3}$. 9**, TMED3 predominantly exists as an ~100 kDa heterotetramer under basal conditions, with faint high-molecular-weight smears also detectable, reflecting low levels of physiological higher-order oligomerization or heterogeneous complexes under normal conditions (PMID: 41364076). Upon CHR treatment, the intensity of the ~100 kDa band increased dose-dependently, while 20 μM CHR further promoted the formation of soluble high-molecular-weight oligomers, as indicated by the enhanced upward smear.

Minor Points

Experimental Validation and Genetic Tools

1. Current siRNA-mediated silencing of TMED3 and ATG7 is weak and insufficient to support mechanistic conclusions. Use several independent siRNA or shRNA sequences with confirmed knockdown efficiency, validated at the protein level. Provide thorough knockdown validation for reliability. Colony formation and autophagy experiments require repetition with the above-mentioned silencing tools to confirm robustness and reproducibility.

RE: Thank you for your comment. We have already addressed these issues previously. The relevant results have been incorporated into the revised manuscript.

2. Effects of siATG7 on apoptosis are weak and inconsistent with pharmacologic inhibitors like chloroquine or wortmannin; conclusions about autophagy's role should be moderated or further supported.

RE: Thank you again. Please refer to the previous response.

Biochemical and Molecular Methods

1. The nature, origin, and purification details of TMED3 protein used in binding and stability (Figures 5M-N) are not provided; this should be clearly detailed in methods.

RE: We have supplemented the relevant information in the revised manuscript.

2. The use of wortmannin as an autophagy inhibitor is misleading since it targets PI3K; authors should justify its use or substitute with more specific autophagy modulators like bafilomycin.

RE: We have supplemented the relevant results in the revised manuscript.

3. Subcellular localization of TMED3 (Figure 5E) should be validated by additional markers for ER (PERK, IRE1, Sec61) and Golgi (GM130) to ensure precise compartment assignment.

RE: We thank the reviewer for this suggestion to further validate the subcellular localization of TMED3. In the revised manuscript, we have extended our immunofluorescence analysis. As shown in **Fig[PbP-R3]. 13**, we perform co-staining of TMED3 with additional specific organelle markers. The results confirm that CHR-induced TMED3 accumulation strongly co-localizes with the ER marker SEC61β, and partially overlaps with the Golgi marker GM130. This refined analysis provides more comprehensive evidence that CHR induces TMED3 accumulation mainly within the

endoplasmic reticulum compartment, consistent with its role in initiating ER stress.

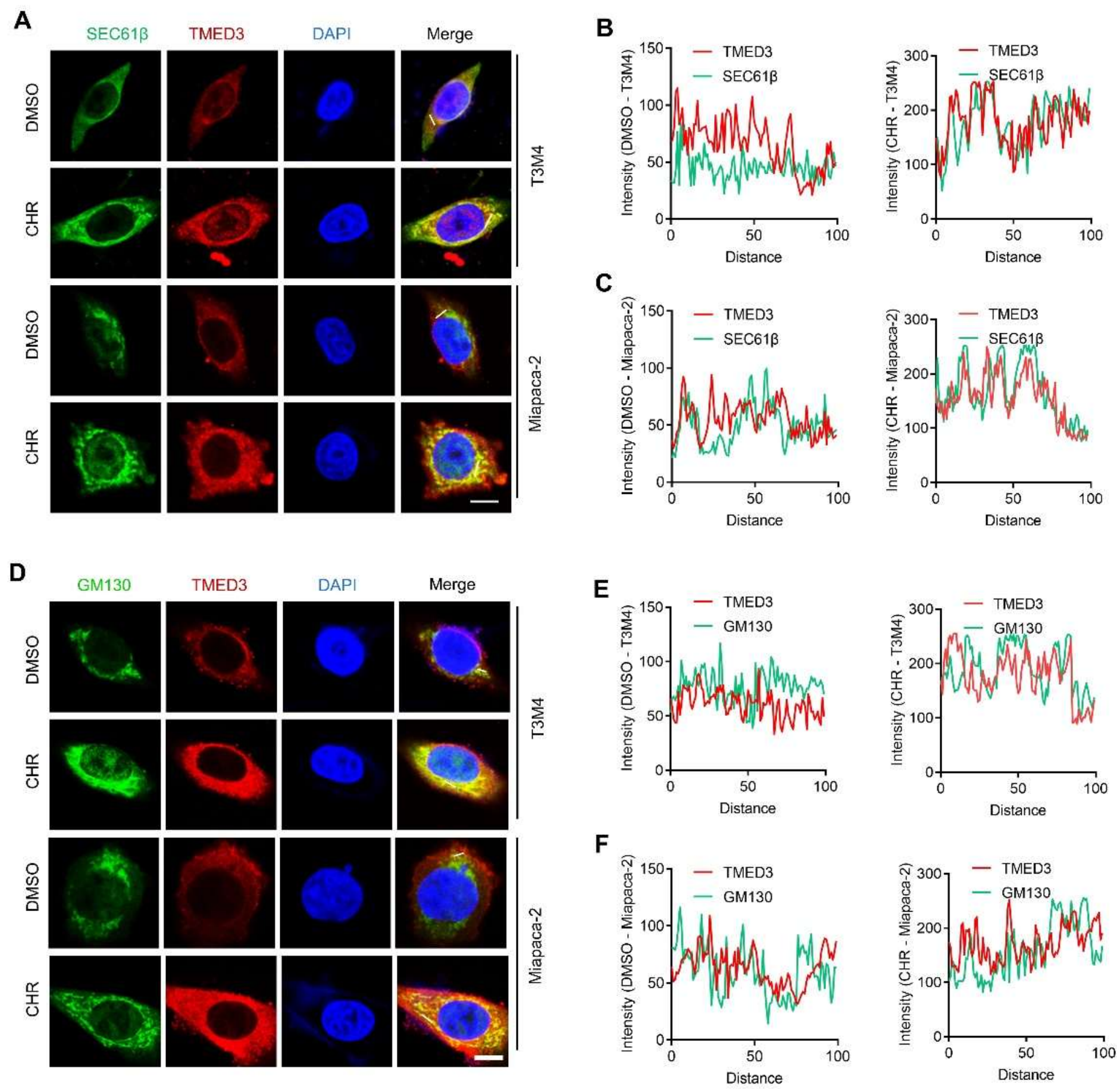

Fig[PbP-R3]. 13 (A-C) Representative images (A) and colocalization analysis of SEC61β and TMED3 (B-C) of immunofluorescent staining for SEC61β (green), TMED3 (red) and DAPI (blue) in T3M4 and Miapaca-2 cells treated with DMSO or CHR, scale bar: 10 μm. **(D-F)** Representative images (D) and colocalization analysis of GM130 and TMED3 (E-F) of immunofluorescent staining for GM130 (green), TMED3 (red) and DAPI (blue) in T3M4 and Miapaca-2 cells treated with DMSO or CHR, scale bar: 10 μm.

Imaging and Data Presentation

1. Immunohistochemistry images, especially for caspase-3 cleavage, are blurry with unspecific staining; higher quality and better characterized images are needed to substantiate apoptosis claims.

RE: We have repeated the experiments and incorporated the new figures into the revised

manuscript.

2. Co-immunofluorescence figures show labeling inconsistencies; e.g., Figure S9E's text mentions TMED3/ERp72 colocalization but the image depicts LC3/ERp72.

RE: Thank you. The error has been corrected.

3. FACS panels are too small, poorly labeled, and hard to interpret; larger, well-annotated plots are necessary for transparency.

RE: Thank you. The figure has been re-edited.

4. Supplementary table (end of main PDF file) lacks proper formatting, impeding readability; they should be reformatted with clear alignment, headings, and explanations.

RE: The new table has been modified and uploaded.

Data Analysis and Interpretation

1. Differential sensitivity of pancreatic cancer lines to CHR (mild effect in Miapaca vs. T3M4) should be explained to characterize variability in response.

RE: Thank you. Based on the new data, we observed that CHR induced TMED3 protein accumulation in all tested pancreatic cancer cell lines (Miapaca-2, PANC-1, SUIT-2, SW1990, and T3M4) in a dose-dependent manner (0, 10, 20 μM). However, the extent of accumulation varied across lines, with the most pronounced effects consistently observed in Miapaca-2 and T3M4 cells **(Fig$^{PbP\text{-}R3}$. 14)**. These data suggest that the differential sensitivity correlates with the cellular capacity for CHR-induced TMED3 aggregation. Cells with higher basal TMED3 expression (T3M4 or Miapaca-2) may

reach a threshold of toxic TMED3 aggregation more readily, leading to stronger ER stress and a more robust cytotoxic response. This finding aligns with our proposed mechanism and provides a rational explanation for the observed variability in drug response.

Figure for reviewers removed

2. A systematic analysis of proteins downregulated by CHR (Figure 5 mass spectrometry) should identify how many localize to ER, Golgi, and mitochondria to determine whether CHR's actions are specific or broadly affect organellar function.

RE: Thank you for your suggestions. We have performed the requested analysis. Cellular component enrichment of downregulated proteins in our proteomic data shows no specific enrichment for ER, Golgi, or mitochondria **(Fig[PbP-R3]. 15).** This pattern, coupled with the specific upregulation and aggregation of the ER protein TMED3, supports a targeted r d organellar toxicity.

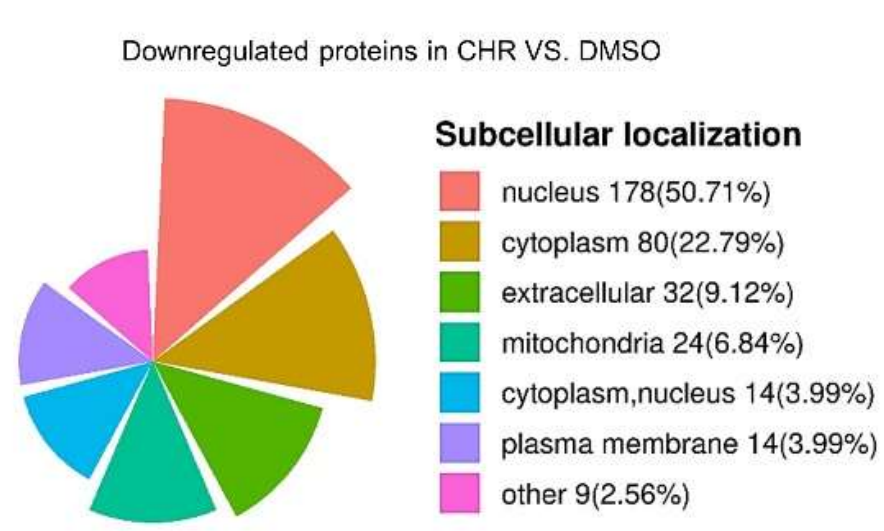

Fig[PbP-R3]. 15 Subcellular localization distribution of downregulated proteins in CHR versus DMSO group.

3. All antibodies and reagents used in the study need detailed description with catalog numbers and RRIDs to ensure reproducibility and facilitate future studies.

RE: The relevant information has been added.

29th Jun 2026

Dear Dr. Zhang,

Thank you for submitting your revised manuscript to EMBO Molecular Medicine. We have now received the reports from referees #1 and #2, who also evaluated the manuscript in light of the concerns raised by referee #3.

As you will see from the reports below, both referees agree that the manuscript has been substantially strengthened during revision and are, overall, supportive of publication. At the same time, both emphasize that the medical implications of the findings should be interpreted with appropriate caution, consistent with the concerns initially raised by referee #3.

We would therefore like to invite you to submit a further revised version of the manuscript. In particular, we ask that you reframe the study in the title, abstract, and discussion to accurately reflect its conceptual advance while avoiding overstating the immediate translational potential of CHR. The manuscript should make clear that the principal advance of the study is the identification of TMED3 as a potentially druggable regulator of ER stress and UPR signaling, which may provide a rationale for future therapeutic strategies, including combination approaches.

We would also welcome any additional experimental work that addresses the remaining concerns raised by referee #2 and further strengthens the mechanistic understanding of the study. Where additional experimental evidence cannot be provided, the corresponding mechanistic claims should be moderated and the study's limitations more explicitly acknowledged.

Please note that, at EMBO Press, we typically allow only one round of major revisions. Accordingly, this will represent the final opportunity to address the remaining concerns raised during peer review. If you would like to discuss the referees' comments or our editorial expectations before preparing your revision, I would be happy to do so by email or via a video call. Please let me know if this would be helpful.

When preparing your revised manuscript, please refer to our guidelines: https://link.springer.com/journal/44321/submission-guidelines#cms-Revised-submissions. We perform an initial quality control of all revised manuscripts before re-review; failure to include requested items will delay the evaluation of your revision.

We require:

1) A .docx formatted version of the manuscript text (including legends for main figures, EV figures and tables). Please make sure that the changes are highlighted to be clearly visible.

2) Individual production quality figure files as .eps, .tif, .jpg (one file per figure). For guidance, download the 'Figure Guide PDF': https://media.springernature.com/original/springer-cms/rest/v1/content/27825798/data/v1.

3) A .docx formatted letter INCLUDING the reviewers' reports and your detailed point-by-point responses to their comments. As part of the EMBO Press transparent editorial process, the point-by-point response is part of the Review Process File (RPF), which will be published alongside your paper.

4) A complete author checklist, which you can download from our author guidelines. Please insert information in the checklist that is also reflected in the manuscript. The completed author checklist will also be part of the RPF.

5) Please note that all corresponding authors are required to supply an ORCID ID for their name upon submission of a revised manuscript.

6) It is mandatory to include a 'Data Availability' section after the Materials and Methods. Before submitting your revision, primary datasets produced in this study need to be deposited in an appropriate public database, and the accession numbers and database listed under 'Data Availability'. Please remember to provide a reviewer password if the datasets are not yet public.

In case you have no data that requires deposition in a public database, please state so in this section. Note that the Data Availability Section is restricted to new primary data that are part of this study.

7) For data quantification: please specify the name of the statistical test used to generate error bars and P values, the number (n) of independent experiments (specify technical or biological replicates) underlying each data point and the test used to calculate p-values in each figure legend. The figure legends should contain a basic description of n, P and the test applied. Graphs must include a description of the bars and the error bars (s.d., s.e.m.).

8) At EMBO Press we ask authors to provide source data for the main manuscript figures. You will receive a separate email with instructions for providing source data with your revised manuscript, including how to upload and organize the files.

9) Our journal encourages inclusion of *data citations in the reference list* to directly cite datasets that were re-used and obtained from public databases. Data citations in the article text are distinct from normal bibliographical citations and should directly link to the database records from which the data can be accessed. In the main text, data citations are formatted as follows: "Data ref: Smith et al, 2001" or "Data ref: NCBI Sequence Read Archive PRJNA342805, 2017". In the Reference list, data citations must be labeled with "[DATASET]". A data reference must provide the database name, accession number/identifiers and a resolvable link to the landing page from which the data can be accessed at the end of the reference.

10) We replaced Supplementary Information with Expanded View (EV) Figures and Tables that are collapsible/expandable online. A maximum of 5 EV Figures can be typeset. EV Figures should be cited as 'Figure EV1, Figure EV2" etc... in the text and their respective legends should be included in the main text after the legends of regular figures.

- For the figures that you do NOT wish to display as Expanded View figures, they should be bundled together with their legends in a single PDF file called *Appendix*, which should start with a short Table of Content. Appendix figures should be referred to in the main text as: "Appendix Figure S1, Appendix Figure S2" etc.

- Additional Tables/Datasets should be labeled and referred to as Table EV1, Dataset EV1, etc. Legends have to be provided in a separate tab in case of .xls files. Alternatively, the legend can be supplied as a separate text file (README) and zipped together with the Table/Dataset file.

11) The paper explained: EMBO Molecular Medicine articles are accompanied by a summary of the articles to emphasize the major findings in the paper and their medical implications for the non-specialist reader. Please provide a draft summary of your article highlighting
- the medical issue you are addressing,
- the results obtained and
- their clinical impact.

This may be edited to ensure that readers understand the significance and context of the research. Please refer to any of our published articles for an example.

12) Author contributions: You will be asked to provide CRediT (Contributor Role Taxonomy) terms in the submission system. These replace a narrative author contribution section in the manuscript.

13) A "Disclosure and Competing Interests Statement" should be provided in the main text.

14) Every published paper includes a 'Synopsis' to further enhance discoverability. Synopses are displayed on the journal webpage and are freely accessible to all readers. They include a short stand first (maximum of 300 characters, including space) as well as 2-5 one-sentences bullet points that summarizes the paper. Please write the bullet points to summarize the key NEW findings. They should be designed to be complementary to the abstract - i.e. not repeat the same text. We encourage inclusion of key acronyms and quantitative information (maximum of 30 words / bullet point). Please use the passive voice. Please attach these in a separate file or send them by email, we will incorporate them accordingly.

Please also suggest a striking image or visual abstract to illustrate your article as a PNG file 550 px wide x 300 px high.

15) Include a Reagents and Tools Table as part of the Methods section, which can be downloaded from our author guidelines.

16) As part of the EMBO Publications transparent editorial process initiative (see our Editorial at http://embomolmed.embopress.org/content/2/9/329), EMBO Molecular Medicine will publish online a Review Process File (RPF) to accompany accepted manuscripts.
In the event of acceptance, this file will be published in conjunction with your paper and will include the anonymous referee reports, your point-by-point response and all pertinent correspondence relating to the manuscript. Let us know whether you agree with the publication of the RPF and as here, if you want to remove or not any figures from it prior to publication.
Please note that the Authors checklist will be published at the end of the RPF.

I look forward to seeing a revised form of your manuscript as soon as possible.

With kind regards,

Lise Roth

Lise Roth, Ph.D
Senior Editor
EMBO Molecular Medicine

***** Reviewer's comments *****

Referee #1 (Comments on Novelty/Model System for Author):

The technical quality is good and has clearly improved in the revised version. The authors added important controls, including TMED3 knockout, mutant validation, dose-response in vivo studies, and toxicity assessment. The statistical analysis appears appropriate, although some in vivo groups remain relatively small.

The general involvement of ER stress, UPR, autophagy, and apoptosis is not entirely novel. However, the identification of the CHR-TMED3 axis as a functional mechanism in pancreatic cancer provides a new and biologically interesting contribution.

The medical impact is potentially relevant but should remain cautiously interpreted. CHR itself is not yet a strong clinical candidate, but the study identifies a potentially druggable TMED3-ER stress vulnerability that may support future combination strategies.

The model systems are appropriate, including pancreatic cancer cell lines, patient-derived organoids, and PDX models. Future studies would benefit from orthotopic or genetically engineered models, longer treatments, PK/PD studies, and more extensive toxicity evaluation.

Referee #1 (Remarks for Author):

The manuscript is now clearer and more accessible, as the revised version presents a coherent mechanistic sequence linking CHR to TMED3 perturbation, ER stress, protective autophagy, and apoptosis. The topic should be of interest beyond pancreatic cancer specialists because it connects natural-product drug discovery with a potentially druggable proteostasis vulnerability.

The revised manuscript is suitable for publication, as the authors have addressed the main mechanistic and translational concerns with substantial new data. Given the breadth of experimental approaches, including cell lines, PDOs, PDXs, target validation, and mechanistic rescue experiments, I believe it is better suited as a full article rather than a short report.

Referee #2 (Comments on Novelty/Model System for Author):

The study employs a broad set of experimental approaches across appropriate in vitro and in vivo model systems. The work is technically solid.

However, the overall novelty should be considered as medium. The principal advance of the study is not the development of CHR as a therapeutic candidate per se, but rather the identification of TMED3 as a potentially druggable upstream regulator of ER stress and UPR signaling. While CHR serves as a useful chemical probe to uncover this biology, its pharmacological properties and preclinical efficacy do not yet establish it as a near-clinical therapeutic agent. In this regard, the conceptual contribution of the manuscript lies primarily in the mechanistic insight into TMED3 biology rather than in the translational development of CHR itself.

Referee #2 (Remarks for Author):

The authors have addressed all my concerns to my satisfaction and have substantially strengthened the manuscript. In particular, the additional genetic validation using TMED3 knockout models, mutagenesis studies, and the expanded biochemical characterization considerably improve the evidence supporting TMED3 as a direct target of CHR.

However, one important mechanistic aspect remains insufficiently resolved. While the authors demonstrate that CHR binds TMED3, promotes TMED3 accumulation/aggregation, and is associated with activation of the UPR, the causal link between TMED3 aggregation and activation of the canonical UPR sensors remains largely speculative.

The current data primarily rely on downstream markers such as p-IRE1α, XBP1s, p-PERK, and CHOP. While these findings clearly demonstrate UPR activation, they do not explain mechanistically how TMED3 aggregation initiates ER stress signaling.

Several important questions therefore remain unanswered:
What is the molecular mechanism by which TMED3 aggregation activates IRE1α and/or PERK?
Are PERK and IRE1α activated directly or indirectly through secondary perturbations of ER homeostasis?
Does CHR-induced TMED3 aggregation induce RIDD activity?
Is XBP1 functionally necessary for the observed phenotype?

The authors propose an interesting model involving altered vesicular trafficking, cytoskeletal remodeling, and recruitment of stress-associated proteins. However, this remains hypothetical and is not directly tested experimentally.

Related to this point, TMED3 is primarily known as a regulator of ER-Golgi trafficking and cargo transport. This raises the question what I consider a central mechanistic question of the study: How does CHR-mediated TMED3 aggregation affect the secretory pathway? Demonstrating alterations in ER-to-Golgi trafficking, cargo transport, protein secretion, Golgi organization, or related secretory functions would substantially strengthen the proposed model and provide a direct mechanistic connection between TMED3 perturbation and ER stress induction.

Personally, I am not a big fan of too many rounds of revision and therefore I do not consider these additional experiments essential for publication, particularly given the substantial improvements made during revision and the satisfactory responses to most of the reviewers' concerns. However, I would encourage the authors to moderate some of the mechanistic claims and discuss more explicitly that the precise molecular connection between TMED3 aggregation, secretory pathway dysfunction, and activation of the UPR remains to be established.

Point-by-point response

The revised sections of the manuscript are marked in blue.

Referee #1 (Comments on Novelty/Model System for Author):

The technical quality is good and has clearly improved in the revised version. The authors added important controls, including TMED3 knockout, mutant validation, dose-response in vivo studies, and toxicity assessment. The statistical analysis appears appropriate, although some in vivo groups remain relatively small.

The general involvement of ER stress, UPR, autophagy, and apoptosis is not entirely novel. However, the identification of the CHR-TMED3 axis as a functional mechanism in pancreatic cancer provides a new and biologically interesting contribution.

The medical impact is potentially relevant but should remain cautiously interpreted. CHR itself is not yet a strong clinical candidate, but the study identifies a potentially druggable TMED3-ER stress vulnerability that may support future combination strategies.

The model systems are appropriate, including pancreatic cancer cell lines, patient-derived organoids, and PDX models. Future studies would benefit from orthotopic or genetically engineered models, longer treatments, PK/PD studies, and more extensive toxicity evaluation.

RE: We appreciate the positive comments and constructive suggestions. We have further emphasized the originality of the newly discovered CHR-TMED3 axis and revised the discussion to moderate the translational expectation of CHR. In addition, we have acknowledged the limitations of our current experimental models. Orthotopic tumor models, PK/PD analysis and systematic toxicity assessment will be implemented in our future investigations.

Referee #1 (Remarks for Author):

The manuscript is now clearer and more accessible, as the revised version presents a coherent mechanistic sequence linking CHR to TMED3 perturbation, ER stress, protective autophagy, and apoptosis. The topic should be of interest beyond pancreatic cancer specialists because it connects natural-product drug discovery with a potentially druggable proteostasis vulnerability.

The revised manuscript is suitable for publication, as the authors have addressed the main mechanistic and translational concerns with substantial new data. Given the breadth of experimental approaches, including cell lines, PDOs, PDXs, target validation, and mechanistic rescue experiments, I believe it is better suited as a full article rather than a short report.

RE: Thank you so much for your comments.

Referee #2 (Comments on Novelty/Model System for Author):

The study employs a broad set of experimental approaches across appropriate in vitro and in vivo model systems. The work is technically solid. However, the overall novelty should be considered as medium. The principal advance of the study is not the development of CHR as a therapeutic candidate per se, but rather the identification of TMED3 as a potentially druggable upstream regulator of ER stress and UPR signaling. While CHR serves as a useful chemical probe to uncover this biology, its pharmacological properties and preclinical efficacy do not yet establish it as a near-clinical therapeutic agent. In this regard, the conceptual contribution of the manuscript lies primarily in the mechanistic insight into TMED3 biology rather than in the translational development of CHR itself.

RE: We appreciate your careful assessment. We agree that the core innovation of this work is the discovery of TMED3 as a druggable regulator of ER stress and UPR pathways. We have revised the manuscript to clarify that CHR is mainly used as a chemical probe, and toned down the translational claims for CHR as a clinical candidate, focusing our discussion on the mechanistic findings of TMED3.

Referee #2 (Remarks for Author):

The authors have addressed all my concerns to my satisfaction and have substantially strengthened the manuscript. In particular, the additional genetic validation using TMED3 knockout models, mutagenesis studies, and the expanded biochemical characterization considerably improve the evidence supporting TMED3 as a direct target of CHR.

However, one important mechanistic aspect remains insufficiently resolved. While the authors demonstrate that CHR binds TMED3, promotes TMED3 accumulation/aggregation, and is associated with activation of the UPR, the causal link

between TMED3 aggregation and activation of the canonical UPR sensors remains largely speculative.

The current data primarily rely on downstream markers such as p-IRE1α, XBP1s, p-PERK, and CHOP. While these findings clearly demonstrate UPR activation, they do not explain mechanistically how TMED3 aggregation initiates ER stress signaling.

Several important questions therefore remain unanswered: What is the molecular mechanism by which TMED3 aggregation activates IRE1α and/or PERK? Are PERK and IRE1α activated directly or indirectly through secondary perturbations of ER homeostasis? Does CHR-induced TMED3 aggregation induce RIDD activity? Is XBP1 functionally necessary for the observed phenotype?

The authors propose an interesting model involving altered vesicular trafficking, cytoskeletal remodeling, and recruitment of stress-associated proteins. However, this remains hypothetical and is not directly tested experimentally.

Related to this point, TMED3 is primarily known as a regulator of ER-Golgi trafficking and cargo transport. This raises the question what I consider a central mechanistic question of the study: How does CHR-mediated TMED3 aggregation affect the secretory pathway? Demonstrating alterations in ER-to-Golgi trafficking, cargo transport, protein secretion, Golgi organization, or related secretory functions would substantially strengthen the proposed model and provide a direct mechanistic connection between TMED3 perturbation and ER stress induction.

Personally, I am not a big fan of too many rounds of revision and therefore I do not consider these additional experiments essential for publication, particularly given the substantial improvements made during revision and the satisfactory responses to most of the reviewers' concerns. However, I would encourage the authors to moderate some of the mechanistic claims and discuss more explicitly that the precise molecular

connection between TMED3 aggregation, secretory pathway dysfunction, and activation of the UPR remains to be established.

RE: We sincerely appreciate your thorough review and positive decision for publication. We have appropriately moderated our mechanistic conclusions in the revised manuscript. We now explicitly state that the precise molecular cascade connecting TMED3 aggregation, secretory pathway disruption and UPR activation requires further verification. All the insightful questions you raised, including how aggregated TMED3 triggers IRE1α/PERK signaling, the involvement of XBP1 and RIDD activity, as well as changes in ER-Golgi cargo trafficking, will form the core directions of our follow-up research. We will systematically investigate these mechanistic gaps in our future work.

17th Jul 2026

Dear Dr. Zhang,

Thank you for submitting your revised manuscript. I will be pleased to accept your manuscript once the following editorial points have been addressed:

1/ Manuscript text:
- Please accept the previous changes, and only keep in track changes any new modification.
- Please note that all corresponding authors are required to supply an ORCID ID for their name upon submission of a revised manuscript. An ORCID identifier is currently missing for Qi Zhang.
- Please change the heading from "Materials and Methods" to "Methods":
o Antibodies: please provide dilutions/concentrations
o Patient-derived tissues are mentioned in two places: please clarify if informed consent was obtained for all patients, and include the full statement that the experiments conformed to the principles set out in the WMA Declaration of Helsinki and the Department of Health and Human Services Belmont Report.
o Clarify the following sentence: "After a certain number of days, a certain concentration of the chemical agents was added to the organoid complete medium".
o Please provide a statement on randomization.
o BIORENDER: remove from Acknowledgments and add as a dedicated Graphics section to the Methods using this format:
Graphics:
(some of the... OR Figure #... OR synopsis) Graphics were created with BioRender.com.
o Remove the Reagents and Tools table from the manuscript file.
- Data Availability Section: please provide specific URLs for your deposited datasets, and kindly note that they must be public before acceptance of the manuscript.

2/ Figures:
- Appendix Figure S3F and Appendix Figure S4C are highly similar, please clarify. Note that figure re-use is allowed, but should be mentioned in the figure legend.
- You are welcome to make some of your appendix figures into EV figures, to make them more accessible to readers. For the figures that you do NOT wish to display as Expanded View figures, they should be bundled together with their legends in a single PDF file called *Appendix*, which should start with a short Table of Content. Appendix figures should be referred to in the main text as: "Appendix Figure S1, Appendix Figure S2" etc.
- Table EV1 should be renamed Dataset EV1.
- Please check the callouts for all figures and figure panels. Callouts are currently missing for Fig 7A, B.
- Please address the queries from our data editors in the figure legends:
1. Please indicate the statistical test used for data analysis in the legends of figures 2A,B; 5B; EV-1A,B.
2. Please note that information related to n is missing in the legends of figures 2A; EV-1A.
3. Although 'n' is provided, please describe the nature of entity for 'n' in the legends of 1D,F; 2H; 4A,D,H; 5G,I; 6D,G; 7B; EV-2L; EV-3B,D,F.

3/ Checklist:
- Please fill in the section 'Experimental study design and statistics', subsection 'sample size'.
- Please check the section 'Ethics', subsection 'studies involving speciment and field samples', as I don't think it applies to your study

4/ Source Data:
- Please provide the raw image data for all the main figure microscopy images.
- Please provide the raw facs data.
- Please note that Figure 1D excel file is empty.
- Source data for figure 5B is missing.

5/ Thank you for providing the Paper Explained. Please include it in the manuscript text file, before the introduction.

6/ I introduced minor edits in your synopsis, please let me know if you agree or amend as you see fit:

Chrysosplenetin (CHR) induces TMED3 aggregation, causing lethal ER stress in pancreatic cancer and identifying TMED3 as a direct target and druggable vulnerability. CHR combined with gemcitabine or autophagy inhibitors enhances antitumor activity in preclinical models.
- Phenotypic screening identifies CHR as a bioactive compound with anti-pancreatic cancer activity.
- CHR induces ER stress-mediated apoptosis but also triggers protective autophagy, which limits its antitumor activity.
- TMED3 is validated as a direct target of CHR, linking its disruption to UPR activation and apoptosis.

- Combining CHR with gemcitabine or autophagy inhibitors enhances antitumor activity in PDX and PDO models.

Please resize your visual abstract to illustrate your article as a PNG file 550 px wide x 300 px high.

7/ As part of the EMBO Publications transparent editorial process initiative (see our Editorial at http://embomolmed.embopress.org/content/2/9/329), EMBO Molecular Medicine will publish online a Review Process File (RPF) to accompany accepted manuscripts.
This file will be published in conjunction with your paper and will include the anonymous referee reports, your point-by-point response and all pertinent correspondence relating to the manuscript. Let us know whether you agree with the publication of the RPF and as here, if you want to remove or not any figures from it prior to publication.
Please note that the Authors checklist will be published at the end of the RPF.

I look forward to receiving your revised manuscript.

Yours sincerely,

Lise Roth

Lise Roth, PhD
Senior Editor
EMBO Molecular Medicine

The authors addressed the remaining editorial issues.

30th Jul 2026

Dear Dr. Zhang,

Thank you for submitting your revised files. I am pleased to inform you that your manuscript is accepted for publication and is now being sent to our publisher to be included in the next available issue of EMBO Molecular Medicine.

Please note that a URL is missing for the deposited proteomic dataset and should be added at proof stage.

Your manuscript will be processed for publication by EMBO Press. It will be copy edited and you will receive page proofs prior to publication. Please note that you will be contacted by Springer Nature Author Services to complete licensing and payment information.

You may qualify for financial assistance for your publication charges - either via a Springer Nature fully open access agreement or an EMBO initiative. Check your eligibility: https://link.springer.com/journal/44321/how-to-publish-with-us

Should you be planning a Press Release on your article, please notify the production team via this form in order to coordinate publication and release dates: https://forms.cloud.microsoft/e/eH4A8JVP6g

If you have any questions, please do not hesitate to contact the Editorial Office. Thank you for your contribution to EMBO Molecular Medicine.

Yours sincerely,

Lise Roth

Lise Roth, Ph.D
Senior Editor
EMBO Molecular Medicine

>>> Please note that it is EMBO Molecular Medicine policy for the transcript of the editorial process (containing referee reports and your response letter) to be published as an online supplement to each paper. If you do NOT want this, you will need to inform the Editorial Office via email immediately. More information is available here: https://link.springer.com/partners/embo-press/editorial-policies#Peer%20review

EMBO Press Author Checklist

Corresponding Author Name: Zhe Zhang	
Journal Submitted to: EMBO Molecular Medcine	
Manuscript Number: EMM-2025-22549	

USEFUL LINKS FOR COMPLETING THIS FORM
The EMBO Journal - Author Guidelines
EMBO Reports - Author Guidelines
Molecular Systems Biology - Author Guidelines
EMBO Molecular Medicine - Author Guidelines

Reporting Checklist for Life Science Articles (updated January
This checklist is adapted from Materials Design Analysis Reporting (MDAR) Checklist for Authors. MDAR establishes a minimum set of requirements in transparent reporting in the life sciences (see Statement of Task: 10.31222/osf.io/9sm4x). Please follow the journal's guidelines in preparing your
Please note that a copy of this checklist will be published alongside your article.

Abridged guidelines for figures
1. Data
The data shown in figures should satisfy the following conditions:
- the data were obtained and processed according to the field's best practice and are presented to reflect the results of the experiments in an accurate and unbiased manner.
- ideally, figure panels should include only measurements that are directly comparable to each other and obtained with the same assay.
- plots include clearly labeled error bars for independent experiments and sample sizes. Unless justified, error bars should not be shown for technical
- if n<5, the individual data points from each experiment should be plotted. Any statistical test employed should be justified.
- Source Data should be included to report the data underlying figures according to the guidelines set out in the authorship guidelines on Data

2. Captions
Each figure caption should contain the following information, for each panel where they are relevant:
- a specification of the experimental system investigated (eg cell line, species name).
- the assay(s) and method(s) used to carry out the reported observations and measurements.
- an explicit mention of the biological and chemical entity(ies) that are being measured.
- an explicit mention of the biological and chemical entity(ies) that are altered/varied/perturbed in a controlled manner.
- the exact sample size (n) for each experimental group/condition, given as a number, not a range;
- a description of the sample collection allowing the reader to understand whether the samples represent technical or biological replicates (including how many animals, litters, cultures, etc.).
- a statement of how many times the experiment shown was independently replicated in the laboratory.
- definitions of statistical methods and measures:
  - common tests, such as t-test (please specify whether paired vs. unpaired), simple χ2 tests, Wilcoxon and Mann-Whitney tests, can be unambiguously identified by name only, but more complex techniques should be described in the methods section;
  - are tests one-sided or two-sided?
  - are there adjustments for multiple comparisons?
  - exact statistical test results, e.g., P values = x but not P values < x;
  - definition of 'center values' as median or average;
  - definition of error bars as s.d. or s.e.m.

Please complete ALL of the questions below.
Select "Not Applicable" only when the requested information is not relevant for your study.

Materials

Newly Created Materials	Information included in the manuscript?	In which section is the information available? (Reagents and Tools Table, Materials and Methods, Figures, Data Availability Section)
New materials and reagents need to be available; do any restrictions apply?	Not Applicable	

Antibodies	Information included in the manuscript?	In which section is the information available? (Reagents and Tools Table, Materials and Methods, Figures, Data Availability Section)
For **antibodies** provide the following information:<br>- Commercial antibodies: RRID (if possible) or supplier name, catalogue number and or/clone number<br>- Non-commercial: RRID or citation	Yes	Reagents and Tools Table, or Materials and Methods

DNA and RNA sequences	Information included in the manuscript?	In which section is the information available? (Reagents and Tools Table, Materials and Methods, Figures, Data Availability Section)
**Short novel DNA or RNA including primers, probes**: provide the sequences.	Yes	Reagents and Tools Table, or Materials and Methods

Cell materials	Information included in the manuscript?	In which section is the information available? (Reagents and Tools Table, Materials and Methods, Figures, Data Availability Section)
**Cell lines:** Provide species information, strain. Provide accession number in repository **OR** supplier name, catalog number, clone number, and/**OR** RRID.	Yes	Materials and Methods
**Primary cultures:** Provide species, strain, sex of origin, genetic modification status.	Yes	Materials and Methods
Report if the cell lines were recently **authenticated** (e.g., by STR profiling) and tested for mycoplasma contamination.	Yes	Materials and Methods

Experimental animals	Information included in the manuscript?	In which section is the information available? (Reagents and Tools Table, Materials and Methods, Figures, Data Availability Section)
**Laboratory animals or Model organisms:** Provide species, strain, sex, age, genetic modification status. Provide accession number in repository **OR** supplier name, catalog number, clone number, **OR** RRID.	Yes	Materials and Methods
**Animal observed in or captured from the field:** Provide species, sex, and age where possible.	Not Applicable	
Please detail **housing and husbandry conditions**.	Yes	Materials and Methods

Plants and microbes	Information included in the manuscript?	In which section is the information available? (Reagents and Tools Table, Materials and Methods, Figures, Data Availability Section)
**Plants:** provide species and strain, ecotype and cultivar where relevant, unique accession number if available, and source (including location for collected wild specimens).	Not Applicable	
**Microbes:** provide species and strain, unique accession number if available, and source.	Not Applicable	

Human research participants	Information included in the manuscript?	In which section is the information available? (Reagents and Tools Table, Materials and Methods, Figures, Data Availability Section)
If collected and within the bounds of privacy constraints report on age, sex and gender or ethnicity for all study participants.	Yes	Materials and Methods

Core facilities	Information included in the manuscript?	In which section is the information available? (Reagents and Tools Table, Materials and Methods, Figures, Data Availability Section)
If your work benefited from core facilities, was their service mentioned in the acknowledgments section?	Yes	Materials and Methods

Design

Study protocol	Information included in the manuscript?	In which section is the information available? (Reagents and Tools Table, Materials and Methods, Figures, Data Availability Section)
If study protocol has been **pre-registered, provide DOI in the manuscript**. For clinical trials, provide the trial registration number **OR** cite DOI.	Not Applicable	
Report the **clinical trial registration number** (at ClinicalTrials.gov or equivalent), where applicable.	Not Applicable	

Laboratory protocol	Information included in the manuscript?	In which section is the information available? (Reagents and Tools Table, Materials and Methods, Figures, Data Availability Section)
Provide DOI OR other citation details if **external detailed step-by-step protocols** are available.	Yes	Materials and Methods

Experimental study design and statistics	Information included in the manuscript?	In which section is the information available? (Reagents and Tools Table, Materials and Methods, Figures, Data Availability Section)
Include a statement about **sample size** estimate even if no statistical methods were used.	Yes	Figure legends
Were any steps taken to minimize the effects of subjective bias when allocating animals/samples to treatment (e.g. **randomization procedure**)? If yes, have they been described?	Yes	Materials and Methods
Include a statement about **blinding** even if no blinding was done.	Yes	Materials and Methods
Describe **inclusion/exclusion criteria** if samples or animals were excluded from the analysis. Were the criteria pre-established?<br><br>If sample or data points were omitted from analysis, report if this was due to attrition or intentional exclusion and provide justification.	Yes	Materials and Methods
For every figure, are **statistical tests** justified as appropriate? Do the data meet the assumptions of the tests (e.g., normal distribution)? Describe any methods used to assess it. Is there an estimate of variation within each group of data? Is the variance similar between the groups that are being statistically compared?	Yes	Materials and Methods

Sample definition and in-laboratory replication	Information included in the manuscript?	In which section is the information available? (Reagents and Tools Table, Materials and Methods, Figures, Data Availability Section)
In the figure legends: state number of times the experiment was **replicated** in laboratory.	Yes	Figures (figure legends)
In the figure legends: define whether data describe **technical or biological replicates**.	Yes	Figures (figure legends)

Ethics

Ethics	Information included in the manuscript?	In which section is the information available? (Reagents and Tools Table, Materials and Methods, Figures, Data Availability Section)
Studies involving **human participants**: State details of **authority granting ethics approval** (IRB or equivalent committee(s), provide reference number for approval.	Yes	Materials and Methods
Studies involving **human participants**: Include a statement confirming that **informed consent** was obtained from all subjects and that the experiments conformed to the principles set out in the WMA Declaration of Helsinki and the Department of Health and Human Services Belmont Report.	Yes	Materials and Methods
Studies involving **human participants: For** publication of **patient photos**, include a statement confirming that consent to publish was obtained.	Not Applicable	
Studies involving experimental **animals**: State details of **authority granting ethics approval** (IRB or equivalent committee(s), provide reference number for approval. Include a statement of compliance with ethical regulations.	Yes	Materials and Methods
Studies involving **specimen and field samples:** State if relevant **permits** obtained, provide details of authority approving study; if none were required, explain why.	Not Applicable	

Dual Use Research of Concern (DURC)	Information included in the manuscript?	In which section is the information available? (Reagents and Tools Table, Materials and Methods, Figures, Data Availability Section)
Could your study fall under dual use research restrictions? Please check biosecurity documents and list of **select agents and toxins** (CDC): https://www.selectagents.gov/sat/list.htm	Not Applicable	
If you used a select agent, is the security level of the lab appropriate and reported in the manuscript?	Not Applicable	
If a study is subject to dual use research of concern regulations, is the name of the **authority granting approval and reference number** for the regulatory approval provided in the manuscript?	Not Applicable	

Reporting

The MDAR framework recommends adoption of discipline-specific guidelines, established and endorsed through community initiatives. Journals have their own policy about requiring specific guidelines and recommendations to complement MDAR.

Adherence to community standards	Information included in the manuscript?	In which section is the information available? (Reagents and Tools Table, Materials and Methods, Figures, Data Availability Section)
State if relevant guidelines or checklists (e.g., **ICMJE, MIBBI, ARRIVE, PRISMA**) have been followed or provided.	Yes	Materials and Methods
For **tumor marker prognostic studies,** we recommend that you follow the **REMARK** reporting guidelines (see link list at top right). See author guidelines, under 'Reporting Guidelines'. Please confirm you have followed these guidelines.	Not Applicable	
For **phase II and III randomized controlled trials**, please refer to the **CONSORT** flow diagram (see link list at top right) and submit the CONSORT checklist (see link list at top right) with your submission. See author guidelines, under 'Reporting Guidelines'. Please confirm you have submitted this list.	Not Applicable	

Data Availability

Data availability	Information included in the manuscript?	In which section is the information available? (Reagents and Tools Table, Materials and Methods, Figures, Data Availability Section)
Have **primary datasets** been deposited according to the journal's guidelines (see 'Data Deposition' section) and the respective accession numbers provided in the Data Availability Section?	Yes	Materials and Methods
Were **human clinical and genomic datasets** deposited in a public access-controlled repository in accordance to ethical obligations to the patients and to the applicable consent agreement?	Not Applicable	
Are **computational models** that are central and integral to a study available without restrictions in a machine-readable form? Were the relevant accession numbers or links provided?	Not Applicable	
If publicly available data were reused, provide the respective **data citations in the reference list**.	Yes	Results and Reference Section
